# Effectiveness of SGLT2 inhibitors, incretin-based therapies, and finerenone on cardiorenal outcomes: a meta-analysis and network meta-analysis

**DOI:** 10.1186/s40842-025-00248-2

**Published:** 2025-12-08

**Authors:** Arveen Shokravi, Jayant Seth, Nelson Lu, G. B. John Mancini

**Affiliations:** 1 Department of Medicine, University of British Columbia, Vancouver, BC V6T 1Z4 Canada; 2 Centre for Cardiovascular Innovation, Division of Cardiology, Department of Medicine, University of British Columbia, Vancouver, BC V5Z 1M9 Canada

## Abstract

**Background:**

Several societies recommend sodium-glucose co-transporter 2 inhibitors (SGLT2i), glucagon-like peptide-1 receptor agonists (GLP-1RA), and finerenone for cardiorenal risk reduction in select populations. This updated meta-analysis assessed the impact of SGLT2i, incretin-based therapies (i.e. GLP-1RAs and tirzepatide), and finerenone on cardiorenal outcomes in established and emerging populations. Additionally, a network meta-analysis (NMA) compared the relative efficacy between treatment classes.

**Methods:**

A systematic search of MEDLINE and CENTRAL from January 2023 to April 2025, supplemented by studies evaluated in our prior meta-analyses, identified 33 randomized controlled trials. Random-effects models were used to generate hazard ratios for outcomes including cardiovascular (CV) mortality, all-cause mortality, heart failure (HF) hospitalization/event, non-fatal myocardial infarction (MI), non-fatal stroke, and kidney composite outcomes in various subpopulations including patients with type 2 diabetes (T2D) with atherosclerotic cardiovascular disease (ASCVD) or high CV risk, chronic kidney disease (CKD), HF with reduced ejection fraction (HFrEF), HF with preserved ejection fraction (HFpEF), HFpEF with obesity, post-MI, acute HF, and ASCVD with overweight/obesity without T2D. NMA was performed when two or more treatment classes were reported for a given outcome within a subpopulation.

**Results:**

Incretin-based therapies decreased CV mortality, all-cause mortality, non-fatal MI, and kidney composite outcomes in CKD, and reduced these outcomes as well as HF hospitalization/events and non-fatal stroke in T2D with ASCVD/high CV risk. In HFpEF with obesity, incretin-based therapies reduced HF hospitalization/events. SGLT2i reduced CV mortality, all-cause mortality, HF hospitalization/events, and kidney composite outcomes in HFrEF, and reduced these outcomes as well as non-fatal MI in CKD and T2D with ASCVD/high CV risk. SGLT2i decreased HF hospitalization/events in HFpEF, and lowered HF hospitalizations in post-MI and acute HF populations. Finerenone reduced HF hospitalizations and kidney composite outcomes in diabetic CKD and reduced HF hospitalizations in HFpEF. Using placebo as the common comparator in the NMA, SGLT2i conferred significantly greater reductions in HF hospitalization/events and kidney composite outcomes compared to incretin-based therapies in T2D with ASCVD/high CV risk and CKD.

**Conclusions:**

These findings confirm the role of SGLT2i, incretin-based therapies, and finerenone in cardiorenal risk reduction across established high-risk groups, including T2D and CKD, and extend benefits to newer populations, including acute HF and post-MI for SGLT2i, and HFpEF with obesity for incretin-based therapies. Indirect NMA evidence further suggests SGLT2i may reduce HF hospitalization/events and kidney composite outcomes more than incretin-based therapies in T2D with ASCVD/high CV risk and CKD populations.

**Graphical Abstract:**

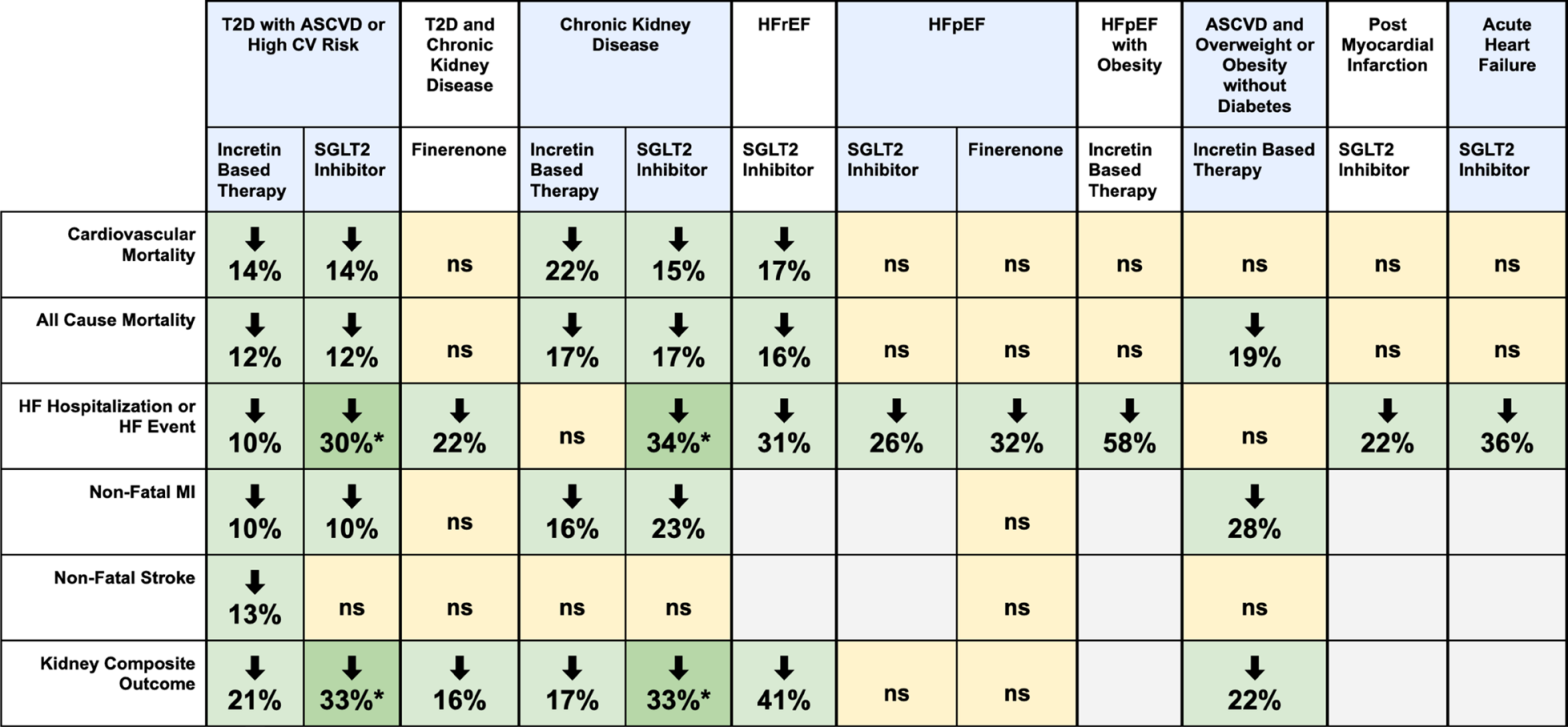

**Supplementary Information:**

The online version contains supplementary material available at 10.1186/s40842-025-00248-2.

## Introduction

In recent years, major international cardiology societies—including the American Heart Association (AHA), American College of Cardiology (ACC), Canadian Cardiovascular Society (CCS), and European Society of Cardiology (ESC)—have recommended the use of sodium-glucose co-transporter 2 inhibitors (SGLT2i) and glucagon-like peptide-1 receptor agonists (GLP-1RA) to reduce cardiorenal morbidity and mortality, with specific recommendations based on distinct patient populations [1–5]. These recommendations have been informed by multiple meta-analyses and an expanding body of randomized controlled trial (RCT) evidence. More recently, the non-steroidal mineralocorticoid receptor antagonist (MRA) finerenone has been incorporated into treatment guidelines by certain societies [5, 6] following evidence supporting its benefit in certain subpopulations, particularly patients with diabetic kidney disease (DKD) [7, 8].

Past meta-analyses have examined the role of SGLT2i and GLP-1RA on cardiorenal outcomes in diverse populations, including those with chronic kidney disease (CKD), heart failure (HF) stratified based on preserved (HFpEF, including persons with HF and mid-range ejection fraction [HFmrEF]) or reduced ejection fraction (HFrEF), and type 2 diabetes (T2D) [9–13]. Since these analyses, there has been an expansion not only in the number of studies, but also in clinical trials assessing newer medications and involving broader patient populations. This includes trials evaluating finerenone in HFpEF, SGLT2i in post-myocardial infarction (MI) populations, and incretin-based therapies in emerging populations, including individuals with HFpEF and obesity, or atherosclerotic cardiovascular disease (ASCVD) and overweight/obesity without T2D.

The goal of this study was to provide an updated meta-analysis on key cardiorenal outcomes in RCTs using SGLT2i, incretin-based therapies, and finerenone across diverse subpopulations. While prior meta-analyses [11–13] primarily focused on patients with T2D and CKD, the present work expands upon these efforts by incorporating newly published RCTs and extending the evaluation to additional subpopulations—including patients with T2D with ASCVD or high cardiovascular (CV) risk, CKD, HFrEF, HFpEF, HFpEF with obesity, post-MI, acute HF, and ASCVD with overweight/obesity without T2D—in order to inform clinical decision-making in a broader spectrum of patients at risk for cardiorenal events. To supplement the findings, a network meta-analysis (NMA) was conducted to compare the relative efficacy of treatment classes.

## Methods

This review was conducted in accordance with the 2020 Preferred Reporting Items for Systematic Reviews and Meta-Analyses (PRISMA) guidelines [14]. This review directly builds upon two prior meta-analyses conducted by one of our co-authors, adding new evidence from 11 RCTs to their previous work [9, 10]. The methodological approach for this review largely reflects these prior studies, with the addition of further statistical analysis to explore consistency and robustness of the results.

### Search strategy, study selection, data extraction

A comprehensive search of MEDLINE and the Cochrane Central Register of Controlled Trials (CENTRAL) was conducted from January 2023, the final search date of our previous analysis [10], through April 2025. A full search strategy is included in the supplementary appendix.

References were uploaded to Covidence electronically merged to eliminate duplicates [15]. Two investigators screened abstracts to determine if they met inclusion criteria. Inclusion criteria included RCTs that examined the effect of SGLT2i, incretin-based therapies (including GLP-1RAs and glucose-dependent insulinotropic polypeptide [GIP]/GLP-1 receptor agonists, excluding dipeptidyl peptidase-4 [DPP-4] inhibitors), or finerenone on one or more cardiorenal outcomes of interest in adults (age ≥ 18 years). DPP-4 inhibitors were excluded from this analysis because major cardiovascular outcome trials have demonstrated non-superiority compared with placebo for major adverse cardiovascular events [3]. Outcomes of interest included: cardiovascular (CV) mortality, all-cause mortality, time to first HF hospitalization or HF event (typically defined as hospitalization, urgent care visit, or oral diuretic intensification), non-fatal MI, non-fatal stroke, major adverse cardiovascular events (MACE), composite of time to first CV death or HF hospitalization/event, and kidney composite outcome. The kidney composite outcome generally includes a combination of decline in estimated glomerular filtration rate (eGFR), development of end-stage kidney disease, need for dialysis or kidney transplant, or death due to renal causes.

Exclusion criteria included studies with no placebo group, fewer than 500 patients, and with follow-up times less than 10 months. Two investigators independently reviewed full texts and extracted study data, with discrepancies resolved by consensus or, when needed, with adjudication by a third reviewer.

All analyses were stratified by subpopulations of interest to enable subgroup analyses and generate more clinically relevant hazard ratios (HRs). These included patients with T2D and ASCVD or high CV risk, CKD, HFrEF, HFpEF, HFpEF with obesity, post-MI, acute HF, and ASCVD with overweight/obesity without T2D.

### Statistical methods

Meta-analyses were conducted with random effects models using the meta package on RStudio [16]. Given that this review builds upon prior meta-analyses that employed the DerSimonian and Laird (DL) random-effects model, we selected the same model for consistency and comparability for the primary analyses. The Cochrane Handbook for meta-analyses notes that DL is prone to under-estimating between-study variance and may yield narrow confidence intervals (CI), and for which studies have demonstrated superior performance of alternative estimators [17]. Accordingly, we repeated all analyses with restricted maximum-likelihood (REML) estimation and applied the modified Hartung-Knapp (HK) adjustment. A flaw of the HK adjustment is that it can yield overly wide CIs when only a few studies are available and or occasionally overly narrow CIs when between-study variance is 0%. In such cases, there is no universally optimal solution, therefore Cochrane recommends doing sensitivity analysis with multiple statistical models [17]. Whenever there were three or fewer studies or between-study variance (i.e. tau-squared) was 0%, we additionally analysed the data using REML with Wald-type CIs to provide a more balanced estimate of uncertainty.

We conducted a network meta-analysis (NMA) to indirectly compare efficacy of treatment classes, using placebo as the reference. NMA was performed when at least two distinct treatment classes were represented for a given outcome within a defined population subgroup. A random-effects model was fitted using the REML estimator to account for between-study variance and treatment effect estimates were expressed as HRs with 95% CI with HK adjustment. Network diagrams were generated and treatment effects relative to placebo were summarized in forest plots. Because all included trials were placebo-controlled, comparisons between treatment classes were derived through indirect evidence using placebo as the common comparator. All analyses were conducted in RStudio using the netmeta package [18].

Forest plots displayed hazard ratios (HR) with 95% CIs, and a p-value less than 0.05 was considered statistically significant. Presence of heterogeneity was assessed using the chi-squared (Cochran’s Q) test (α = 0.05), and its magnitude was quantified using the I² statistic, where I² < 25% indicated low, 25% to 49% indicated moderate, and 50% or greater indicated substantial heterogeneity. Between-study variance was quantified using tau-squared (τ²). Publication bias was assessed using funnel plots and Egger’s regression test when more than 10 studies were included, with asymmetry defined as *p* < 0.05 suggesting potential small-study effects.

### Quality of evidence and GRADE

The Cochrane Risk of Bias 2.0 (RoB 2.0) tool was used to assess the methodological quality of included trials. The Grading of Recommendations, Assessment, Development and Evaluations (GRADE) framework was used to assess the certainty of evidence across the outcomes in the meta-analysis. Each outcome was independently assessed by two reviewers. The GRADE assessment within the ‘impression’ component was informed by multiple factors, including the precision, risk of bias, inconsistency, indirectness, and publication bias. Although multiple statistical models were used for sensitivity analyses, GRADE ratings were derived from results using the DL method to maintain consistency with the previous meta-analyses this work builds on.

## Results

### Overview of included studies and trial populations

**Fig. 1:**
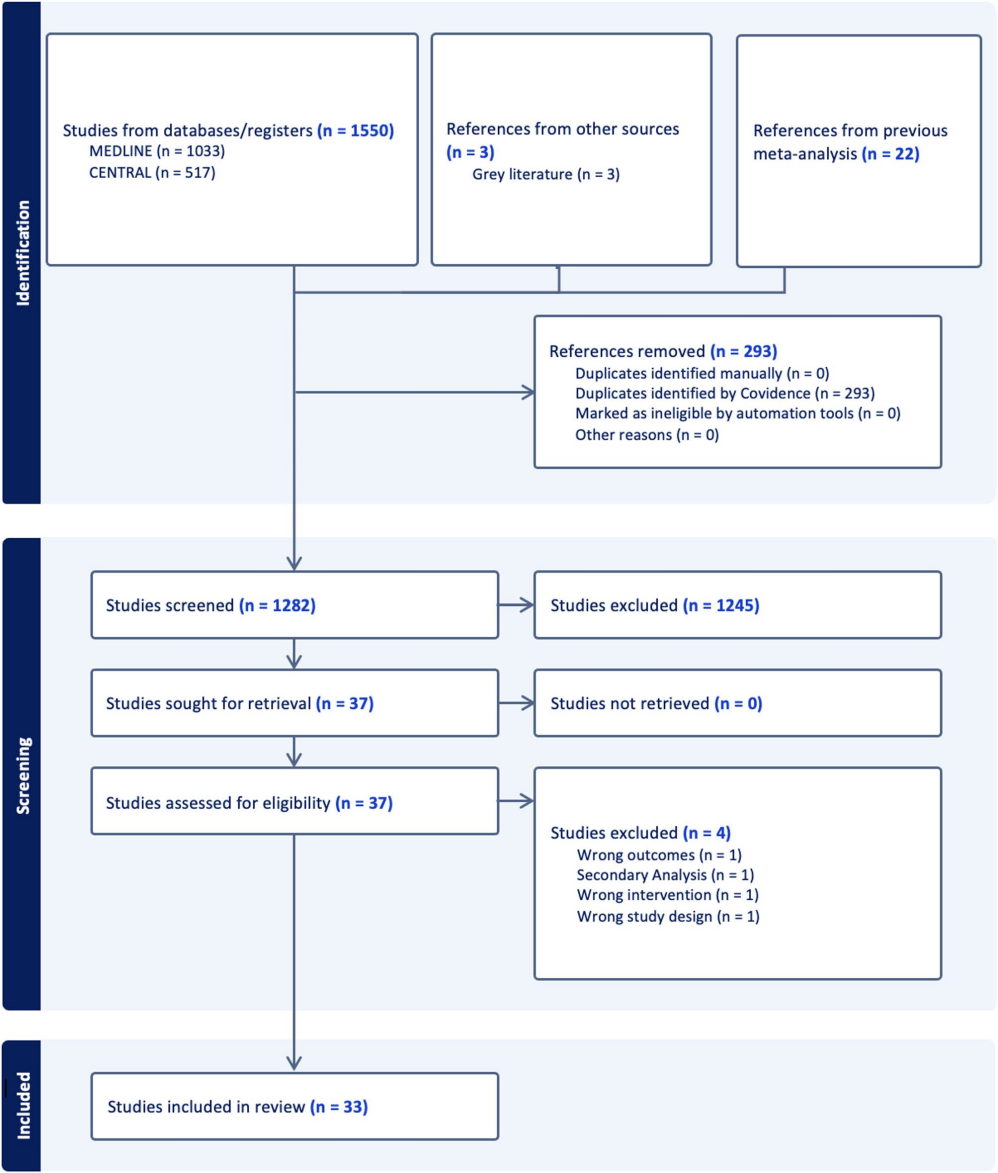
PRISMA flow diagram showing study selection process

A total of 1550 studies identified through searches of MEDLINE and CENTRAL were imported into Covidence for screening. An additional three studies were identified through a grey literature search. Additionally, we incorporated data from 22 trials included in the previous meta-analyses on which this review builds [9, 10], comprising 14 trials of SGLT2 inhibitors [19–32] and 8 trials of GLP-1RAs [33–40]. Following the removal of duplicates, 1282 unique records remained. After title and abstract screening, 1245 studies were excluded, and 37 proceeded to full-text review. Of these, 33 studies met the inclusion criteria and were selected for data extraction. The study selection process is shown in Fig. 1. Baseline information on the 11 newly included trials [7, 8, 41–49] can be found in Supplementary Table S2. Additional data were obtained from five post-hoc analyses of the newly included trials [50–55]. In total, this review included over 200,000 patients across 33 trials: 16 on SGLT2i, 14 on incretin-based therapies, and 3 on finerenone. A table displaying the full titles of all referenced trials, their corresponding acronyms, and subpopulations where trials or their corresponding post-hoc analyses were analyzed are available in Supplementary Appendix Table S1.

### Risk of bias and GRADE certainty

The risk of bias (RoB) assessments was completed for the 11 newly added studies using the Cochrane RoB 2.0 tool. Of these, 4 studies were assessed as having “some concerns” for overall risk of bias and 7 were rated as “low risk.” The main contributors to “some concerns” were related to reporting of the outcome and measurement of the outcome domains. The cumulative GRADE assessment across the full dataset largely reflects moderate certainty across the majority of outcomes. The updated RoB and GRADE assessments for the new trials are presented in Supplemental Tables S3 and S4. RoB assessments for the other 22 included trials are reported in the prior publications on which this analysis builds [9, 10].

### Cardiorenal outcome data

**Fig. 2:**
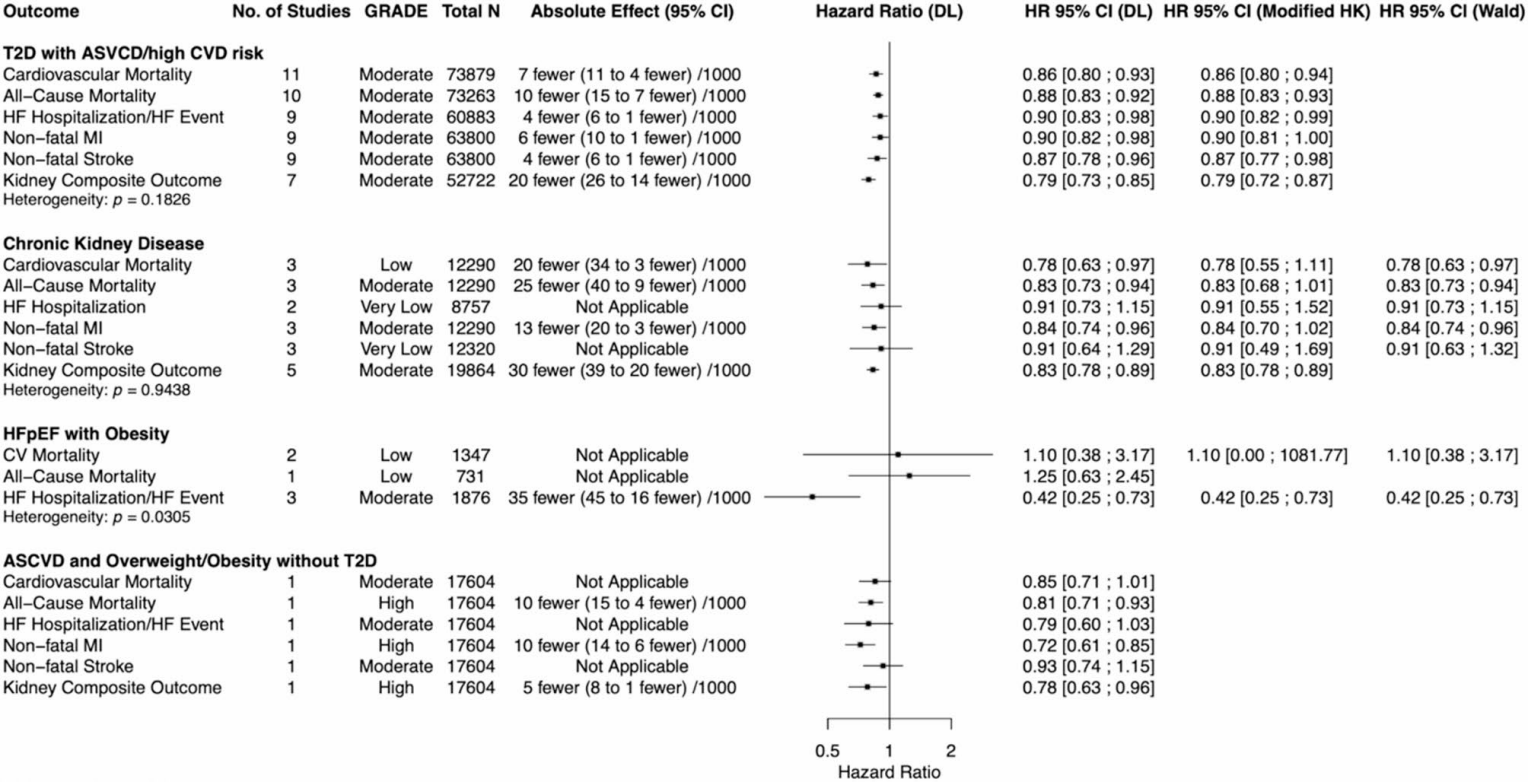
Summary of the effects of incretin-based therapies versus placebo on cardiorenal outcomes. DL = DerSimonian and Laird; modified HK = restricted maximum-likelihood (REML) estimation with the modified Hartung-Knapp adjustment; Wald = REML estimation with Wald-type confidence intervals. REML with Wald-type confidence intervals are presented for outcomes where there were three or fewer studies or between-study variance was 0%

**Fig. 3:**
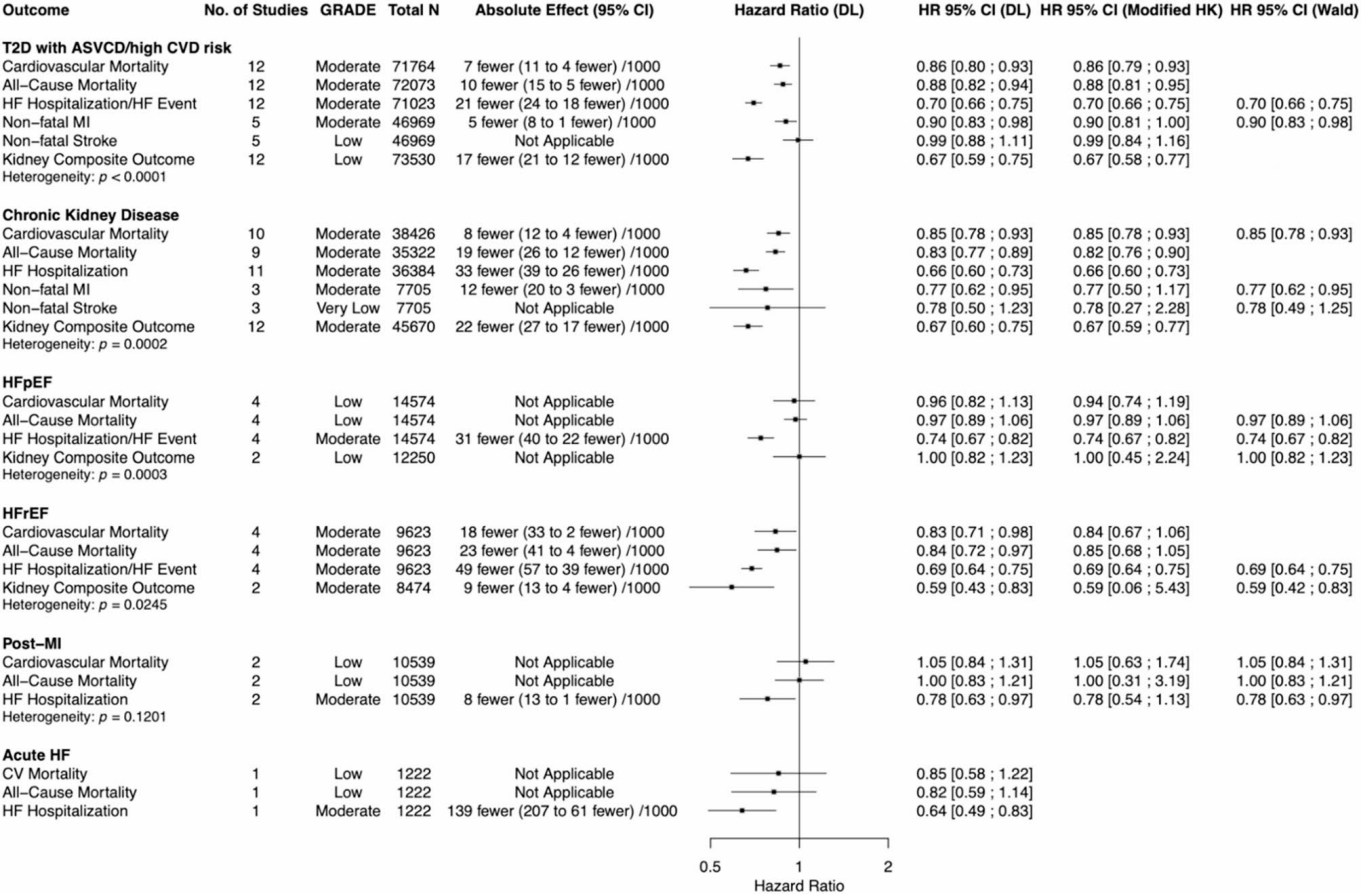
Summary of the effects of SGLT2 inhibitors versus placebo on cardiorenal outcomes. DL = DerSimonian and Laird; modified HK = restricted maximum-likelihood (REML) estimation with the modified Hartung-Knapp adjustment; Wald = REML estimation with Wald-type confidence intervals. REML with Wald-type confidence intervals are presented for outcomes where there were three or fewer studies or between-study variance was 0%

**Fig. 4:**
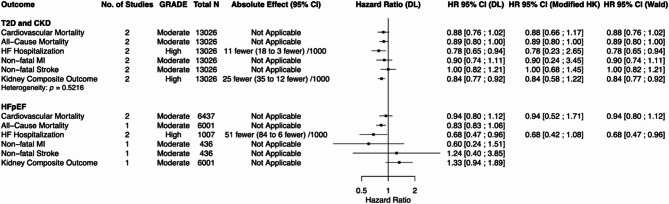
Summary of the effects of finerenone versus placebo on cardiorenal outcomes. DL = DerSimonian and Laird; modified HK = restricted maximum-likelihood (REML) estimation with the modified Hartung-Knapp adjustment; Wald = REML estimation with Wald-type confidence intervals. REML with Wald-type confidence intervals are presented for outcomes where there were three or fewer studies or between-study variance was 0%

Figures 2, 3, and 4 display HRs and their 95% CIs, along with GRADE certainty, total number of studies, and total population size for each outcome of interest (CV mortality, all-cause mortality, HF hospitalization or HF event, non-fatal MI, non-fatal stroke, kidney composite outcome) within each subpopulation for incretin-based therapies, SGLT2i, and finerenone respectively.

Data for the DL and REML-modified HK models are presented when more than two data points were available for a given outcome. For outcomes informed by ≤ 3 studies or when τ² = 0%, REML-Wald results were additionally reported in the main figures. Data for all statistical models for all outcomes can be found in the supplementary appendix (Supplemental Figures S1-S62).

Data for the overall population, which pools all available trial data, are presented in the supplementary appendix (Supplemental Figures S1–S10); however, these findings should be interpreted with caution given the heterogeneity in study populations. The overall population analysis included data from all eligible studies, with each trial represented once using data from the full study population. Subgroup-level data were not used to avoid double-counting participants.

Data for composite outcomes such as CV mortality or HF hospitalization (Supplemental Figures S4, S14, S24, S33), CV mortality or HF hospitalization/event (Supplemental Figures S3, S13, S23, S32, S42, S46, S60), and MACE (Supplemental Figures S9, S19, S28, S38, S50, S57, S62), can be found in the supplementary appendix. If data for HF hospitalization/event were available, they were presented in the main text; if separate data for HF hospitalization alone were available for a given population, they were included in the supplement. (Supplemental Figures S6, S16, S25, S35, S55, S56).

Absolute risk reductions per 1,000 patients, along with corresponding 95% CIs, are presented in Supplementary Table S4. Funnel plots assessing publication bias, with their corresponding Egger’s regression test can be found in the supplementary appendix (Supplemental Figures S89-S112).

### Incretin-based therapies

Figure 2 summarizes the efficacy of incretin-based therapies across various cardiorenal outcomes. Data is presented for the following subpopulations: (1) T2D with ASCVD/high CV risk, (2) CKD, (3) HFpEF and obesity, (4) ASCVD and overweight/obesity without T2D.

#### Incretin-based therapies: T2D with ASCVD/high CV risk

Among individuals with T2D with ASCVD/high CV risk, incretin-based therapies were associated with statistically significant reductions in CV mortality, all-cause mortality, HF hospitalization/event, non-fatal MI, non-fatal stroke, and kidney composite outcome under the DL model. Sensitivity analyses using the REML-modified HK model yielded comparable HRs, with all outcomes remaining statistically significant except for non-fatal MI, which no longer met significance.

#### Incretin-based therapies: chronic kidney disease

Among individuals with CKD, incretin-based therapies were associated with statistically significant reductions in CV mortality, all-cause mortality, non-fatal MI, and kidney composite outcome under the DL model. HF hospitalization and non-fatal stroke showed non-significant trends toward benefit. Sensitivity analyses using the REML-modified HK model yielded consistent HRs; however, CV mortality, all-cause mortality, and non-fatal MI no longer met statistical significance. These outcomes were informed by ≤ 3 studies, and all three outcomes remained statistically significant in the REML-Wald model.

#### Incretin-based therapies: HFpEF with obesity

Among individuals with HFpEF and obesity, incretin-based therapies were associated with a statistically significant reduction in time to first HF hospitalization/event using the DL model, which persisted in sensitivity analyses using both the REML-modified HK and REML-Wald models. No significant effects were observed for CV or all-cause mortality. Outcome data including a broader HFpEF subgroup analysis including subsets of the SELECT trial and the FLOW trial (which were restricted to patients with left ventricular ejection fraction [LVEF] ≥ 40% and LVEF ≥ 50% respectively), with SELECT enrolling participants with body mass index (BMI) ≥ 27 kg/m² and FLOW reporting a mean BMI of 32 kg/m² across the study population, demonstrated similar directional effects and statistical significance across cardiorenal outcomes (Supplemental Figures S30-S39).

#### Incretin-based therapies: ASCVD and overweight/obesity with no T2D

Among individuals with ASCVD and overweight/obesity without T2D, evidence was derived exclusively from the SELECT trial, which evaluated semaglutide in participants with BMI ≥ 27 kg/m². Incretin-based therapy was associated with statistically significant reductions in all-cause mortality, non-fatal MI, and kidney composite outcome. CV mortality, HF hospitalization/event, and non-fatal stroke showed favorable but non-significant downward trends.

### SGLT2 inhibitors

Figure 3 summarizes the efficacy of SGLT2i across various cardiorenal outcomes. Analyses were conducted on the following subpopulations: (1) T2D and ASCVD/high CV risk, (2) CKD, (3) HFpEF, (4) HFrEF, (5) post-MI, and (6) acute HF.

#### SGLT2i: T2D with ASCVD/high CV risk

Among individuals with T2D with ASCVD/high CV risk, SGLT2i were associated with statistically significant reductions in CV mortality, all-cause mortality, HF hospitalization/event, non-fatal MI, and kidney composite outcome under the DL model. No significant effect was observed for non-fatal stroke. In sensitivity analyses using the REML-modified HK model, all statistically significant findings remained significant except for non-fatal MI.

#### SGLT2i: chronic kidney disease

Among individuals with CKD, SGLT2i were associated with statistically significant reductions in CV mortality, all-cause mortality, HF hospitalization/event, non-fatal MI, and kidney composite outcome under the DL model. Non-fatal stroke showed a non-significant downward trend and did not reach statistical significance in either the REML-modified HK or REML-Wald models. In sensitivity analyses using the REML-modified HK model, non-fatal MI lost statistical significance; however, remained significant in the REML-Wald model.

#### SGLT2i: heart failure with preserved ejection fraction

Among individuals with HFpEF, SGLT2i were associated with a statistically significant reduction in time to first HF hospitalization/event across the DL, REML-modified HK, and REML-Wald models. No significant effects were observed for CV mortality, all-cause mortality, or kidney composite outcome.

#### SGLT2i: heart failure with reduced ejection fraction

Among individuals with HFrEF, SGLT2i were associated with statistically significant reductions in CV mortality, all-cause mortality, HF hospitalization/event, and kidney composite outcome under the DL model. Sensitivity analyses using the REML-modified HK model yielded comparable HRs; however, CV mortality, all-cause mortality, and kidney composite outcome no longer met statistical significance. The kidney composite outcome, informed by only two studies, remained statistically significant under the REML-Wald model. HF hospitalization/event remained significant across all models.

#### SGLT2i: post-myocardial infarction

Among individuals in the post-MI setting, SGLT2i were associated with a statistically significant reduction in time to first HF hospitalization/event under the DL and REML-Wald models, but not under the REML-modified HK model. CV mortality and all-cause mortality were not statistically significant across any model. In a sub-analysis restricted to post-MI patients without T2D, a similar significant reduction in HF hospitalization/event was observed across the DL and REML-Wald models (Supplemental Figure S56).

#### SGLT2i: acute heart failure

Among individuals with acute HF, SGLT2i were associated with a statistically significant reduction in HF hospitalization. CV mortality and all-cause mortality showed non-significant downward trends. In a pooled analysis of SOLOIST-WHF and EMPULSE, SGLT2 inhibitors significantly reduced the composite of total CV mortality or HF hospitalization/HF event across all three statistical models (Supplemental Figure S60).

### Finerenone

Finerenone was evaluated in two subpopulations: (1) individuals with T2D and CKD and (2) those with HFpEF. Figure 4 summarizes the effects of finerenone on cardiorenal outcomes across these groups.

#### Finerenone: diabetes and chronic kidney disease

Among individuals with diabetic CKD, finerenone was associated with statistically significant reductions in HF hospitalization and kidney composite outcome under the DL model. These effects were not statistically significant under the REML-modified HK model but remained significant in the REML-Wald model. CV mortality, all-cause mortality, and non-fatal MI showed non-significant downward trends, while no effect was observed for non-fatal stroke.

#### Finerenone: heart failure with preserved ejection fraction

Among individuals with HFpEF, finerenone was associated with a statistically significant reduction in HF hospitalization under the DL model. This effect was not statistically significant under the REML-modified HK model but remained significant in the REML-Wald model. No significant effects were observed for CV mortality across models. All-cause mortality, non-fatal MI, non-fatal stroke, and kidney composite outcome were each assessed in only one study and were not significant.

### Network meta-analysis

A network meta-analysis (NMA) was conducted across three populations with available data: (1) T2D with ASCVD/high CV risk, (2) CKD, and (3) HFpEF. For the T2D with ASCVD/high CV risk and CKD NMAs, all three drug classes were included in the analysis. Finerenone data were incorporated into both analyses but primarily reflect patients with diabetic kidney disease (DKD) and should be interpreted cautiously. In the HFpEF NMA, all three classes were compared; however, the incretin-based therapy data predominantly represent patients with HFpEF and overweight/obesity, which should be considered when interpreting the results.

In T2D with ASCVD/high CV risk, SGLT2i reduced HF hospitalization/event when compared to incretin-based therapies (HR 0.78, 95% CI 0.70–0.87), with no significant difference when compared to finerenone (HR 0.90, 95% CI 0.75–1.07) or between incretin-based therapies and finerenone (HR 1.15, 95% CI 0.95–1.38). In T2D with ASCVD/high CV risk, SGLT2i reduced the kidney composite outcome when compared to incretin-based therapies (HR 0.84, 95% CI 0.73–0.96) and finerenone (HR 0.78, 95% CI 0.65–0.94), with no significant difference between incretin-based therapies and finerenone (HR 0.93, 95% CI 0.78–1.11).

In CKD, SGLT2i reduced HF hospitalization when compared to incretin-based therapies (HR 0.72, 95% CI 0.58–0.90), with no significant difference when compared to finerenone (HR 0.84, 95% CI 0.67–1.07) or between incretin-based therapies and finerenone (HR 1.17, 95% CI 0.88–1.56). In CKD, SGLT2i reduced the kidney composite outcome when compared to incretin-based therapies (HR 0.82, 95% CI 0.73–0.91) and finerenone (HR 0.81, 95% CI 0.71–0.92), with no significant difference between incretin-based therapies and finerenone (HR 0.99, 95% CI 0.87–1.12). No significant differences were observed for CV mortality, all-cause mortality, non-fatal MI, or non-fatal stroke in the T2D with ASCVD/high CV risk and CKD subpopulations.

In the HFpEF NMA, there were no significant differences between any of the drug classes for CV mortality, all-cause mortality, or HF hospitalization/event. Similarly, for the kidney composite outcome, no significant difference was observed between SGLT2i and finerenone, and no data were available for incretin-based therapies.

Forest plots, network diagrams, as well data for other composite outcomes (e.g., CV mortality or HF hospitalization, MACE) are presented in the Supplementary Appendix (Supplemental Figure S63-S88).

## Discussions

**Fig. 5:**
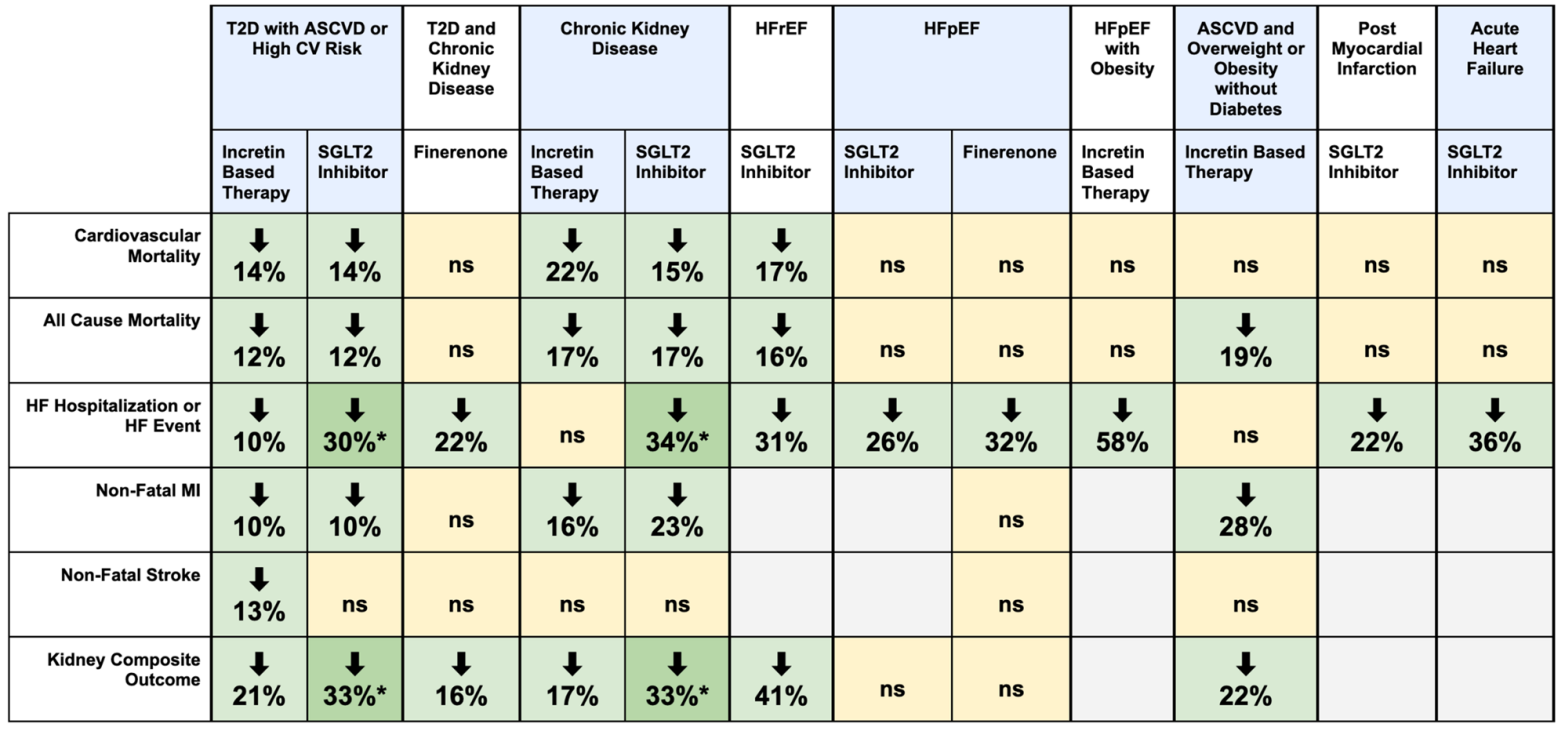
Green cells indicate statistically significant (*p* < 0.05) reductions in the specified outcome, while yellow cells indicate non-significant (ns) results. Blank cells denote outcomes not reported in the available studies. (*) denotes that the treatment class significantly outperformed the comparator drug class within the specified population, based on indirect comparisons from the network meta-analysis using placebo as the common comparator. Data reflected in this figure are derived from analyses conducted using the DerSimonian and Laird random-effects model. Abbreviations: ASCVD = atherosclerotic cardiovascular disease; CV = cardiovascular; HFpEF = heart failure with preserved ejection fraction; HFrEF = heart failure with reduced ejection fraction; MI = myocardial infarction; T2D = type 2 diabetes

This review presents an updated meta-analysis of trials reaffirming the cardiorenal risk-reducing benefits of SGLT2 inhibitors in patients with T2D, CKD, and HF, and of incretin-based therapies in those with T2D and CKD. Emerging data now extend the therapeutic relevance of incretin-based therapies to individuals with established ASCVD and overweight/obesity without diabetes, and HFpEF with obesity, while SGLT2 inhibitors show promise in post-MI and acute HF settings. This shift further challenges the historical paradigm of glucose-lowering agents being reserved for glycemic management alone and highlights the evolving role for these agents in the broader prevention of cardiorenal events. Other important developments since earlier analyses include the incorporation of finerenone and tirzepatide into the cardiorenal risk reduction narrative. These newer therapeutic tools are very relevant to the recently proposed, integrative concept of the Cardio-Kidney Metabolic Syndrome [56] and Systemic Metabolic Disorders [57]. To complement these findings, our NMA compared the relative efficacy of SGLT2i, incretin-based therapies, and finerenone within the broader T2D, CKD, and HFpEF populations. Figure 5 summarizes the main findings of the manuscript.

### Incretin-based therapies

This analysis examined the effects of incretin-based therapies on cardiorenal outcomes, including both GLP-1RA, and the dual GIP/GLP-1 receptor agonist tirzepatide. In the T2D population with ASCVD/high CV risk, several outcomes reached statistical significance, partly due to the inclusion of newer incretin-based therapy trials that shifted previously non-significant findings seen in earlier analyses [9, 11]. The addition of data from the FLOW and SOUL trials rendered the reduction in non-fatal MI statistically significant, a result not observed in earlier meta-analyses [9, 11]. In our analysis, there was a non-significant reduction in HF hospitalization among patients with T2D and ASCVD/high CV risk and became statistically significant with the inclusion of broader HF event data from the SOUL and STEP-HFpEF DM trials.

In the CKD population, beyond their renal benefits, incretin-based therapies also led to significant reductions in CV mortality, all-cause mortality, non-fatal MI, and MACE. This finding was in part driven by the addition of relatively newer data from the FLOW trial for CV mortality, all-cause mortality, and non-fatal MI, as well as the inclusion of the FLOW trial and a subset of the SELECT trial for MACE outcomes. This reinforces the growing evidence base supporting the cardioprotective effects of incretin-based therapies, especially in patients with diabetes and CKD. The 2024 Kidney Disease Improving Global Outcomes (KDIGO) guidelines recommend the use of GLP-1RAs in “adults with T2D and CKD who have not achieved individualized glycemic targets despite use of metformin and SGLT2 inhibitor treatment” [58]. The majority of GLP-1RA trials included in this meta-analysis explored outcomes in participants with CKD and concurrent T2D, with the exception of the SELECT trial. Notably, the SELECT trial demonstrated 31% reduction in MACE, suggesting a role for semaglutide in overweight or obese patients without T2D but with CKD. However, additional trials are warranted to evaluate long-term outcomes in non-diabetic patients with CKD of diverse etiologies before incretin-based therapies can be broadly recommended for the wider CKD population.

In the HFpEF population with obesity, incretin-based therapies were associated with a significant 40% reduction in time to first HF hospitalization/event. This finding was driven primarily by the SUMMIT trial, which contributed the greatest weight to the analysis (Supplemental Figure S43). In a post-hoc analysis of the SUMMIT trial [59], there was no statistical difference on the effect of tirzepatide on worsening HF event among patients with and without CKD, suggesting that the benefits of incretins in patients with HFpEF and obesity go beyond baseline CKD status. However, this finding should be interpreted in the context of the known limitations of estimating kidney function, as significant discrepancies between creatinine- and cystatin C–based eGFR can lead to misclassification of CKD status [60]. Ultimately, incretin-based therapies demonstrate promising benefits in patients with HFpEF with obesity, particularly in reducing the risk of HF hospitalization/event. This reinforces its therapeutic potential and warrants further investigation and consideration in future HFpEF management strategies in a subset of patients. However, additional evidence is needed to clarify their efficacy in non-diabetic populations, as most data to date are derived from cohorts with high rates of comorbid T2D.

Data on the effects of incretin-based therapies in individuals with ASCVD and overweight/obesity without T2D were limited to the SELECT trial. Among the outcomes of interest, semaglutide was associated with statistically significant reductions in all-cause mortality, CV mortality or HF hospitalization/event, non-fatal MI, MACE, and the composite kidney outcome [46].

Early results from the SURPASS-CVOT trial, which compared tirzepatide with dulaglutide in patients with T2D and ASCVD, demonstrated noninferiority for MACE, with preliminary evidence suggesting potential benefits in all-cause mortality, glycemic control, weight reduction, and kidney outcomes, with these findings pending full publication [61].

### SGLT2i

Across diverse patient populations, SGLT2i significantly reduced the risk of several outcomes. In subpopulations with T2D and ASCVD/high CV risk, as well as in those with CKD, SGLT2i lowered the risk of CV mortality, all-cause mortality, HF hospitalization/event, non-fatal MI, MACE, and the kidney composite outcome. Similar to previous analyses, no reduction in non-fatal stroke risk was observed in either the T2D or CKD subpopulations [10]. In the HFrEF subpopulation, significant reductions were seen in CV mortality, all-cause mortality, HF hospitalization/event, and the kidney composite outcome. While benefits were also observed in HFpEF populations, they were more modest, with a significant reduction primarily in time to first HF hospitalization/event.

In the post-MI population, pooled data from DAPA-MI and EMPACT-MI showed a significant reduction in time to first HF hospitalization, driven largely by EMPACT-MI, which contributed over 80% of the weight (Supplemental Figure S42). Notably, subgroup analysis from EMPACT-MI indicated that the benefit was confined to patients without baseline T2D, with no significant effect observed in those with diabetes [43]. The pooled analysis limited to DAPA-MI and the non-diabetic subgroup of EMPACT-MI remained significant, suggesting potential utility of SGLT2 inhibitors in non-diabetic post-MI patients.

Evidence supporting SGLT2i in the peri-acute HF setting was primarily derived from the SOLOIST-WHF trial, which demonstrated a significant reduction in total HF hospitalization events. The DAPA ACT HF-TIMI 68 trial was recently published after the date of our original search was therefore not included in this review. This trial found in-hospital initiation of dapagliflozin in patients hospitalized for acute HF had no statistically significant reduction in the risk of worsening HF events or CV death. However, a prespecified meta-analysis of SOLOIST-WHF, EMPULSE, and DAPA ACT HF-TIMI 68 demonstrated that in-hospital initiation of SGLT2i significantly reduced the risk of worsening HF or CV death by 29% and all-cause mortality by 43% [62].

### Finerenone

Finerenone has demonstrated an improved safety and tolerability profile compared to traditional steroidal MRAs in specific patient populations [63, 64]. Of the three RCTs on finerenone included in our analysis, FIGARO-DKD and FIDELIO-DKD focused on populations with T2D and CKD. In contrast, the FINEARTS-HF trial evaluated patients with HF with a LVEF ≥ 40%. This heterogeneity limits the interpretability of pooled effect estimates, as the underlying pathophysiology and therapeutic goals differ between these groups.

In participants with T2D and CKD, finerenone led to a statistically significant reduction in HF hospitalization/event and in the kidney composite outcome. In a pooled analysis of all three studies involving patients with HF and LVEF ≥ 40%, finerenone was associated with a significant reduction in the composite outcome of CV mortality or HF hospitalization. In the analysis of a subpopulation from FIGARO-DKD and FIDELIO-DKD with HF and LVEF ≥ 40%, finerenone led to a significant reduction in HF hospitalization/event. In patients with HFmrEF or HFpEF, finerenone appears to confer clinical benefits across the spectrum of kidney risk. In a prespecified analysis of FINEARTS-HF, finerenone consistently reduced CV mortality and total HF events, and improved HF-related health status (as measured by Kansas City Cardiomyopathy Questionnaire-Total Symptom Score [KCCQ-TSS]), regardless of baseline KDIGO risk category [65].

A meta-analysis of the three finerenone trials, titled FINE-HEART, was published in 2024 and employed individual participant-level data, allowing for harmonized endpoint definitions and more granular time-to-event analyses [66]. Interestingly, it demonstrated a statistically significant 20% reduction in the risk of the kidney composite outcome, largely driven by FIDELIO-DKD and FIGARO-DKD [66]. In contrast, while our trial-level meta-analysis showed a consistent direction of effect, the pooled kidney composite outcome data across the three trials did not reach statistical significance. This discrepancy likely reflects differences in methodology, as FINE-HEART was able to apply consistent outcome definitions across trials and conduct more statistically powerful analyses. Nevertheless, these findings highlight finerenone’s cardiorenal efficacy and reinforce its role in patients with overlapping cardiovascular, renal, and metabolic risk.

### Class-level comparison of therapeutic efficacy

Our NMA provides a comparative overview of class-level efficacy across T2D with ASCVD/high CV risk, CKD, and HFpEF populations. SGLT2i demonstrated superior efficacy in reducing both HF hospitalization and kidney composite outcome compared to incretin-based therapies in T2D with ASCVD/high CV risk and CKD subgroups. SGLT2i also outperformed finerenone for these outcomes; however, this comparison should be interpreted with caution, as most finerenone data were derived from a broader diabetic kidney disease population. In contrast, no significant differences were observed between drug classes in the HFpEF population across CV mortality, all-cause mortality, or HF outcomes. The NMA findings should be interpreted with caution and viewed as hypothesis generating rather than definitive. While indirect NMA comparisons reached statistical significance for some outcomes, class-level estimates against placebo show overlapping confidence intervals, and head-to-head trial evidence is lacking. Nonetheless, these findings highlight the differential cardiorenal benefits of each medication class depending on the underlying comorbidity, reinforcing the need for phenotype-specific therapeutic strategies, and the importance of determining the optimal sequencing of therapy in patients eligible for dual or triple therapy.

### Limitations

Some limitations should be acknowledged when interpreting the findings of this review. Heterogeneity in study design, patient populations, and endpoint definitions must be considered when evaluating pooled results. Specifically, definitions of HF based on ejection fraction, laboratory cutoffs for CKD, and components of the kidney composite outcome varied across trials, which may impact the consistency and validity of subgroup analyses. In the case of the finerenone trials, the FINEARTS-HF trial differed significantly in its inclusion criteria compared to FIDELIO-DKD and FIGARO-DKD, enrolling a HFpEF population rather than patients with T2D and CKD. This clinical heterogeneity complicates pooled analyses, particularly for kidney outcomes, where the contribution of FINEARTS-HF may not be directly comparable to the other finerenone trials. Combining HF hospitalization data with broader HF event data introduces additional heterogeneity. While this approach broadens event capture, it may reduce the specificity of the outcome. A dedicated analysis focused solely on HF events could help clarify treatment effects more precisely. Additionally, studies were limited for certain subpopulations, particularly those post-MI, acute HF, and ASCVD and overweight/obesity without T2D. Similarly, HFpEF with obesity subpopulation was largely derived from STEP-HFpEF and SELECT, while the finerenone evidence came from relatively few studies. Subgroup findings based on limited number of trials should therefore be interpreted with caution. Subpopulation findings based on a limited number of trials should therefore be interpreted with caution, and in these cases, results can be considered exploratory rather than definitive. Next, this study was not prospectively registered, which limits transparency regarding pre-specified objectives and any post hoc modifications to the analytical approach. Additionally, meta-regression analyses could not be performed due to an insufficient number of trials for the majority of outcomes per subgroup, as such analyses are generally underpowered when conducted with fewer than ten studies [17]. Although a NMA was performed, certain subgroup comparisons are limited by heterogeneity in population definitions. Additionally, NMAs rely on assumptions of transitivity and consistency, which may not be fully satisfied given heterogeneity across trials. GRADE ratings were derived from DL estimates for consistency with prior meta-analyses, though this approach has recognized limitations and may overestimate certainty of evidence. Lastly, Finerenone data largely reflect the DKD population but were applied to both T2D and CKD subgroups, while incretin-based therapy data in HFpEF predominantly represent overweight or obesity with HFpEF populations. Lastly, publication bias was assessed using Egger’s test and funnel plots for outcomes with ten or more studies; however, several subgroup and outcome analyses included fewer trials, precluding formal assessment, and thus residual publication bias cannot be excluded.

### Future directions

Future research should focus on underrepresented populations, including various non-diabetic populations, to better define the role of these therapies beyond traditional indications. Data suggest complementary benefits from dual combination therapy [67–72]; however, randomized trial evidence on hard clinical endpoints, whether for dual or triple therapy, remains limited, highlighting an important area for future investigation. As the evidence base continues to expand, updated clinical guidelines are warranted to reflect the broader applicability of these agents in cardiorenal risk reduction.

Other recent trials have been published, and while not included in our analysis due different patient populations, may contribute to future standards of care. The STRIDE trial evaluated the effects of once-weekly semaglutide on walking capacity, and quality of life in patients with T2D and symptomatic peripheral artery disease (PAD) [73]. Semaglutide significantly improved maximum walking distance, pain-free walking distance, and vascular quality-of-life scores over 52 weeks, and reduced need for rescue interventions [73].​ While event rates were low, semaglutide was associated with a lower incidence of all-cause mortality (1% vs. 2%); however, this difference was not statistically significant [73]. To our knowledge this is the first large RCT to evaluate the effects of a GLP-1RA on functional outcomes in patients with symptomatic PAD, addressing a major therapeutic gap in this high-risk population. The DAPA-TAVI trial evaluated the effects of dapagliflozin patients with HF and aortic stenosis undergoing transcatheter aortic valve implantation (TAVI) [74]. Dapagliflozin significantly reduced the 1-year incidence of the composite of all-cause mortality or worsening HF compared to standard care, driven primarily by fewer HF events [74]. Although subjects in this trial met existing indications for use of SGLT2i, this trial provides important evidence for their use in the elderly post-TAVI population that has been underrepresented in prior cardiovascular outcome trials.

##  Conclusion 

This updated analysis supports the benefits of incretin-based therapies, SGLT2 inhibitors, and finerenone in reducing cardiorenal events across diverse populations, particularly those with T2D, ASCVD, CKD, and HF. Emerging data suggest potential benefits in additional high-risk groups including acute HF and post-MI for SGLT2i, and HFpEF with obesity for incretin-based therapies, as well as non-diabetic populations, particularly individuals with ASCVD and overweight/obesity. Our network-metanalysis suggests that the optimal therapeutic choice may vary depending on underlying patient comorbidities, emphasizing the need for phenotype-specific therapeutic strategies. These findings support the expanding role of these therapies in cardiorenal risk reduction. As the evidence base grows, future updates to clinical guidelines should reflect these findings and account for the emerging benefits of these therapies in non-diabetic and other high-risk populations.

## Supplementary Information

Below is the link to the electronic supplementary material.

Supplementary Material 1

## Data Availability

No datasets were generated or analysed during the current study.

## Declarations

### Generative AI and AI-assisted technologies in the writing process

During the preparation of this work the authors used ChatGPT to proofread and improve readability of the text. After using this tool, the authors reviewed and edited the content as needed and take full responsibility for the content of the publication.
