# Supplementary material for: Effectiveness of SGLT2 inhibitors, incretin-based therapies, and finerenone on cardiorenal outcomes: a meta-analysis and network meta-analysis
Source: Cardiovasc Diabetol Endocrinol Rep. 2025 Dec 8;11:37. doi: 10.1186/s40842-025-00248-2 (PMC12683810; doi:10.1186/s40842-025-00248-2)
Supplement: Supplementary file 1 — Supplementary Material 1 [file 40842_2025_248_MOESM1_ESM.docx]

**Supplementary Appendix**

**Supplementary Appendix Table S1**: Full Trial Titles & Corresponding Acronyms - **Page 2 - 3**

**Supplementary Appendix Table S2**: Trial Baseline Information - **Page 4 - 5**

**Supplementary Appendix Table S3**: Cochrane Risk of Bias 2.0 for Included Studies - **Page 5**

**Supplementary Appendix Table S4**: GRADE Evidence Rating and Relative and Absolute Risk Reduction - **Page 6 - 21**

**Search Strategy - Page 21 - 23**

**Figures S1-S53**: Forest Plots

- **Figures S1-S10:** Overall Population - **Page 24 - 53**
- **Figures S11-S20:** T2D with ASCVD/High CVD Risk - **Page 54 - 83**
- **Figures S21-S29:** Chronic Kidney Disease - **Page 84 - 98**
- **Figures S30-S39:** Heart Failure - **Page 99 - 117**
- **Figures S40-S43:** HFpEF with Obesity - **Page 117 - 119**
- **Figures S44-S51:** ASCVD and Overweight/Obesity without T2D - **Page 120 - 121**
- **Figures S52-S57-:** Post-MI - **Page 122 - 126**
- **Figures S58-S62:** Acute HF - **Page 127 – 128**

**Figures S63-S88**: Network Meta Analysis - **Page 129 - 154

Figures S89-S112**: Funnel Plots - **Page 155 - 178**

**Supplemental Table S1. Full Trial Titles, Corresponding Acronyms, and Subpopulations Where Trials Were Analyzed**

| **Acronym** | **Full Title** | **Subpopulations Where Given Study or Its Post-hoc Analysis Was Included** |
| --- | --- | --- |
| Incretin-Based Therapies | | |
| FLOW | Effects of Semaglutide on Chronic Kidney Disease in Patients with Type 2 Diabetes | T2D, CKD, HF |
| SELECT | Semaglutide and Cardiovascular Outcomes in Obesity without Diabetes | HF, ASCVD and overweight/obese without T2D |
| SOUL | Oral Semaglutide and Cardiovascular Outcomes in High-Risk Type 2 Diabetes | T2D |
| STEP-HFpEF | Semaglutide in Patients with Heart Failure with Preserved Ejection Fraction and Obesity | HF, HFpEF with Obesity |
| STEP-HFpEF DM | Semaglutide in Patients with Obesity-Related Heart Failure and Type 2 Diabetes | T2D, HF, HFpEF with Obesity |
| SUMMIT | Tirzepatide for Heart Failure with Preserved Ejection Fraction and Obesity | HF, HFpEF with Obesity |
| Harmony Outcomes | Albiglutide and cardiovascular outcomes in patients with type 2 diabetes and cardiovascular disease (Harmony Outcomes): a double-blind, randomised placebo-controlled trial | T2D |
| REWIND | Dulaglutide and cardiovascular outcomes in type 2 diabetes (REWIND): a double-blind, randomised placebo-controlled trial | T2D |
| AMPLITUDE-O | Cardiovascular and Renal Outcomes with Efpeglenatide in Type 2 Diabetes | T2D |
| EXSCEL | Effects of Once-Weekly Exenatide on Cardiovascular Outcomes in Type 2 Diabetes | T2D, CKD |
| LEADER | Liraglutide and Cardiovascular Outcomes in Type 2 Diabetes | T2D, CKD |
| ELIXA | Lixisenatide in Patients with Type 2 Diabetes and Acute Coronary Syndrome | T2D |
| PIONEER 6 | Oral Semaglutide and Cardiovascular Outcomes in Patients with Type 2 Diabetes | T2D |
| SUSTAIN-6 | Semaglutide and Cardiovascular Outcomes in Patients with Type 2 Diabetes | T2D |
| SGLT2i | |  |
| DAPA-MI | Dapagliflozin in Myocardial Infarction without Diabetes or Heart Failure | Post-MI |
| EMPACT-MI | Empagliflozin after Acute Myocardial Infarction | T2D, Post-MI |
| CANVAS | Canagliflozin and Cardiovascular and Renal Events in Type 2 Diabetes | T2D, CKD |
| CREDENCE | Canagliflozin and Renal Outcomes in Type 2 Diabetes and Nephropathy | T2D, CKD |
| DECLARE-TIMI 58 | Dapagliflozin and Cardiovascular Outcomes in Type 2 Diabetes | T2D, CKD, HF |
| DELIVER | Dapagliflozin in Heart Failure with Mildly Reduced or Preserved Ejection Fraction | T2D, CKD, HF |
| DAPA-CKD | Dapagliflozin in Patients with Chronic Kidney Disease | T2D, CKD |
| DAPA-HF | Dapagliflozin in Patients with Heart Failure and Reduced Ejection Fraction | T2D, CKD, HF |
| EMPA-KIDNEY | Empagliflozin in Patients with Chronic Kidney Disease | T2D, CKD |
| EMPA-REG OUTCOME | Empagliflozin, Cardiovascular Outcomes, and Mortality in Type 2 Diabetes | T2D, CKD |
| EMPEROR-PRESERVED | Empagliflozin in Heart Failure with a Preserved Ejection Fraction | T2D, CKD, HF |
| EMPEROR-REDUCED | Cardiovascular and Renal Outcomes with Empagliflozin in Heart Failure | T2D, CKD, HF |
| EMPULSE | The SGLT2 inhibitor empagliflozin in patients hospitalized for acute heart failure: a multinational randomized trial | Acute HF |
| VERTIS CV | Cardiovascular Outcomes with Ertugliflozin in Type 2 Diabetes | T2D, CKD, HF |
| SCORED | Sotagliflozin in Patients with Diabetes and Chronic Kidney Disease | T2D, CKD |
| SOLOIST-WHF | Sotagliflozin in Patients with Diabetes and Recent Worsening Heart Failure | T2D, Acute HF |
| Finerenone | |  |
| FIDELIO-DKD | Effect of Finerenone on Chronic Kidney Disease Outcomes in Type 2 Diabetes | T2D, CKD |
| FIGARO-DKD | Cardiovascular Events with Finerenone in Kidney Disease and Type 2 Diabetes | T2D, CKD |
| FINEARTS-HF | Finerenone in Heart Failure with Mildly Reduced or Preserved Ejection Fraction | HF |

Supplemental Table S1. Full trial titles and corresponding acronyms for major clinical studies included in this review.

**Supplemental Table S2: Trial Baseline Information**

| **Trial Name** | **Author, Year** | **Intervention** | **Comorbidities** | **Total sample size** | **Intervention sample size** | **Control sample size** | **% Male** | **Mean age** | **Follow-up time/study duration** |
| --- | --- | --- | --- | --- | --- | --- | --- | --- | --- |
| EMPACT-MI | Butler et al, 2024 | Empagliflozin 10 mg daily vs placebo | Patients hospitalized for acute MI and at risk for HF | 6522 | 3260 | 3262 | 75.1% Intervention  75.1% Control | 63.6±11.0 Intervention  63.7±10.8 Control | 17.9 months (median) |
| DAPA-MI | James et al., 2023 | Dapagliflozin 10mg daily vs placebo | Patients without diabetes or chronic HF, presenting with acute MI  and impaired left ventricular systolic function | 4017 | 2019 | 1998 | 80.8% Intervention  79.0% Control | 63.0±11.06 Intervention  62.8±10.64 Control | 11.6 months (median) |
| SUMMIT | Packer et al., 2024 | Tirzepatide (up to 15 mg once weekly) vs placebo | Patients with HFpEF and obesity (BMI ≥30) | 731 | 364 | 267 | 45.1% Intervention  47.4% Control | 65.5±10.5 Intervention  65.0±10.9 Control | 104 weeks (median) |
| FLOW | Perkovic et al., 2024 | Semaglutide (1.0 mg weekly) vs placebo | T2D and CKD | 3533 | 1767 | 1766 | 70.6% Intervention 68.9%  Control, 69.7% Total | 66.6±9.0 Intervention  66.7±9.0 Control  66.6±9.0 Total | 3.4 years (median) |
| SELECT | Lincoff et al., 2023 | Semaglutide (2.4 mg weekly) vs placebo | Preexisting CVD and a BMI ≥ 27 and no diabetes | 17,604 | 8803 | 8801 | 72.2% Intervention  72.5% Control | 61.6±8.9 Intervention  61.6±8.8 Control | 39.8±9.4 months (mean) |
| SOUL | McGuire et al., 2025 | Oral semaglutide (14 mg once daily) vs placebo | T2D with ASCVD or CKD (or both) | 9,650 | 4,825 | 4,825 | 71.5% Intervention  70.7% control | 66.1 ± 7.6 Intervention  66.1 ± 7.5 Control | 47.5 months (mean) |
| STEP-HFpEF | Kosiborod et al., 2023 | Semaglutide (2.4 mg weekly) vs placebo | HFpEF, obesity (BMI ≥30) | 529 | 263 | 266 | 43.3% Intervention  44.4% Control,  43.9% Total | 70 (62–75)  Intervention  69 (62–75) Control  69 (62–75) Total | 52 weeks treatment,  followed by a 5-week follow-up period |
| STEP-HFpEF DM | Kosiborod et al., 2024 | Semaglutide (2.4 mg weekly) vs placebo | HFpEF, obesity (BMI ≥30), and T2D | 616 | 310 | 306 | 58.7% Intervention  52.6% Control | 69.0 (62.0–74.0) Intervention  70.0 (63.0–75.0) Control | 52 weeks treatment,  followed by a 5-week follow-up period |
| FINEARTS-HF | Solomon et al, 2024 | Finerenone (20 or 40mg once daily) vs placebo | symptomatic HF and a LVEF≥40% | 6001 | 3003 | 2998 | 45.1% Intervention  45.9% Control | 71.9±9.6 Intervention  72.0±9.7 Control | 32 months (median) |
| FIGARO-DKD | Pitt et al, 2021 | Finerenone (20 mg once daily) vs placebo | Patients with CKD and T2D | 7352 | 3686 | 3666 | 2528 (68.6%) Intervention  2577 (70.3%) Control  5105 (69.4%) Total | 64.1±9.7 Intervention,  64.1±10.0 Control  64.1±9.8 Total | 3.4 years (median) |
| FIDELIO-DKD | Bakris et al., 2020 | Finerenone (20 mg once daily) vs placebo | patients with CKD and T2D | 5734 | 2833 | 2841 | 1953 (68.9%) Intervention,  2030 (71.5%) Control,  3983 (70.2%) Total | 65.4±8.9 Intervention,  65.7±9.2 Control,  65.6±9.1 Total | 2.6 years (median) |

Supplemental Table S2. Baseline characteristics and study design details of clinical trials, including trial name, intervention, comorbidities, sample size, demographics, and follow-up duration.

**Supplemental Table S3: Cochrane Risk of Bias 2.0 for Included Studies**

| Study, Year | Randomization Process | Intervention Deviation | Missing Outcome Data | Measurement of the Outcome | Reporting of the Outcome | Overall Risk |
| --- | --- | --- | --- | --- | --- | --- |
| DAPA-MI, 2023 | Low Risk | Low Risk | Low Risk | Low Risk | Some Concerns | Some Concerns |
| EMPACT-MI, 2024 | Low Risk | Low Risk | Low Risk | Low Risk | Low Risk | Low Risk |
| FLOW, 2024 | Low Risk | Low Risk | Low Risk | Low Risk | Low Risk | Low Risk |
| SELECT, 2023 | Low Risk | Low Risk | Low Risk | Low Risk | Low Risk | Low Risk |
| SOUL, 2025 | Low Risk | Low Risk | Low Risk | Low Risk | Low Risk | Low Risk |
| STEP-HFpEF, 2023 | Low Risk | Low Risk | Low Risk | Some Concerns | Low Risk | Some Concerns |
| STEP-HFpEF DM, 2024 | Low Risk | Low Risk | Low Risk | Some Concerns | Low Risk | Some Concerns |
| SUMMIT, 2024 | Low Risk | Low Risk | Low Risk | Some Concerns | Some Concerns | Some Concerns |
| FIDELIO-DKD, 2020 | Low Risk | Low Risk | Low Risk | Low Risk | Low Risk | Low Risk |
| FIGARO-DKD, 2021 | Low Risk | Low Risk | Low Risk | Low Risk | Low Risk | Low Risk |
| FINEARTS-HF, 2024 | Low Risk | Low Risk | Low Risk | Low Risk | Low Risk | Low Risk |

Supplemental Table S3. Cochrane Risk of Bias 2.0 assessment for included studies, summarizing risk across domains of randomization, intervention deviations, missing data, outcome measurement, and reporting.

**Supplemental Table S4: GRADE Evidence Rating and Relative and Absolute Risk Reduction**

| **Incretin-Based Therapies** | | | | | | | | | | |
| --- | --- | --- | --- | --- | --- | --- | --- | --- | --- | --- |
| Certainty assessment | | | | | | # of events / # of  patients; Event rate | | Effect | | Certainty |
| No of studies | Risk of Bias | Inconsistency | Indirectness | Imprecision | Other Consideration | Incretin | Control | Relative (95% CI) | Absolute  (95%  CI) |  |
| **Overall Population** | | | | | | | | | | |
| CV Mortality | | | | | | | | | | |
| 13 | Serious | Not Serious | Not Serious | Not Serious | None^a^ | 1945/  46763 (4.2%) | 2181/  45451 (4.8%) | HR 0.86 (0.81 - 0.92) | 7 fewer per 1000 (from 9 fewer to 4 fewer) | ⨁⨁⨁◯  Moderate |
| All-Cause Mortality | | | | | | | | | | |
| 12 | Serious | Not Serious | Not Serious | Not Serious | None^a^ | 3176/  46453 (6.8%) | 3554/  45145 (7.9%) | HR 0.87 (0.83 - 0.91) | 10 fewer per 1000 (from 13 fewer to 7 fewer) | ⨁⨁⨁◯  Moderate |
| HF Hospitalization/Event | | | | | | | | | | |
| 12 | Serious | Not Serious | Not Serious | Not Serious | Serious^c^ | 1155/  40528 (2.8%) | 1286/  39219 (3.3%) | HR 0.87 (0.79 - 0.96) | 4 fewer per 1000 (from 7 fewer to 1 fewer) | ⨁⨁◯◯  Low |
| Non-Fatal MI | | | | | | | | | | |
| 10 | Serious | Not Serious | Not Serious | Not Serious | None^a^ | 1871/  41358 (4.5%) | 2057/  40046 (5.1%) | HR 0.87 (0.79 - 0.96) | 7 fewer per 1000 (from 11 fewer to 2 fewer) | ⨁⨁⨁◯  Moderate |
| Non-Fatal Stroke | | | | | | | | | | |
| 10 | Serious | Not Serious | Not Serious | Not Serious | None^a^ | 971/  41358 (2.3%) | 1067/ 40046 (2.7%) | HR 0.88 (0.80 - 0.96) | 3 fewer per 1000 (from 5 fewer to 1 fewer) | ⨁⨁⨁◯  Moderate |
| Kidney Composite Outcome | | | | | | | | | | |
| 8 | Serious | Not Serious | Not Serious | Not Serious | None^b^ | 2375/  35836 (6.6%) | 2667/  34490 (7.7%) | HR 0.79 (0.74 - 0.84) | 16 fewer per 1000  (from 20 fewer to 12 fewer) | ⨁⨁⨁◯  Moderate |
| CV Mortality or HF Hospitalization/HF Event | | | | | | | | | | |
| 8 | Serious | Not Serious | Not Serious | Not Serious | None^b^ | 1516/  28243 (5.4%) | 1741/  28245 (6.2%) | HR 0.86 (0.80 - 0.93) | 9 fewer per 1000  (from 12 fewer to 4 fewer) | ⨁⨁⨁◯  Moderate |
| MACE | | | | | | | | | | |
| 11 | Serious | Not Serious | Not Serious | Not Serious | None^a^ | 4503/  46089 (9.8%) | 5059/  44778 (11.3%) | HR 0.85 (0.81 - 0.90) | 17 fewer per 1000 (from 21 fewer to 11 fewer) | ⨁⨁⨁◯  Moderate |
| **T2D with ASCVD/High CV RIsk** | | | | | | | | | | |
| CV Mortality | | | | | | | | | | |
| 11 | Serious | Not Serious | Not Serious | Not Serious | None^a^ | 1714/  37596 (4.6%) | 1914/  36283 (5.3%) | HR 0.86 (0.80 - 0.93) | 7 fewer/  1000  (from 11 fewer to 4 fewer) | ⨁⨁⨁◯  Moderate |
| All-Cause Mortality | | | | | | | | | | |
| 10 | Serious | Not Serious | Not Serious | Not Serious | None^a^ | 2782/  37286 (7.5%) | 3081/  35977 (8.6%) | HR 0.88 (0.83 - 0.92) | 10 fewer per 1000  (from 15 fewer to 7 fewer) | ⨁⨁⨁◯  Moderate |
| HF Hospitalization/Event | | | | | | | | | | |
| 9 | Serious | Not Serious | Not Serious | Not Serious | None^b^ | 1045/  31098 (3.4%) | 1126/  29785 (3.8%) | HR 0.9 (0.83 - 0.98) | 4 fewer per 1000  (from 6 fewer to 1 fewer) | ⨁⨁⨁◯  Moderate |
| Non-Fatal MI | | | | | | | | | | |
| 9 | Serious | Not Serious | Not Serious | Not Serious | None^b^ | 1637/  32555 (5.0%) | 1735/  31245 (5.6%) | HR 0.9 (0.82 - 0.98) | 6 fewer per 1000 (from 10 fewer to 1 fewer) | ⨁⨁⨁◯  Moderate |
| Non-Fatal Stroke | | | | | | | | | | |
| 9 | Serious | Not Serious | Not Serious | Not Serious | None^b^ | 817/  32555 (2.5%) | 902/ 31245 (2.9%) | HR 0.87 (0.78 - 0.96) | 4 fewer per 1000 (from 6 fewer to 1 fewer) | ⨁⨁⨁◯  Moderate |
| Kidney Composite Outcome | | | | | | | | | | |
| 7 | Serious | Not Serious | Not Serious | Not Serious | None^b^ | 2220/  27033 (8.2%) | 2469/  25689 (9.6%) | HR 0.79 (0.73 - 0.85) | 20 fewer per 1000 (from 26 fewer to 14 fewer) | ⨁⨁⨁◯  Moderate |
| CV Mortality or HF Hospitalization/HF Event | | | | | | | | | | |
| 5 | Serious | Not Serious | Not Serious | Not Serious | None^b^ | 1159/  18813 (6.2%) | 1289/  18851 (6.8%) | HR 0.89 (0.82 - 0.96) | 8 fewer per 1000 (from 12 fewer to 3 fewer) | ⨁⨁⨁◯  Moderate |
| MACE | | | | | | | | | | |
| 10 | Serious | Not Serious | Not Serious | Not Serious | None^a^ | 3934/  37286 (10.6%) | 4358/  35977 (12.1%) | HR 0.86 (0.82 - 0.91) | 17 fewer per 1000  (from 22 fewer to 11 fewer) | ⨁⨁⨁◯  Moderate |
| **Chronic Kidney Disease** | | | | | | | | | | |
| CV Mortality | | | | | | | | | | |
| 3 | Serious | Serious | Not Serious | Not Serious | None^b^ | 447/ 6124 (7.3%) | 560/  6166 (9.1%) | HR 0.78 (0.63 - 0.97) | 20 fewer per 1000 (from 34 fewer to 3 fewer) | ⨁⨁◯◯  Low |
| All-Cause Mortality | | | | | | | | | | |
| 3 | Serious | Not Serious | Not Serious | Not Serious | None^b^ | 758/ 6124 (12.4%) | 902/  6166 (14.6%) | HR 0.83 (0.73 - 0.94) | 25 fewer per 1000 (from 40 fewer to 9 fewer) | ⨁⨁⨁◯  Moderate |
| HF Hospitalization | | | | | | | | | | |
| 2 | Serious | Serious | Not Serious | Serious | None^b^ | 299/  4357  (6.9%) | 322/  4400  (7.3%) | HR 0.91  (0.73 -  1.15) | 7 fewer per 1000 (from 20 fewer to 11 more) | ⨁◯◯◯  Very Low |
| Non-Fatal MI | | | | | | | | | | |
| 3 | Serious | Not Serious | Not Serious | Not Serious | None^b^ | 411/  6124 (6.7%) | 483/  6166 (7.8%) | HR 0.84 (0.74 - 0.96) | 13 fewer per 1000  (from 20 fewer to 3 fewer) | ⨁⨁⨁◯  Moderate |
| Non-Fatal Stroke | | | | | | | | | | |
| 3 | Serious | Serious | Not Serious | Serious | None^b^ | 228/  6124 (3.7%) | 242/  6166 (3.9%) | HR 0.91 (0.64 - 1.29) | 4 fewer per 1000 (from 14 fewer to 11 more) | ⨁◯◯◯  Very Low |
| Kidney Composite Outcome | | | | | | | | | | |
| 5 | Serious | Not Serious | Not Serious | Not Serious | None^b^ | 1532/  9882 (15.5%) | 1783/  9982 (19.9%) | HR 0.83 (0.78 - 0.89) | 30 fewer per 1000  (from 39 fewer to 20 fewer) | ⨁⨁⨁◯  Moderate |
| CV Mortality or HF Hospitalization/HF Event | | | | | | | | | | |
| 1 | Serious | Not Applicable | Not Serious | Serious | None^a^ | 22/  167 (13.1%) | 34/  168 (20.2%) | HR 0.61 (0.36 - 1.05) | 79 fewer per 1000 (from 130 fewer to 10 more) | ⨁⨁◯◯  Low |
| MACE | | | | | | | | | | |
| 8 | Serious | Not Serious | Not Serious | Not Serious | None^b^ | 1420/  10493 (13.5%) | 1674/  10528 (15.9%) | HR 0.83 (0.76 - 0.90) | 27 fewer per 1000 (from 38 fewer to 16 fewer) | ⨁⨁⨁◯  Moderate |
| **HFpEF with Obesity** | | | | | | | | | | |
| CV Mortality | | | | | | | | | | |
| 2 | Serious | Not Serious | Not Serious | Serious | None^b^ | 10/  674 (1.5%) | 9/  673 (1.3%) | HR 1.10 (0.38 - 3.17) | 1 more per 1000 (from 8 fewer to 29 more) | ⨁⨁◯◯  Low |
| All-Cause Mortality | | | | | | | | | | |
| 1 | Serious | Not Applicable | Not Serious | Serious | None^b^ | 19/364 (5.2%) | 15/367 (4.1%) | HR 1.25 (0.63 - 2.45) | 10 more per 1000 (from 15 fewer to 59 more) | ⨁⨁◯◯  Low |
| HF Hospitalization/Event | | | | | | | | | | |
| 3 | Serious | Not Serious | Not Serious | Not Serious | None^b^ | 20/  937 (2.1%) | 56/  939 (6.0%) | HR 0.42 (0.25 - 0.73) | 35 fewer per 1000  (from 45 fewer to 16 fewer) | ⨁⨁⨁◯  Moderate |
| CV Mortality or HF Hospitalization/HF Event | | | | | | | | | | |
| 3 | Serious | Not Serious | Not Serious | Not Serious | None^b^ | 46/  937 (4.9%) | 88/  899 (9.8%) | HR 0.56 (0.35 - 0.90) | 43 fewer per 1000  (from 64 fewer to 10 fewer) | ⨁⨁⨁◯  Moderate |
| **ASCVD and Overweight/Obese without T2D** | | | | | | | | | | |
| CV Mortality | | | | | | | | | | |
| 1 | Not Serious | Not Applicable | Not Serious | Serious | None^b^ | 223/  8803 (2.5%) | 262/  8801 (3.0%) | HR 0.85 (0.71 - 1.01) | 5 fewer per 1000 (from 9 fewer to 0 more) | ⨁⨁⨁◯  Moderate |
| All-Cause Mortality | | | | | | | | | | |
| 1 | Not Serious | Not Applicable | Not Serious | Not Serious | None^b^ | 375/  8803 (4.3%) | 458/  8801 (5.2%) | HR 0.81 (0.71 - 0.93) | 10 fewer per 1000 (from 15 fewer to 4 fewer) | ⨁⨁⨁⨁  High |
| HF Hospitalization/Event | | | | | | | | | | |
| 1 | Not Serious | Not Applicable | Not Serious | Serious | None^b^ | 97/  8803 (1.1%) | 122/  8801 (1.4%) | HR 0.79 (0.60 - 1.03) | 3 fewer per 1000  (from 6 fewer to 0 more) | ⨁⨁⨁◯  Moderate |
| Non-Fatal MI | | | | | | | | | | |
| 1 | Not Serious | Not Applicable | Not Serious | Not Serious | None^b^ | 234/  8803 (2.7%) | 322/  8801 (3.7%) | HR 0.72 (0.61 - 0.85) | 10 fewer per 1000 (from 14 fewer to 6 fewer) | ⨁⨁⨁⨁  High |
| Non-Fatal Stroke | | | | | | | | | | |
| 1 | Not Serious | Not Applicable | Not Serious | Serious | None^b^ | 154/  8803 (1.7%) | 165/ 8801 (1.9%) | HR 0.93 (0.74 - 1.15) | 1 fewer per 1000  (from 5 fewer to 3 more) | ⨁⨁⨁◯  Moderate |
| Kidney Composite Outcome | | | | | | | | | | |
| 1 | Not Serious | Not Applicable | Not Serious | Not Serious | None^b^ | 155/ 8803 (1.8%) | 198/  8801 (2.2%) | HR 0.78 (0.63 - 0.96) | 5 fewer per 1000 (from 8 fewer to 1 fewer) | ⨁⨁⨁⨁  High |
| CV Mortality or HF Hospitalization/HF Event | | | | | | | | | | |
| 1 | Not Serious | Not Applicable | Not Serious | Not Serious | None^b^ | 320/  8803 (3.6%) | 384/  8801 (4.4%) | HR 0.83 (0.72 - 0.96) | 7 fewer per 1000 (from 12 fewer to 2 fewer) | ⨁⨁⨁⨁  High |
| MACE | | | | | | | | | | |
| 1 | Not Serious | Not Applicable | Not Serious | Not Serious | None^b^ | 569/  8803 (6.5%) | 701/  8801 (8.0%) | HR 0.80 (0.72 - 0.90) | 16 fewer per 1000 (from 22 fewer to 8 fewer) | ⨁⨁⨁⨁  High |

a = No noticeable asymmetry in funnel plot and Egger’s Test for asymmetry was non-significant (p >0.05).

b = Too few studies (n<10) to assess publication bias via Egger’s Test

c = p <0.05 on funnel plot.

| **SGLT2i** | | | | | | | | | | |
| --- | --- | --- | --- | --- | --- | --- | --- | --- | --- | --- |
| Certainty assessment | | | | | | # of events / # of  patients; Event rate | | Effect | | Certainty |
| No of studies | Risk of Bias | Inconsistency | Indirectness | Imprecision | Other Consideration | SGLT2i | Control | Relative (95% CI) | Absolute  (95%  CI) |  |
| **OVERALL POPULATION** | | | | | | | | | | |
| CV Mortality | | | | | | | | | | |
| 15 | Serious | Not Serious | Not Serious | Not Serious | None^a^ | 2464/  53764 (4.6%) | 2431/  47188 (5.2%) | HR 0.88 (0.82 - 0.93) | 6 fewer per 1000 (from 9 fewer to 4 fewer) | ⨁⨁⨁◯  Moderate |
| All-Cause Mortality | | | | | | | | | | |
| 15 | Serious | Not Serious | Not Serious | Not Serious | None^a^ | 4018/  53764 (7.5%) | 3929/  47188 (8.3%) | HR 0.89 (0.84 - 0.94) | 9 fewer per 1000 (from 13 fewer to 5 fewer) | ⨁⨁⨁◯  Moderate |
| HF Hospitalization/Event | | | | | | | | | | |
| 14 | Serious | Not Serious | Not Serious | Not Serious | None^a^ | 2367/  50460 (4.7%) | 3092/ 43883 (7.0%) | HR 0.70 (0.67 - 0.74) | 21 fewer per 1000  (from 23 fewer to 18 fewer) | ⨁⨁⨁◯  Moderate |
| Non-Fatal MI | | | | | | | | | | |
| 5 | Serious | Not Serious | Not Serious | Not Serious | None^b^ | 1211/  26765 (4.5%) | 994/  20204 (4.9%) | HR 0.90 (0.83 - 0.98) | 5 fewer per 1,000 (from 8 fewer to 1 fewer) | ⨁⨁⨁◯  Moderate |
| Non-Fatal Stroke | | | | | | | | | | |
| 5 | Serious | Not Serious | Not Serious | Serious | None^b^ | 744/  26765 (2.8%) | 560/  20204 (2.8%) | HR 0.99 (0.88 - 1.11) | 0 fewer per 1000  (from 3 fewer to 3 more) | ⨁⨁◯◯  Low |
| Kidney Composite Outcome | | | | | | | | | | |
| 12 | Serious | Serious | Not Serious | Not Serious | None^a^ | 1899/  47835 (4.0%) | 2177/  41303 (5.3%) | HR 0.68  (0.60 - 0.77) | 17 fewer per 1000 (from 21 fewer to 12 fewer) | ⨁⨁◯◯  Low |
| CV Mortality or HF Hospitalization/HF Event | | | | | | | | | | |
| 15 | Serious | Not Serious | Not Serious | Not Serious | None^a^ | 4531/  54029 (8.4%) | 5162/  47453 (10.9%) | HR 0.78 (0.75 - 0.82) | 24 fewer per 1000  (from 27 fewer to 20 fewer) | ⨁⨁⨁◯  Moderate |
| MACE | | | | | | | | | | |
| 10 | Serious | Not Serious | Not Serious | Not Serious | None^a^ | 3646/  40140 (9.1%) | 3352/  33565 (10%) | HR 0.89 (0.84 - 0.93) | 11 fewer per 1000 (from 16 fewer to 7 fewer) | ⨁⨁⨁◯  Moderate |
| **T2D with ASCVD/High CV RIsk** | | | | | | | | | | |
| CV Mortality | | | | | | | | | | |
| 12 | Serious | Not Serious | Not Serious | Not Serious | None^a^ | 1841/  39166 (4.7%) | 1741/  32598 (5.3%) | HR 0.86 (0.80 - 0.93) | 7 fewer per 1000 (from 11 fewer to 4 fewer) | ⨁⨁⨁◯  Moderate |
| All-Cause Mortality | | | | | | | | | | |
| 12 | Serious | Not Serious | Not Serious | Not Serious | None^a^ | 2914/  39319 (7.4%) | 2744/  32754 (0.84%) | HR 0.88 (0.82 - 0.94) | 10 fewer per 1000  (from 15 fewer to 5 fewer) | ⨁⨁⨁◯  Moderate |
| HF Hospitalization/Event | | | | | | | | | | |
| 12 | Serious | Not Serious | Not Serious | Not Serious | None^a^ | 1798/  38791 (4.6%) | 2293/  32232 (7.1%) | HR 0.70 (0.66 - 0.75) | 21 fewer per 1000  (from 24 fewer to 18 fewer) | ⨁⨁⨁◯  Moderate |
| Non-Fatal MI | | | | | | | | | | |
| 5 | Serious | Not Serious | Not Serious | Not Serious | None^b^ | 1211/  26765 (4.5%) | 994/  20204 (4.9%) | HR 0.90 (0.83 - 0.98) | 5 fewer per 1,000 (from 8 fewer to 1 fewer) | ⨁⨁⨁◯  Moderate |
| Non-Fatal Stroke | | | | | | | | | | |
| 5 | Serious | Not Serious | Not Serious | Serious | None^b^ | 744/  26765 (2.8%) | 560/  20204 (2.8%) | HR 0.99 (0.88 - 1.11) | 0 fewer per 1000  (from 3 fewer to 3 more) | ⨁⨁◯◯  Low |
| Kidney Composite Outcome | | | | | | | | | | |
| 12 | Serious | Serious | Not Serious | Not Serious | None^a^ | 1899/  47835 (4.0%) | 2177/  41303 (5.3%) | HR 0.68  (0.60 - 0.77) | 17 fewer per 1000 (from 21 fewer to 12 fewer) | ⨁⨁◯◯  Low |
| CV Mortality or HF Hospitalization/HF Event | | | | | | | | | | |
| 12 | Serious | Not Serious | Not Serious | Not Serious | Serious^c^ | 3314/  39166 (8.5%) | 3694/  32598 (11.3%) | HR 0.77  (0.73 - 0.80) | 26 fewer per 1000  (from 31 fewer to 23 fewer) | ⨁⨁◯◯  Low |
| MACE | | | | | | | | | | |
| 7 | Serious | Not Serious | Not Serious | Not Serious | None^b^ | 3246/  32665 (9.9%) | 2924/  26110 (11.2%) | HR 0.88  (0.82 - 0.93) | 13 fewer per 1000  (from 20 fewer to 8 fewer) | ⨁⨁⨁◯  Moderate |
| **Chronic Kidney Disease** | | | | | | | | | | |
| CV Mortality | | | | | | | | | | |
| 10 | Serious | Not Serious | Not Serious | Not Serious | None^a^ | 947/  19623 (4.8%) | 1041/  18803 (5.5%) | HR 0.85 (0.78 - 0.93) | 8 fewer per 1000 (from 12 fewer to 4 fewer) | ⨁⨁⨁◯  Moderate |
| All-Cause Mortality | | | | | | | | | | |
| 9 | Serious | Not Serious | Not Serious | Not Serious | None^b^ | 68/720 (9.4%) | 83/738 (11.2%) | HR 0.83 (0.77 - 0.89) | 19 fewer per 1000  (from 26 fewer to 12 fewer) | ⨁⨁⨁◯  Moderate |
| HF Hospitalization | | | | | | | | | | |
| 11 | Serious | Not Serious | Not Serious | Not Serious | None^a^ | 1211/  19005 (6.4%) | 1672/  17379 (9.6%) | HR 0.66 (0.60 - 0.73) | 33 fewer per 1000  (from 39 fewer to 26 fewer) | ⨁⨁⨁◯  Moderate |
| Non-Fatal MI | | | | | | | | | | |
| 3 | Serious | Not Serious | Not Serious | Not Serious | None^b^ | 155/  3918 (4.0%) | 197/  3787 (5.2%) | HR 0.77  (0.62 - 0.95) | 12 fewer per 1000 (from 20 fewer to 3 fewer) | ⨁⨁⨁◯  Moderate |
| Non-Fatal Stroke | | | | | | | | | | |
| 3 | Serious | Serious | Not Serious | Serious | None^b^ | 103/  3918 (2.6%) | 129/  3787 (3.4%) | HR 0.78  (0.50 - 1.23) | 8 fewer per 1000 (from 17 fewer to 8 more) | ⨁◯◯◯  Very Low |
| Kidney Composite Outcome | | | | | | | | | | |
| 12 | Serious | Not Serious | Not Serious | Not Serious | None^a^ | 1134/  23746 (4.8%) | 1479/  21924 (6.7%) | HR 0.67  (0.60 - 0.75) | 22 fewer per 1000 (from 27 fewer to 17 fewer) | ⨁⨁⨁◯  Moderate |
| CV Mortality or HF Hospitalization/HF Event | | | | | | | | | | |
| 12 | Serious | Not Serious | Not Serious | Not Serious | None^a^ | 2015/  20468 (9.8%) | 2618/  20403 (12.8%) | HR 0.75  (0.70 - 0.79) | 32 fewer per 1000 (from 39 fewer to 27 fewer) | ⨁⨁⨁◯  Moderate |
| MACE | | | | | | | | | | |
| 6 | Serious | Not Serious | Not Serious | Not Serious | None^b^ | 1178/  14439 (8.2%) | 1259/  13497 (9.3%) | HR 0.85  (0.78 - 0.92) | 14 fewer per 1000  (from 21 fewer to 8 fewer) | ⨁⨁⨁◯  Moderate |
| **HFpEF** | | | | | | | | | | |
| CV Mortality | | | | | | | | | | |
| 4 | Serious | Not Serious | Not Serious | Serious | None^b^ | 551/  7470 (7.4%) | 564/  7104 (7.9%) | HR 0.96  (0.82 - 1.13) | 3 fewer per 1000 (from 14 fewer to 10 more) | ⨁⨁◯◯  Low |
| All-Cause Mortality | | | | | | | | | | |
| 4 | Serious | Not Serious | Not Serious | Serious | None^b^ | 1066/  7470 (14.3%) | 1064/  7104 (15.0%) | HR 0.97  (0.89 - 1.06) | 4 fewer per 1000 (17 fewer to 9 more) | ⨁⨁◯◯  Low |
| HF Hospitalization/Event | | | | | | | | | | |
| 4 | Serious | Not Serious | Not Serious | Not Serious | None^b^ | 667/  7470 (8.9%) | 856/  7104 (12.0%) | HR 0.74  (0.67 - 0.82) | 31 fewer per 1000 (from 40 fewer to 22 fewer) | ⨁⨁⨁◯  Moderate |
| Kidney Composite Outcome | | | | | | | | | | |
| 2 | Serious | Not Serious | Not Serious | Serious | None^b^ | 187/6128 (3.1%) | 185/  6122 (3.0%) | HR 1.00  (0.82 - 1.23 | 0 fewer per 1000  (from 5 fewer to 7 more) | ⨁⨁◯◯  Low |
| CV Mortality or HF Hospitalization/HF Event | | | | | | | | | | |
| 6 | Serious | Not Serious | Not Serious | Not Serious | None^b^ | 1237/  8849 (14.0%) | 1476/  8467 (17.4%) | HR 0.79  (0.73 - 0.86) | 37 fewer per 1000  (from 47 fewer to 24 fewer) | ⨁⨁⨁◯  Moderate |
| **HFrEF** | | | | | | | | | | |
| CV Mortality | | | | | | | | | | |
| 4 | Serious | Not Serious | Not Serious | Not Serious | None^b^ | 481/  4873 (9.9%) | 543/  4750 (11.4%) | HR 0.84  (0.71 - 0.98) | 18 fewer per 1000  (from 33 fewer to 2 fewer) | ⨁⨁⨁◯  Moderate |
| All-Cause Mortality | | | | | | | | | | |
| 4 | Serious | Not Serious | Not Serious | Not Serious | None^b^ | 617/  4873 (12.7%) | 690/  4750 (14.5%) | HR 0.84  (0.72 - 0.97) | 23 fewer per 1000  (from 41 fewer to 4 fewer) | ⨁⨁⨁◯  Moderate |
| HF Hospitalization/Event | | | | | | | | | | |
| 4 | Serious | Not Serious | Not Serious | Not Serious | None^b^ | 551/  4873 (11.3%) | 749/  4750 (15.8%) | HR 0.69  (0.64 - 0.75) | 49 fewer per 1000 (from 57 fewer to 39 fewer) | ⨁⨁⨁◯  Moderate |
| Kidney Composite Outcome | | | | | | | | | | |
| 2 | Serious | Not Serious | Not Serious | Not Serious | None^b^ | 58/  4236 (1.4%) | 97/  4238 (2.3%) | HR 0.59  (0.42 - 0.83) | 9 fewer per 1000  (from 13 fewer to 4 fewer) | ⨁⨁⨁◯  Moderate |
| CV Mortality or HF Hospitalization/HF Event | | | | | | | | | | |
| 6 | Serious | Not Serious | Not Serious | Not Serious | None^b^ | 1031/  5739 (18.0%) | 1294/  5642 (22.9%) | HR 0.75  (0.69 - 0.81) | 57 fewer per 1000 (from 71 fewer to 44 fewer) | ⨁⨁⨁◯  Moderate |
| **Post-MI** | | | | | | | | | | |
| CV Mortality | | | | | | | | | | |
| 2 | Serious | Not Serious | Not Serious | Serious | None^b^ | 159/  5279 (3.0%) | 154/  5260 (2.9%) | HR 1.05 (0.83 - 1.31) | 1 more per 1000  (from 5 fewer to 9 more) | ⨁⨁◯◯  Low |
| All-Cause Mortality | | | | | | | | | | |
| 2 | Serious | Not Serious | Not Serious | Serious | None^b^ | 210/  5279 (4.0%) | 211/  5260 (4.0%) | HR 1.00 (0.83 - 1.21) | 1 more per 1000  (from 7 fewer to 8 more) | ⨁⨁◯◯  Low |
| HF Hospitalization | | | | | | | | | | |
| 2 | Serious | Not Serious | Not Serious | Not Serious | None^b^ | 145/  5279 (2.7%) | 185/  5260 (3.5%) | HR 0.78 (0.63 - 0.97) | 8 fewer per 1000  (from 13 fewer to 1 fewer) | ⨁⨁⨁◯  Moderate |
| CV Mortality or HF Hospitalization | | | | | | | | | | |
| 2 | Serious | Not Serious | Not Serious | Serious | None^b^ | 281/  5279 (5.3%) | 311/  5260 (5.9%) | HR 0.91 (0.77 - 1.07) | 5 fewer per 1000 (from 14 fewer to 4 more) | ⨁⨁◯◯  Low |
| MACE | | | | | | | | | | |
| 1 | Serious | Not Applicable | Not Serious | Serious | None^b^ | 68/2019 (3.4%) | 72/1998 (3.6%) | HR 0.97 (0.67 - 1.31) | 1 fewer per 1000 (from 12 fewer to 11 more) | ⨁⨁◯◯  Low |
| **Acute HF** | | | | | | | | | | |
| CV Mortality | | | | | | | | | | |
| 1 | Serious | Not Applicable | Not Serious | Serious | None^b^ | 51/608 (8.4%) | 58/614 (9.4%) | HR 0.84 (0.58 - 1.22) | 15 fewer per 1000  (from 40 fewer to 21 more) | ⨁⨁◯◯  Low |
| All-Cause Mortality | | | | | | | | | | |
| 1 | Serious | Not Applicable | Not Serious | Serious | None^b^ | 65/608 (10.7%) | 76/614 (12.4%) | HR 0.82 (0.59 - 1.14) | 22 fewer per 1000  (from 51 fewer to 17 more) | ⨁⨁◯◯  Low |
| HF Hospitalization (total event analysis) | | | | | | | | | | |
| 1 | Serious | Not Applicable | Not Serious | Not Serious | None^b^ | 194/608 (31.9%) | 297/614 (48.4%) | HR 0.64 (0.49 - 0.83) | 139 fewer per 1000  (from 207 fewer to 61 fewer) | ⨁⨁⨁◯  Moderate |
| CV Mortality or HF Hospitalization/Event (total event analysis) | | | | | | | | | | |
| 2 | Serious | Not Serious | Not Serious | Not Serious | None^b^ | 269/873 (30.8%) | 404/879 (46%) | HR 0.67 (0.54 - 0.84) | 152 fewer per 1000  (from 211 fewer to 74 fewer) | ⨁⨁⨁◯  Moderate |
| MACE (total event analysis) | | | | | | | | | | |
| 1 | Serious | Not Applicable | Not Serious | Not Serious | None^b^ | 247/608 (40.6%) | 330/614 (53.7%) | HR 0.72 (0.56 - 0.92) | 150 fewer per 1000  (from 236 fewer to 43 fewer) | ⨁⨁⨁◯  Moderate |

a = No noticeable asymmetry in funnel plot and Egger’s Test for asymmetry was non-significant (p >0.05).

b = Too few studies (n<10) to assess publication bias via Egger’s Test

c = p <0.05 on funnel plot.

| **Finerenone** | | | | | | | | | | |
| --- | --- | --- | --- | --- | --- | --- | --- | --- | --- | --- |
| Certainty assessment | | | | | | # of events / # of  patients; Event rate | | Effect | | Certainty |
| No of studies | Risk of Bias | Inconsistency | Indirectness | Imprecision | Other Consideration | Finerenone | Control | Relative (95% CI) | Absolute  (95%  CI) |  |
| **Finerenone - 3 Trials Pooled** | | | | | | | | | | |
| CV Mortality | | | | | | | | | | |
| 3 | Not Serious | Not Serious | Not Serious | Serious | None^b^ | 564/  9522 | 624/  9505 | 0.90 (0.84 to 0.91) | 7 fewer per 1000 (from 11 fewer to 6 fewer) | ⨁⨁⨁◯  Moderate |
| All-Cause Mortality | | | | | | | | | | |
| 3 | Not Serious | Not Serious | Not Serious | Not Serious | None^b^ | 1043/  9522 | 1136/  9505 | 0.91 (0.84 to 0.99) | 11 fewer per 1000 (from 19 fewer to 1 fewer per 1000) | ⨁⨁⨁⨁  High |
| Kidney Composite Outcome | | | | | | | | | | |
| 3 | Not Serious | Serious | Not Serious | Serious | None^b^ | 929/  9522 | 1050/  9505 | 0.92 (0.76 to 1.10) | 9 fewer per 1000 (from 27 fewer to 11 more per 1000) | ⨁⨁◯◯  Low |
| **Diabetic Kidney Disease** | | | | | | | | | | |
| CV Mortality | | | | | | | | | | |
| 2 | Not Serious | Not Serious | Not Serious | Serious | None^b^ | 322/  6519 | 364/  6507 | 0.88 (0.76 to 1.02) | 7 fewer per 1000 (from 13 fewer to 1 more per 1000) | ⨁⨁⨁◯  Moderate |
| All-Cause Mortality | | | | | | | | | | |
| 2 | Not Serious | Not Serious | Not Serious | Serious | None^b^ | 552/  6519 | 614/  6507 | 0.89 (0.80 to 1.00) | 10 fewer per 1000 (from 19 fewer to 0 per 1000) | ⨁⨁⨁◯  Moderate |
| HF Hospitalization | | | | | | | | | | |
| 2 | Not Serious | Not Serious | Not Serious | Not Serious | None^b^ | 256/  6519 | 325/  6507 | 0.78 (0.65 to 0.94) | 11 fewer per 1000 (from 18 fewer to 3 fewer per 1000) | ⨁⨁⨁⨁  High |
| Non-Fatal MI | | | | | | | | | | |
| 2 | Not Serious | Not Serious | Not Serious | Serious | None^b^ | 173/  6519 | 189/  6507 | 0.90 (0.74 to 1.11) | 3 fewer per 1000 (from 8 fewer to 3 more per 1000) | ⨁⨁⨁◯  Moderate |
| Non-Fatal Stroke | | | | | | | | | | |
| 2 | Not Serious | Not Serious | Not Serious | Serious | None^b^ | 198/  6519 | 198/  6507 | 1.0 (0.82 to 1.21) | 0 fewer per 1000 (from 6 fewer to 6 more per 1000) | ⨁⨁⨁◯  Moderate |
| Kidney Composite Outcome | | | | | | | | | | |
| 2 | Not Serious | Not Serious | Not Serious | Not Serious | None^b^ | 854/  6519 | 995/  6507 | 0.84 (0.77 to 0.92) | 25 fewer per 1000 (from 35 fewer to 12 fewer per 1000) | ⨁⨁⨁⨁  High |
| CV Mortality or HF Hospitalization | | | | | | | | | | |
| 2 | Not Serious | Not Serious | Not Serious | Not Serious | None^b^ | 578/  6519 | 689/  6507 | 0.83 (0.71 to 0.97) | 18 fewer per 1000 (from 31 fewer to 3 fewer per 1000) | ⨁⨁⨁⨁  High |
| **HFpEF** | | | | | | | | | | |
| CV Mortality | | | | | | | | | | |
| 2 | Not Serious | Not Serious | Not Serious | Serious | None^b^ | 262/  3198 | 282/  3239 | 0.94 (0.80 to 1.12) | 5 fewer per 1000  (from 17 fewer to 10 more) | ⨁⨁⨁◯  Moderate |
| All-Cause Mortality | | | | | | | | | | |
| 1 | Not Serious | Not Applicable | Not Serious | Serious | None^b^ | 491/  3003 | 522/  2998 | 0.93 (0.83 to 1.06) | 12 fewer per 1000 (from 30 fewer to 10 more per 1000) | ⨁⨁⨁◯  Moderate |
| HF Hospitalization | | | | | | | | | | |
| 2 | Not Serious | Not Serious | Not Serious | Not Serious | None^b^ | 52/  485 | 83/  522 | 0.68 (0.47 to 0.96) | 51 fewer per 1000  (from 84 fewer to 6 fewer) | ⨁⨁⨁⨁  High |
| Non-Fatal MI | | | | | | | | | | |
| 1 | Not Serious | Not Applicable | Not Serious | Serious | None^b^ | 7/195 | 13/241 | 0.60 (0.24 to 1.51) | 22 fewer per 1000 (from 41 fewer to 28 more per 1000) | ⨁⨁⨁◯  Moderate |
| Non-Fatal Stroke | | | | | | | | | | |
| 1 | Not Serious | Not Applicable | Not Serious | Serious | None^b^ | 6/195 (3.1%) | 6/241  (2.5%) | 1.24 (0.40 to 3.85) | 6 more per 1000  (from 15 fewer to 71 more) | ⨁⨁⨁◯  Moderate |
| Kidney Composite Outcome | | | | | | | | | | |
| 1 | Not Serious | Not Applicable | Not Serious | Serious | None^b^ | 75/  3003 | 55/  2998 | 1.33 (0.94 to 1.89) | 6 more per 1000 (from 1 fewer to 16 more per 1000) | ⨁⨁⨁◯  Moderate |
| CV Mortality or HF Hospitalization/Event | | | | | | | | | | |
| 3 | Not Serious | Not Serious | Not Serious | Not Serious | None^b^ | 584/  3488 (16.7%) | 647/  3520 (18.4%) | HR 0.84 (0.76 - 0.92) | 29 fewer per 1000  (from 44 fewer to 15 fewer) | ⨁⨁⨁⨁  High |

Supplemental Table S4. GRADE evidence profile, summarizing certainty of evidence, relative and absolute risk reductions, and effect estimates across key outcomes. a = No noticeable asymmetry in funnel plot and Egger’s Test for asymmetry was non-significant (p >0.05). b = Too few studies (n<10) to assess publication bias via Egger’s Test.

**Search Strategy**

**MEDLINE**

1 exp diabetes mellitus, type 2/ 189998

2 diabetes mellitus type 2.tw. 3086

3 ((type 2 adj2 diabetes) or Obesity or atherosclerotic cardiovascular disease).tw. 506986

4 or/1-3 565504

5 (canagliflozin or empagliflozin or dapagliflozin or sodium glucose cotransporter?1 inhibitor? or SGLT?1 inhibitor? or SGLT?2 inhibitor? or sglt inhibitor? or sodium glucose cotransporter inhibitor? or glucagon-like peptide?1 agonist? or glp?1 agonist? or glp agonist? or Dulaglutide or Semaglutide or Exenatide or Liraglutide or Lixisenatide or albiglutide or finerenone or tirzepatide or non-steroidal mineralocorticoid receptor antagonists?).mp. 23621

6 exp myocardial infarction/ or myocardial infarction.tw. 297049

7 exp stroke/ or stroke.tw. 385937

8 (adverse cardiovascular event? or adverse cardiac event?).tw. or exp death/ or death?.tw. or mortality.tw. 2102817

9 exp heart failure/ or heart failure.mp. 286269

10 exp Renal Insufficiency, Chronic/ 144536

11 (chronic kidney disease? or ckd or kidney failure or renal failure).tw. 192269

12 or/6-11 2939820

13 or/6-11 2939820

14 randomized controlled trial.pt. 638825

15 controlled clinical trial.pt. 95703

16 Random Allocation/ 108475

17 Double-Blind Method/ 184268

18 single-blind method/ 35059

19 Placebos/ 36103

20 "Research Design"/ 131469

21 evaluation studies/ 265956

22 Comparative Study/ 1950070

23 exp Longitudinal Studies/ 184743

24 cross-over studies/ 59211

25 clinical trial.tw. 223303

26 clinical trial.pt. 541452

27 latin square.tw. 5741

28 (time adj series).tw. 53501

29 (before adj2 after adj (stud$ or trial$ or design$)).tw. 6149

30 placebo$.tw. 266994

31 random$.tw. 1627713

32 (matched communities or matched schools or matched populations).tw. 414

33 control$.tw. 5071854

34 (comparison group$ or control group$).tw. 662020

35 matched pairs.tw. 8652

36 (outcome study or outcome studies).tw. 9443

37 ((singl$ or doubl$ or trebl$ or tripl$) adj (blind$ or mask)).tw. 209531

38 (quasiexperimental or quasi experimental or pseudo experimental).tw. 25376

39 (nonrandomi?ed or non randomi?ed or pseudo randomi?sed or quasi randomi?ed).tw. 44456

40 prospective$.tw. 977107

41 volunteer$.tw. 229152

42 or/14-41 8870827

43 13 and 42 962351

44 4 and 5 and 43 2723

45 limit 44 to yr="2023 -Current" 1033

**Cochrane Central Register of Controlled Trials (CENTRAL)**

1 exp diabetes mellitus, type 2/ 26313

2 diabetes mellitus type 2.tw. 1388

3 ((type 2 adj2 diabetes) or Obesity or atherosclerotic cardiovascular disease).tw. 77815

4 or/1-3 83813

5 (canagliflozin or empagliflozin or dapagliflozin or sodium glucose cotransporter?1 inhibitor? or SGLT?1 inhibitor? or SGLT?2 inhibitor? or sglt inhibitor? or sodium glucose cotransporter inhibitor? or glucagon-like peptide?1 agonist? or glp?1 agonist? or glp agonist? or Dulaglutide or Semaglutide or Exenatide or Liraglutide or Lixisenatide or albiglutide or finerenone or tirzepatide or non-steroidal mineralocorticoid receptor antagonists?).mp. 11776

6 exp myocardial infarction/ or myocardial infarction.tw. 36567

7 exp stroke/ or stroke.tw. 69451

8 (adverse cardiovascular event? or adverse cardiac event?).tw. or exp death/ or death?.tw. or mortality.tw. 163164

9 exp heart failure/ or heart failure.mp. 38749

10 exp Renal Insufficiency, Chronic/ 9751

11 (chronic kidney disease? or ckd or kidney failure or renal failure).tw. 21894

12 or/6-11 267052

13 or/6-11 267052

14 randomized controlled trial.pt. 0

15 controlled clinical trial.pt. 0

16 Random Allocation/ 25546

17 Double-Blind Method/ 167137

18 single-blind method/ 27001

19 Placebos/ 26475

20 "Research Design"/ 15354

21 evaluation studies/ 1

22 Comparative Study/ 4

23 exp Longitudinal Studies/ 8819

24 cross-over studies/ 47592

25 clinical trial.tw. 207635

26 clinical trial.pt. 0

27 latin square.tw. 1144

28 (time adj series).tw. 1374

29 (before adj2 after adj (stud$ or trial$ or design$)).tw. 990

30 placebo$.tw. 380401

31 random$.tw. 1290136

32 (matched communities or matched schools or matched populations).tw. 68

33 control$.tw. 1028546

34 (comparison group$ or control group$).tw. 306812

35 matched pairs.tw. 1287

36 (outcome study or outcome studies).tw. 1761

37 ((singl$ or doubl$ or trebl$ or tripl$) adj (blind$ or mask)).tw. 335826

38 (quasiexperimental or quasi experimental or pseudo experimental).tw. 6584

39 (nonrandomi?ed or non randomi?ed or pseudo randomi?sed or quasi randomi?ed).tw. 11072

40 prospective$.tw. 242108

41 volunteer$.tw. 82690

42 or/14-41 1702198

43 13 and 42 219743

44 4 and 5 and 43 2172

45 limit 44 to yr="2023 -Current" 517

### **OVERALL POPULATION**

**Supplemental Figure S1 – Cardiovascular Mortality (Overall)**

DerSimonian and Laird:


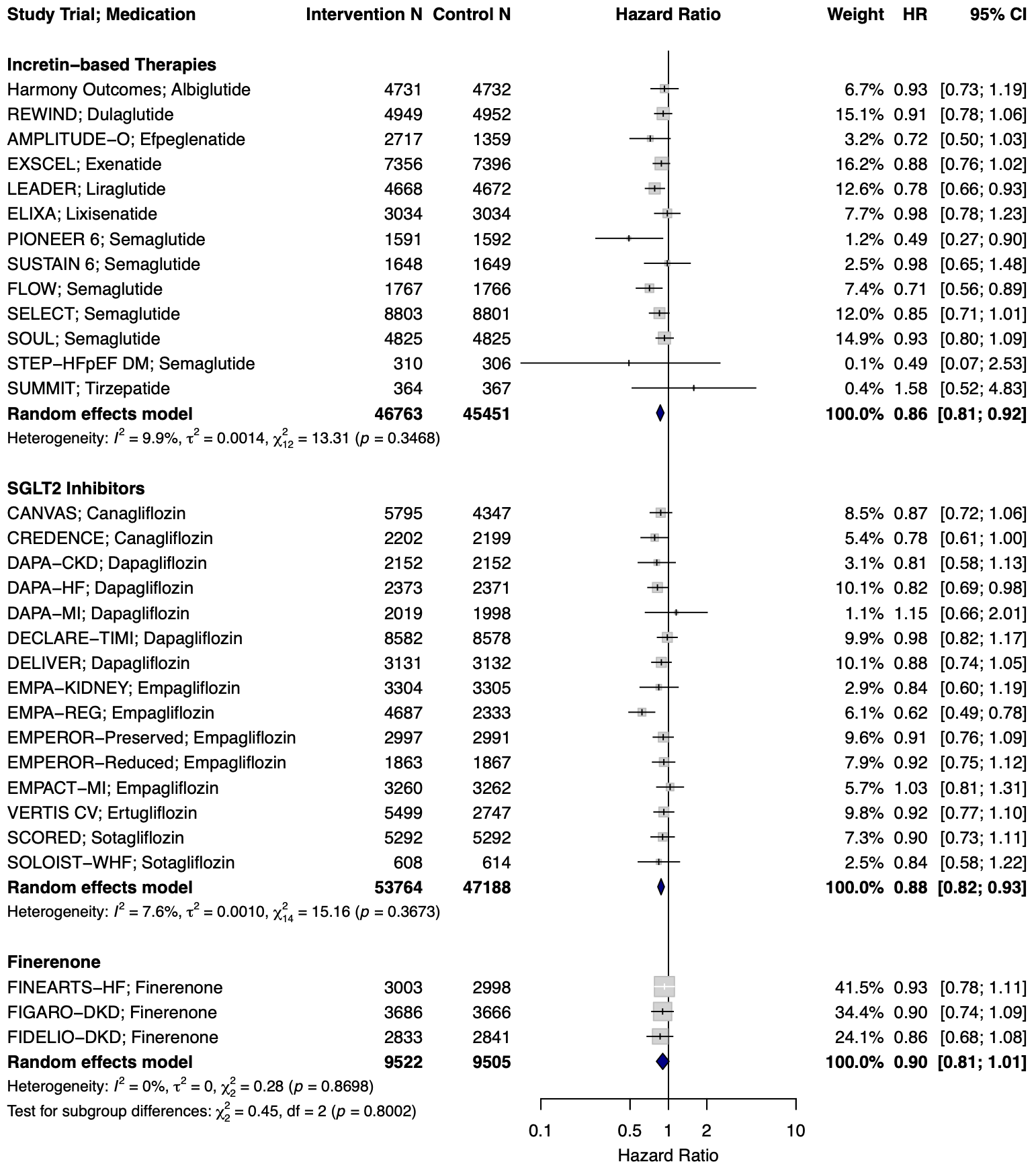


REML–modified HK:


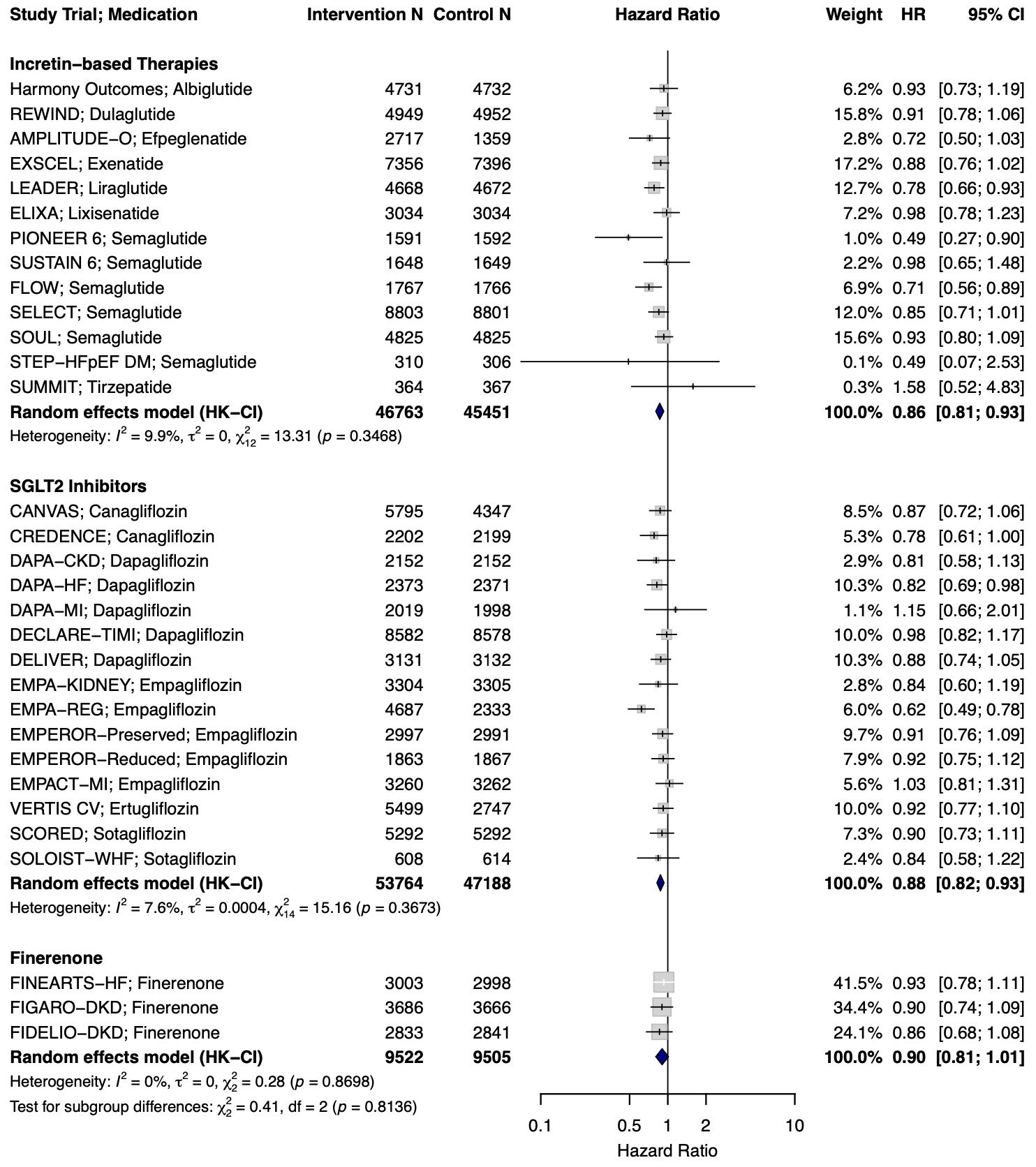


REML–Wald:


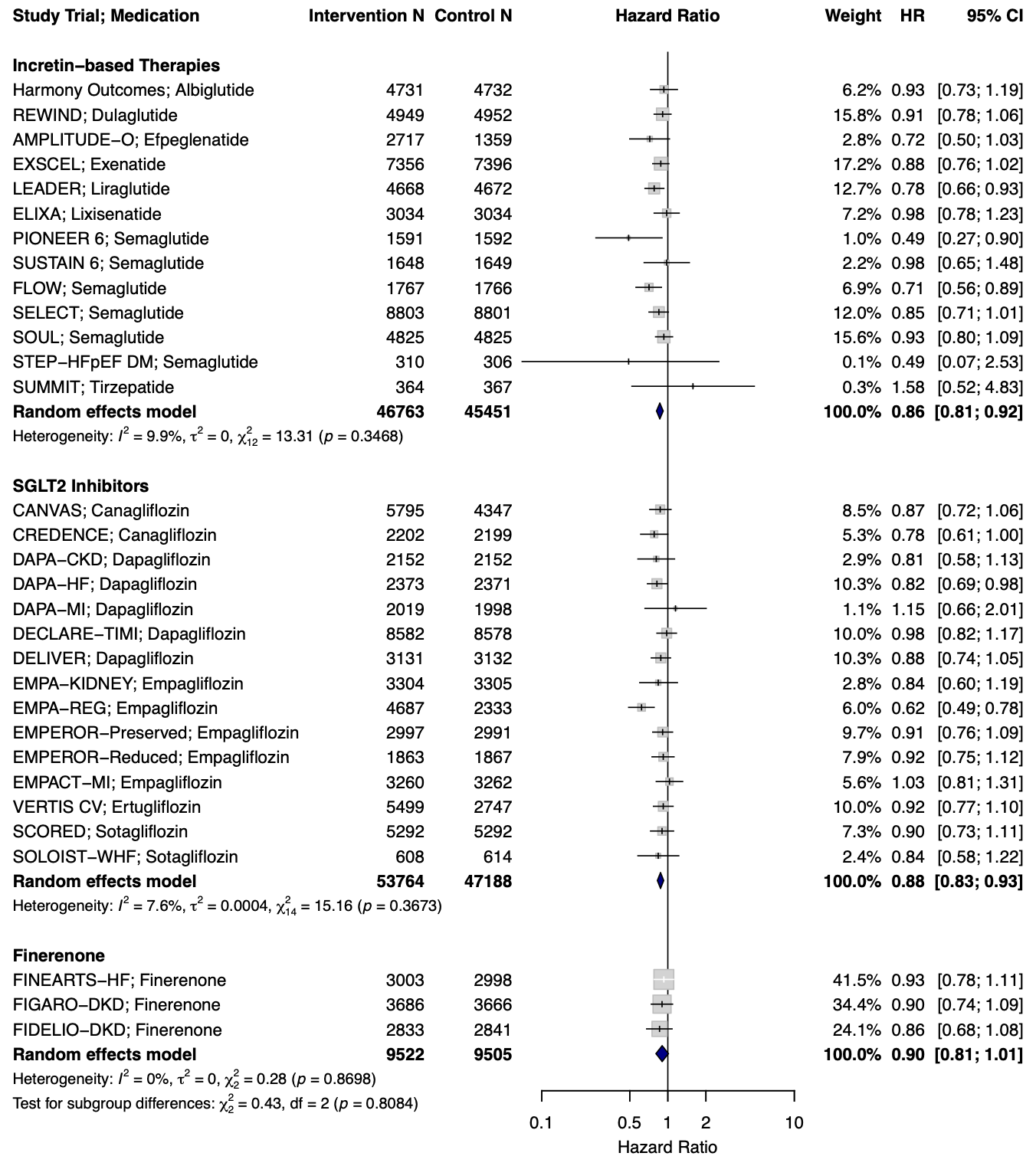


**Supplemental Figure S2 – All-Cause Mortality (Overall)**

DerSimonian and Laird:

**
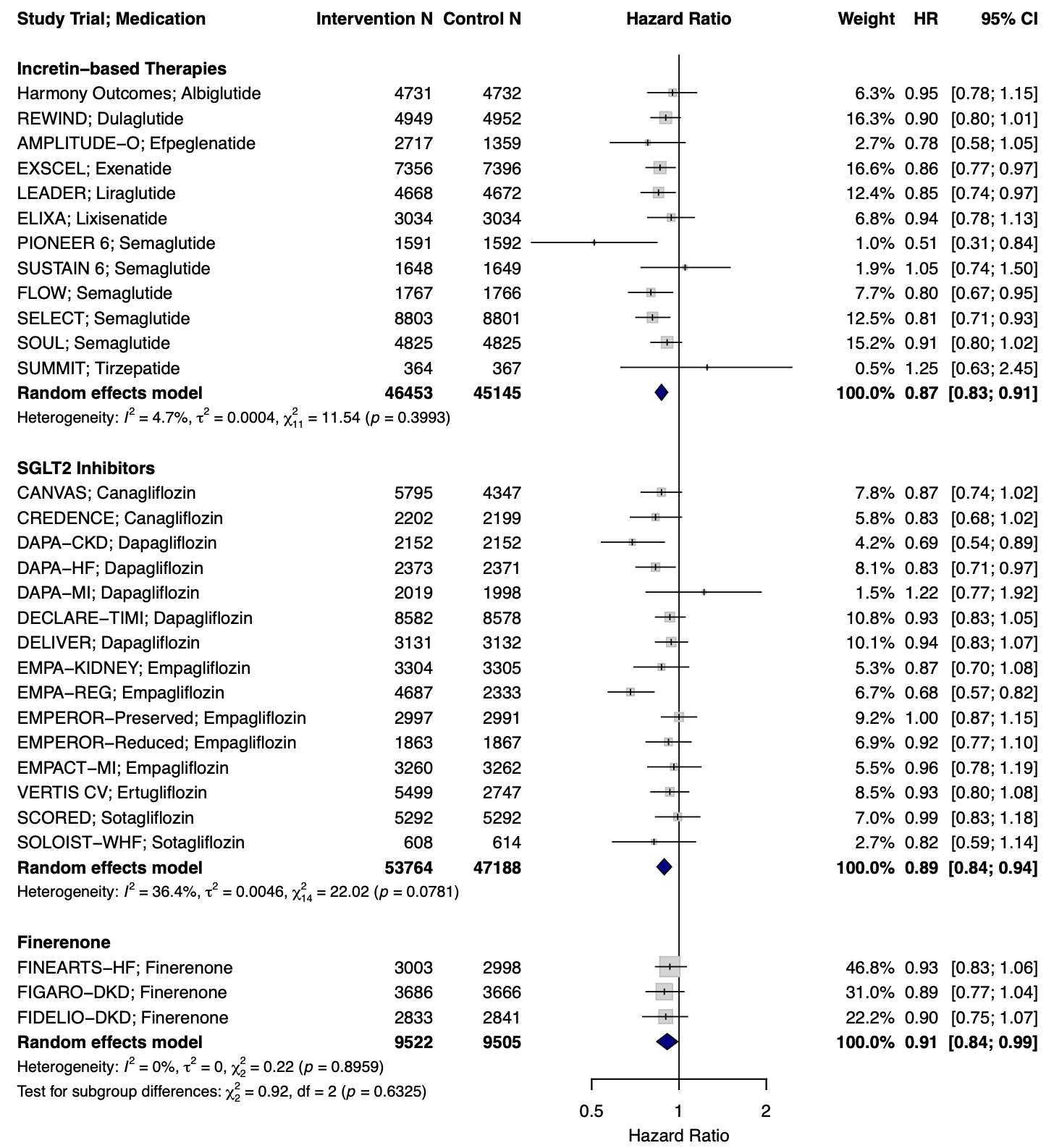
**

REML–modified HK:

**
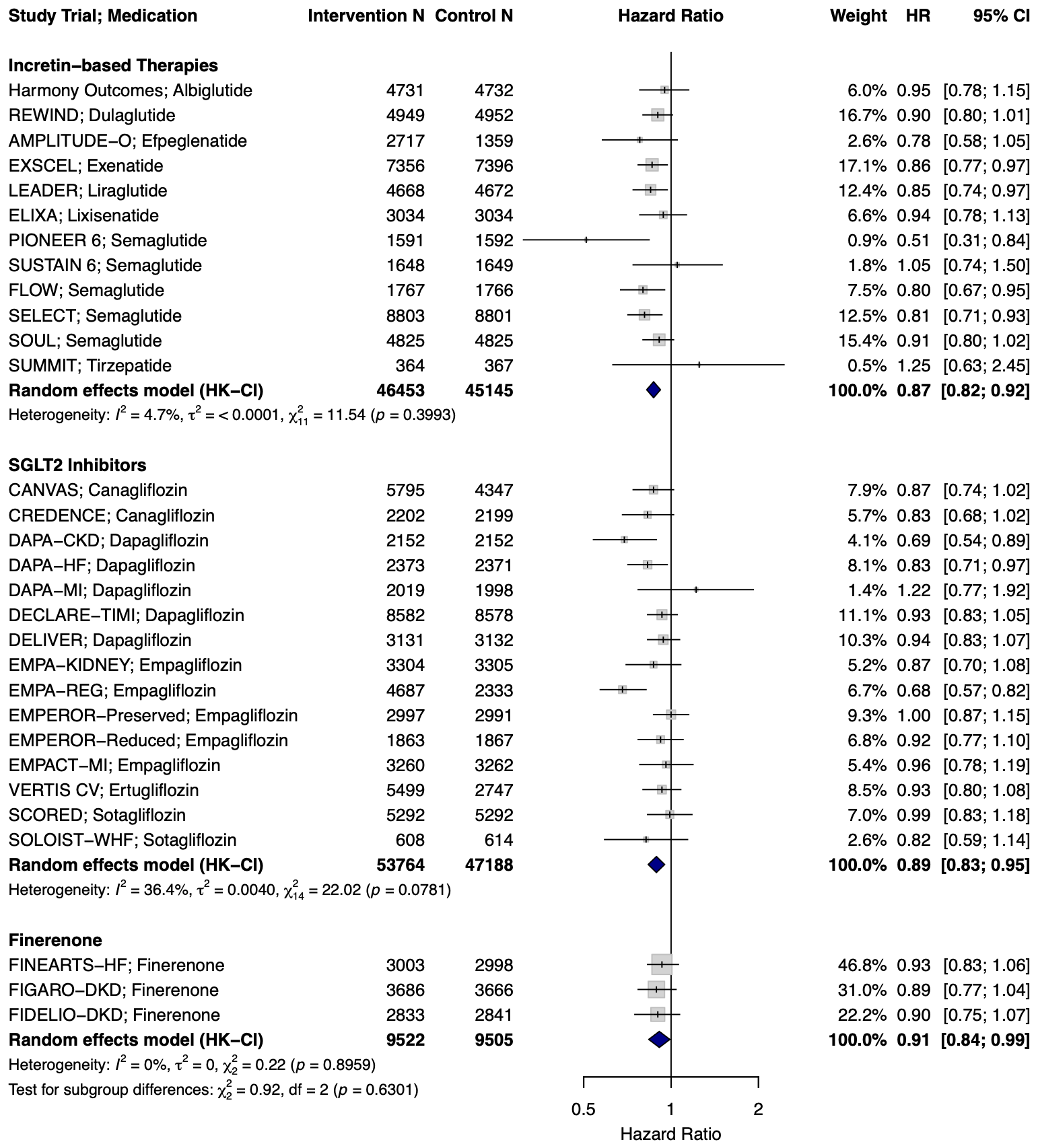
**

REML–Wald:

**
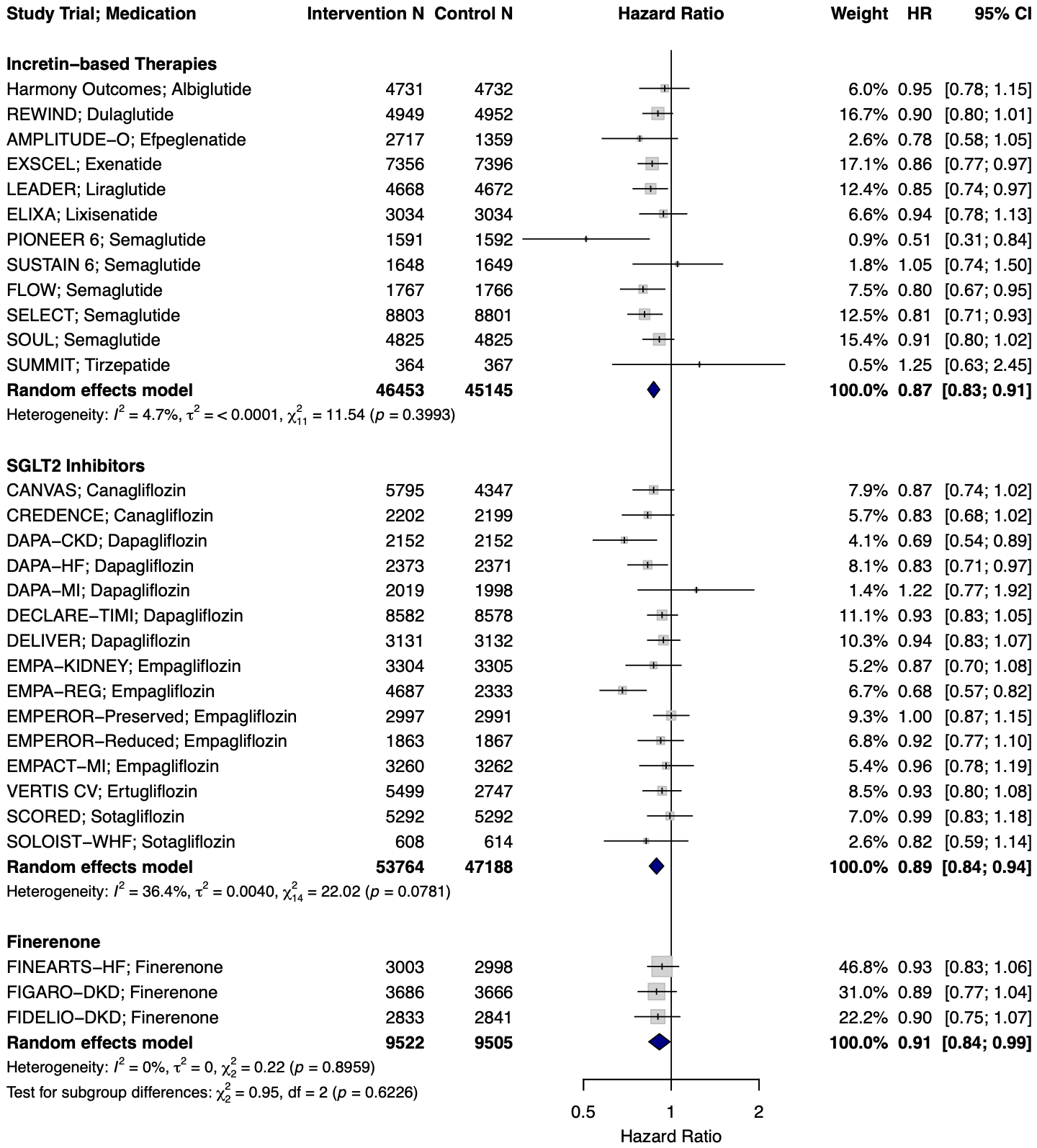
**

**Supplemental Figure S3 – CV Mortality or HF Hospitalization/HF Events (Overall)**

DerSimonian and Laird:


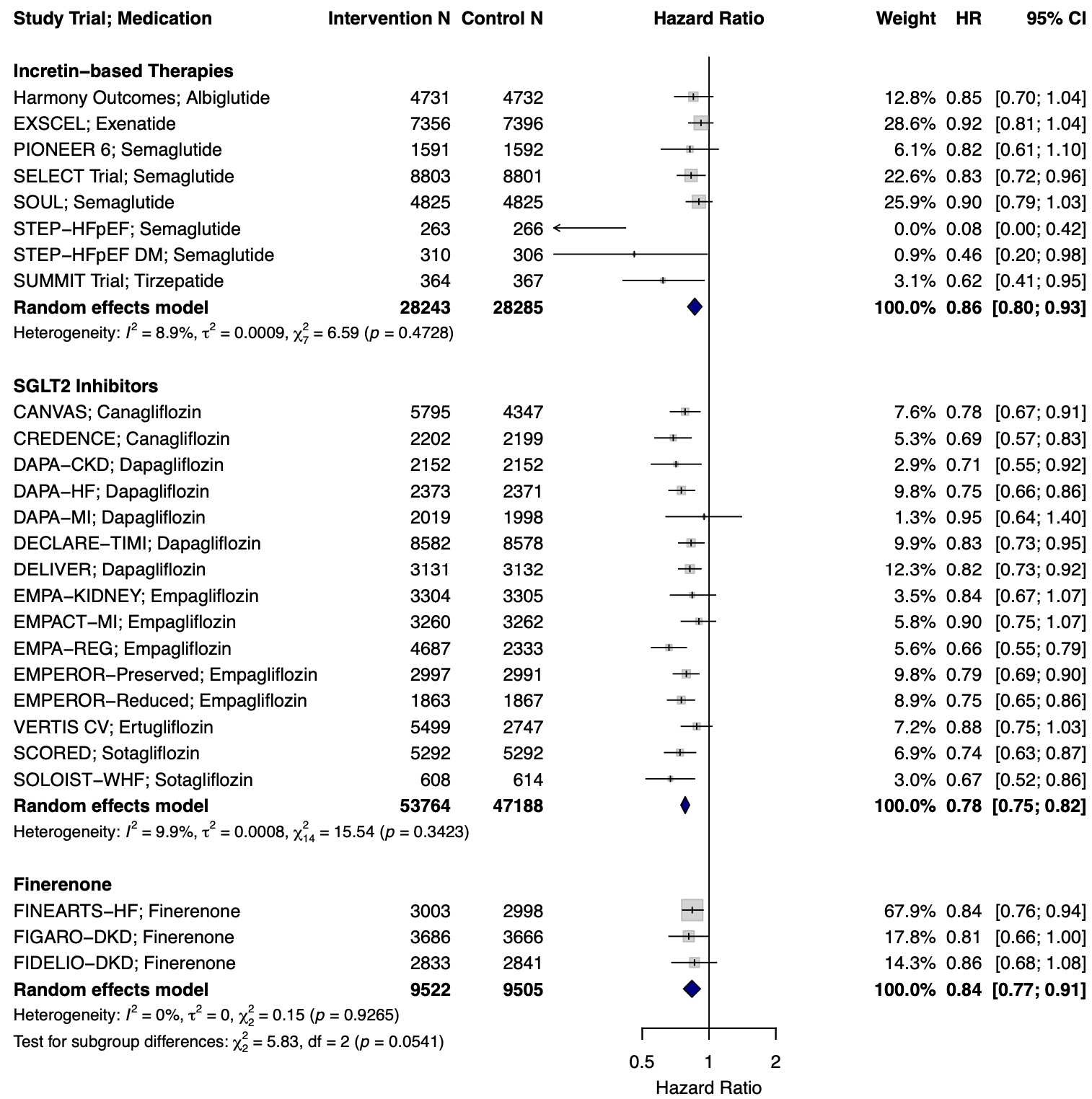


REML–modified HK:

**
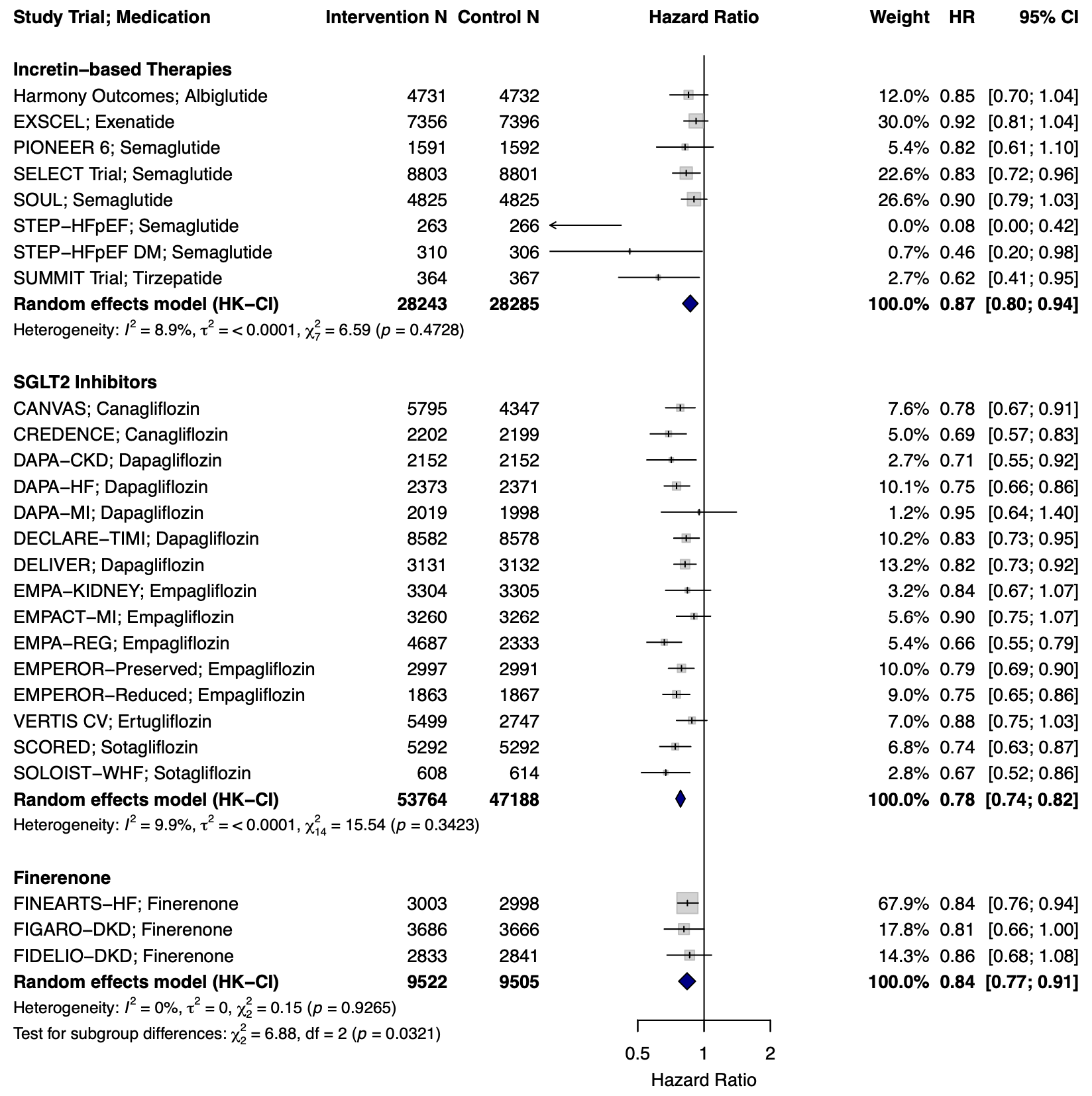
**

REML–Wald:

**
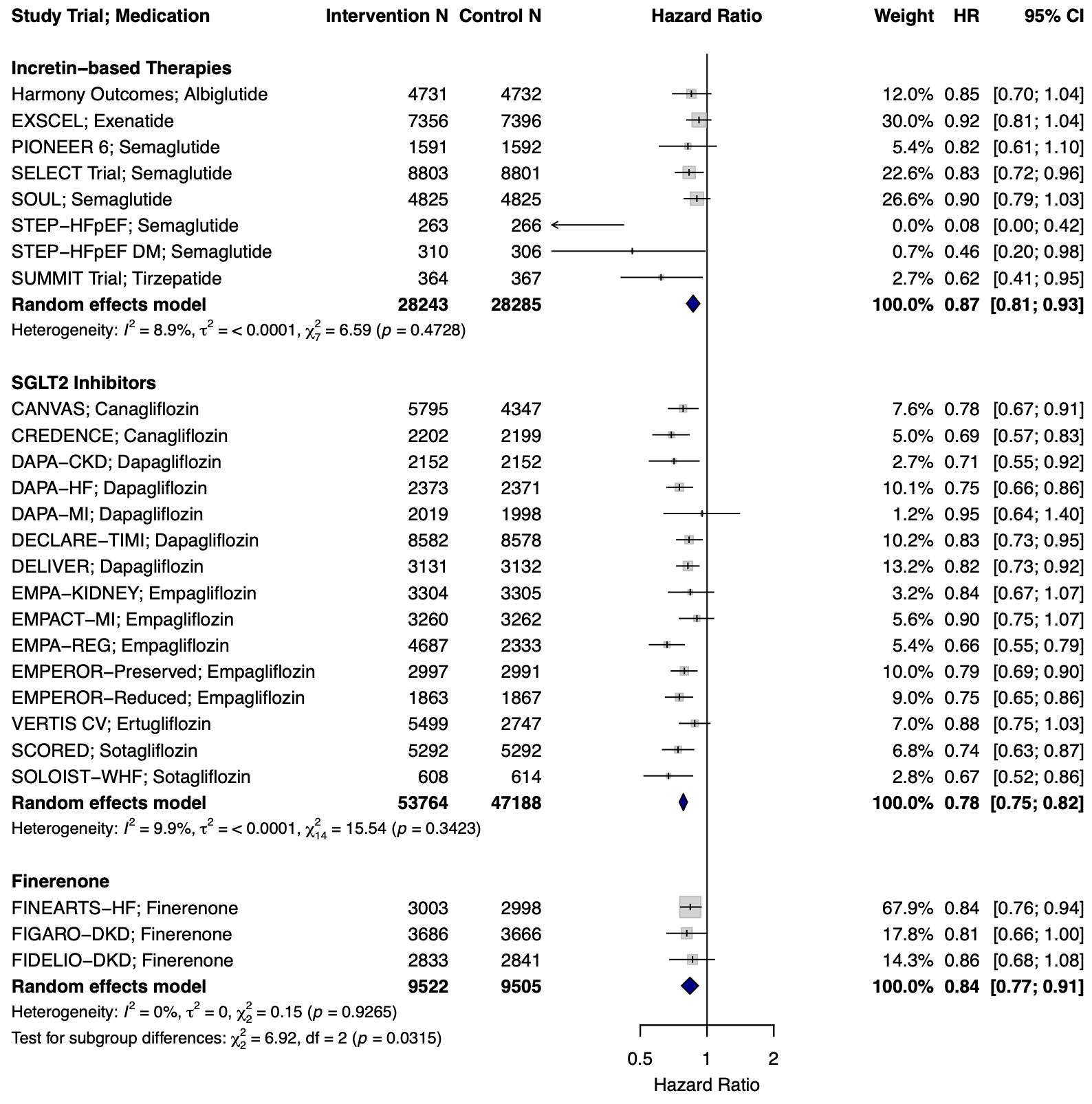
**

**Supplemental Figure S4 – CV Mortality or HF Hospitalization (Overall)**

DerSimonian and Laird:


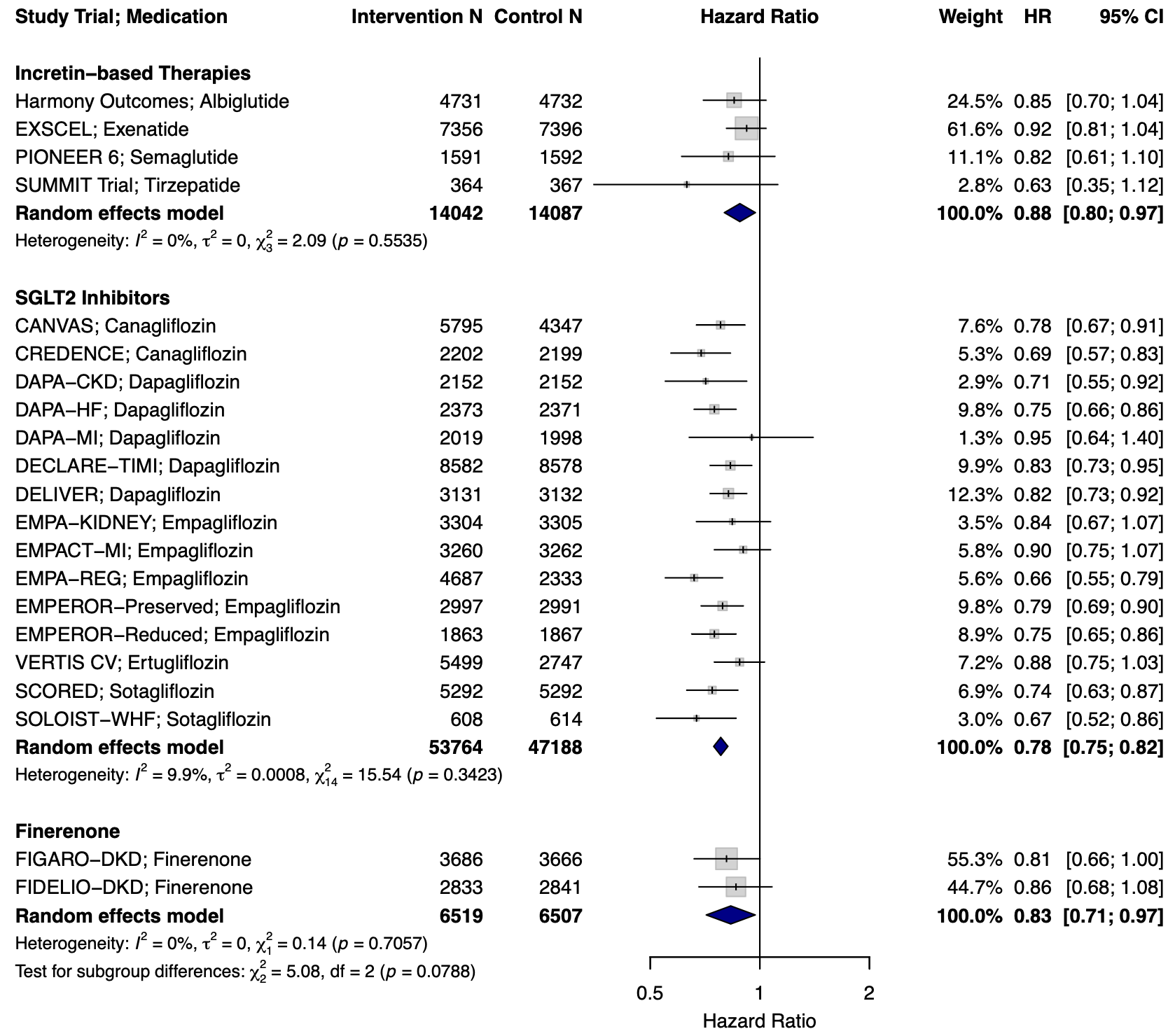


REML–modified HK:

**
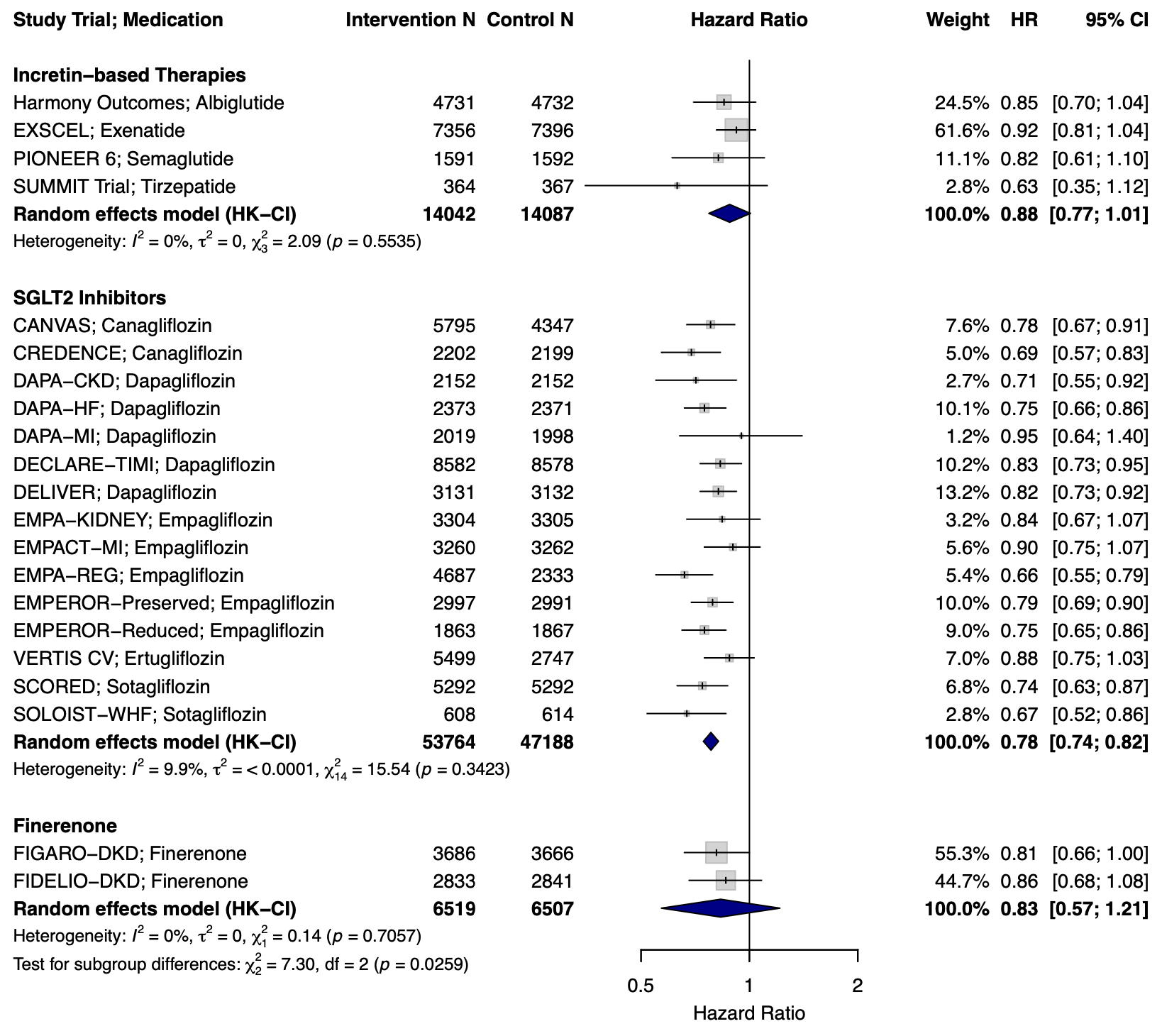
**

REML–Wald:

**
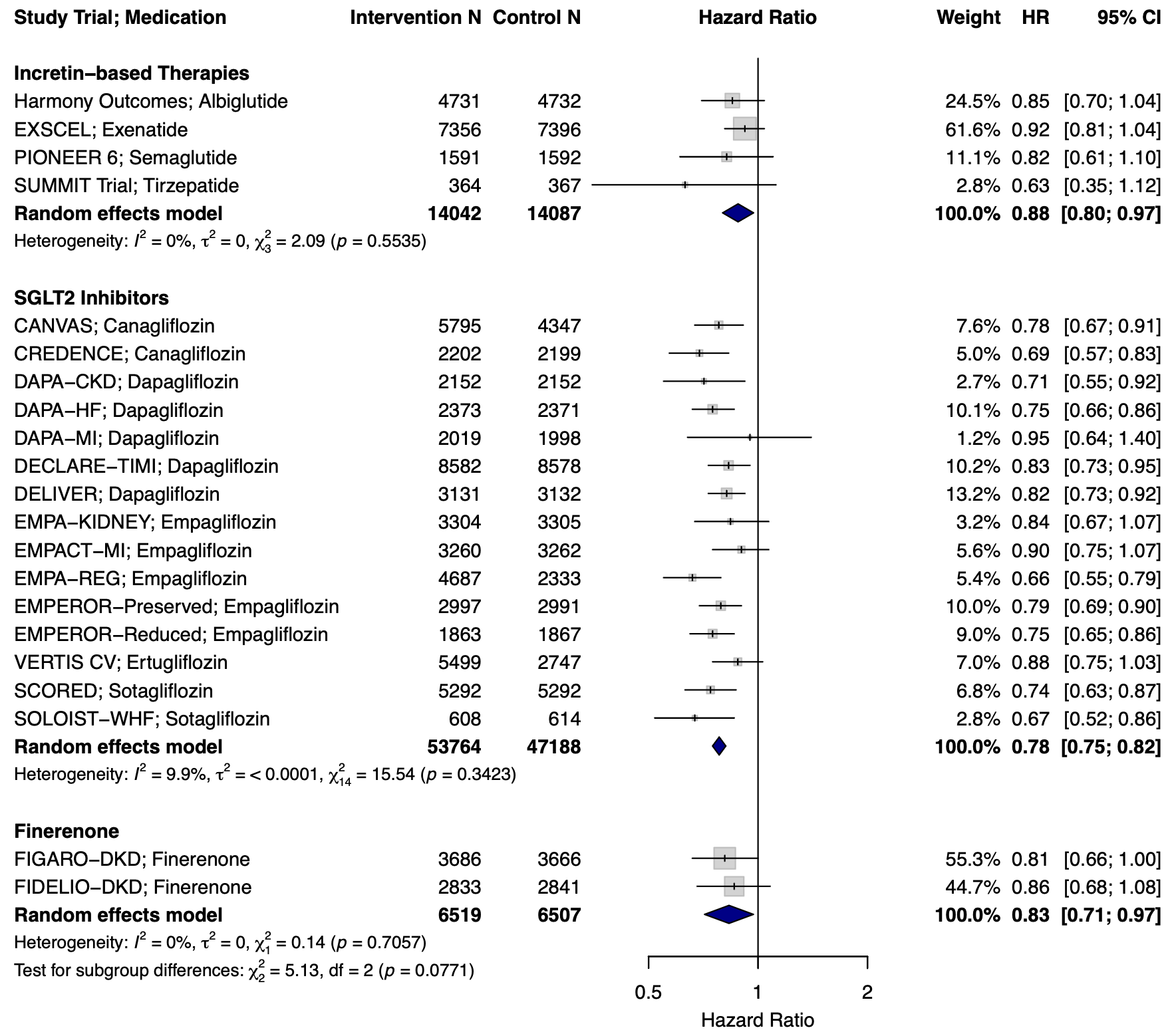
**

**Supplemental Figure S5 – HF Hospitalization/HF Events (Overall)**

DerSimonian and Laird:


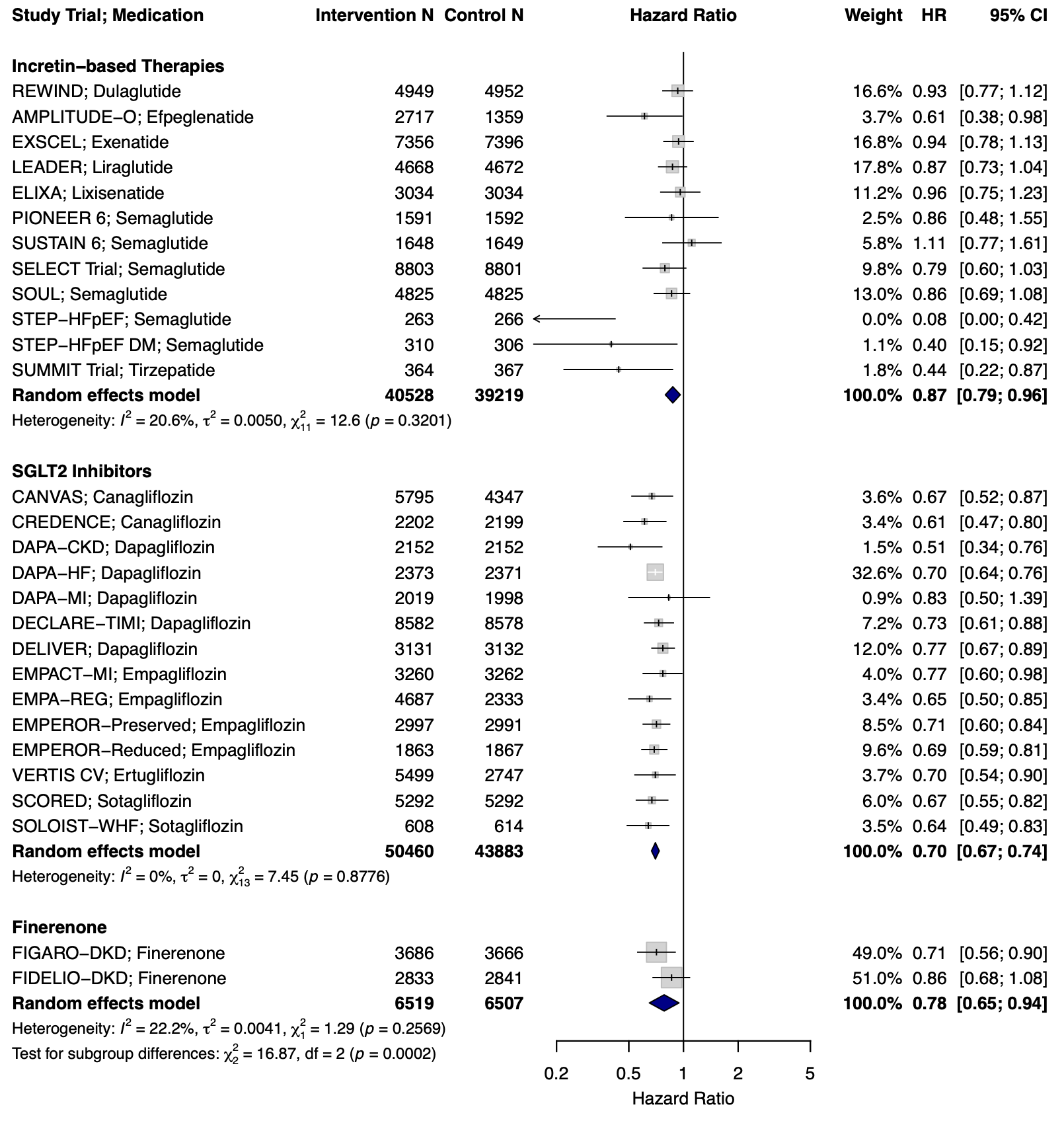


REML–modified HK:

**
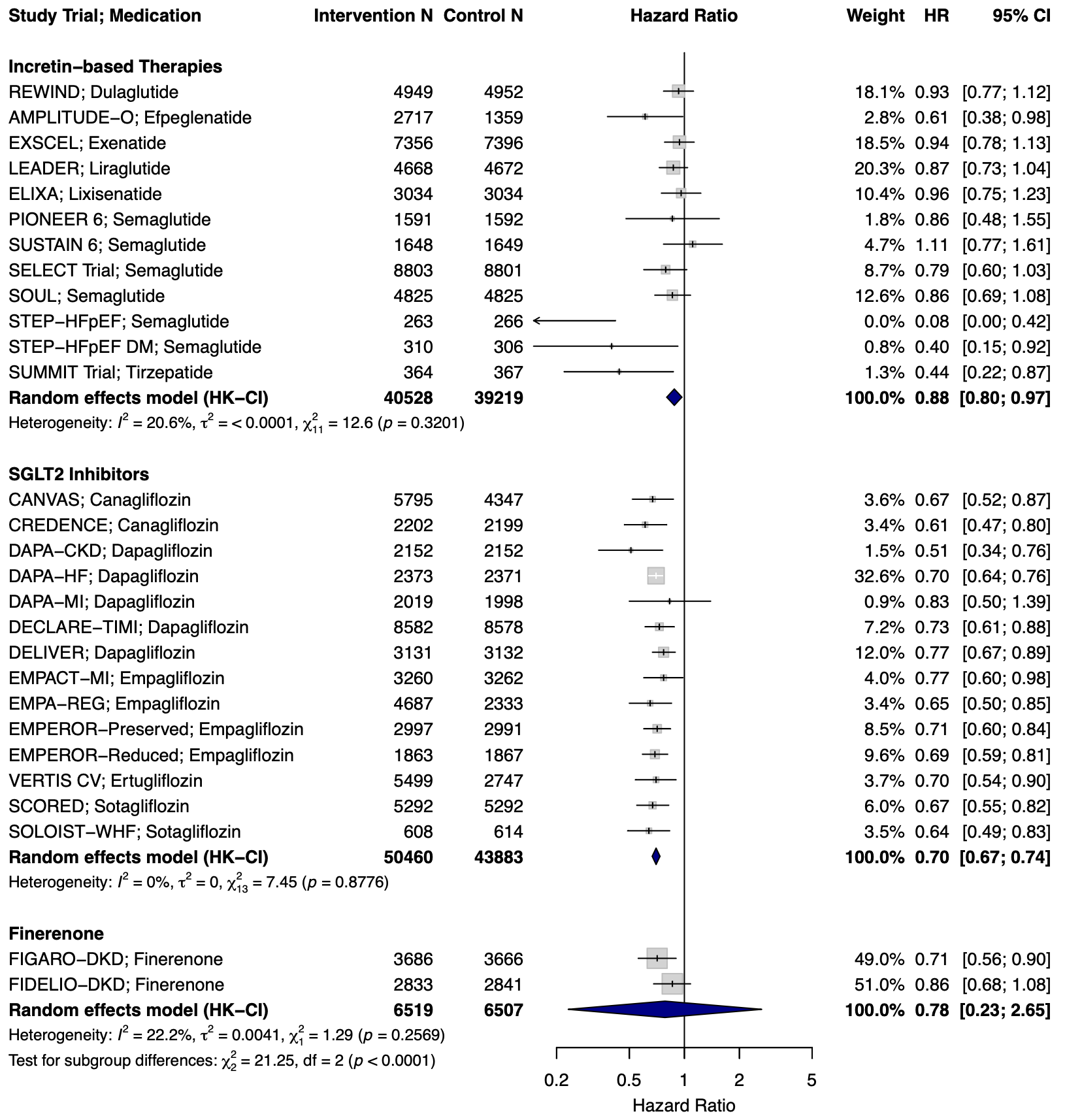
**

REML–Wald:

**
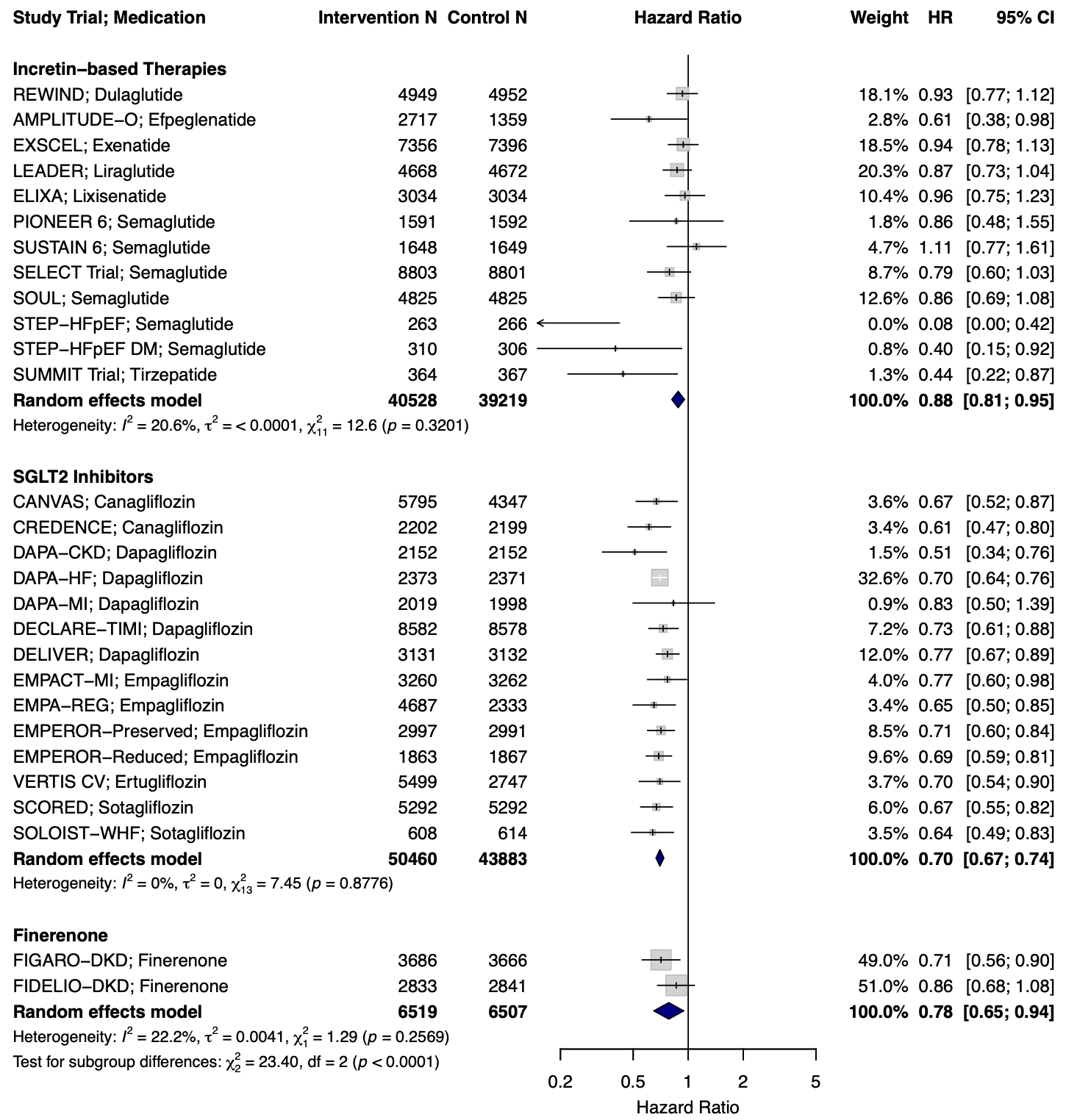
**

**Supplemental Figure S6 – HF Hospitalization (Overall)**

DerSimonian and Laird:


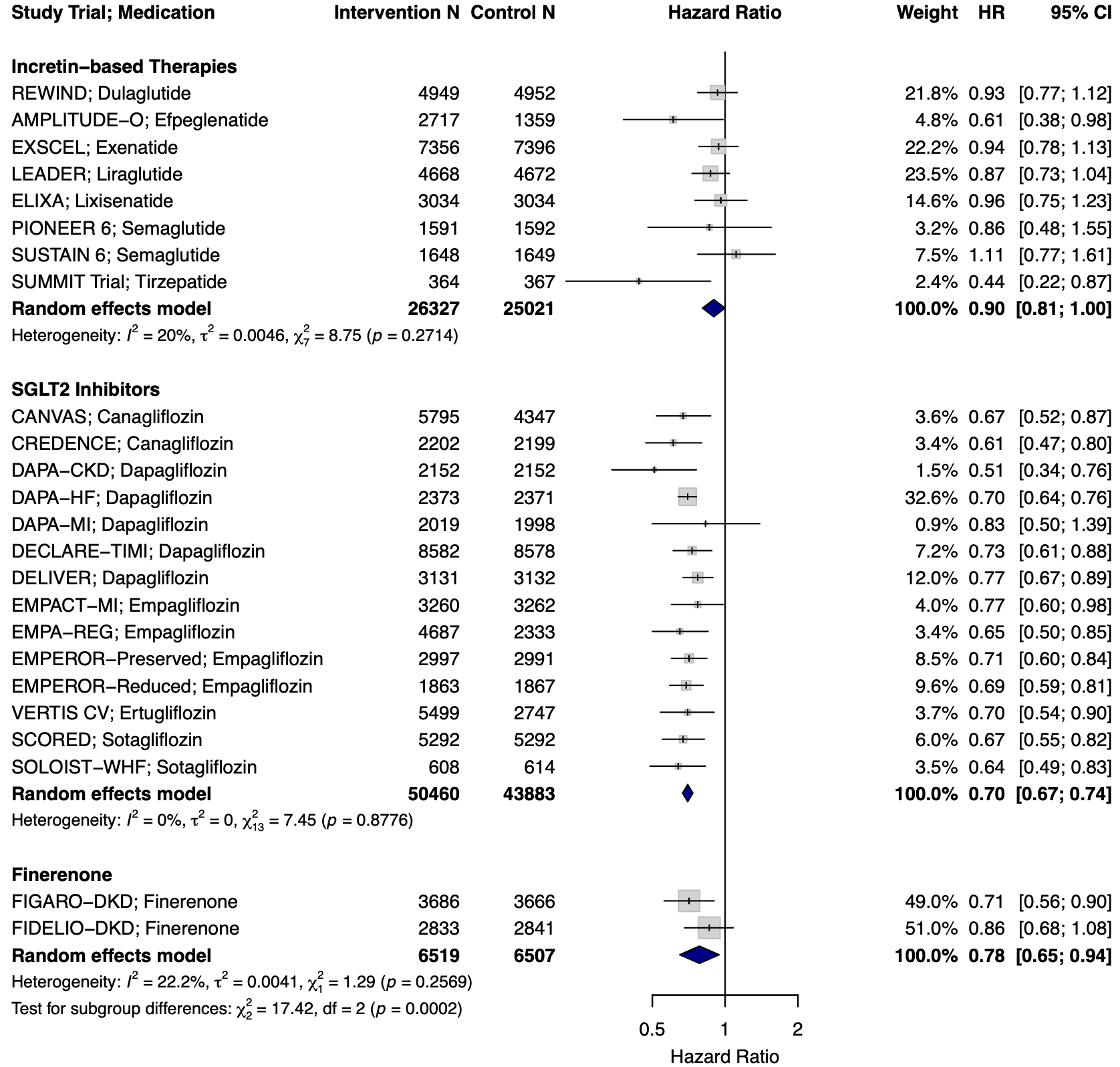


REML–modified HK:

**
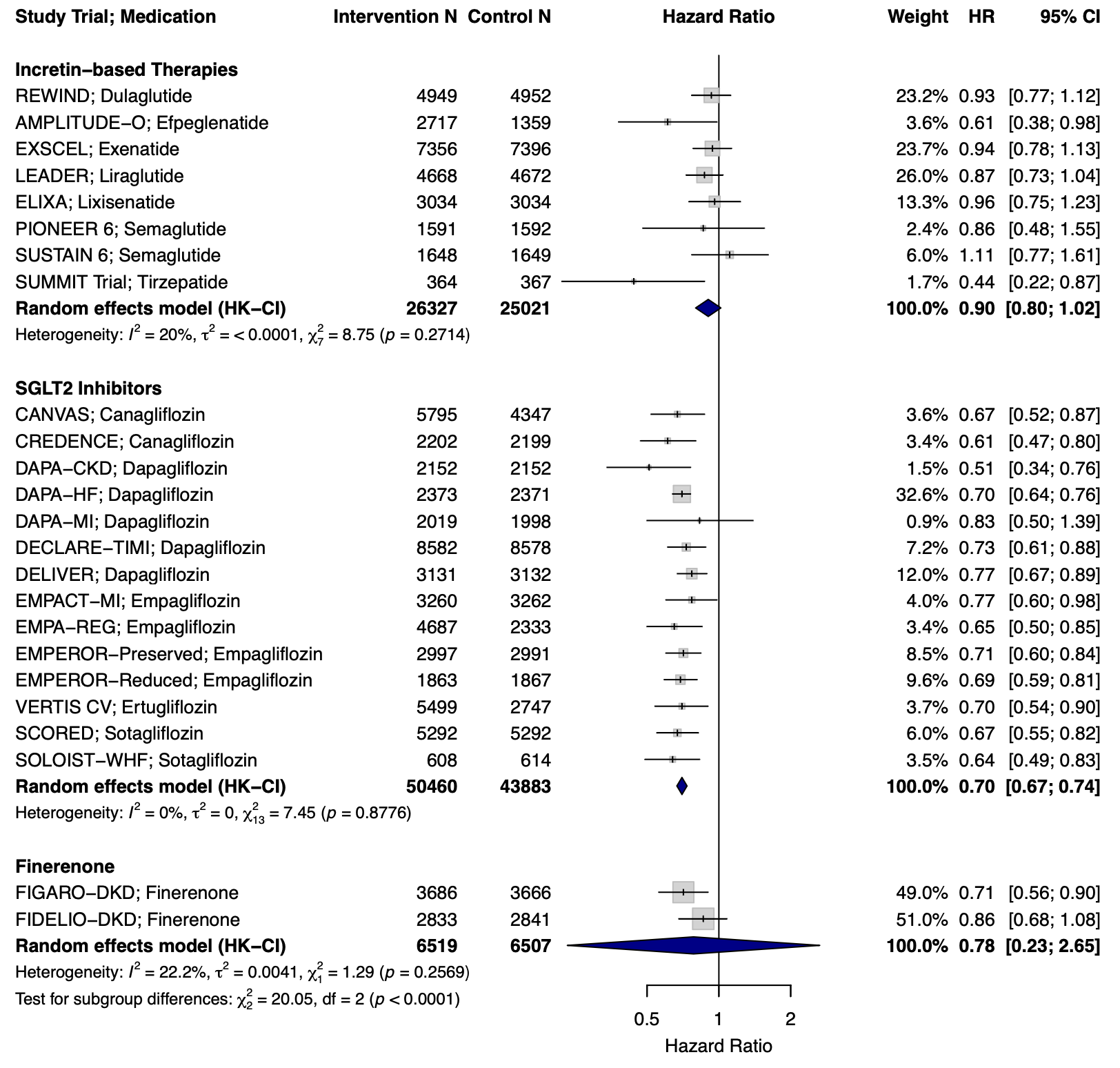
**

REML–Wald:

**
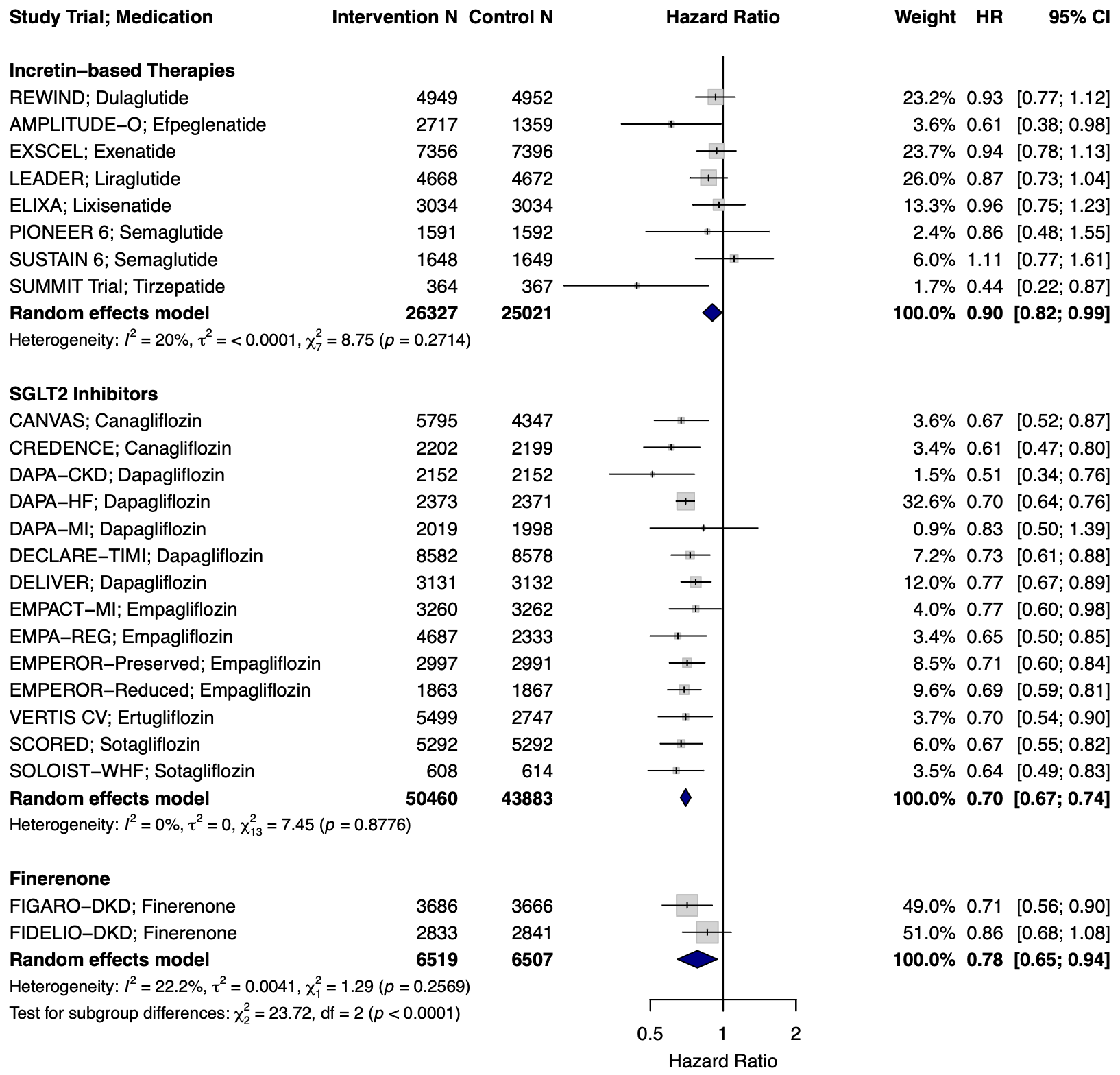
**

**Supplemental Figure S7 – Non-fatal Myocardial Infarction (Overall)**

DerSimonian and Laird:


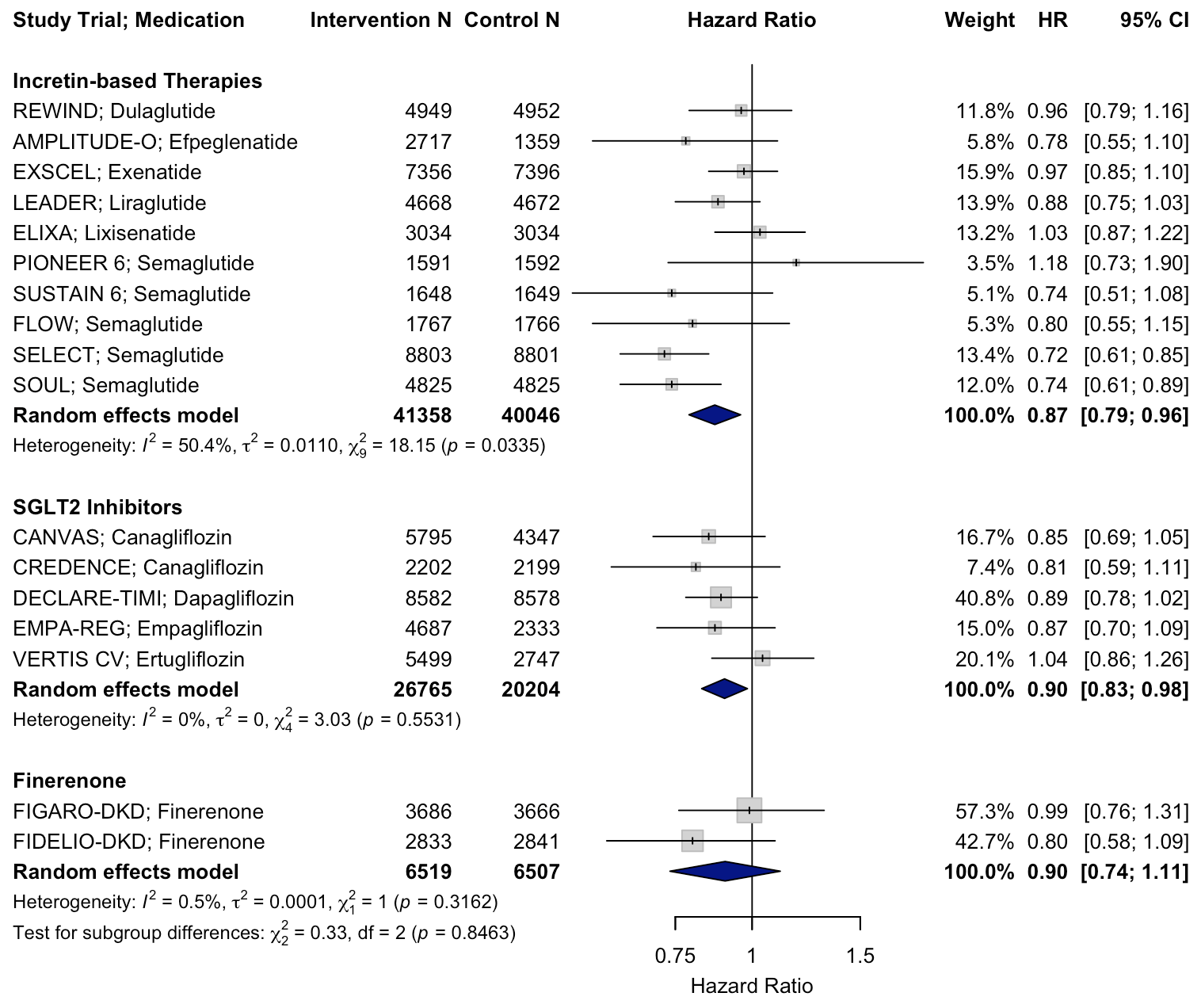


REML–modified HK:

**
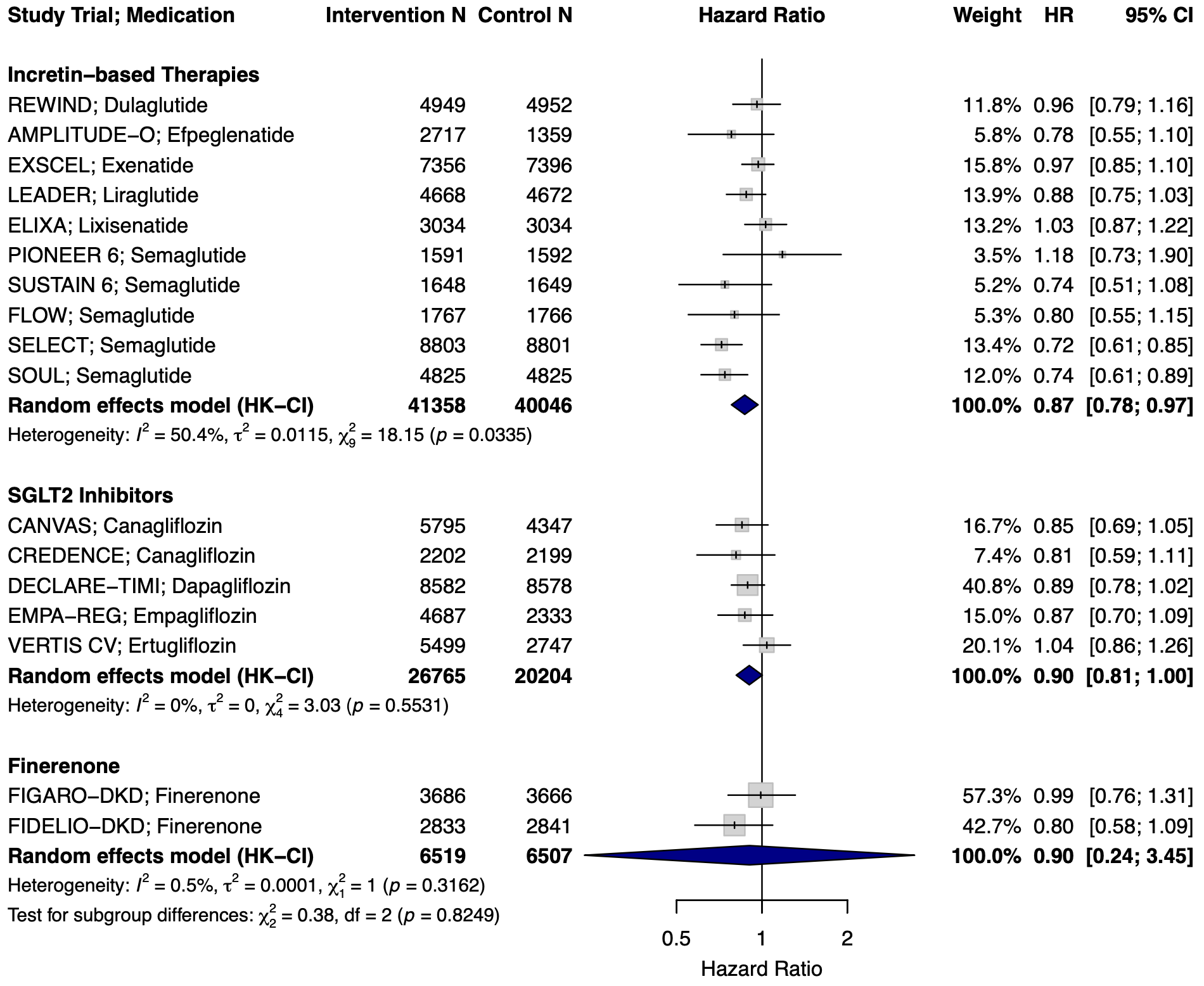
**

REML–Wald:

**
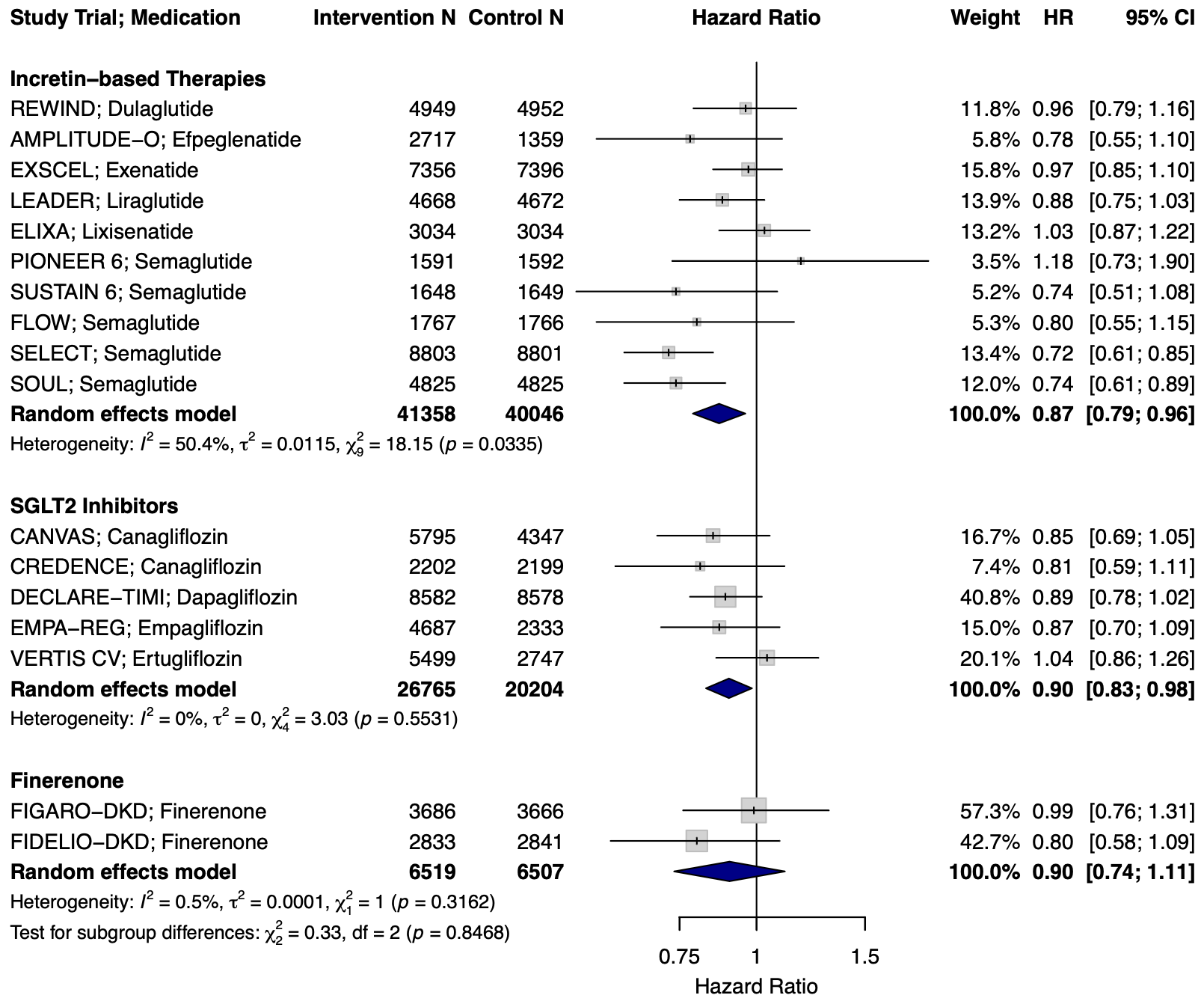
**

**Supplemental Figure S8 – Non-fatal Stroke (Overall)**

DerSimonian and Laird:


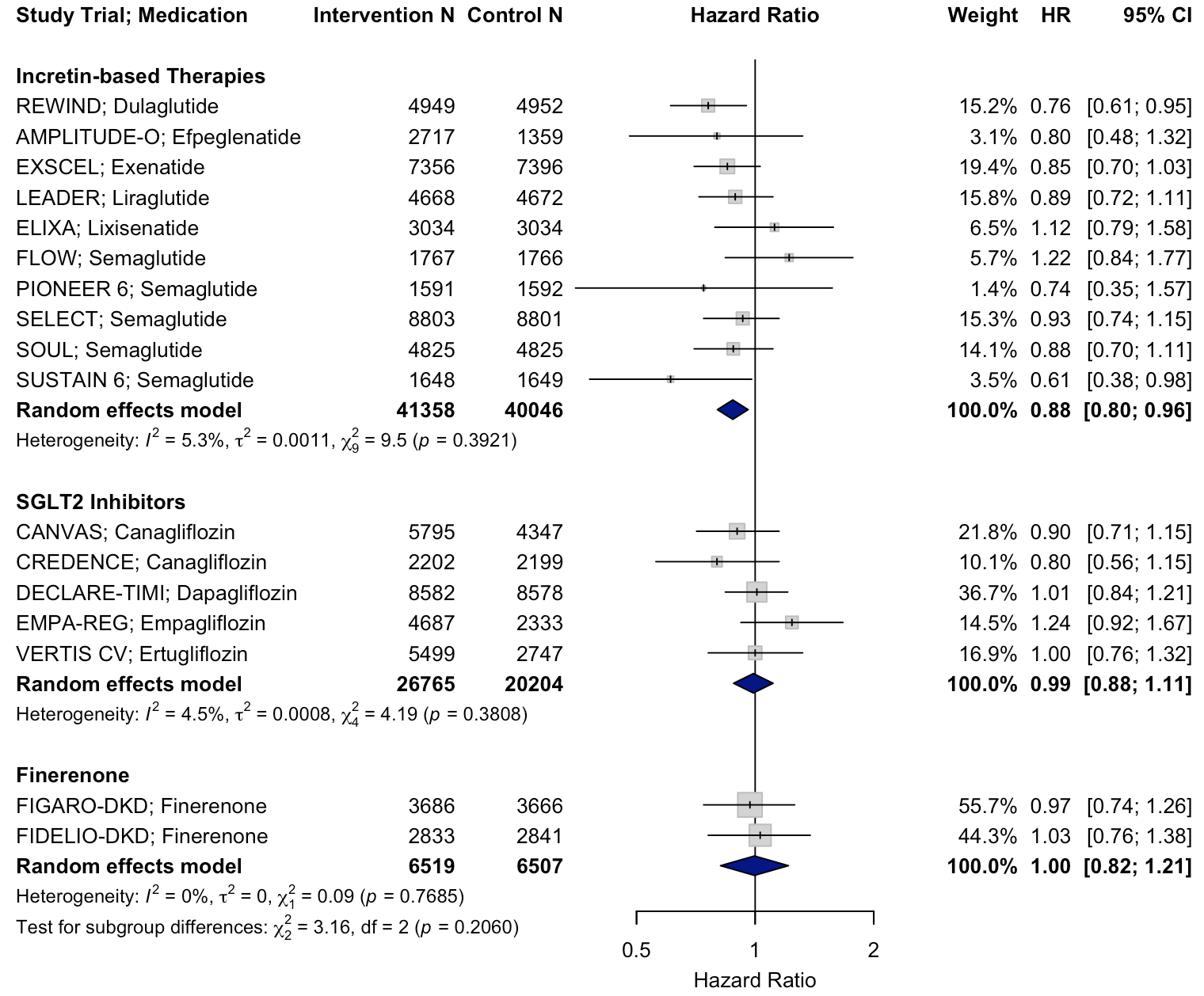


REML–modified HK:

**
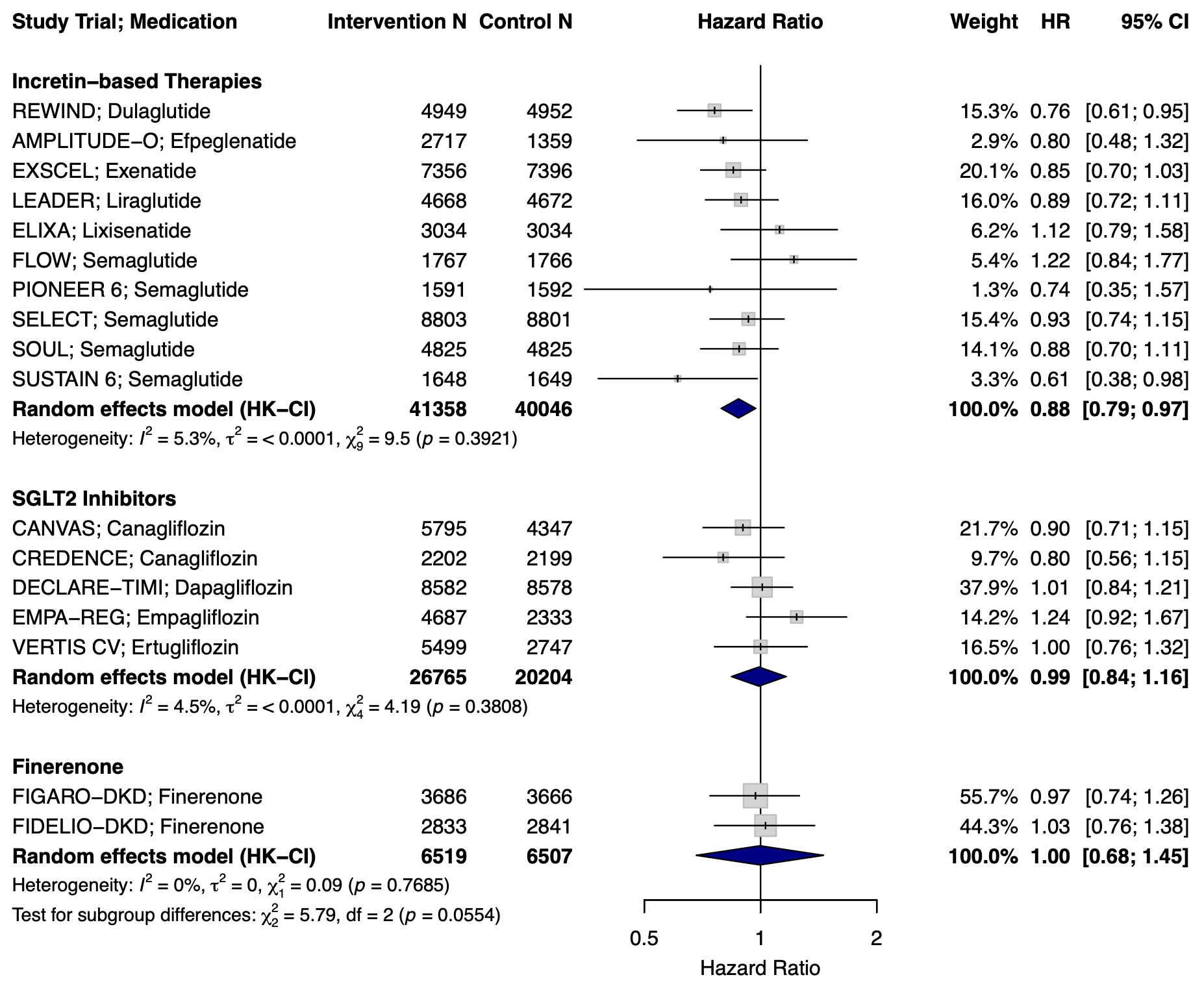
**

REML–Wald:

**
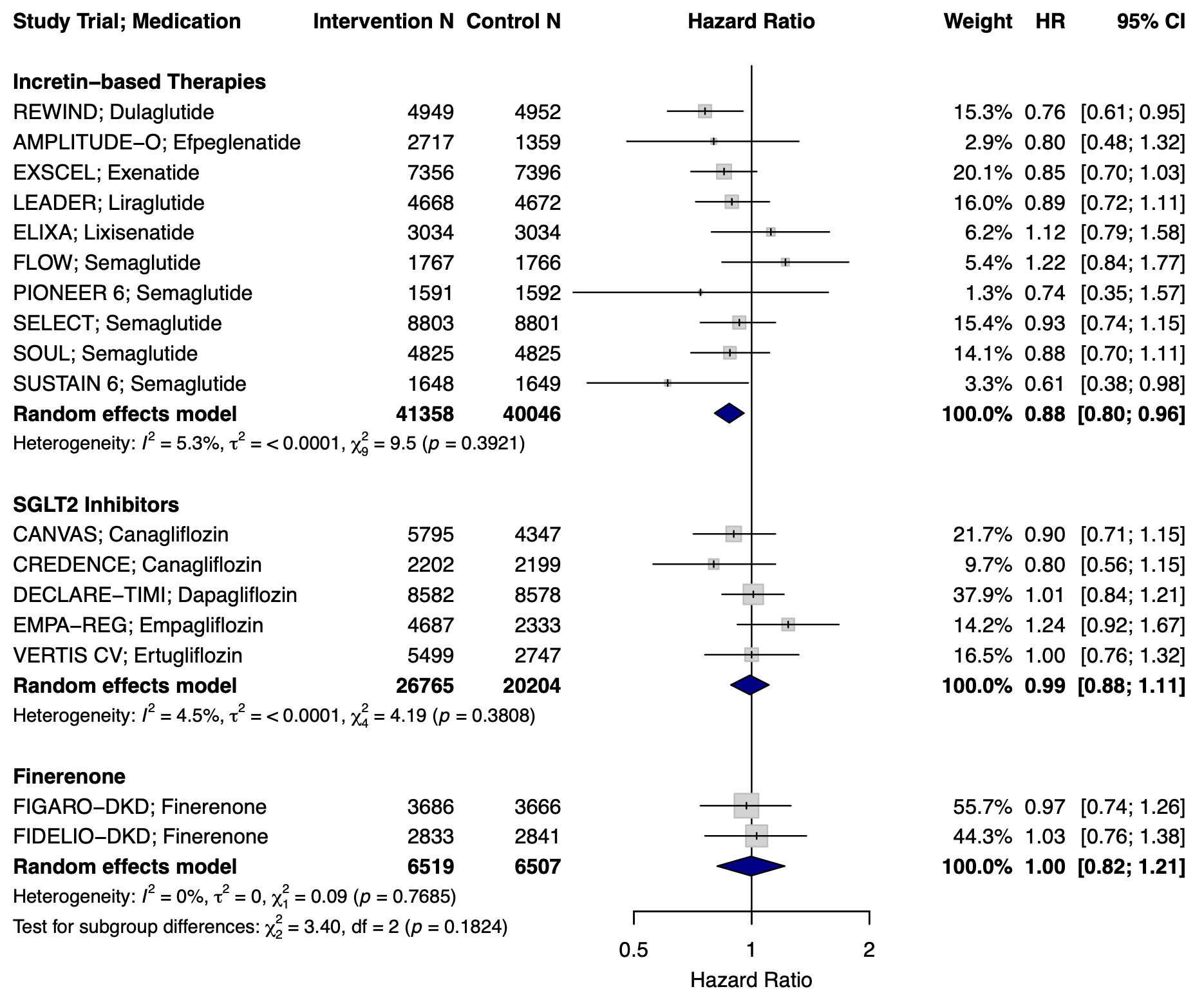
**

**Supplemental Figure S9 – MACE (Overall)**

DerSimonian and Laird:


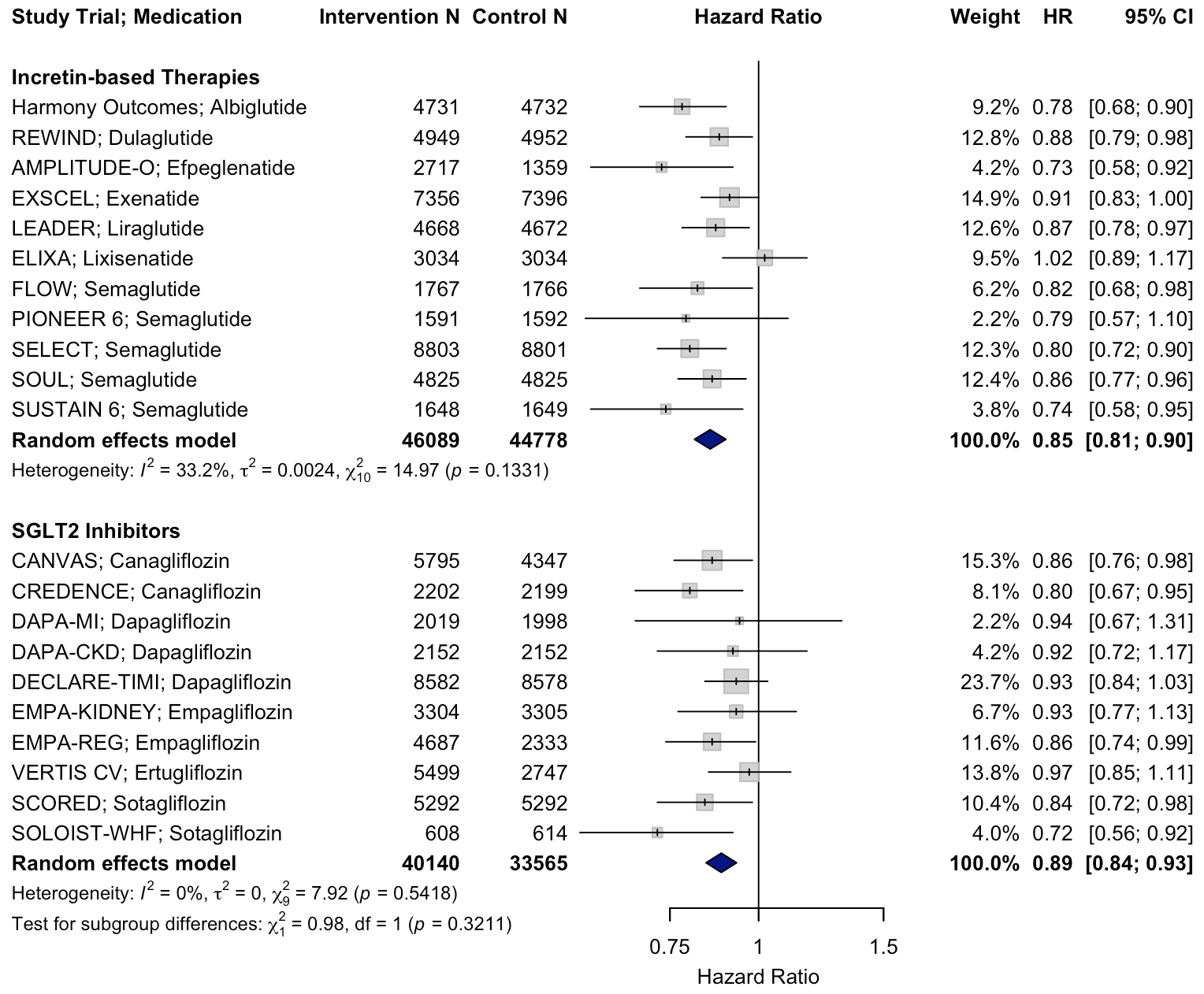


REML–modified HK:

**
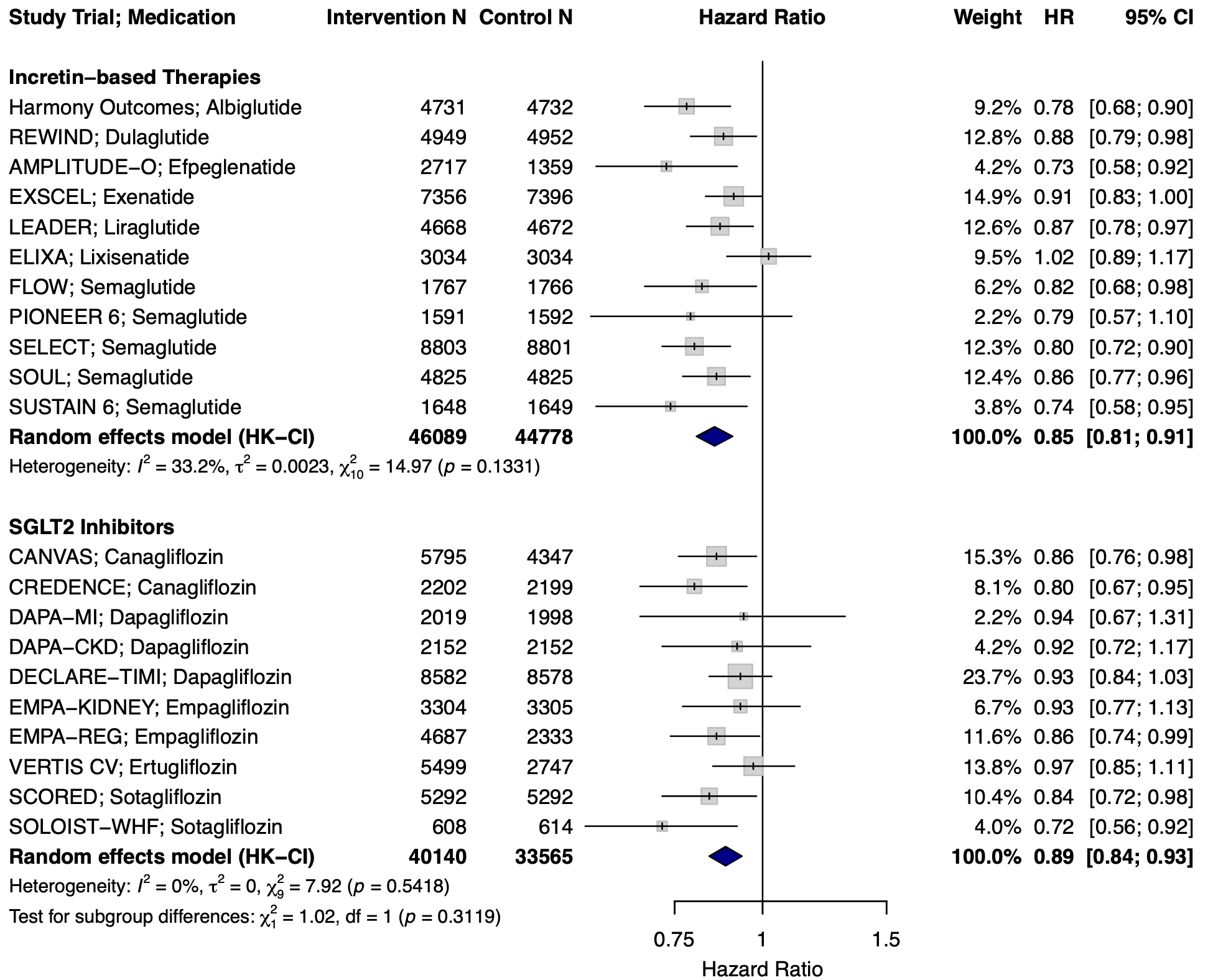
**

REML–Wald:

**
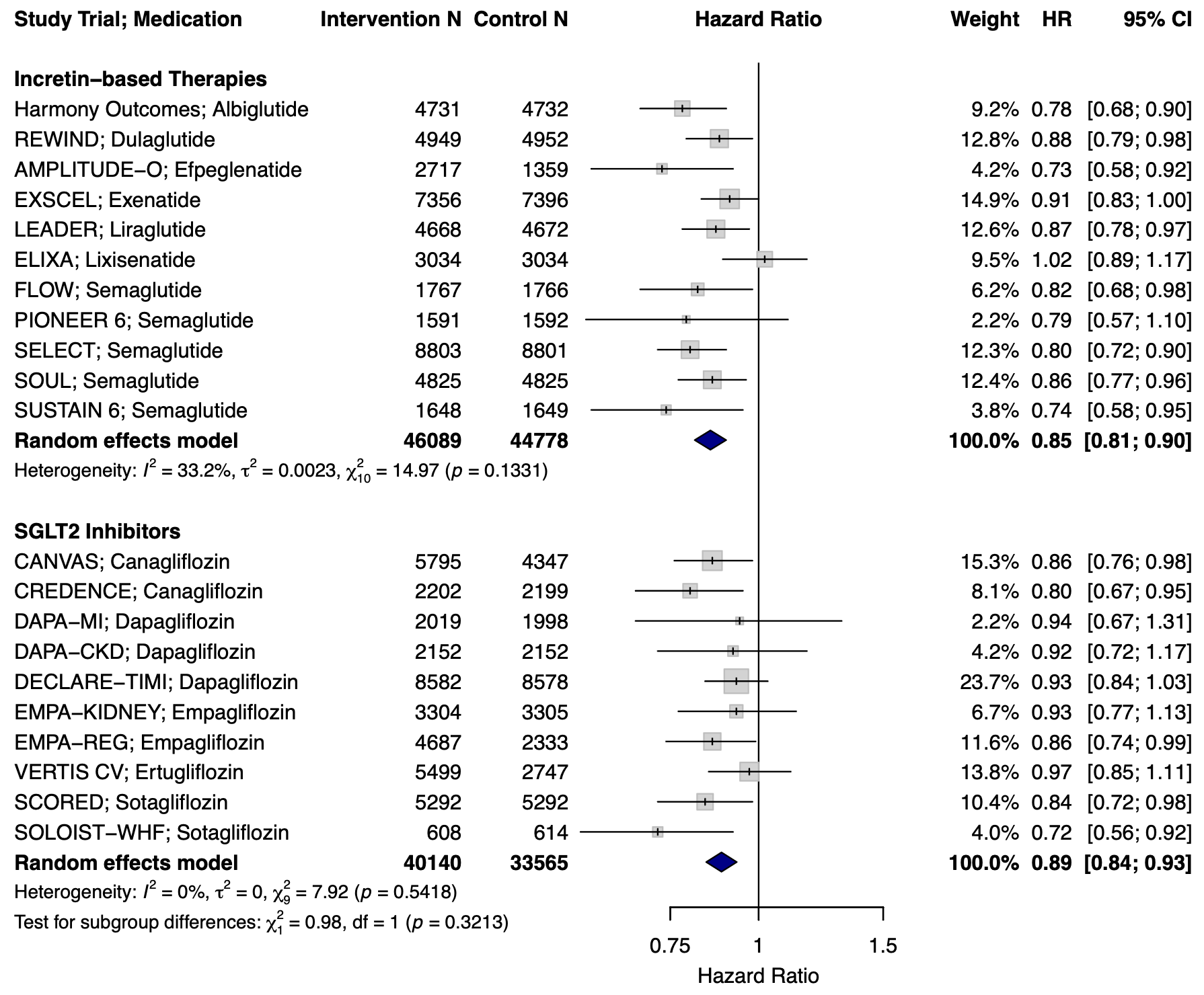
**

**Supplemental Figure S10 – Kidney Composite Outcome (Overall)**

DerSimonian and Laird:


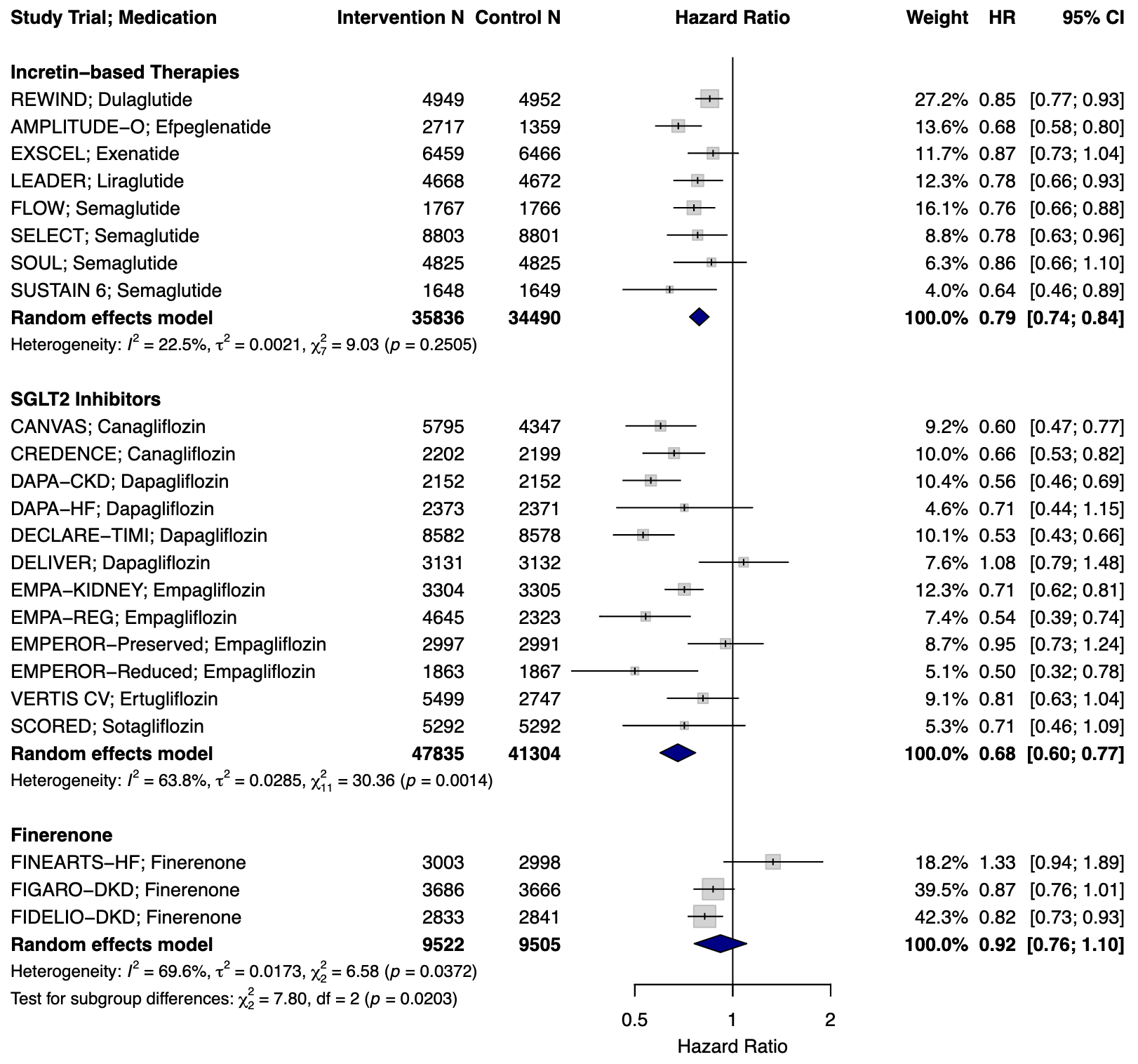


REML–modified HK:

**
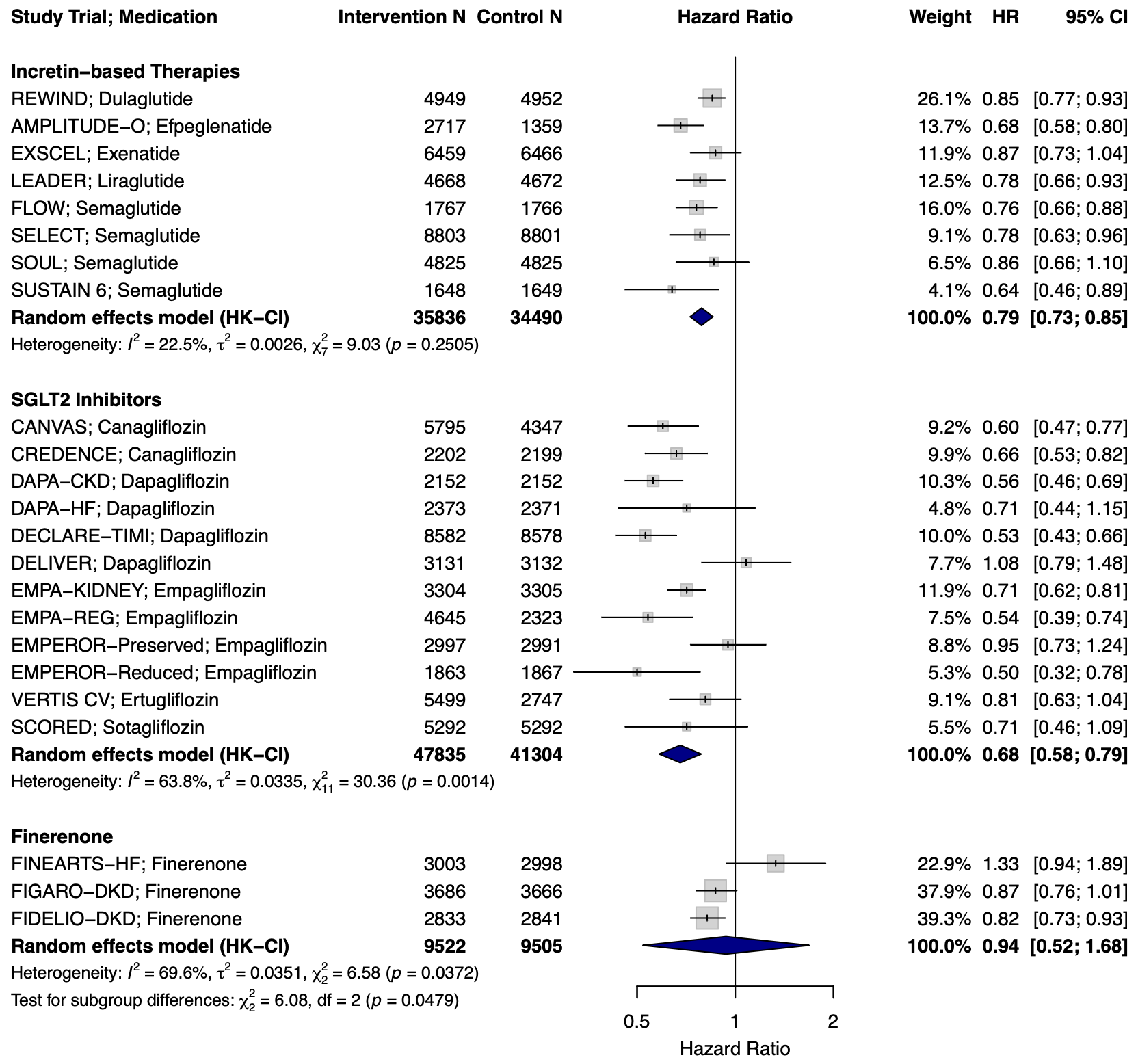
**

REML–Wald:

**
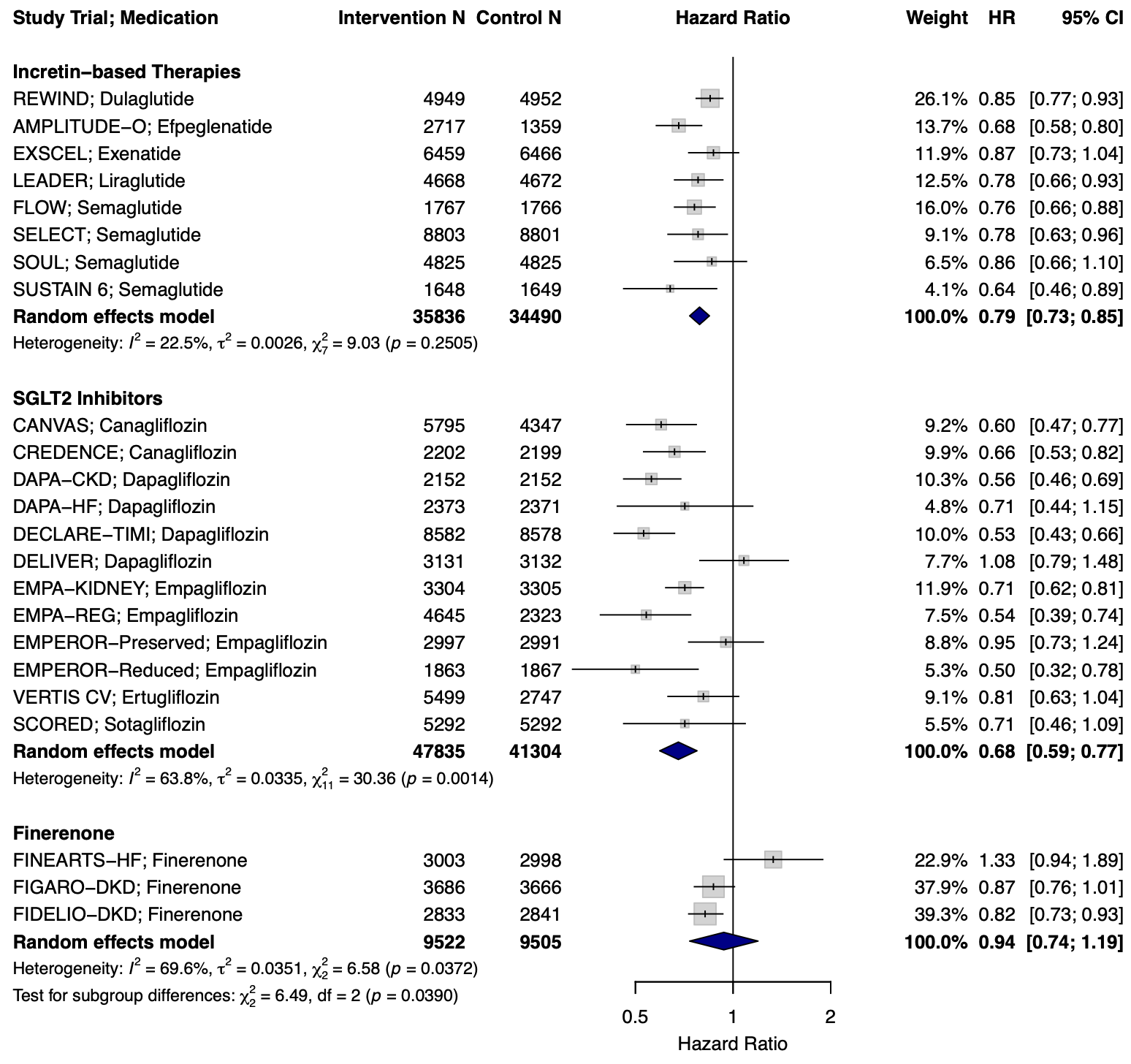
**

### **T2D with ASCVD/high CV Risk**

**Supplemental Figure S11 – Cardiovascular Mortality (T2DM with ASCVD / high CVD risk)**

DerSimonian and Laird:


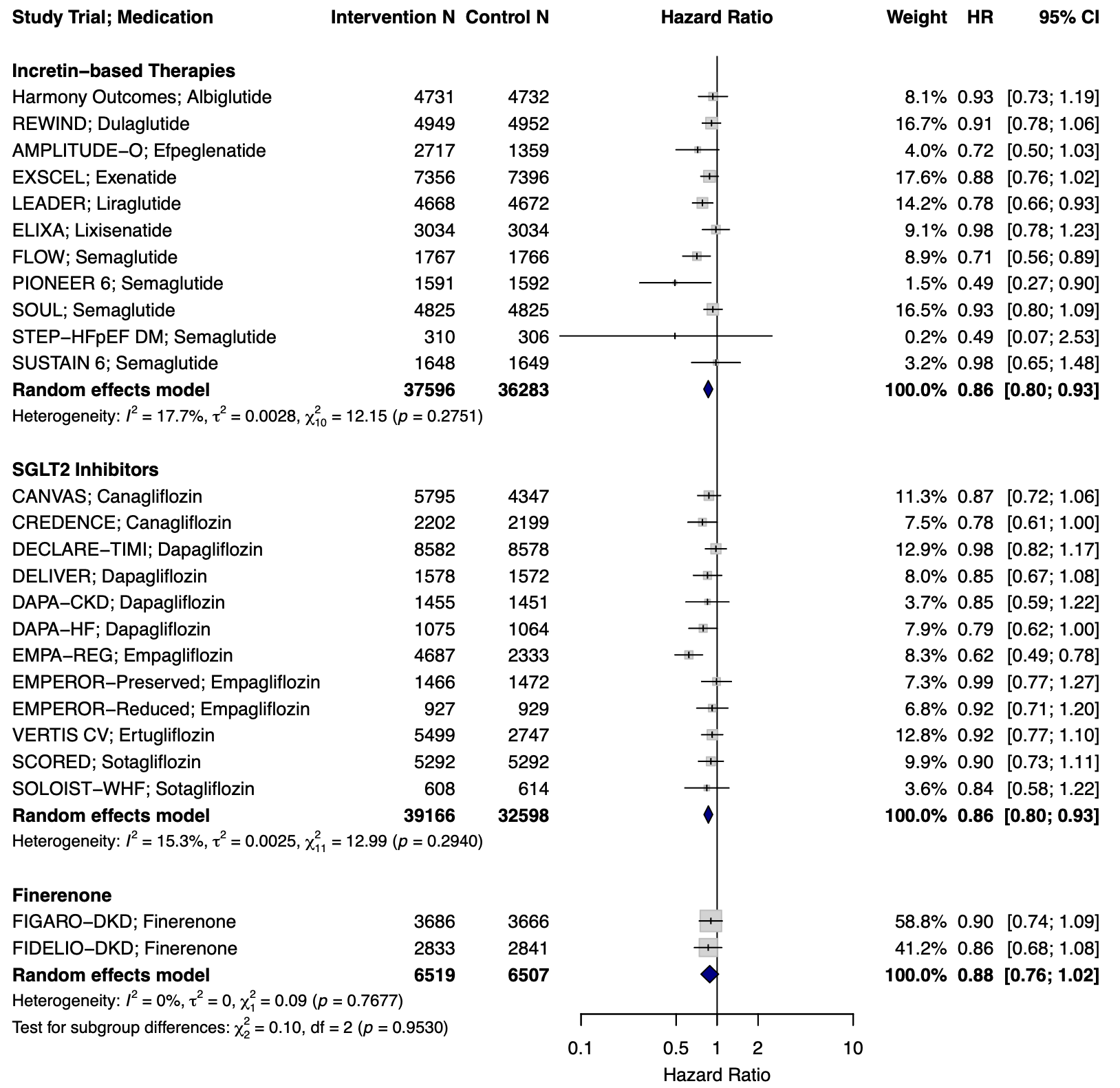


REML–modified HK:


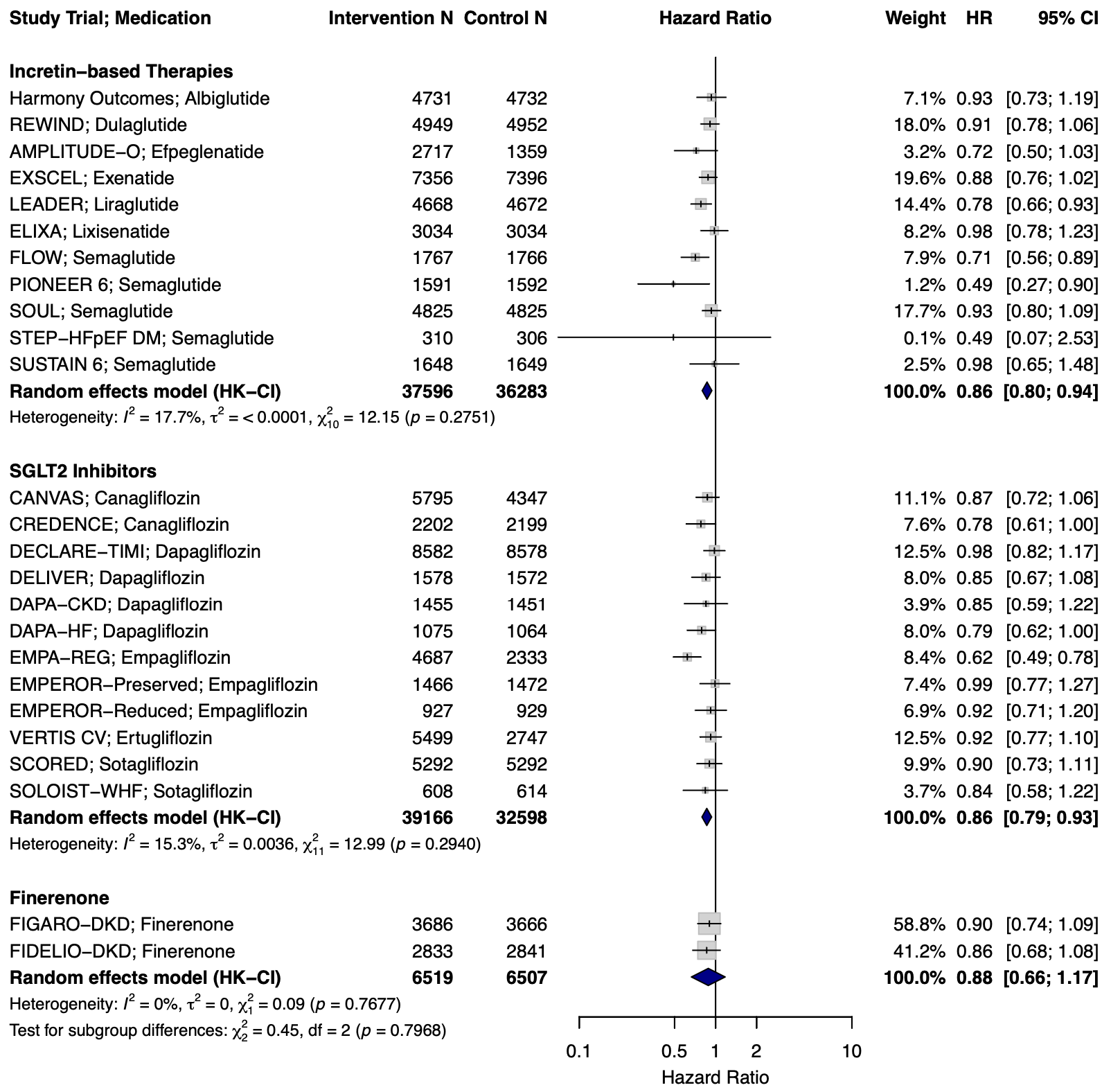


REML–Wald:


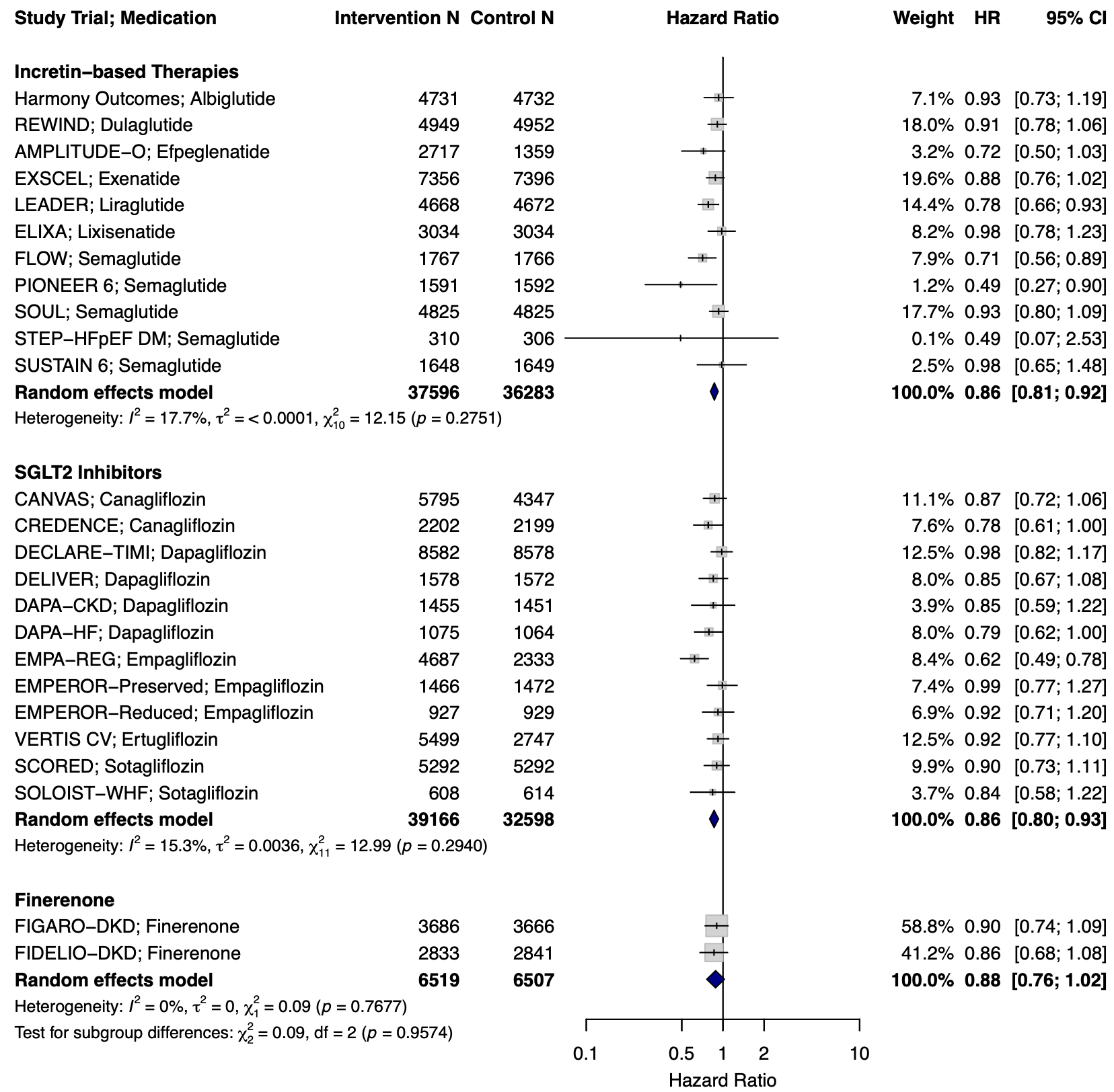


**Supplemental Figure S12 – All-Cause Mortality (T2DM with ASCVD / high CVD risk)**

DerSimonian and Laird:


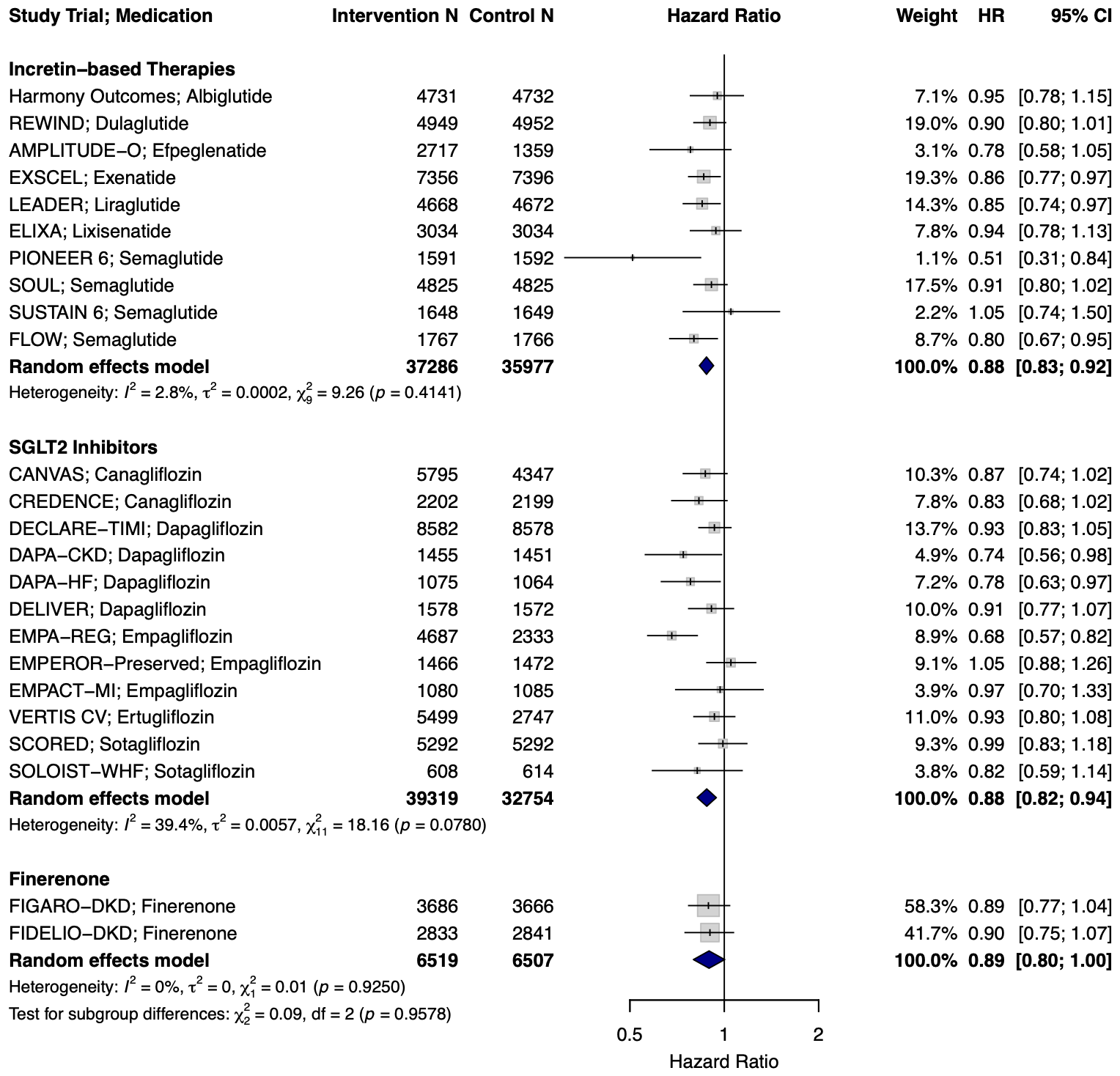


REML–modified HK:


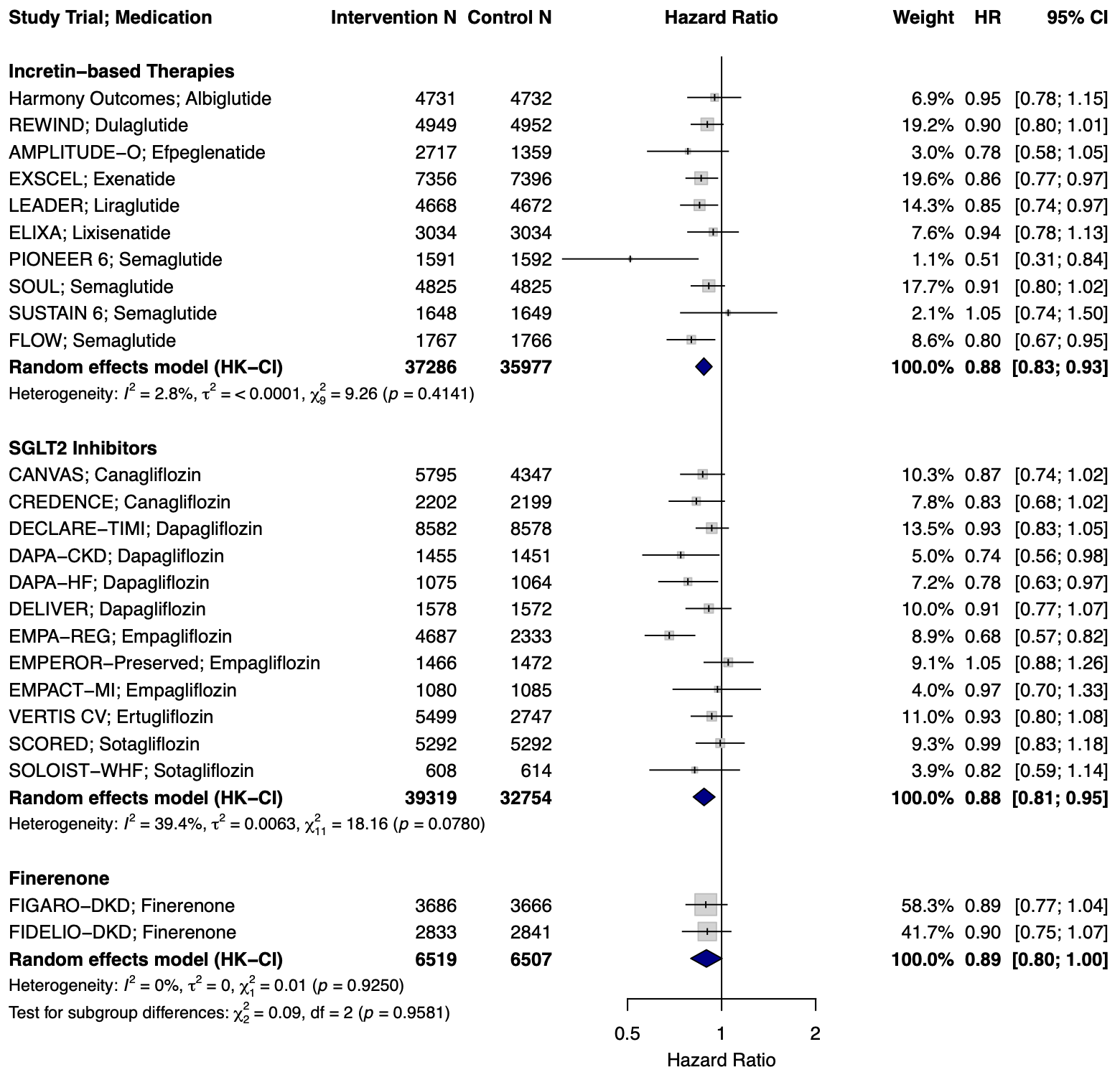


REML–Wald:

**
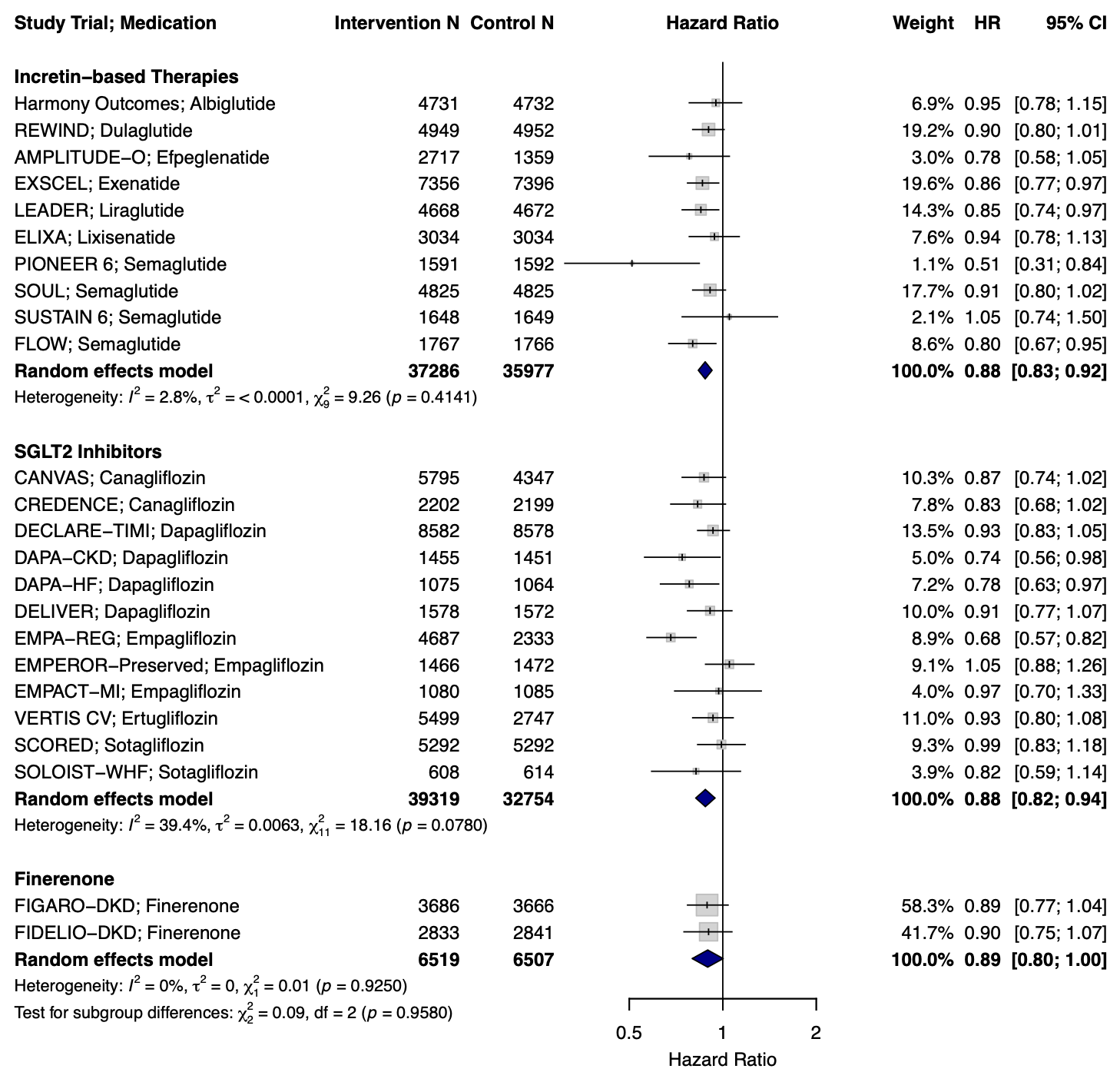
**

**Supplemental Figure S13 – CV Mortality or HF Hospitalization/HF Events (T2DM with ASCVD / high CVD risk)**

DerSimonian and Laird:


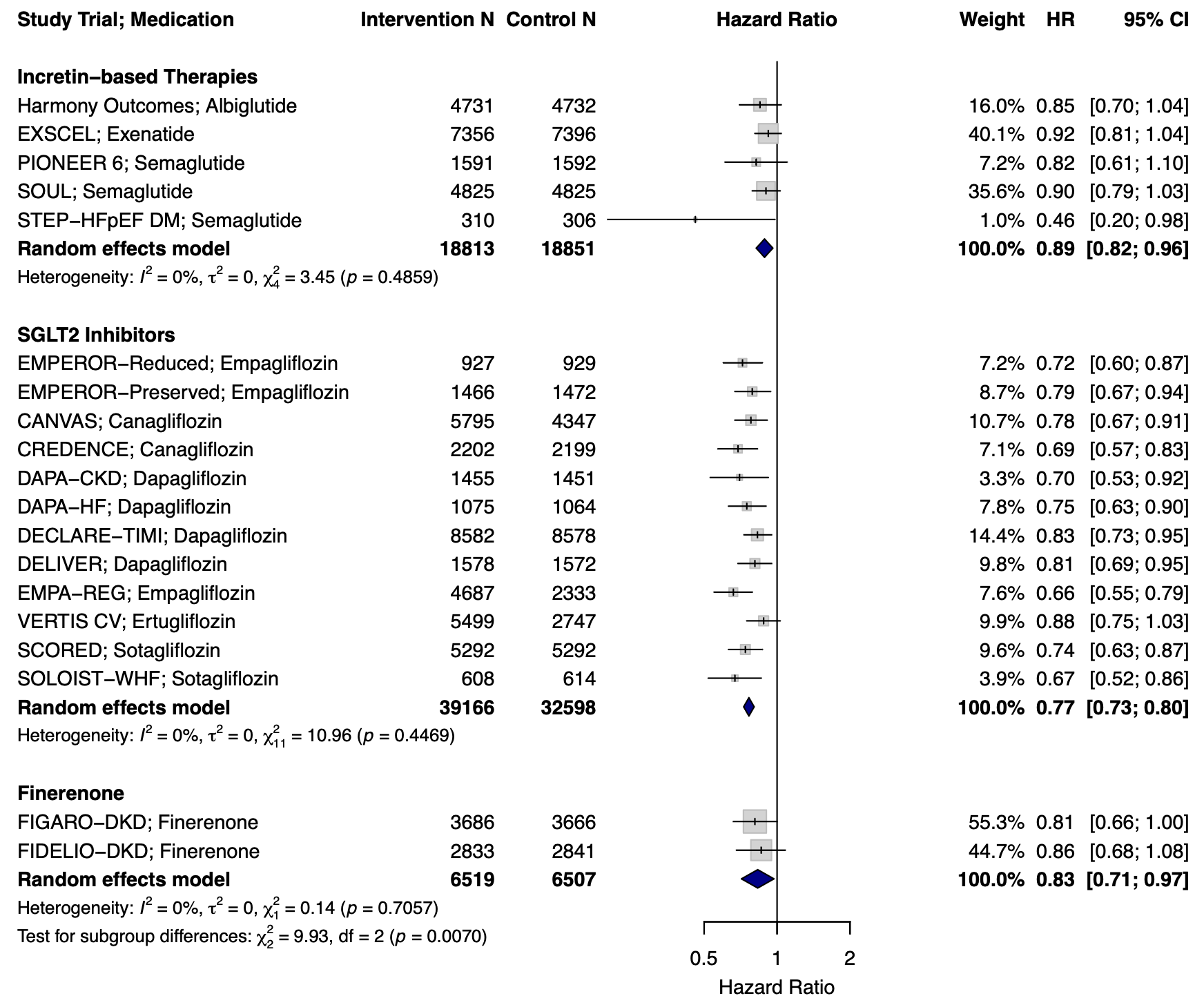


REML–modified HK:


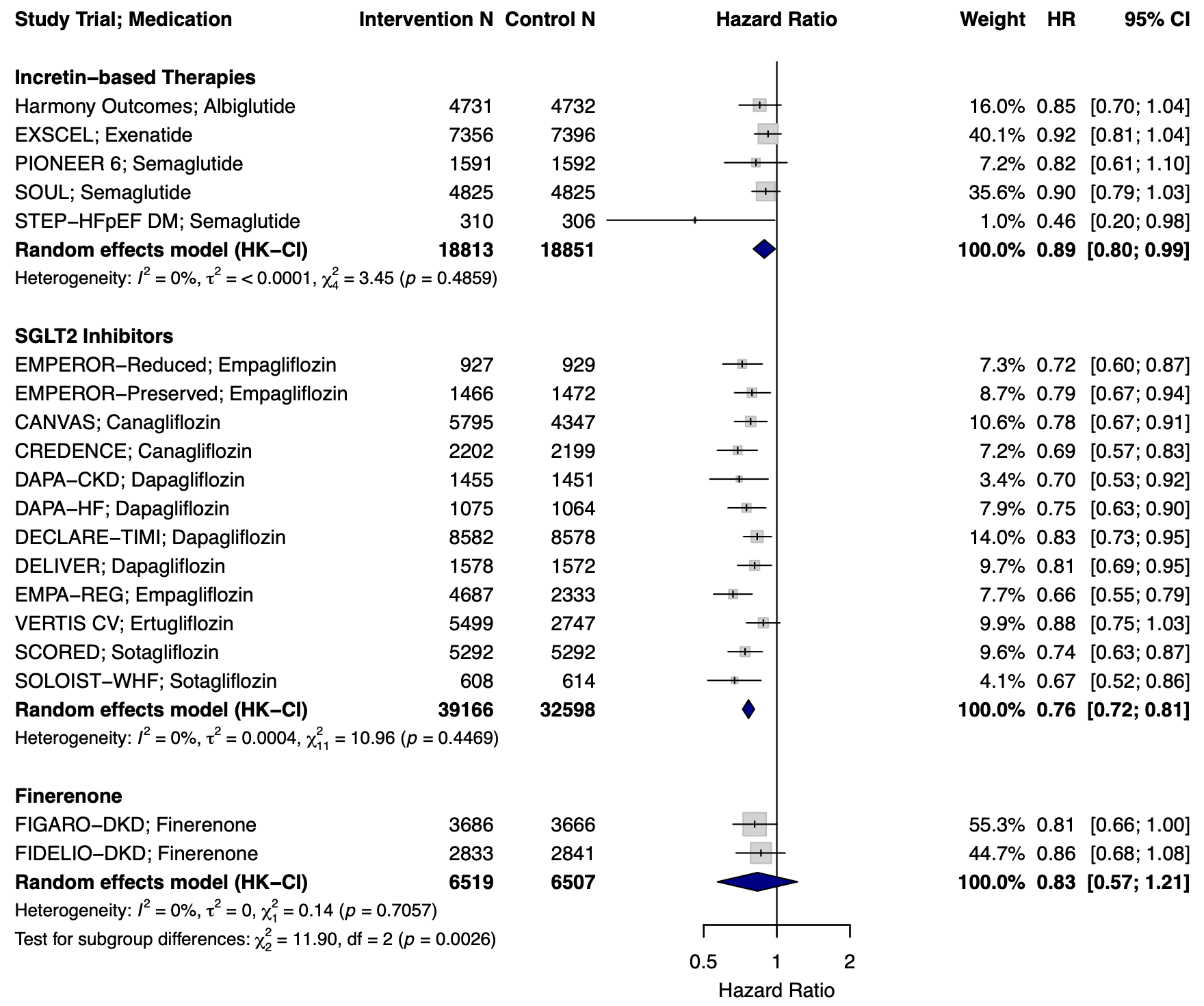


REML–Wald:

**
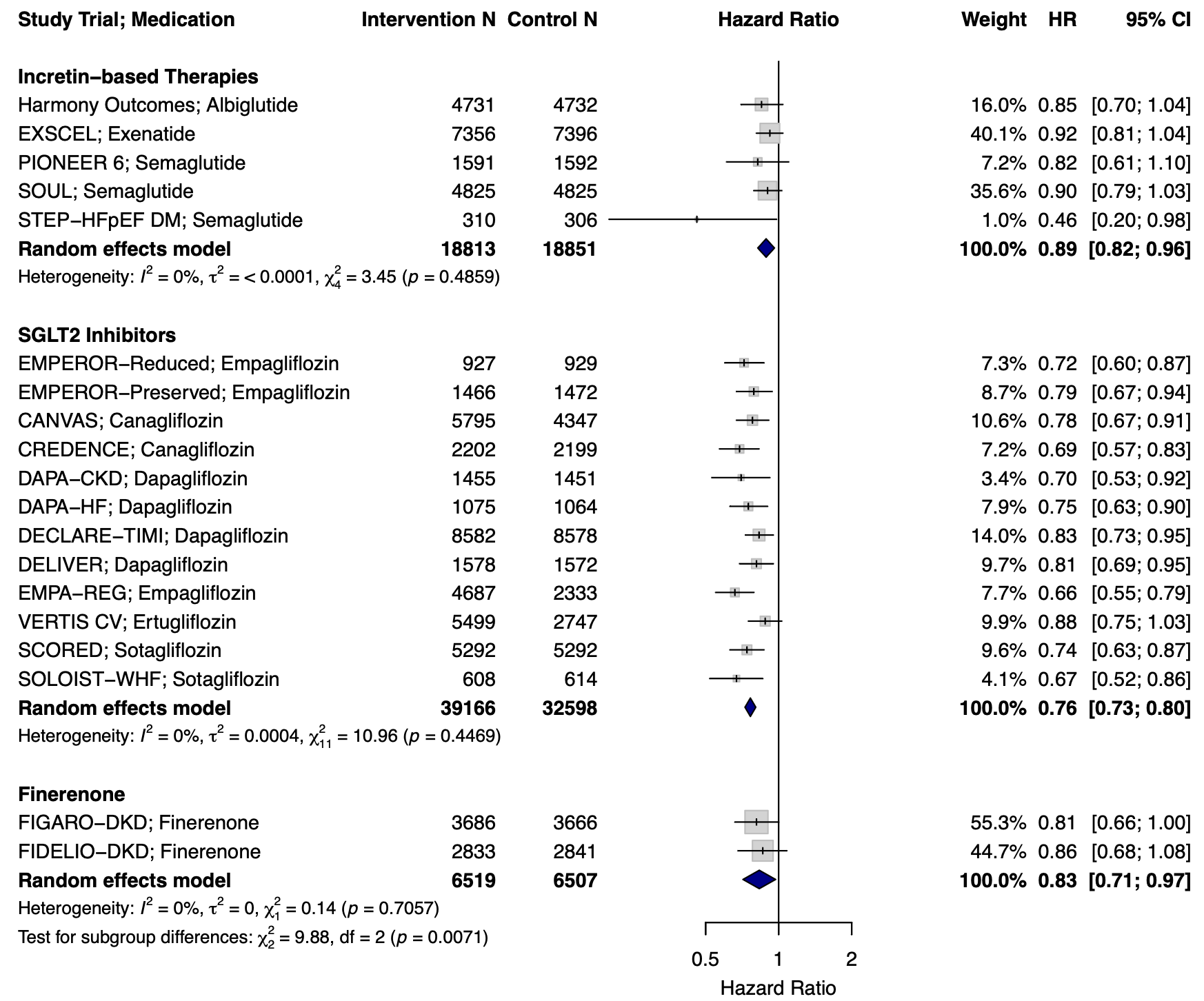
**

**Supplemental Figure S14 – CV Mortality or HF Hospitalization (T2DM with ASCVD / high CVD risk)**

DerSimonian and Laird:


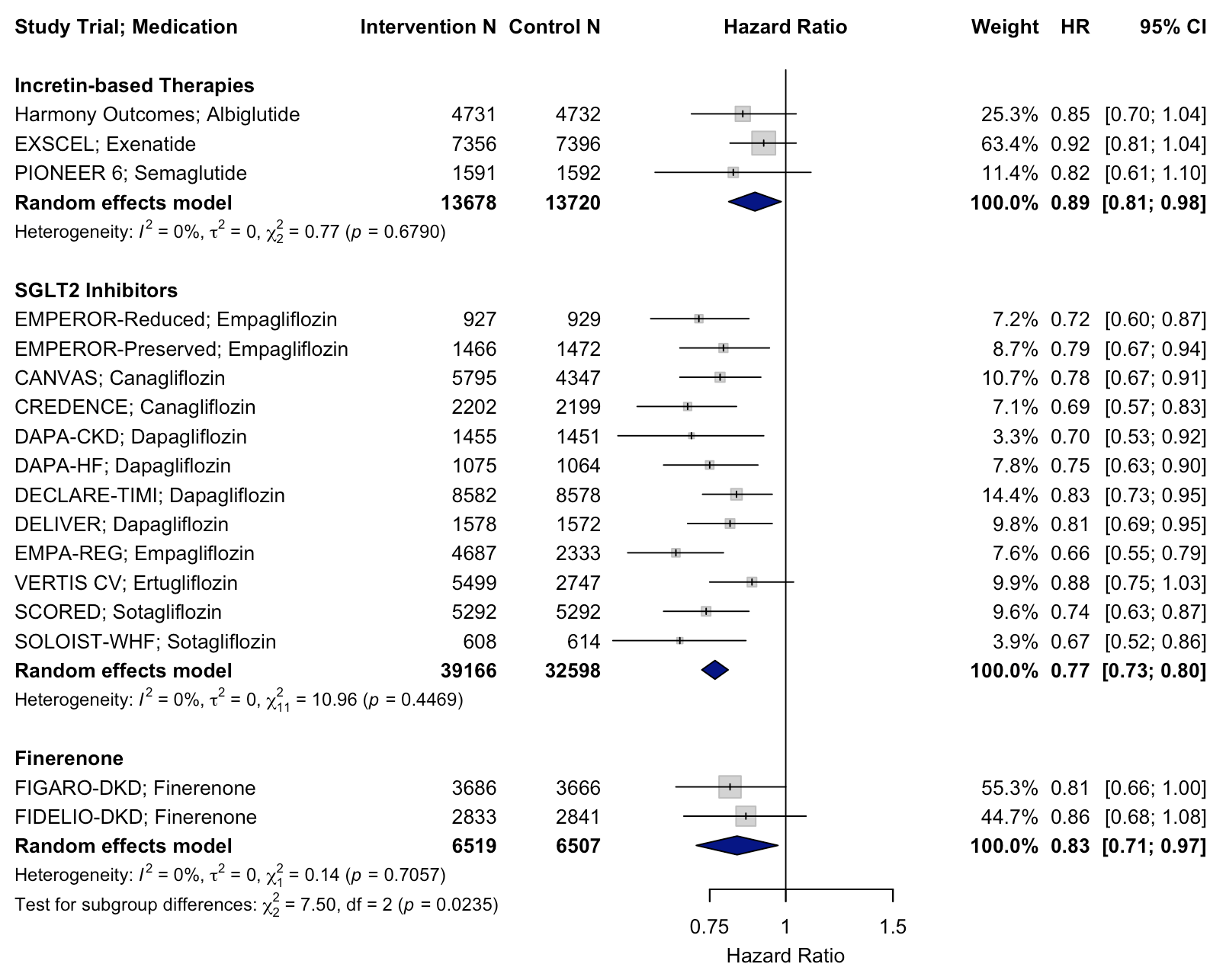


REML–modified HK:

**
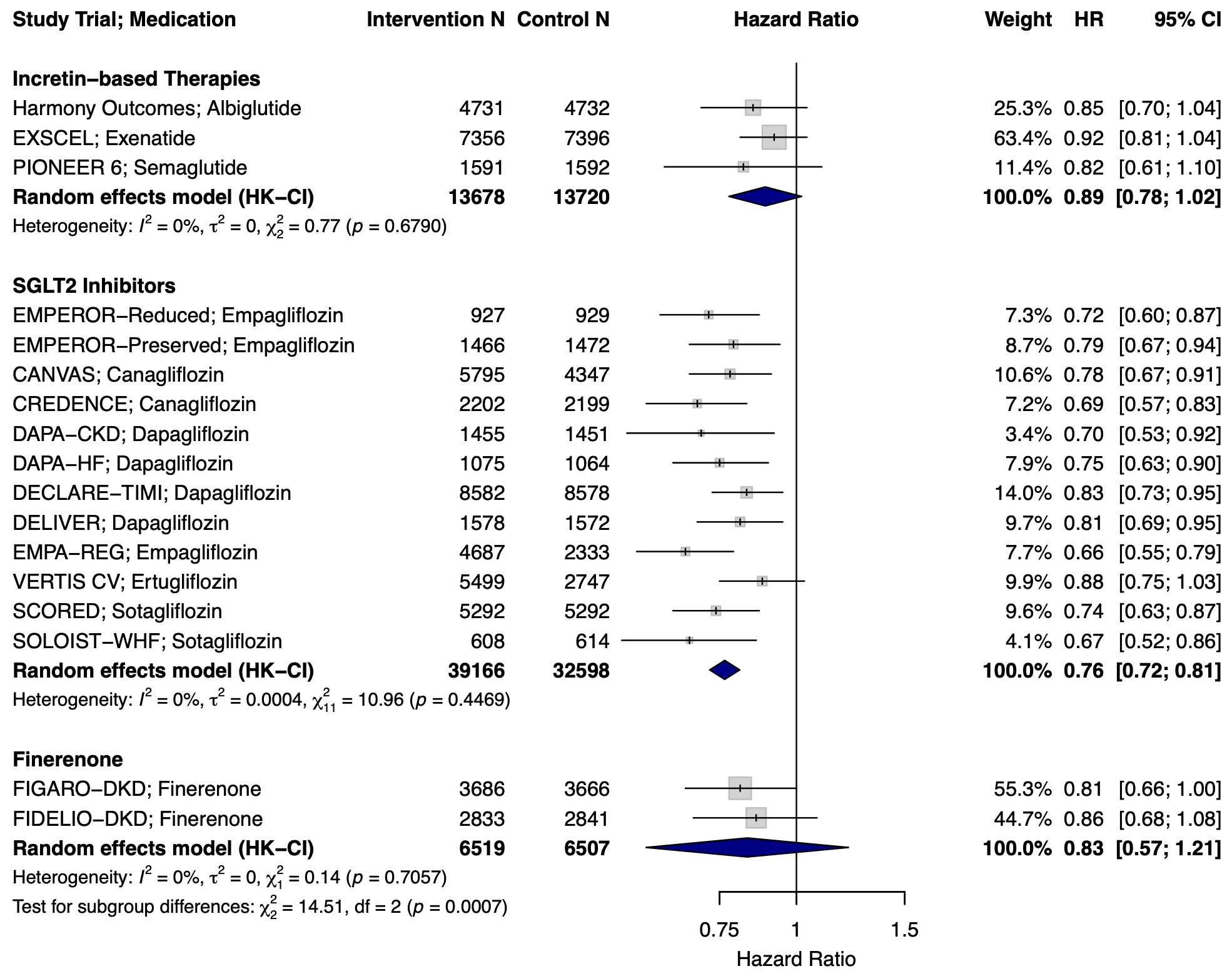
**

REML–Wald:

**
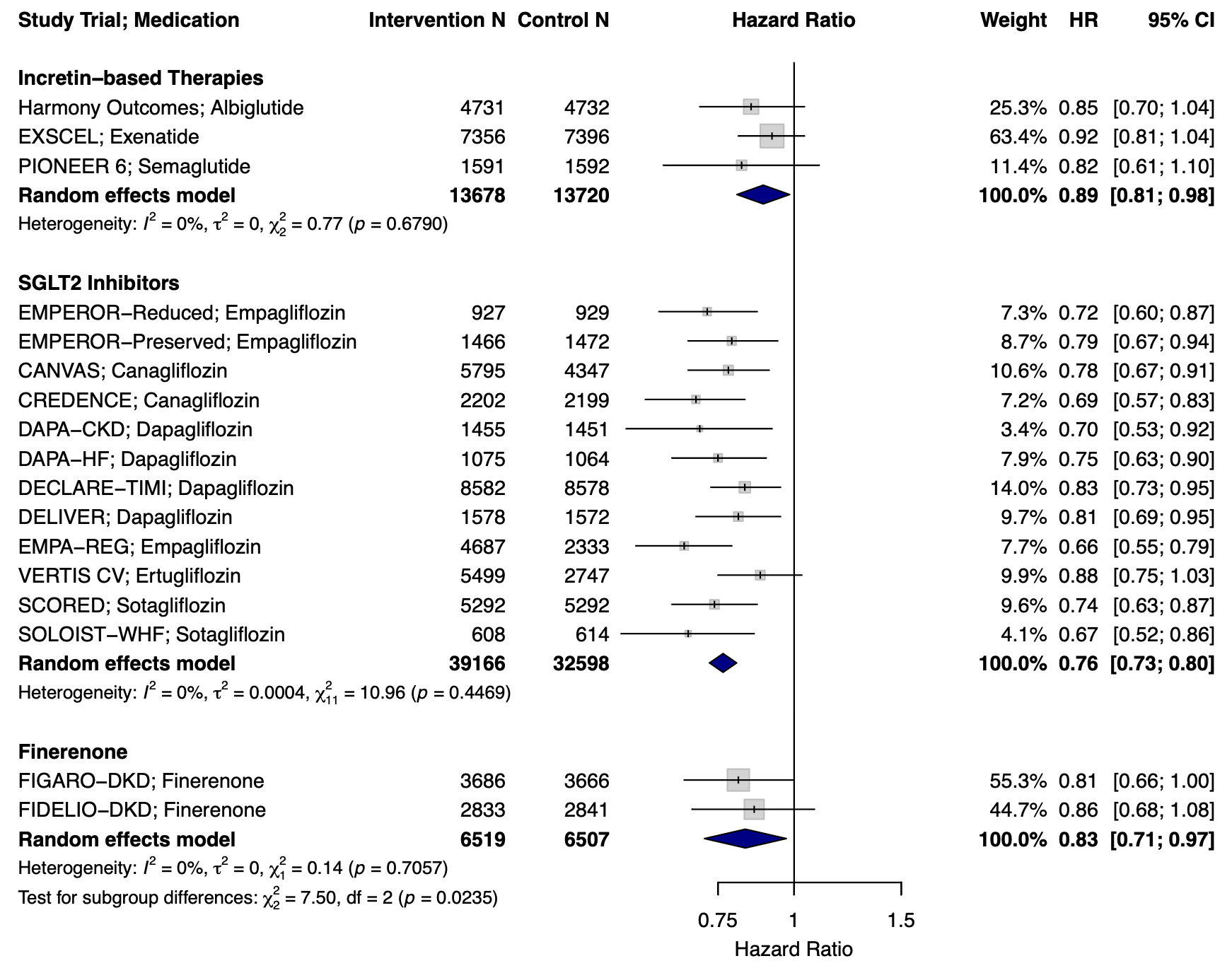
**

**Supplemental Figure S15 – HF Hospitalization/HF Events (T2DM with ASCVD / high CVD risk)**

DerSimonian and Laird:


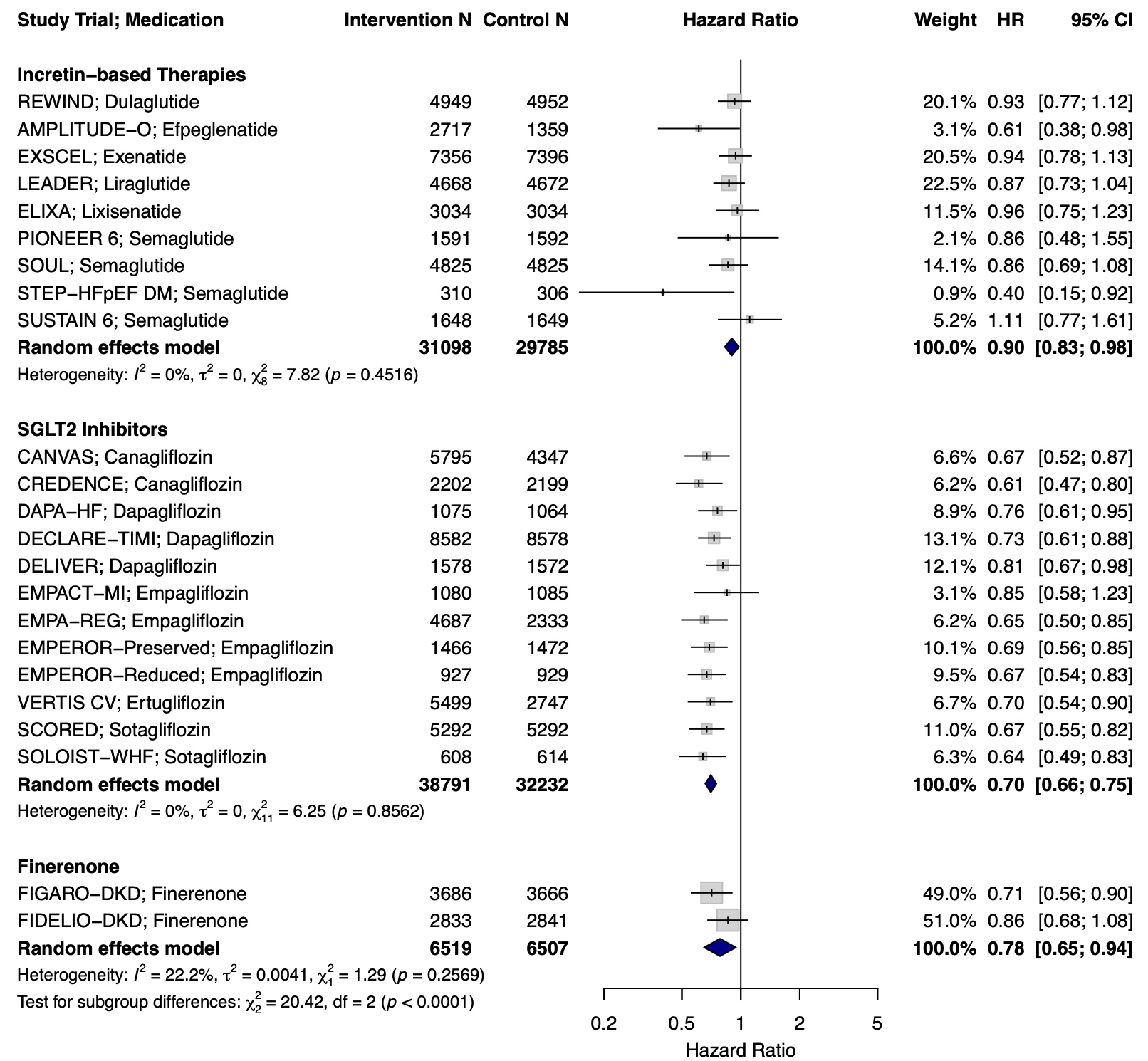


REML–modified HK:

**
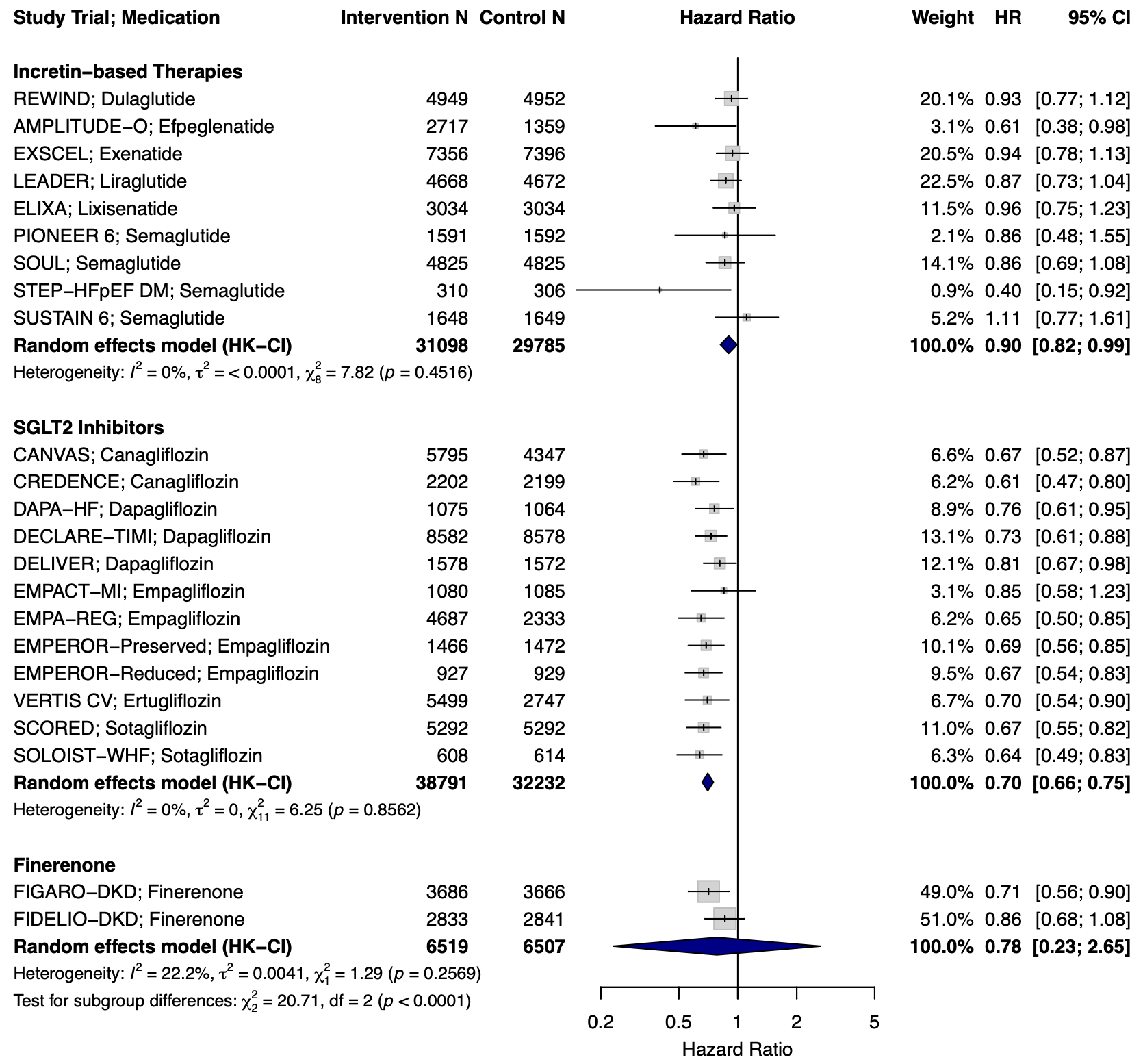
**

REML–Wald:

**
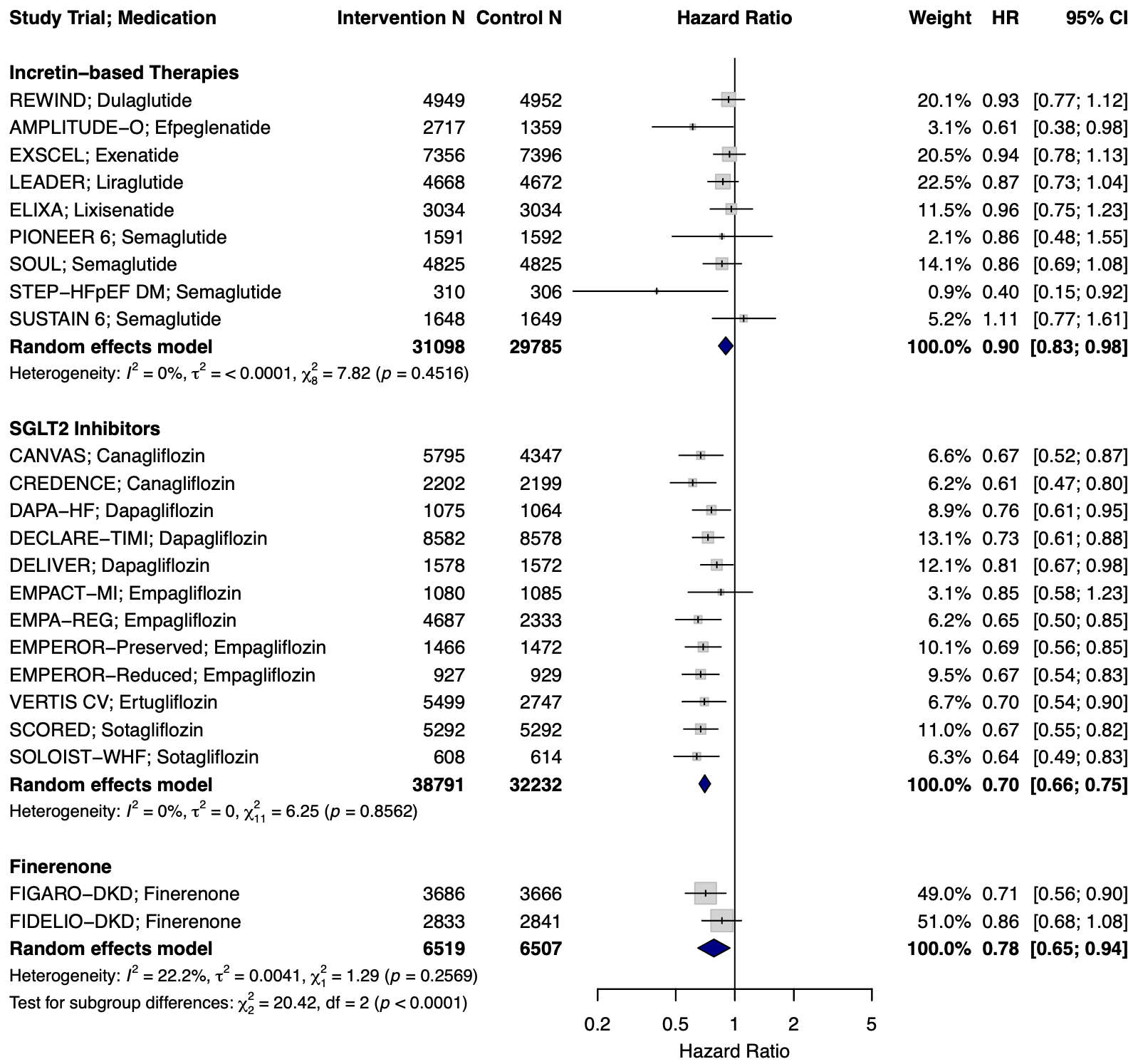
**

**Supplemental Figure S16 – HF Hospitalization (T2DM with ASCVD / high CVD risk)**

DerSimonian and Laird:


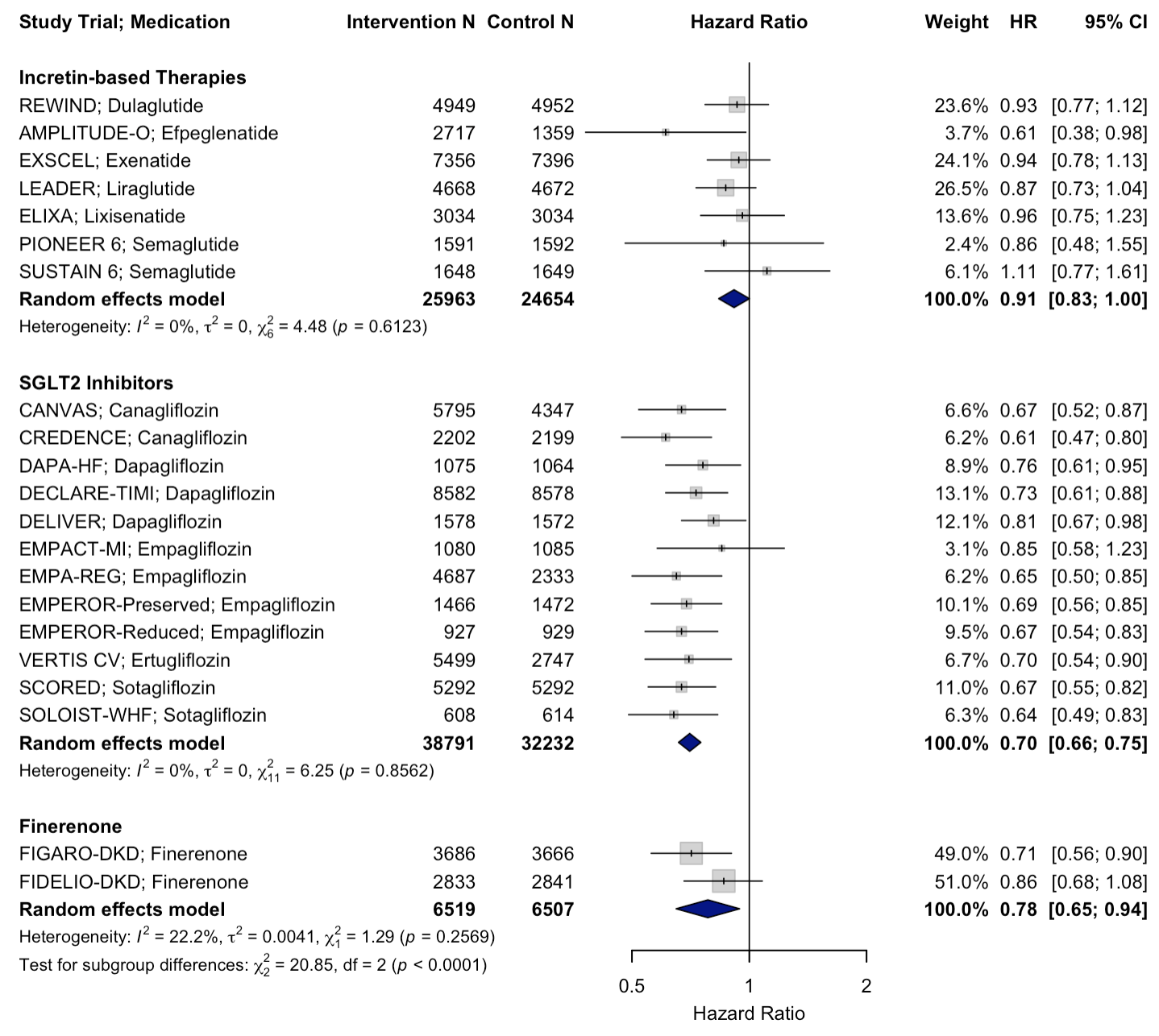


REML–modified HK:

**
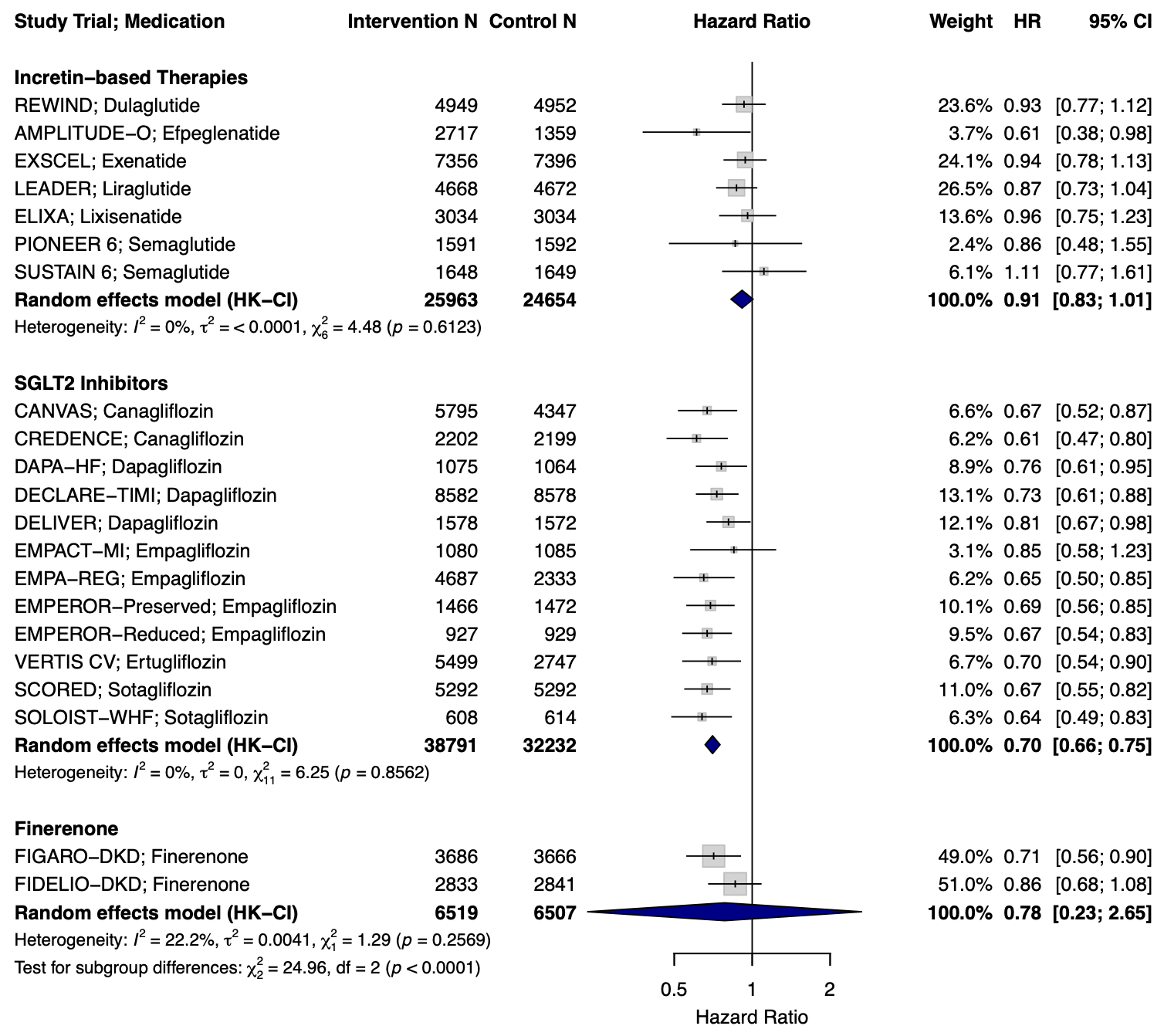
**

REML–Wald:

**
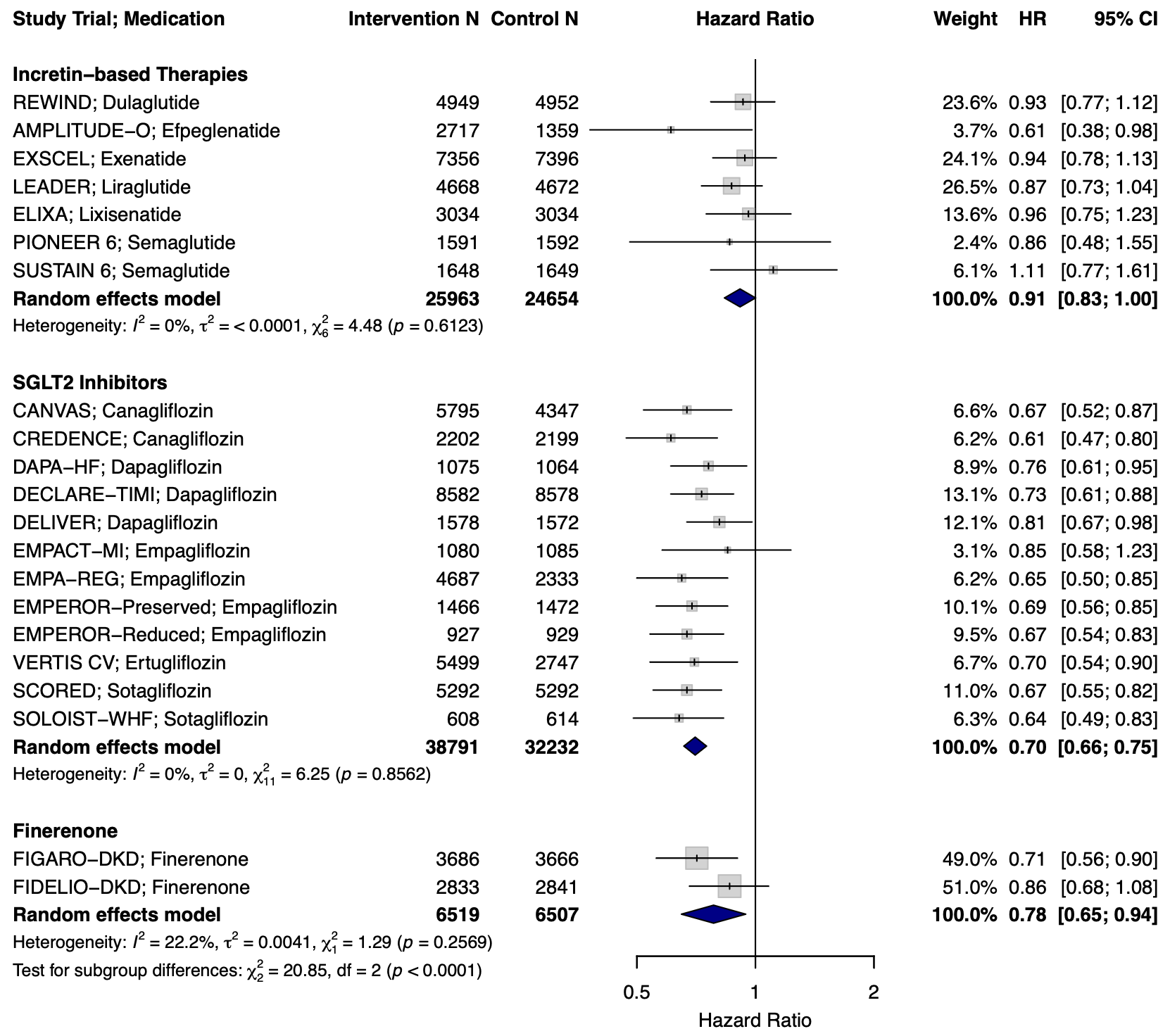
**

**Supplemental Figure S17 – Non-fatal Myocardial Infarction (T2DM with ASCVD / high CVD risk)**

DerSimonian and Laird:


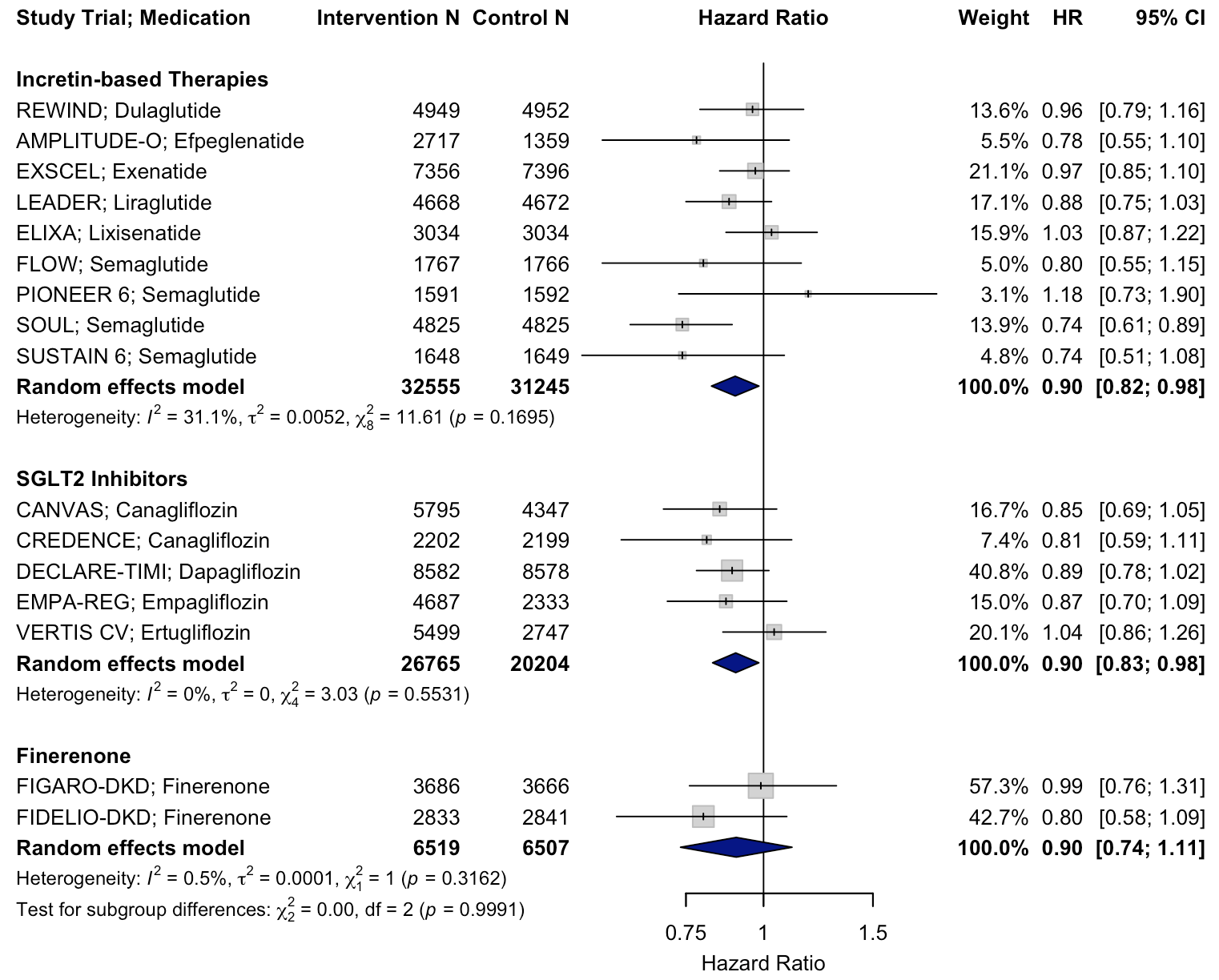


REML–modified HK:

**
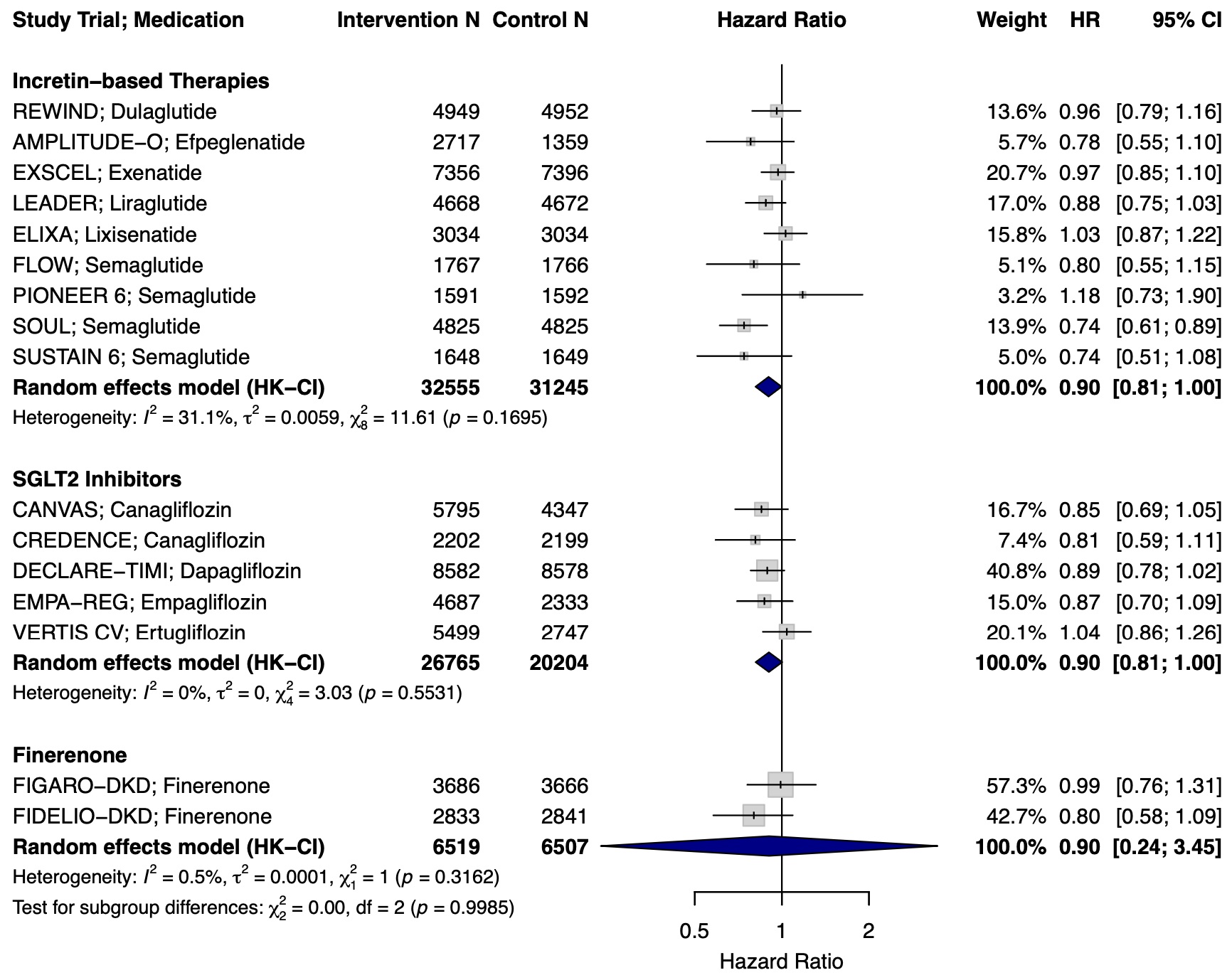
**

REML–Wald:

**
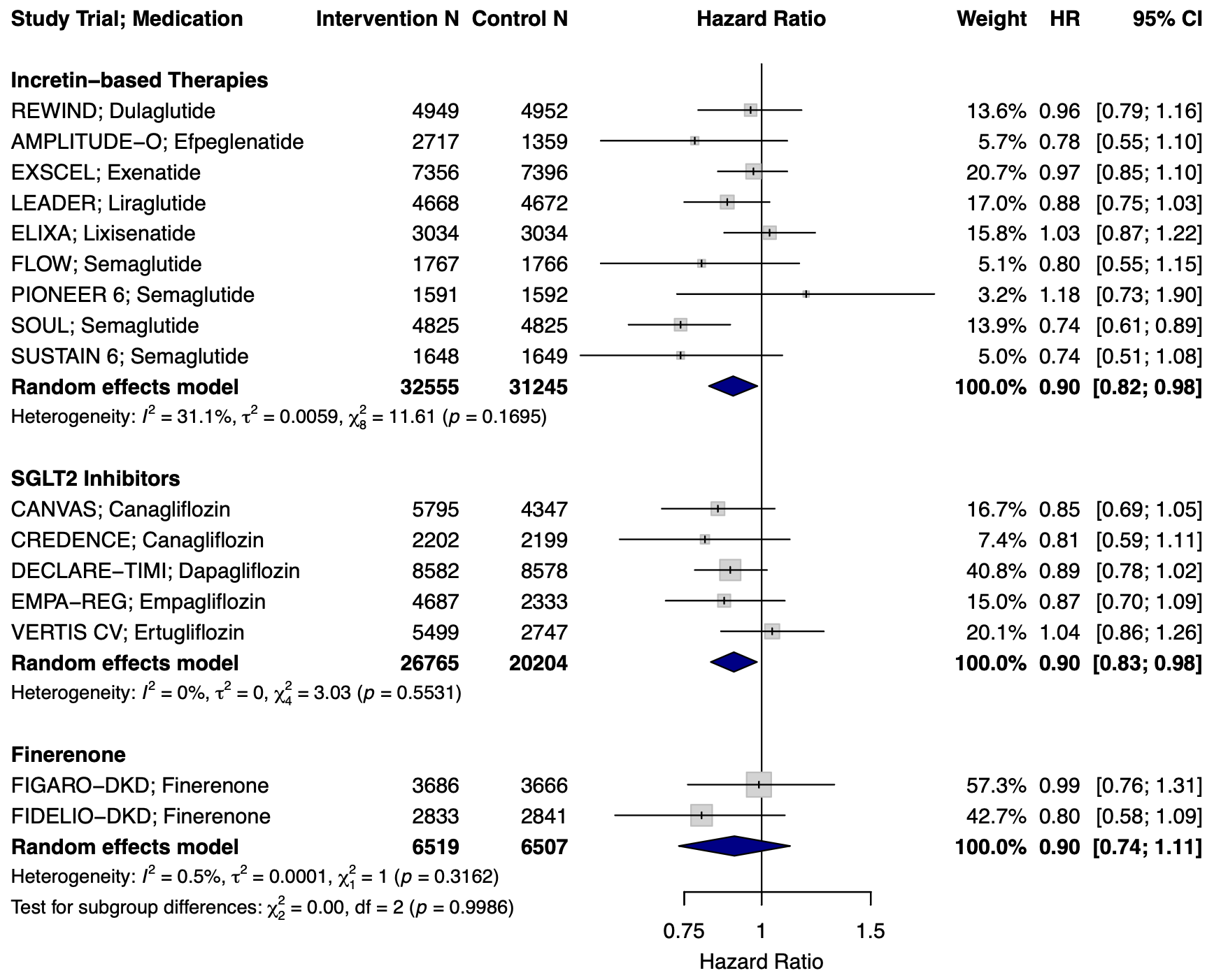
**

**Supplemental Figure S18 – Non-fatal Stroke (T2DM with ASCVD / high CVD risk)**

DerSimonian and Laird:


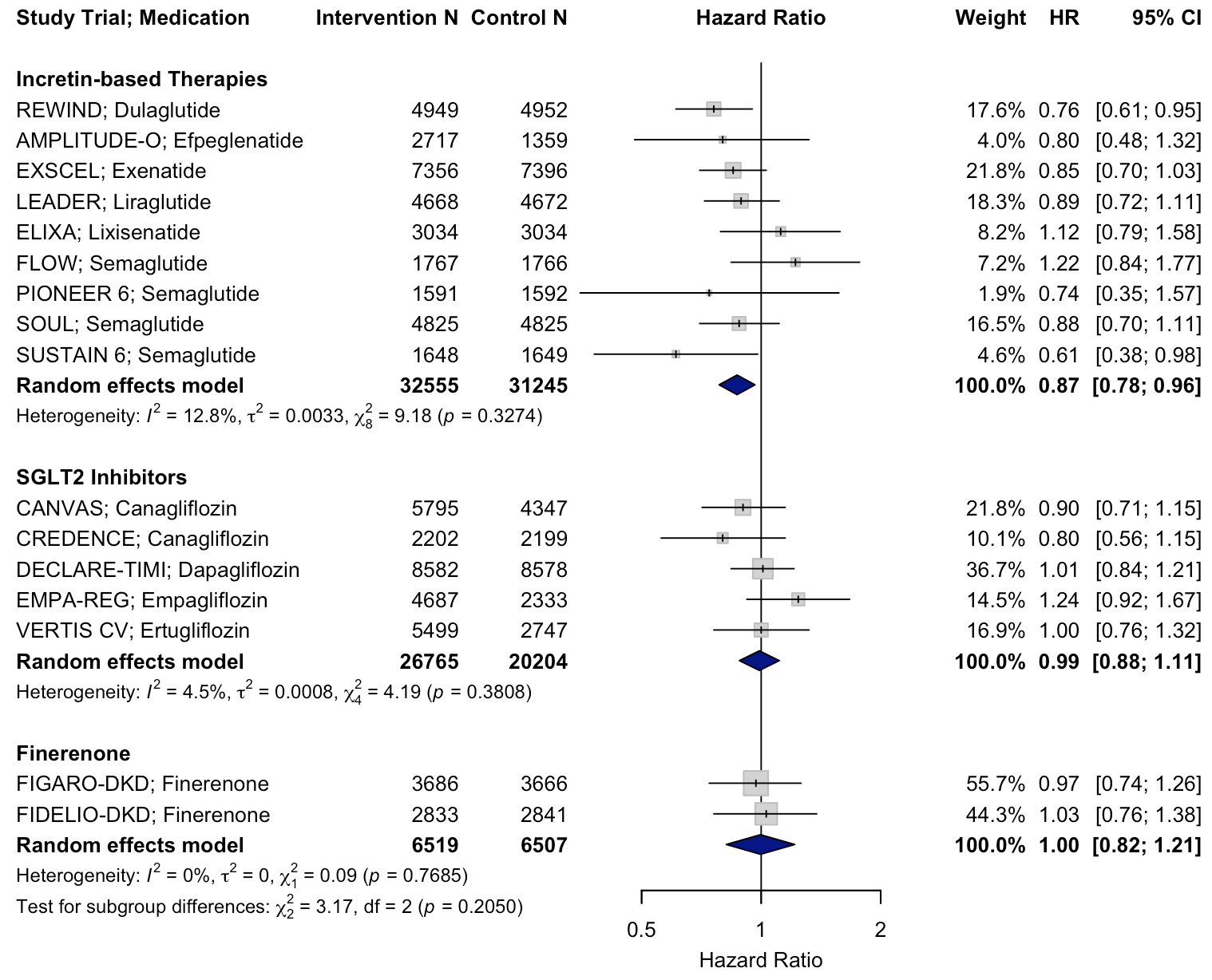


REML–modified HK:

**
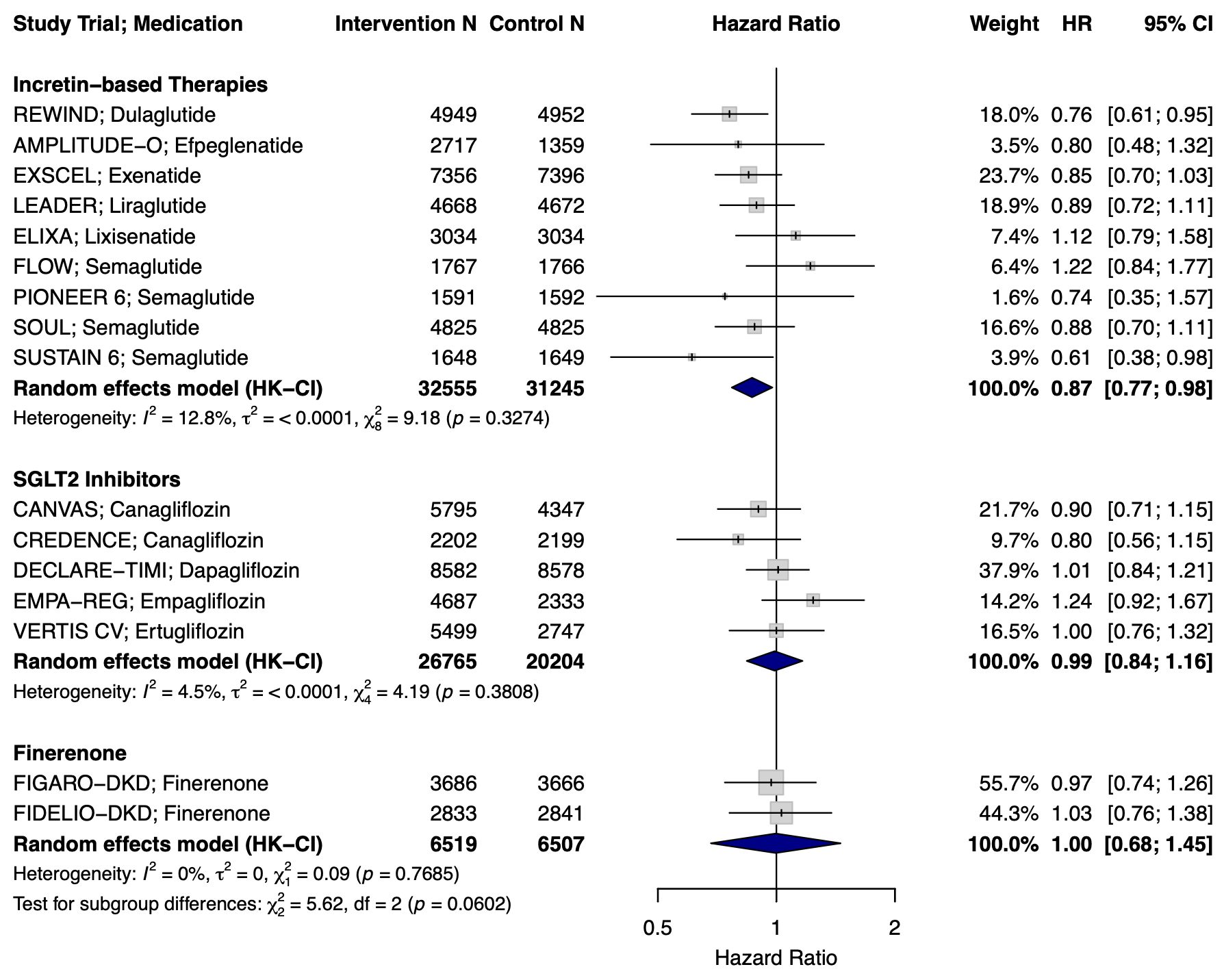
**

REML–Wald:

**
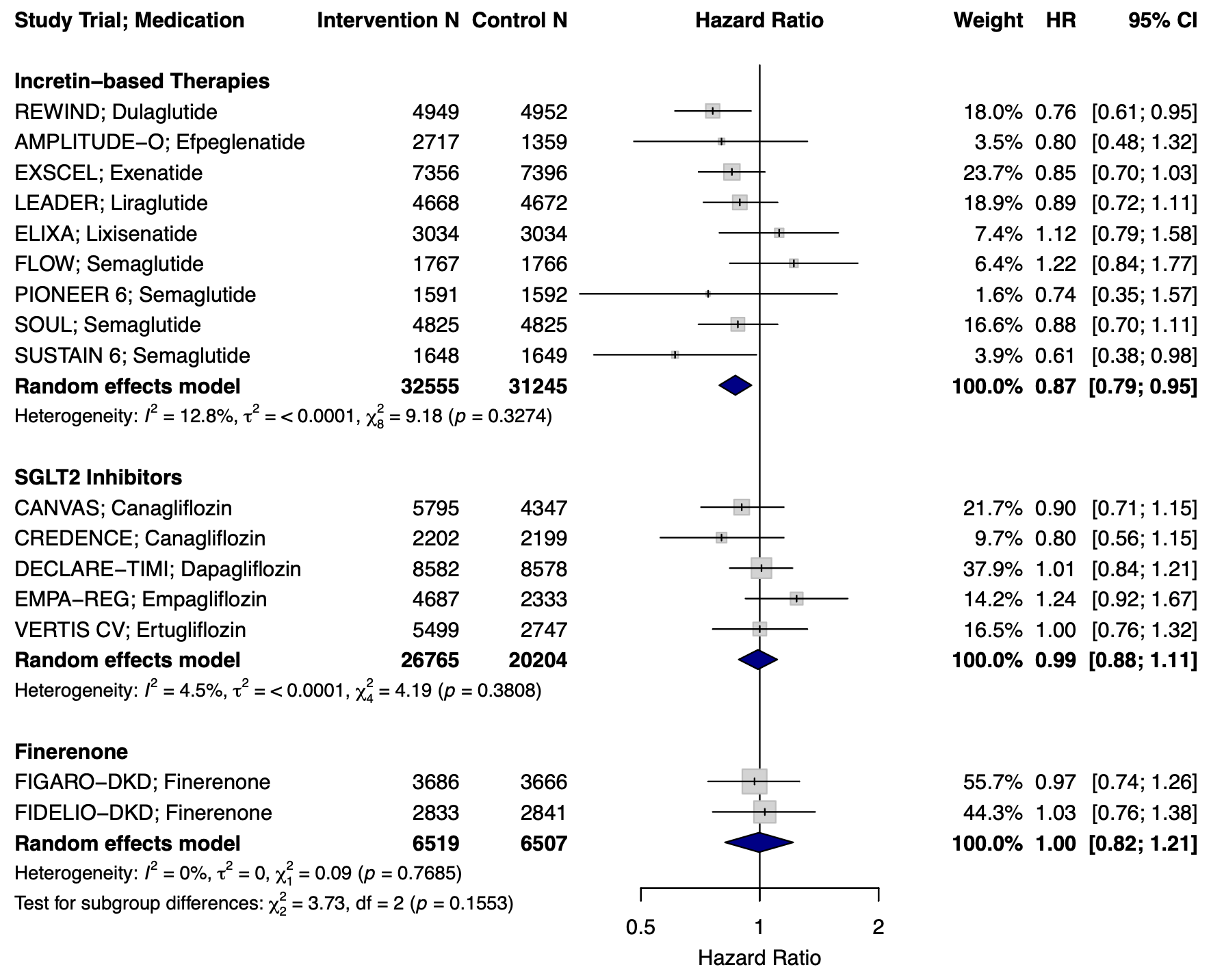
**

**Supplemental Figure S19 – MACE (T2DM with ASCVD / high CVD risk)**

DerSimonian and Laird:


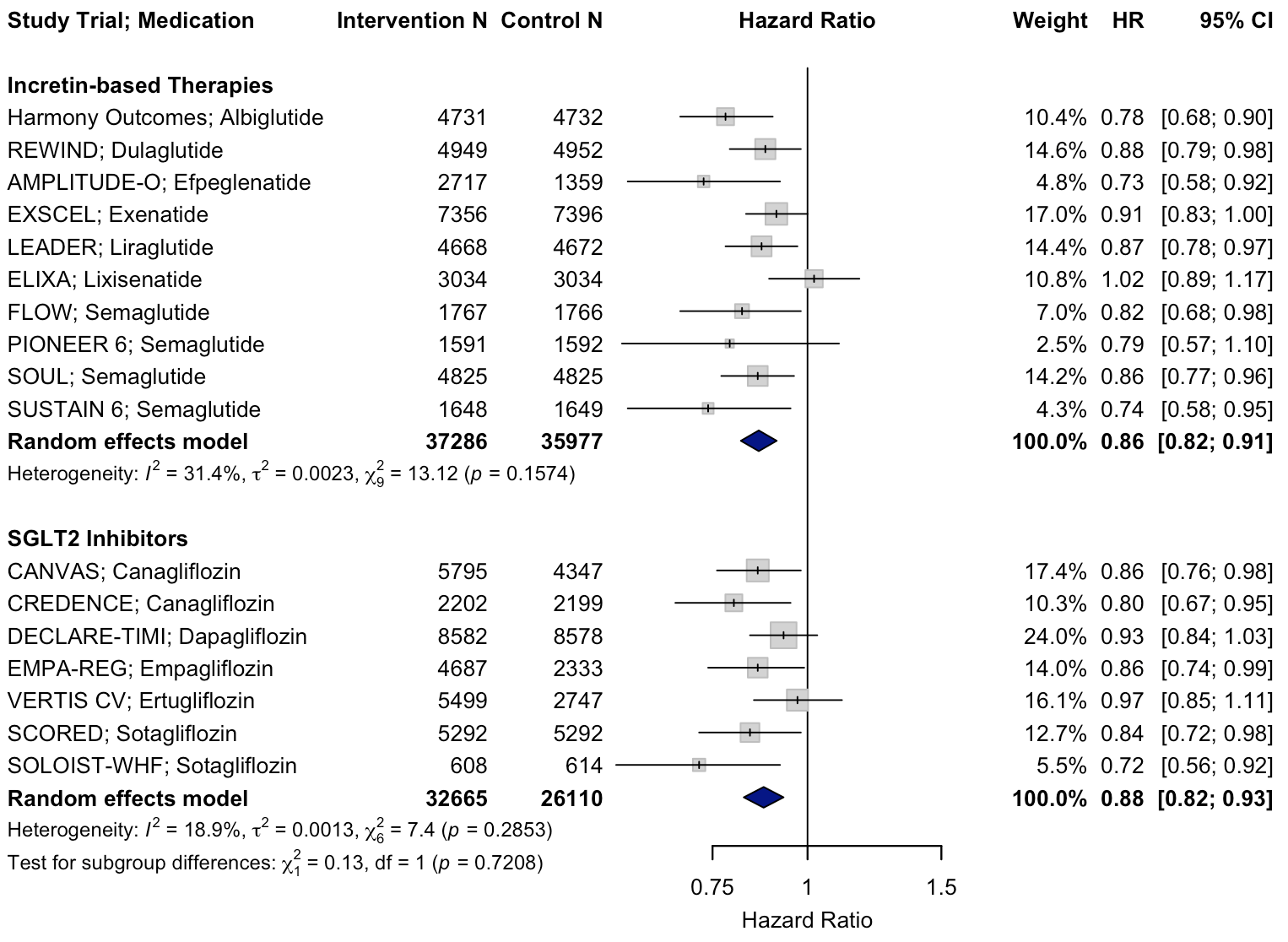


REML–modified HK:

**
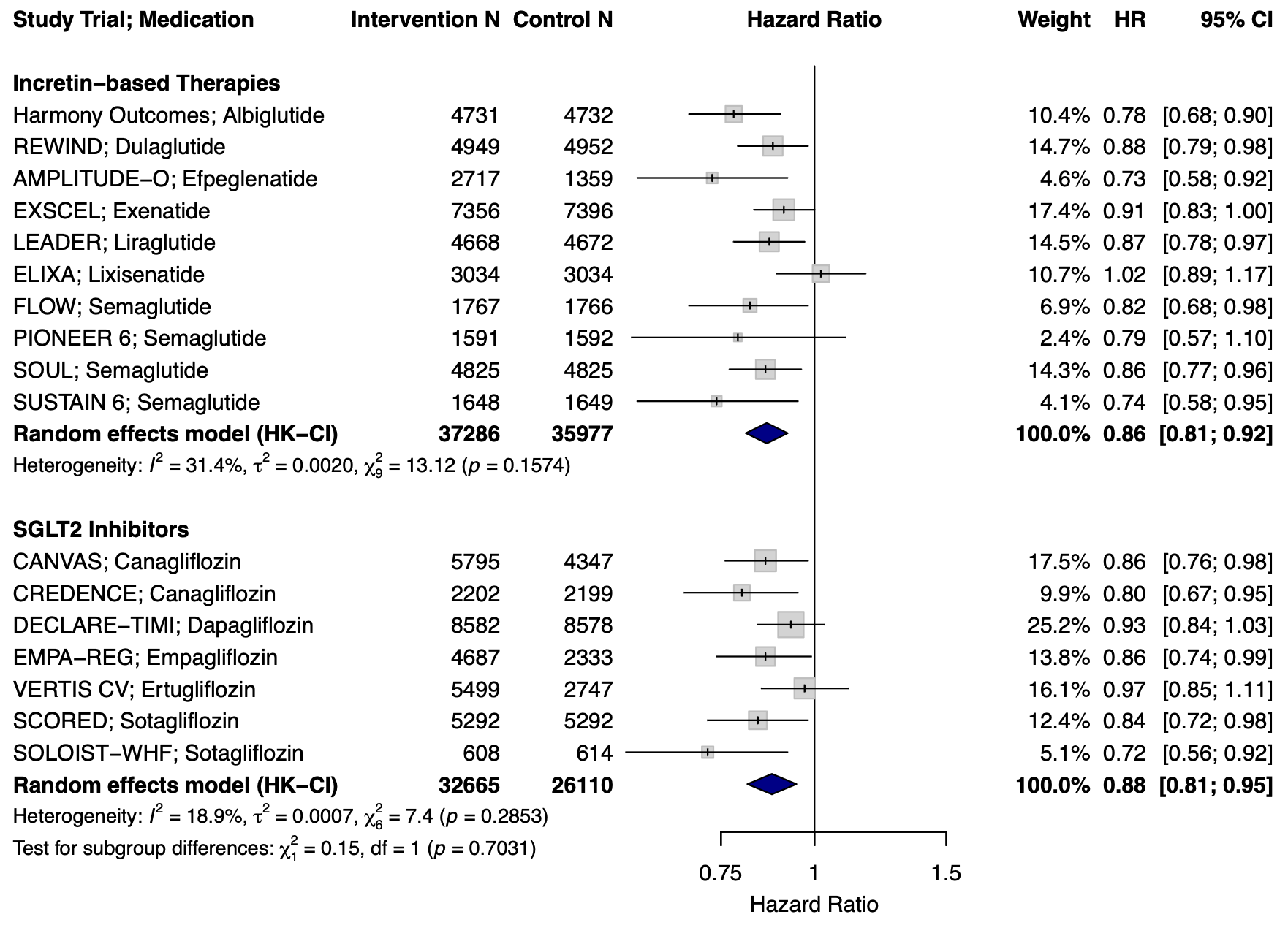
**

REML–Wald:

**
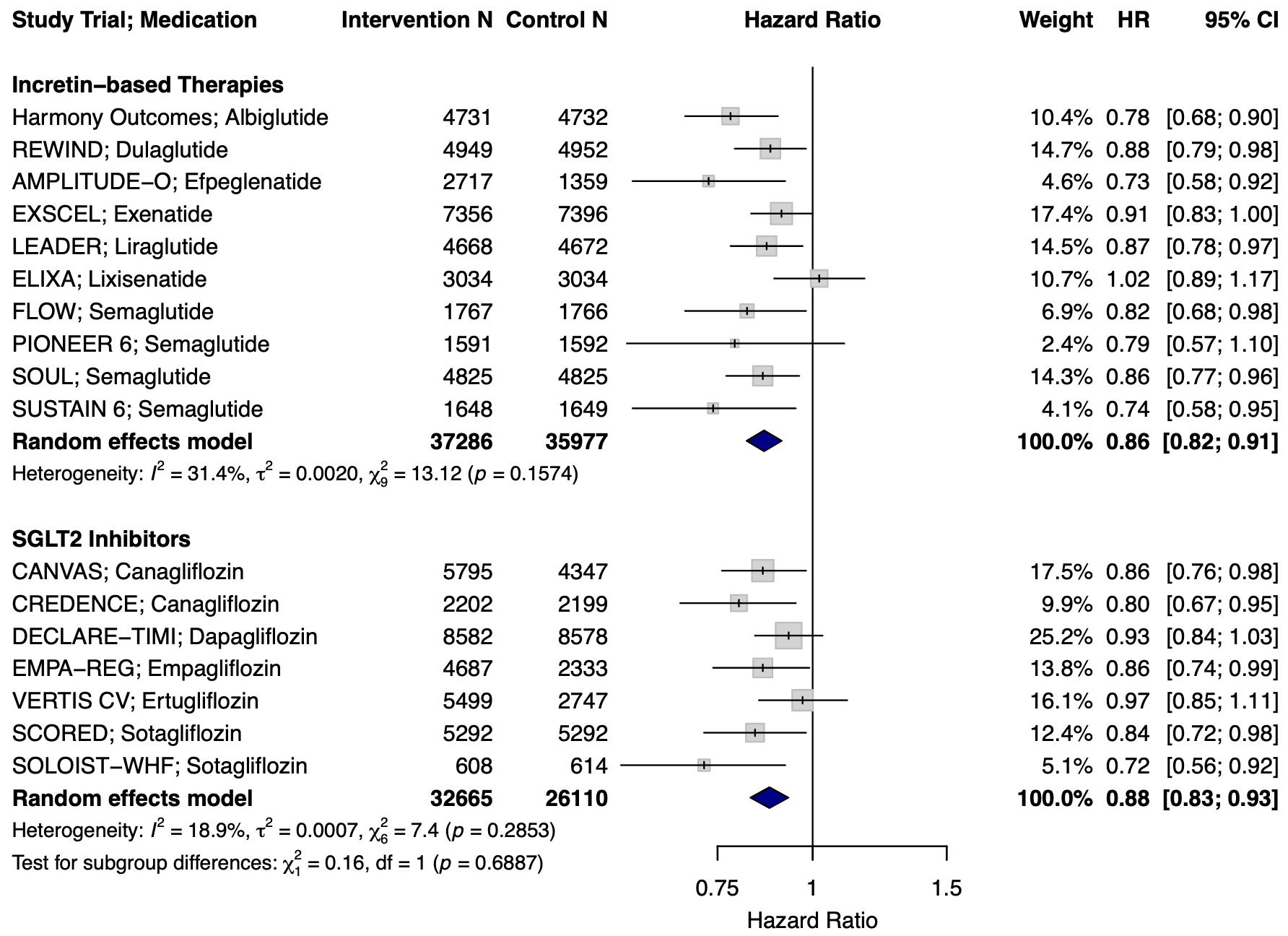
**

**Supplemental Figure S20 – Kidney Composite Outcome (T2DM with ASCVD / high CVD risk)**

DerSimonian and Laird:


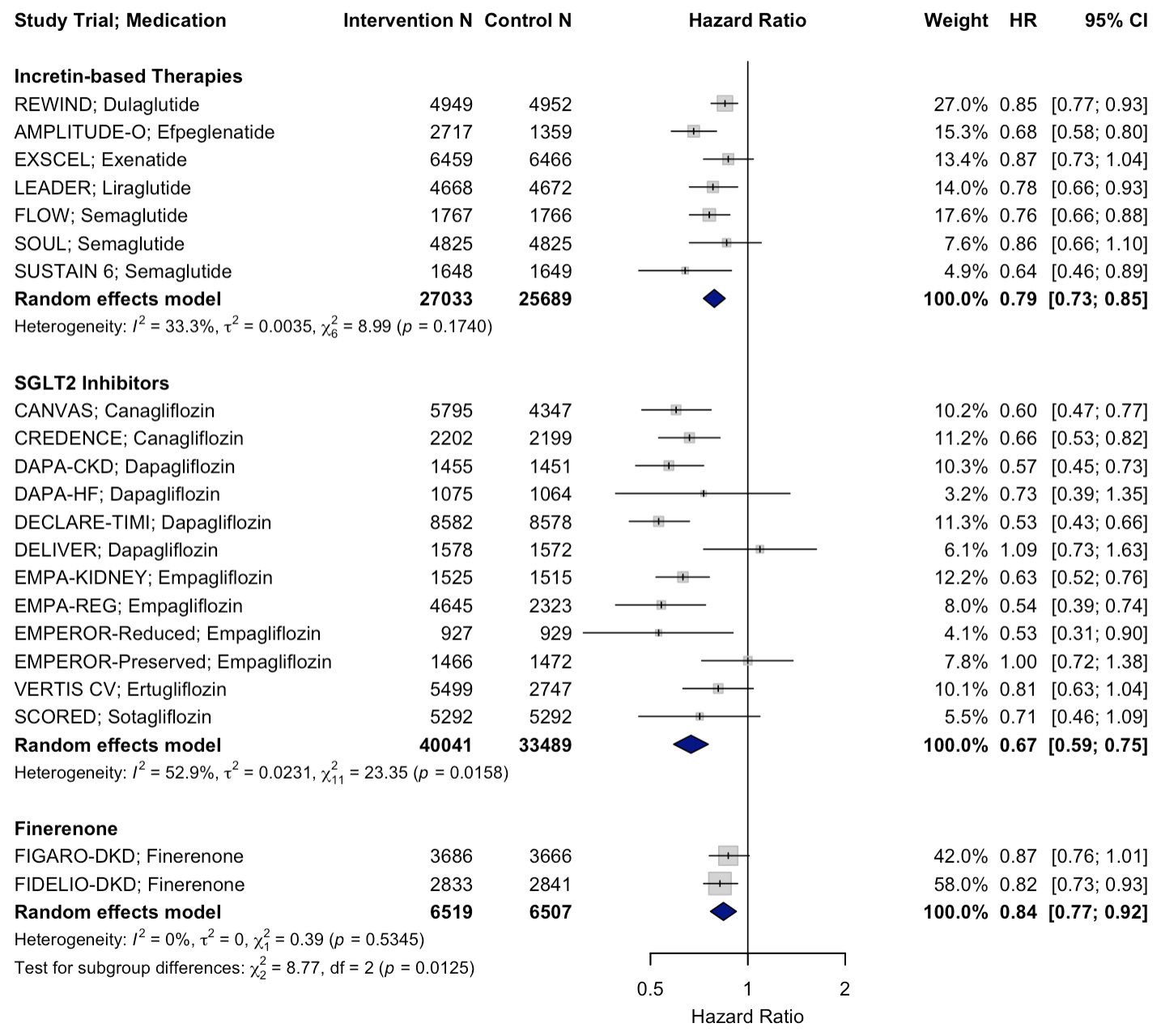


REML–modified HK:

**
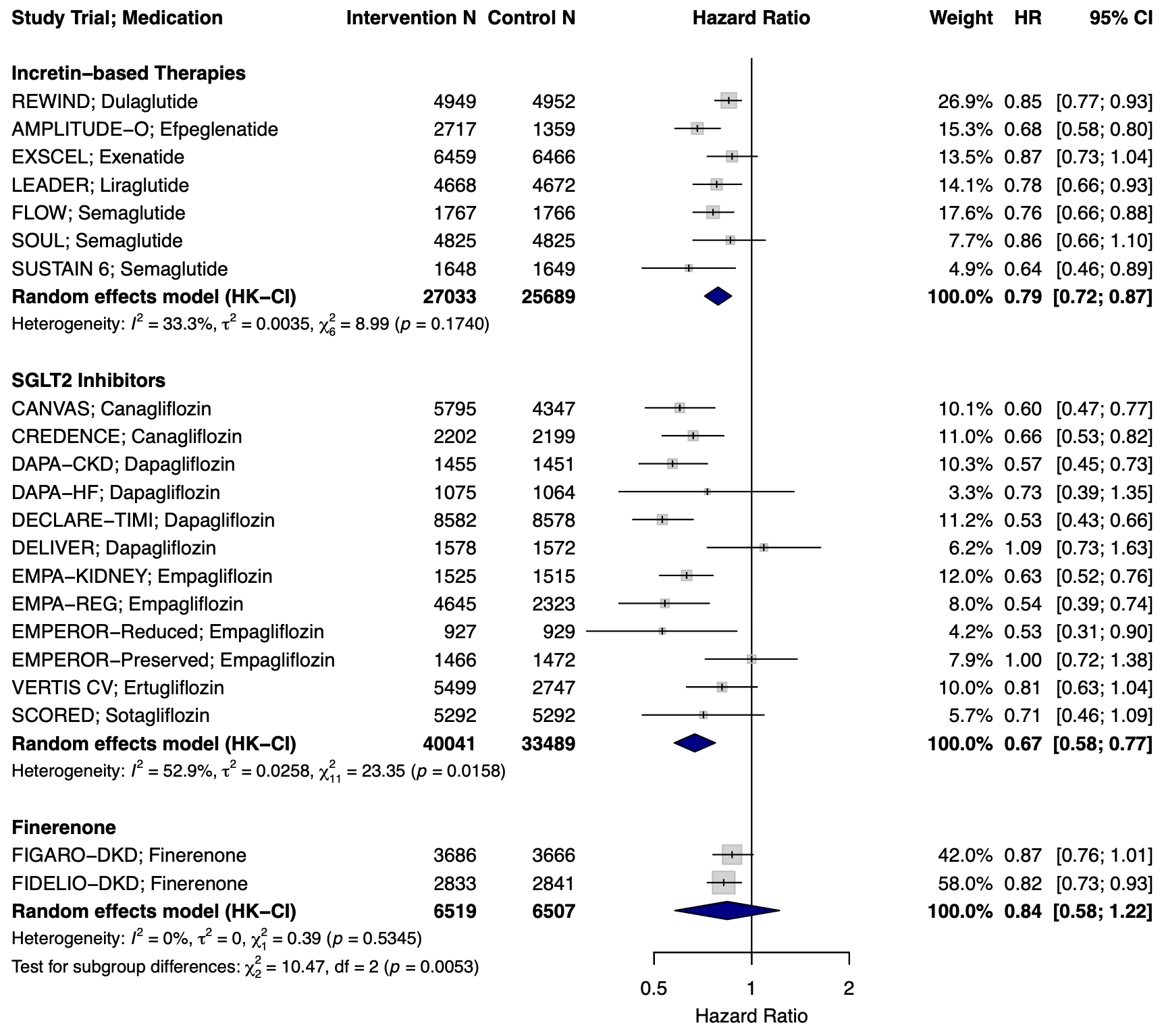
**

REML–Wald:

**
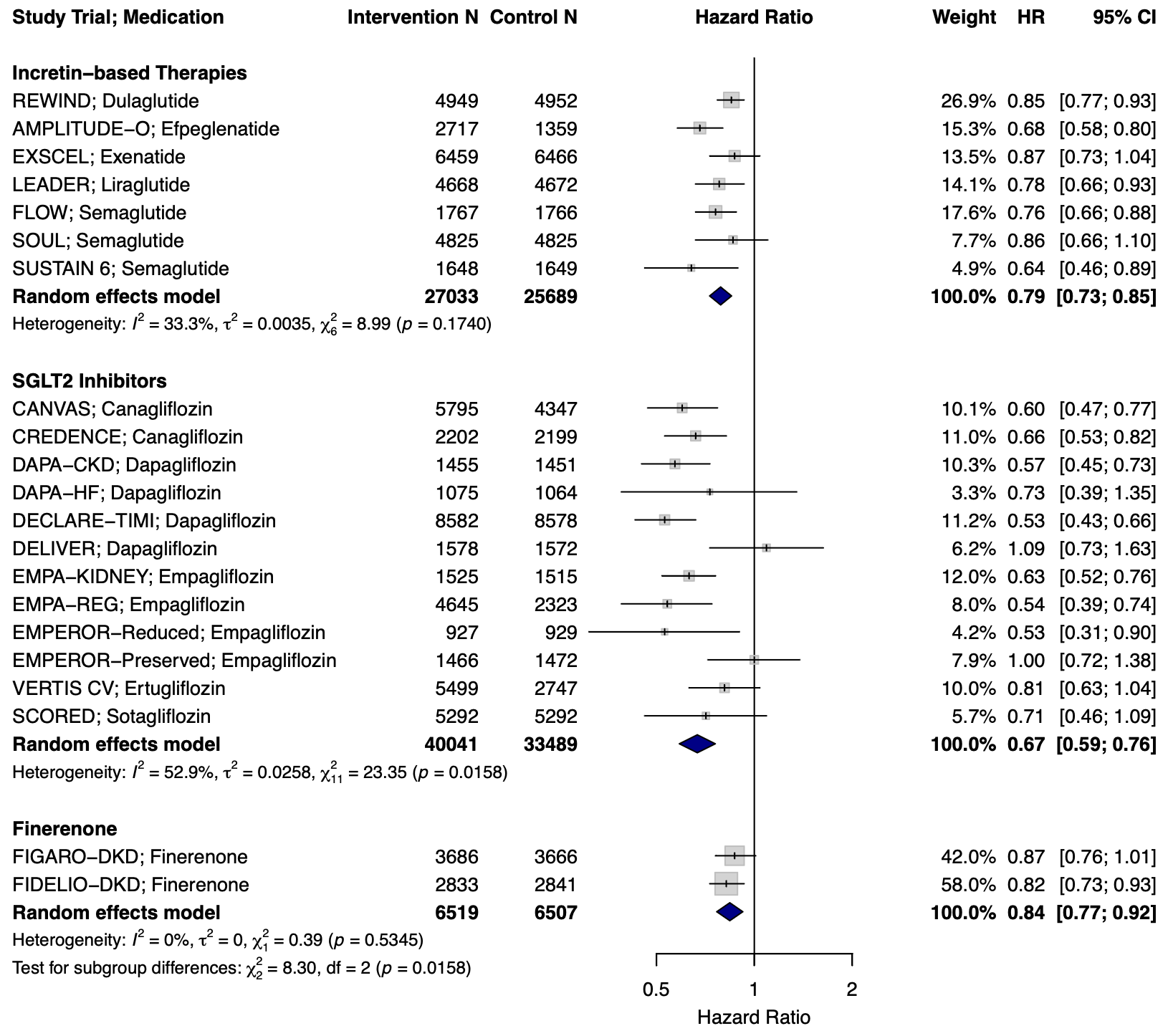
**

### **Chronic Kidney Disease**

**Supplemental Figure S21 – Cardiovascular Mortality (Chronic Kidney Disease)**

DerSimonian and Laird:


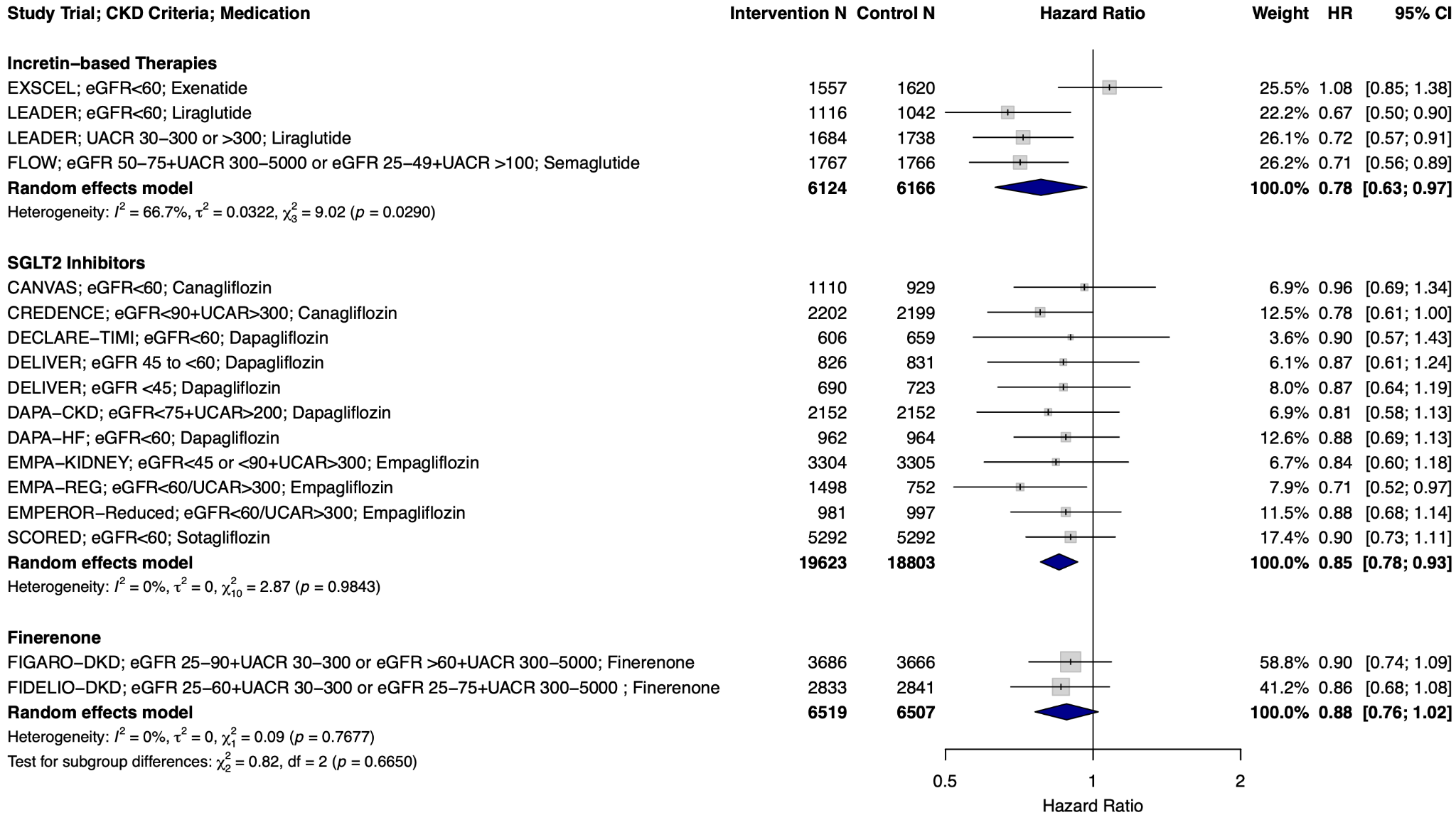


REML–modified HK:

**
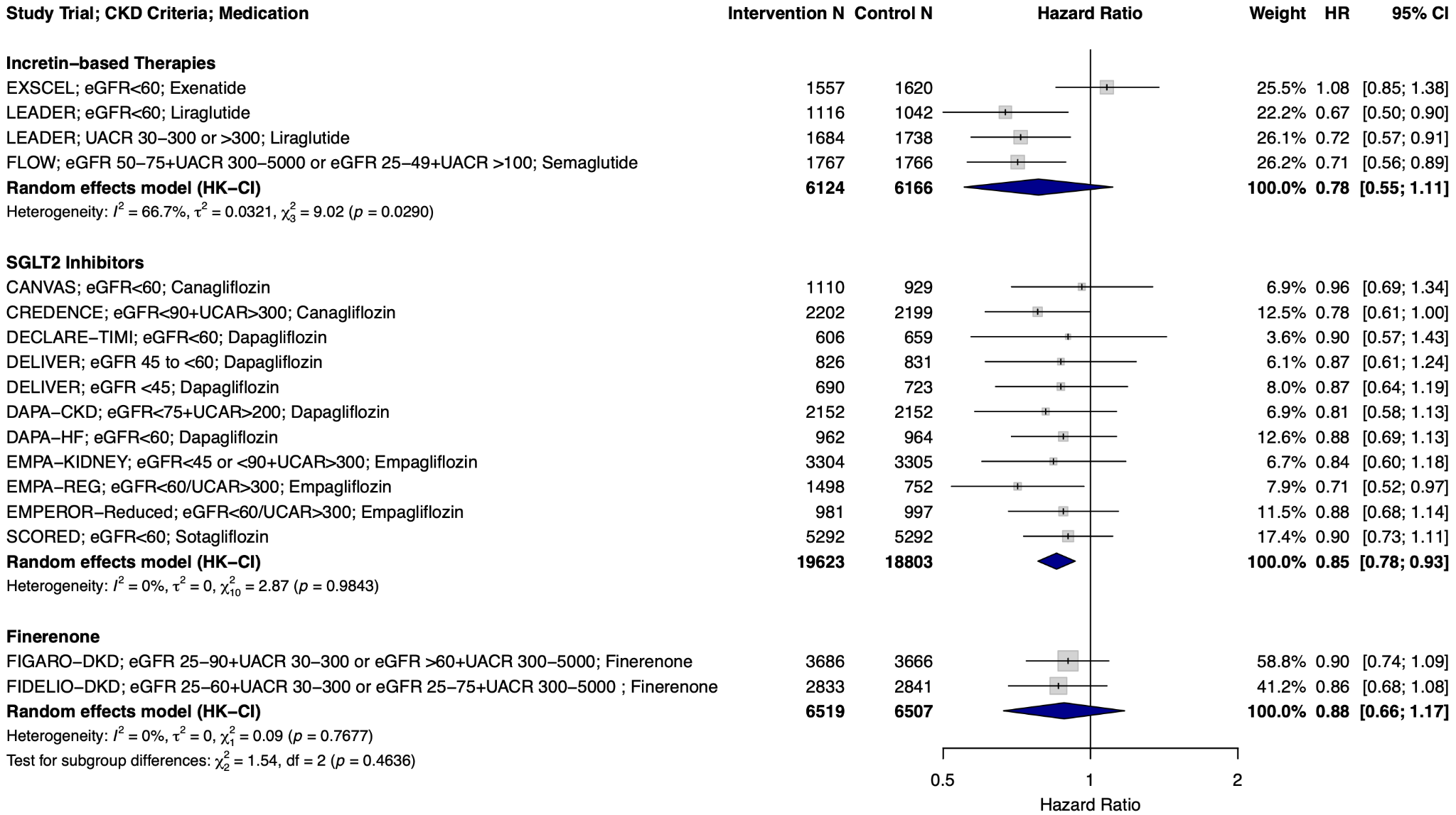
**

REML–Wald:

**
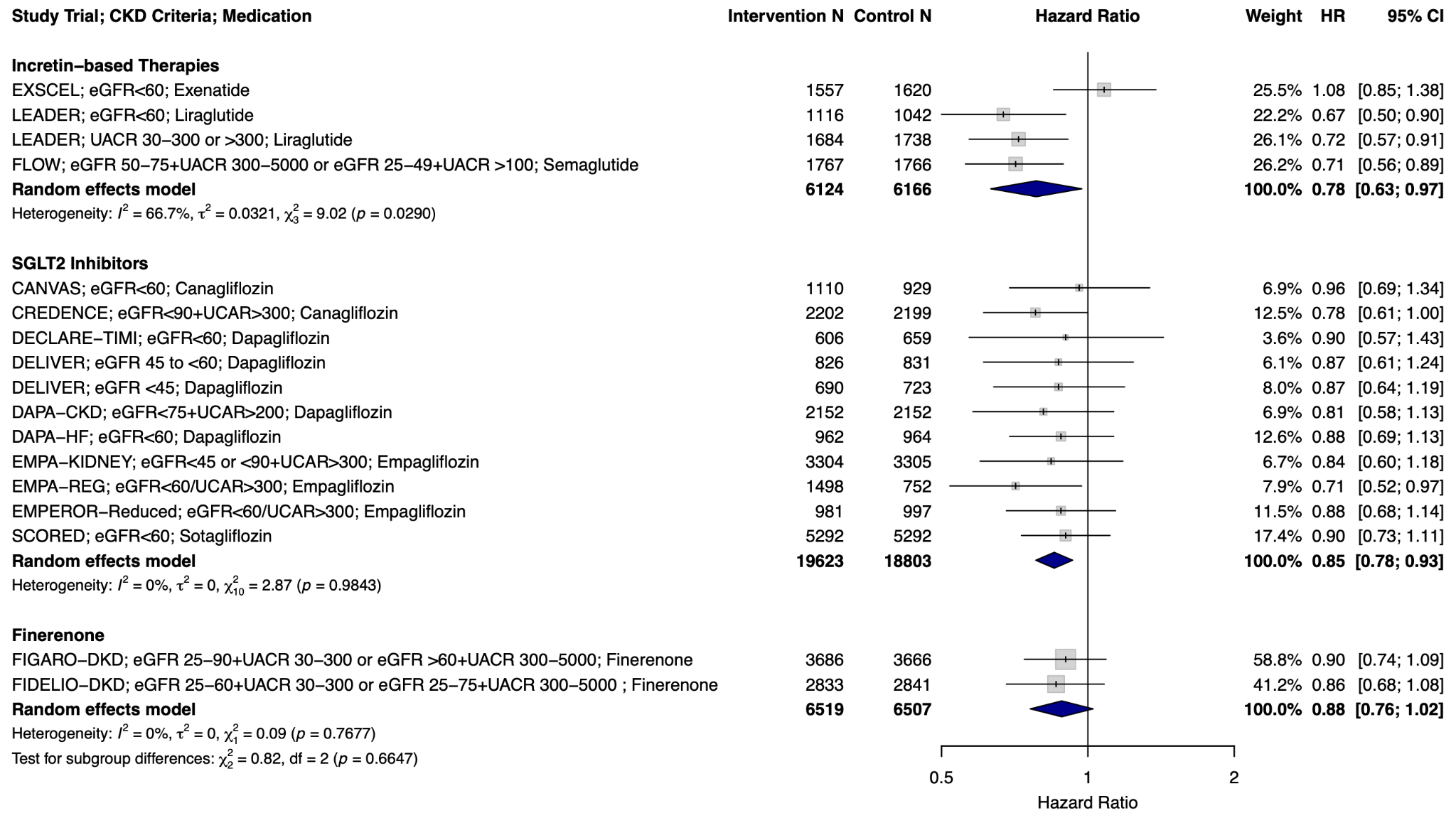
**

**Supplemental Figure S22 – All-Cause Mortality (Chronic Kidney Disease)**

DerSimonian and Laird:


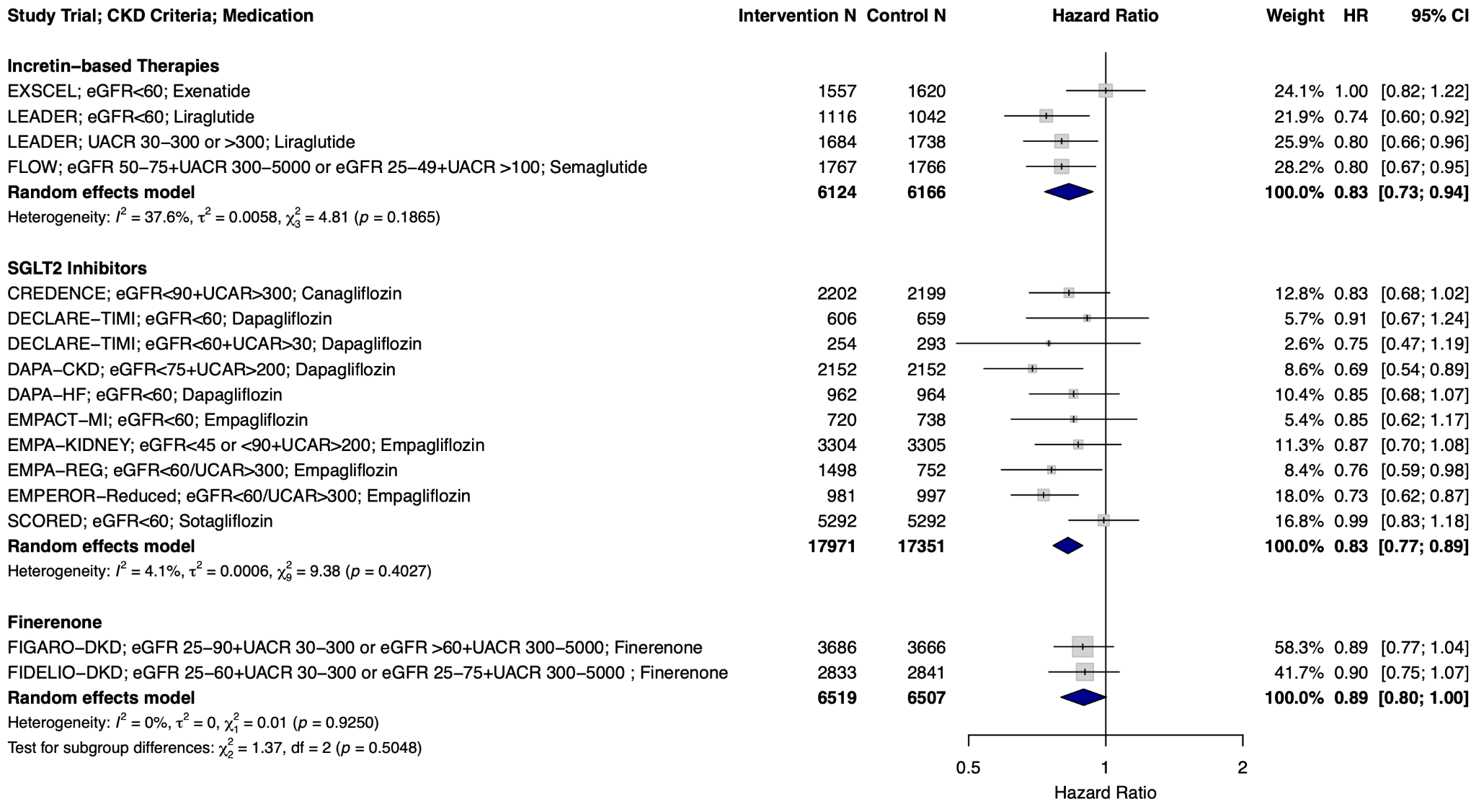


REML–modified HK:


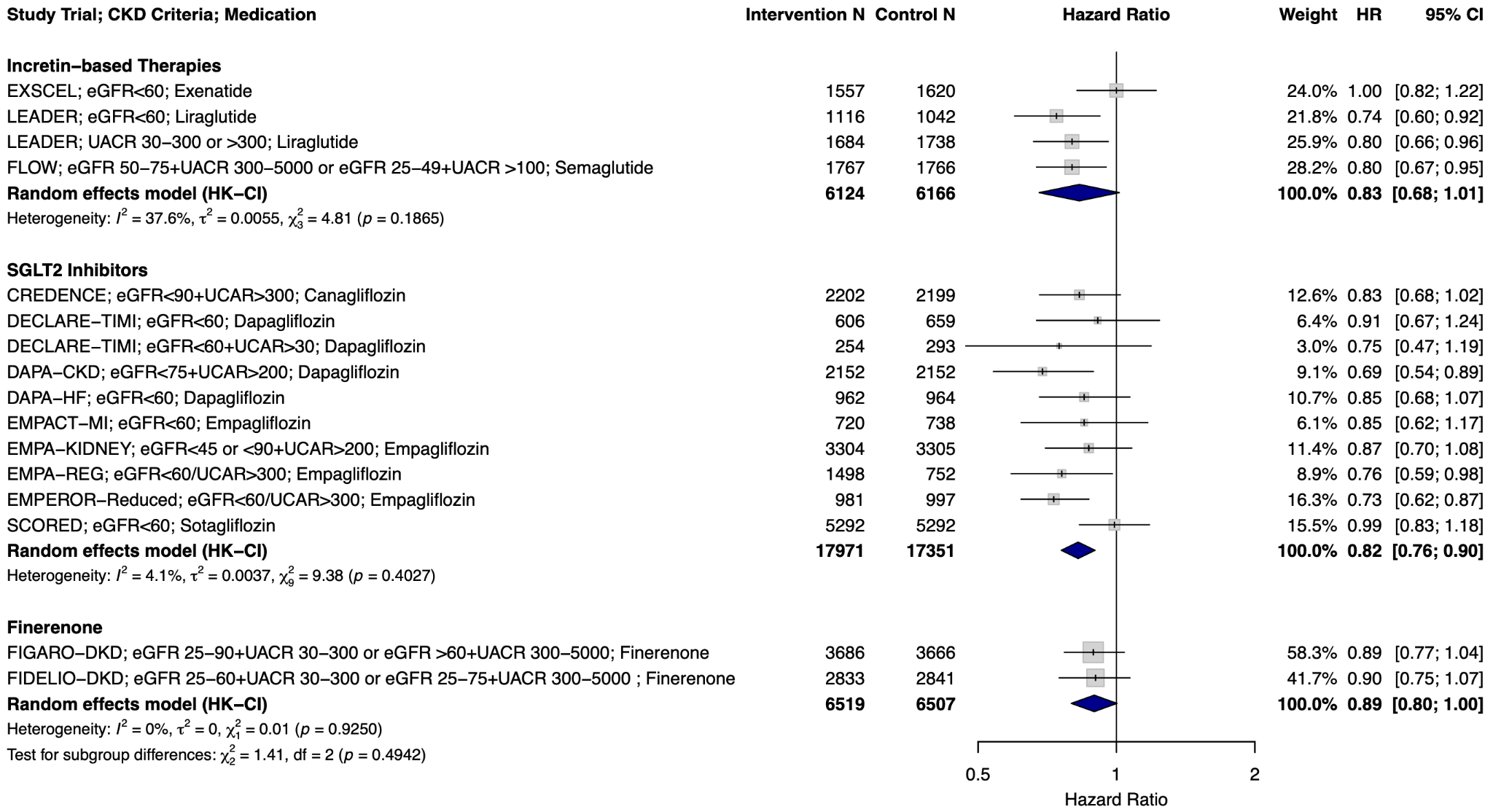


REML–Wald:


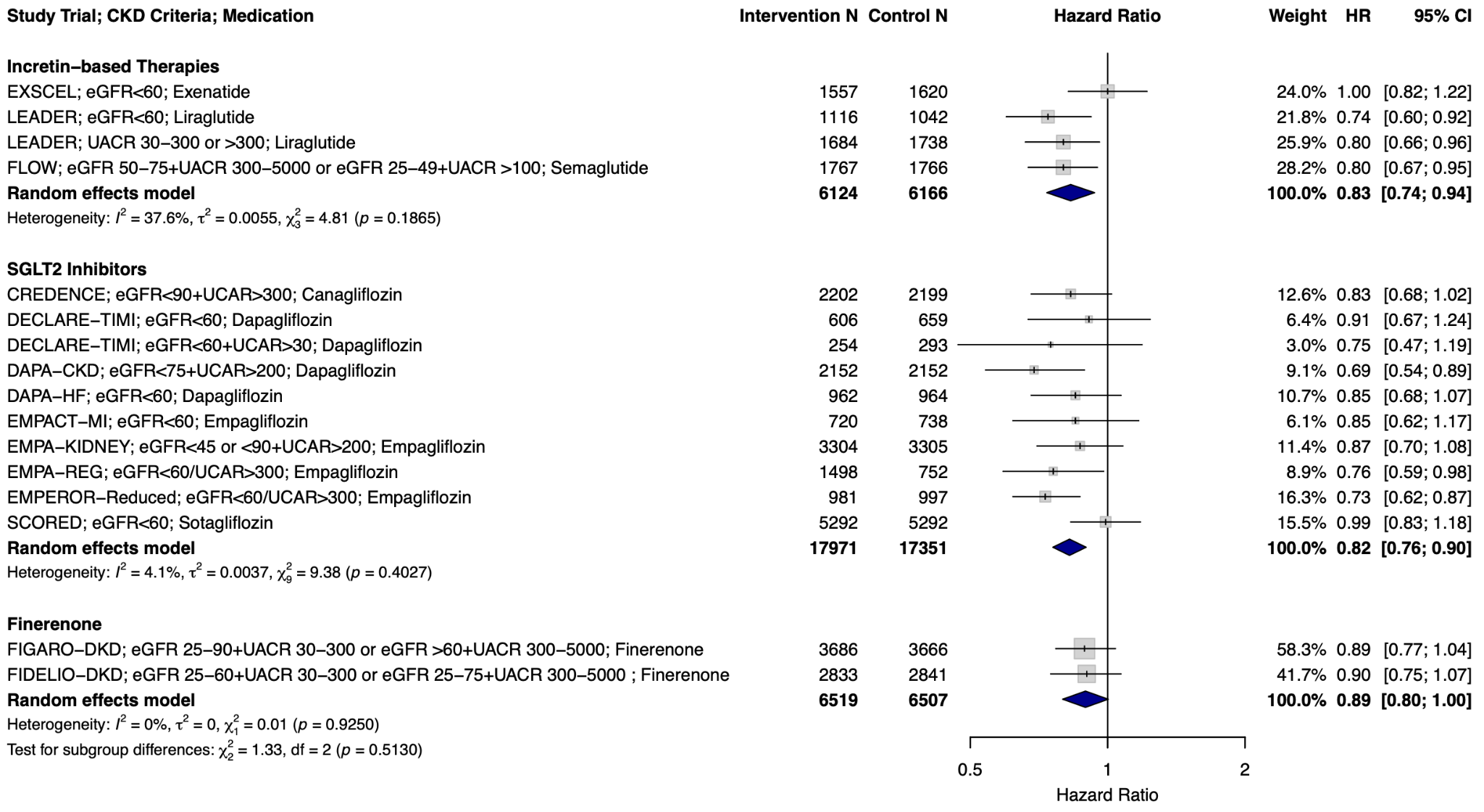


**Supplemental Figure S23 – CV Mortality or HF Hospitalization/HF Events (Chronic Kidney Disease)**

DerSimonian and Laird:


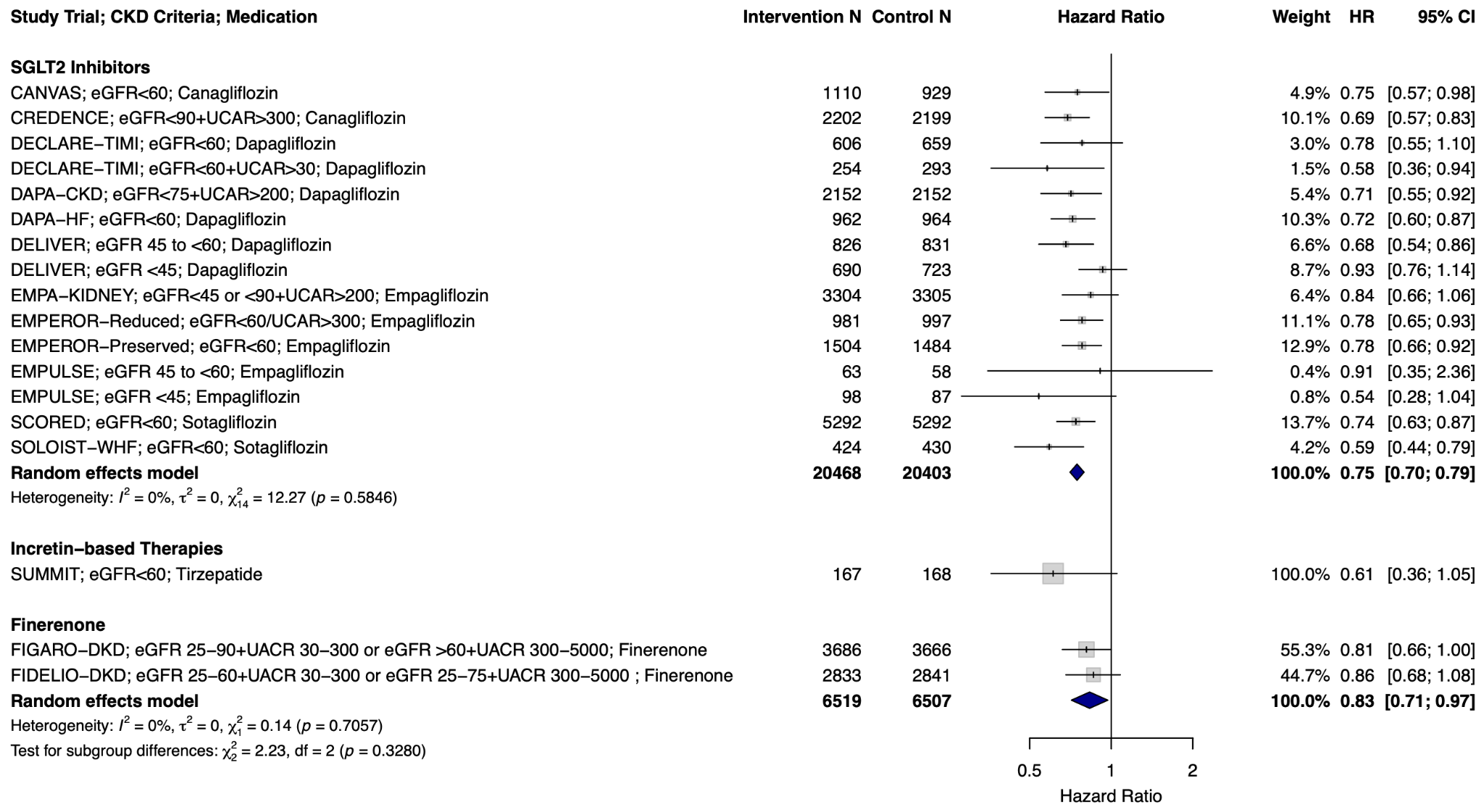


REML–modified HK:

**
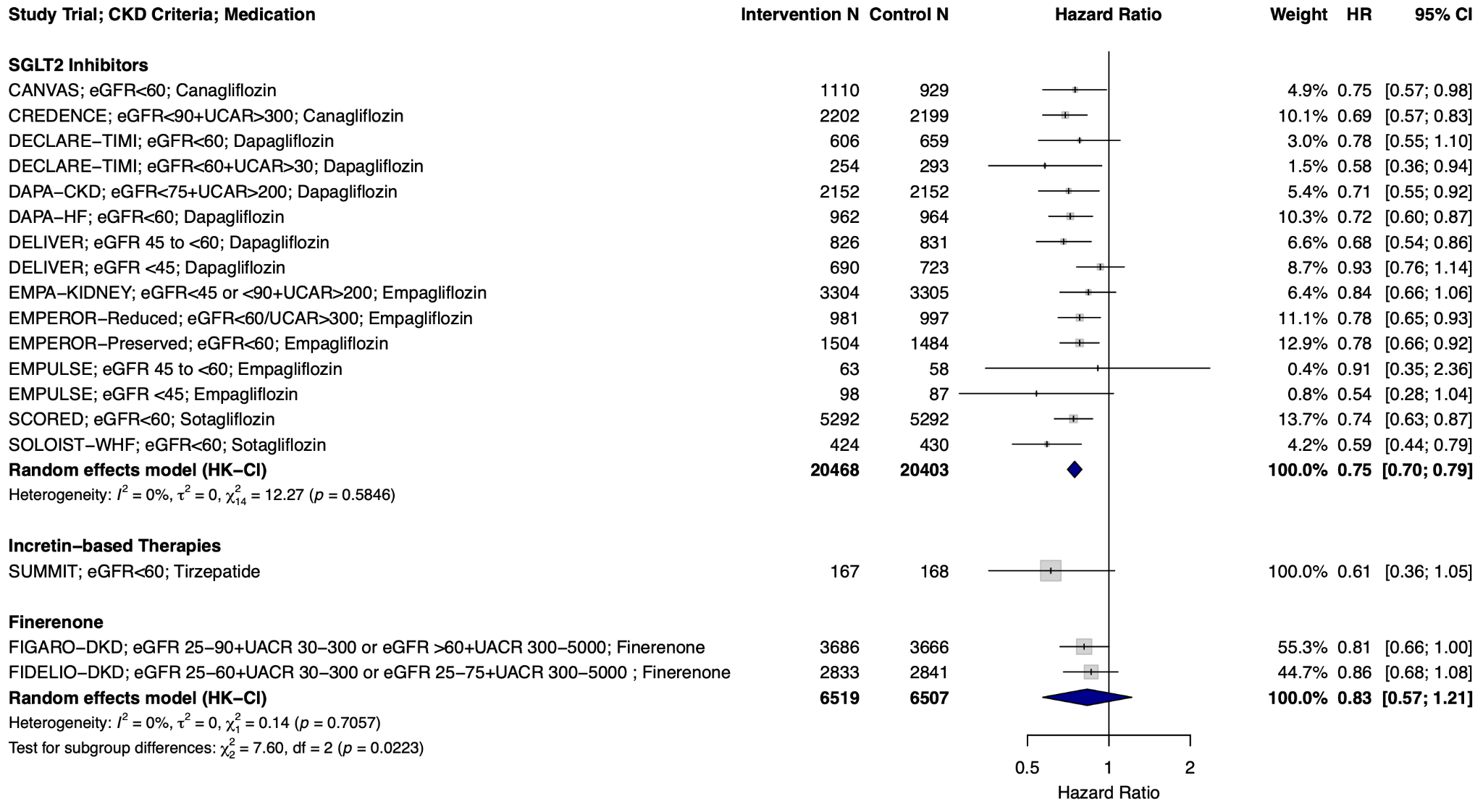
**

REML–Wald:

**
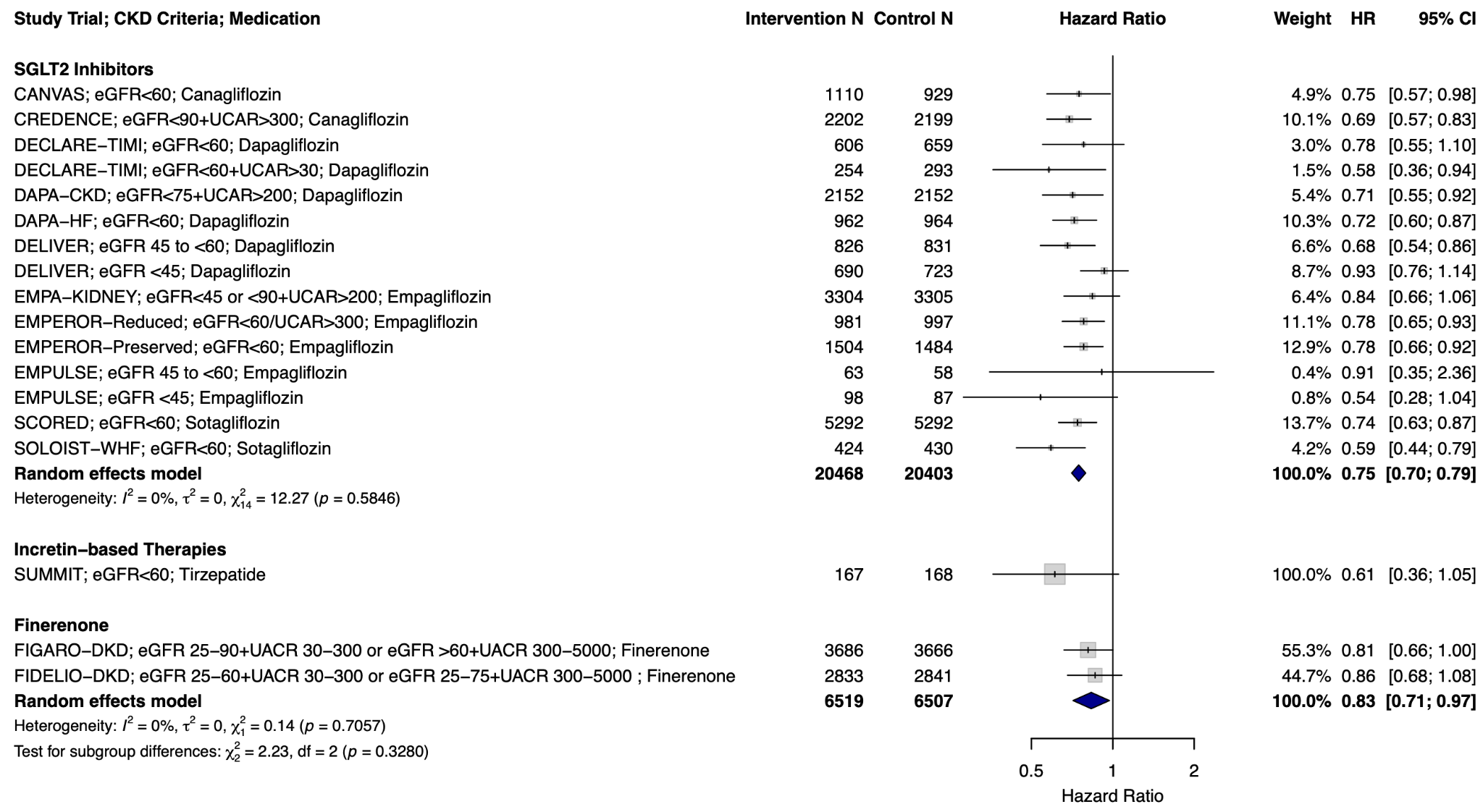
**

**Supplemental Figure S24 – CV Mortality or HF Hospitalization (Chronic Kidney Disease)**

DerSimonian and Laird:


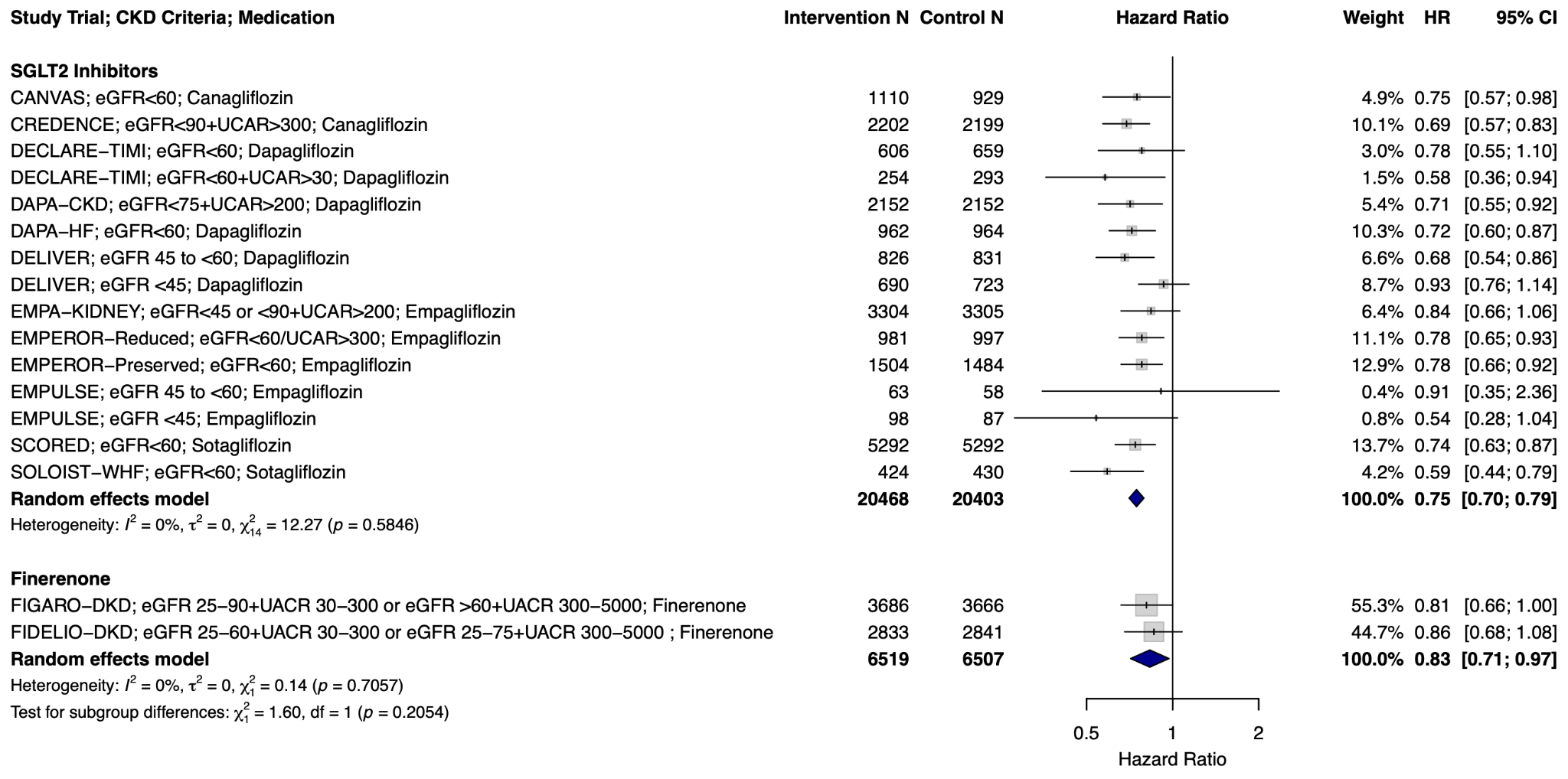


REML–modified HK:


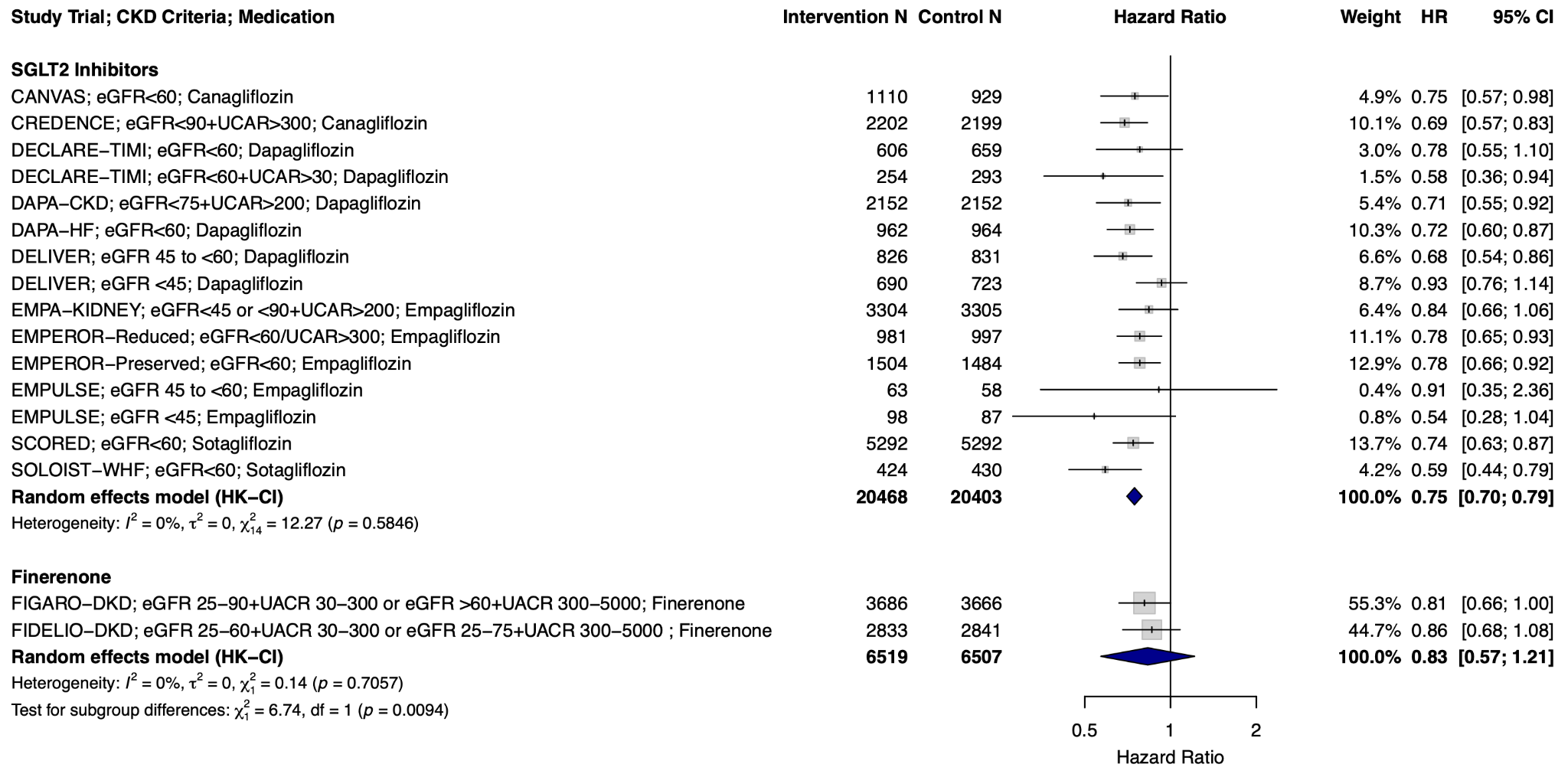


REML–Wald:


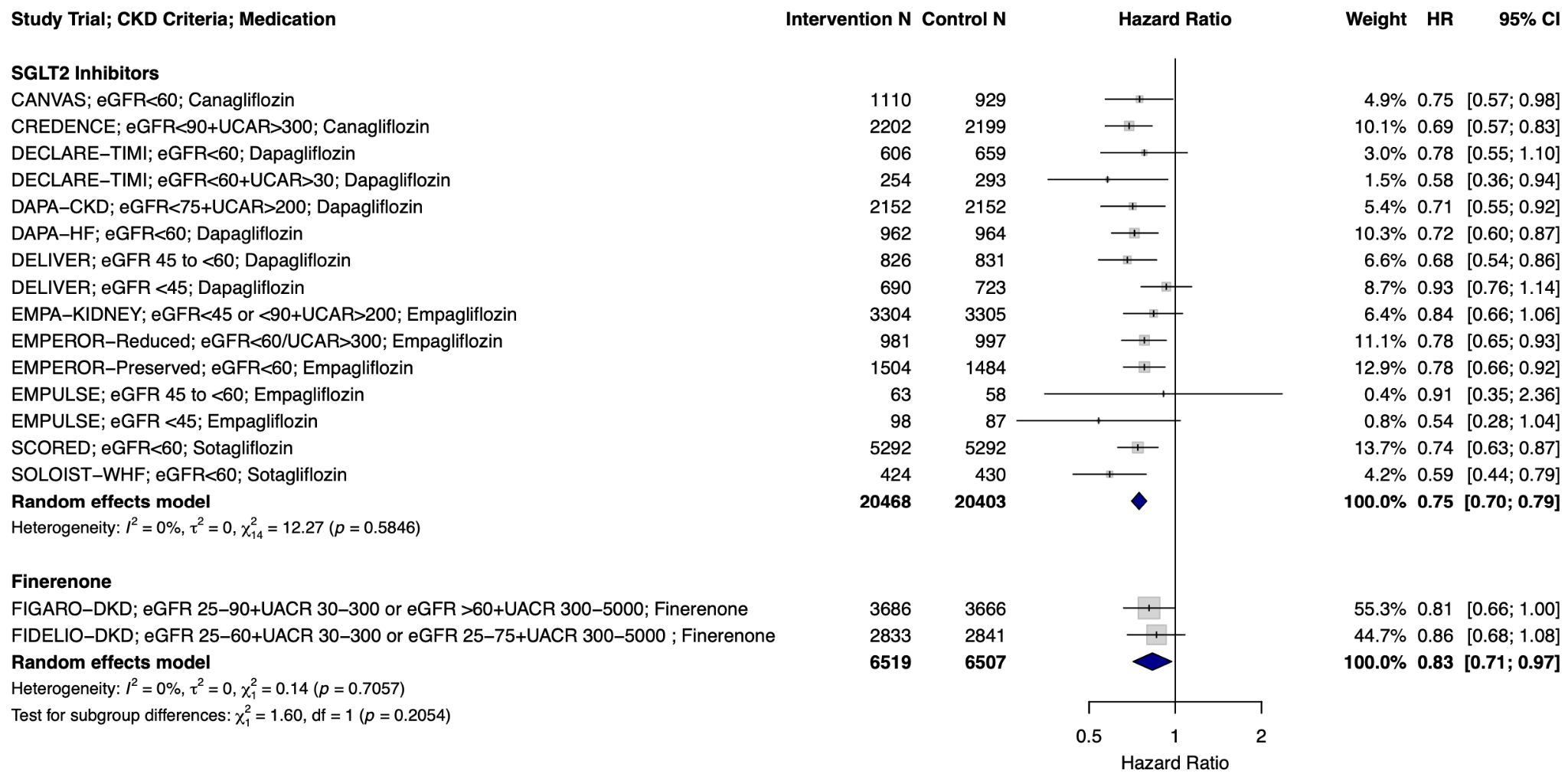


**Supplemental Figure S25 – HF Hospitalization (Chronic Kidney Disease)**

DerSimonian and Laird:


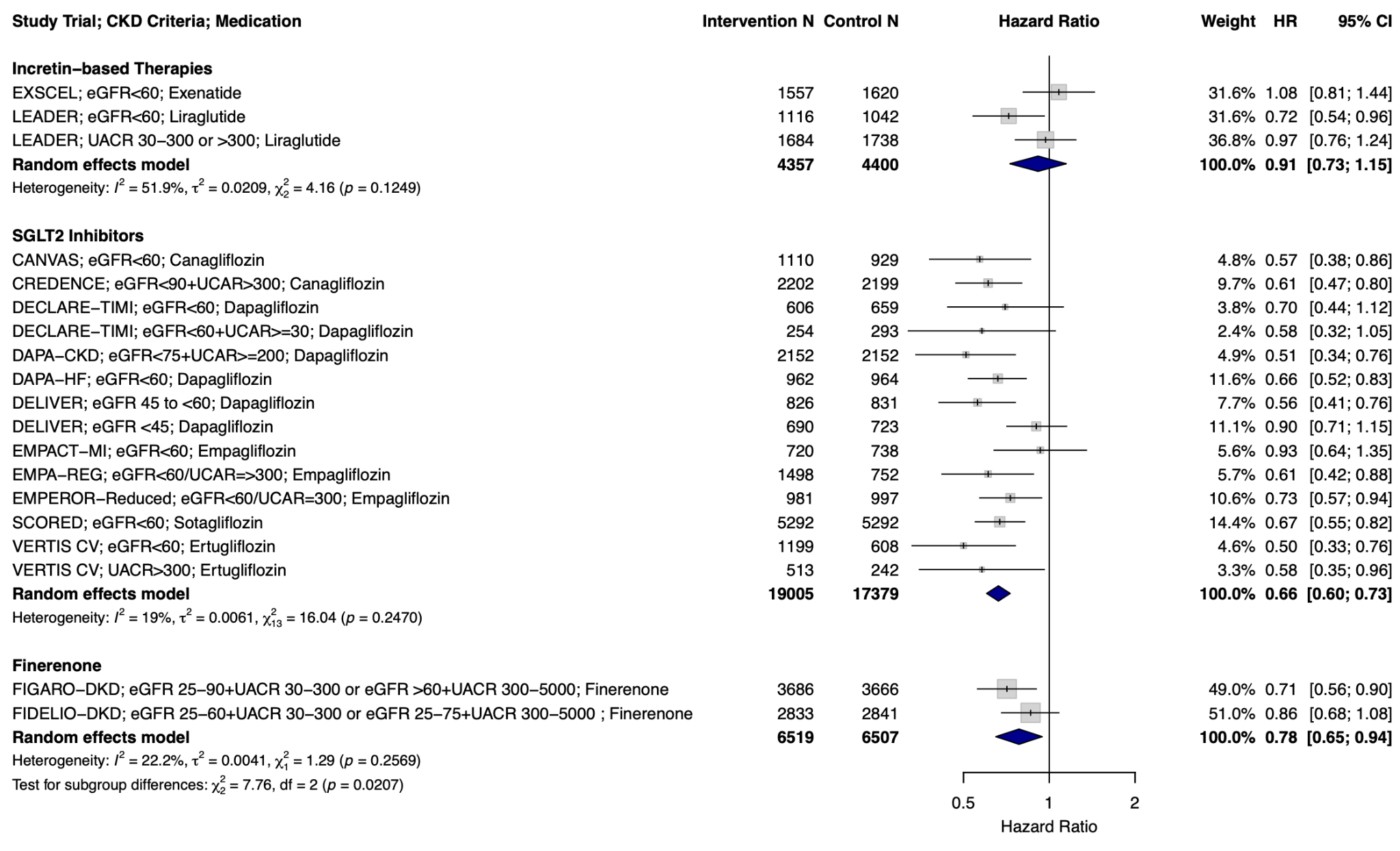


REML–modified HK: **
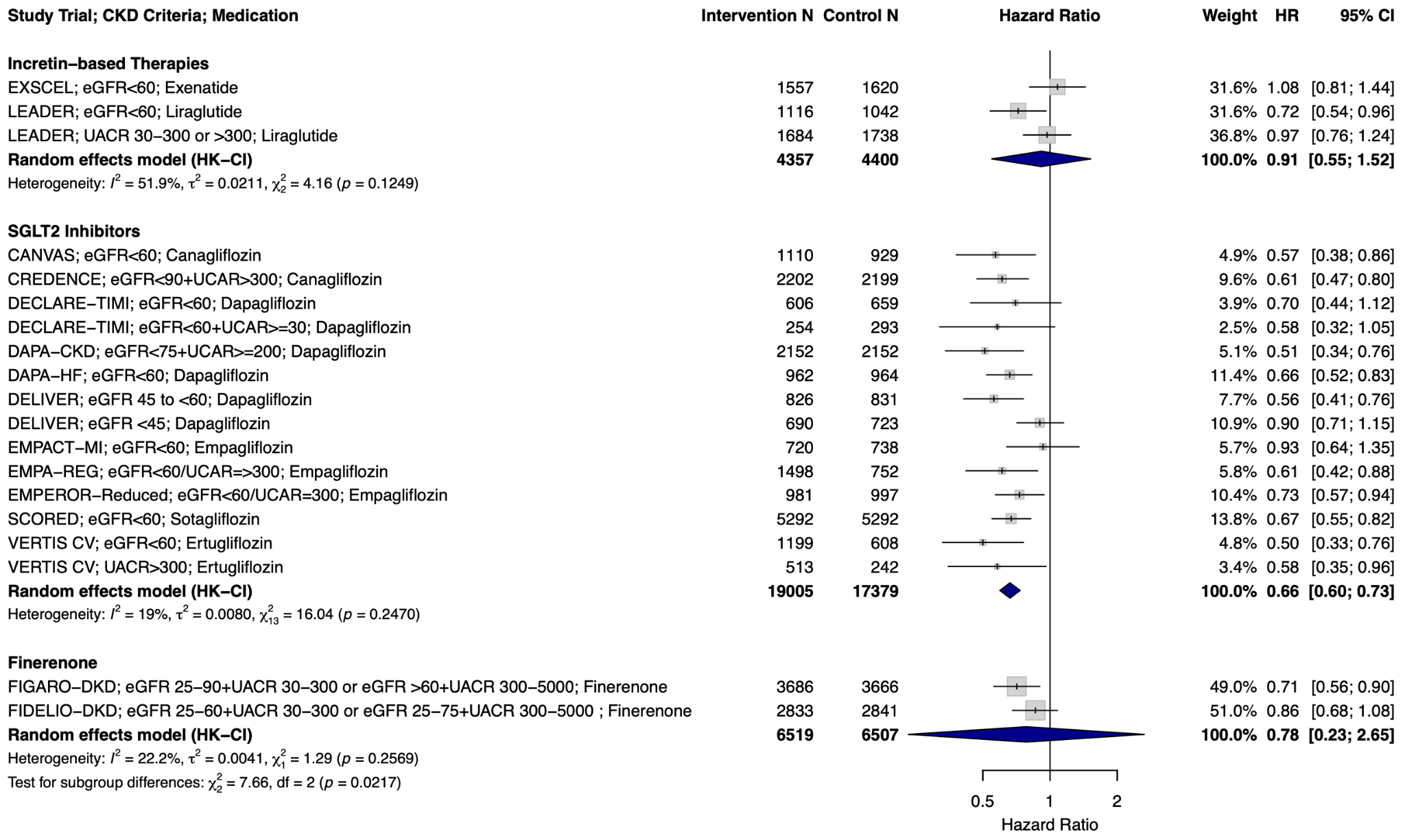
**

REML–Wald:

**
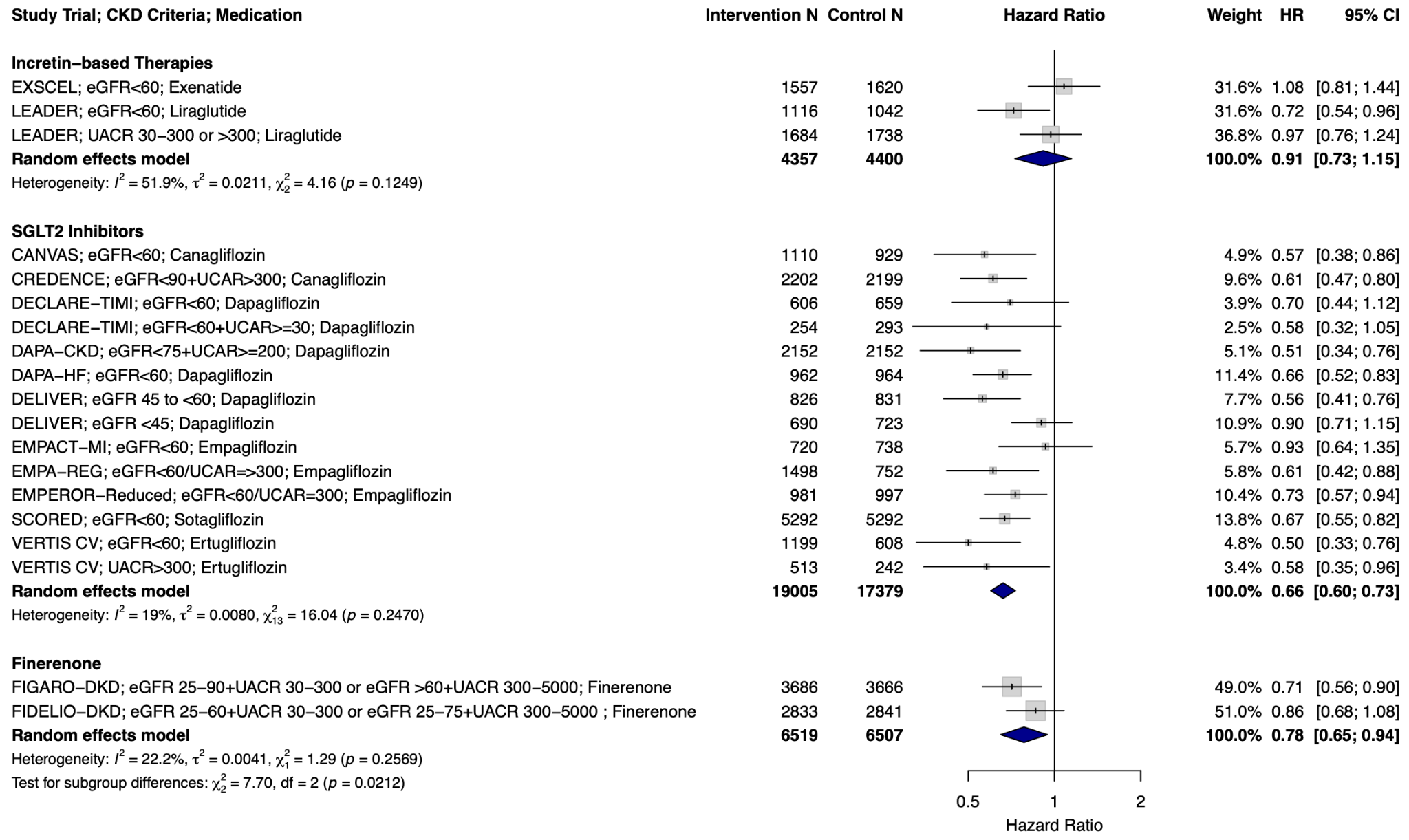
**

**Supplemental Figure S26 – Non-fatal Myocardial Infarction (Chronic Kidney Disease)**

DerSimonian and Laird:


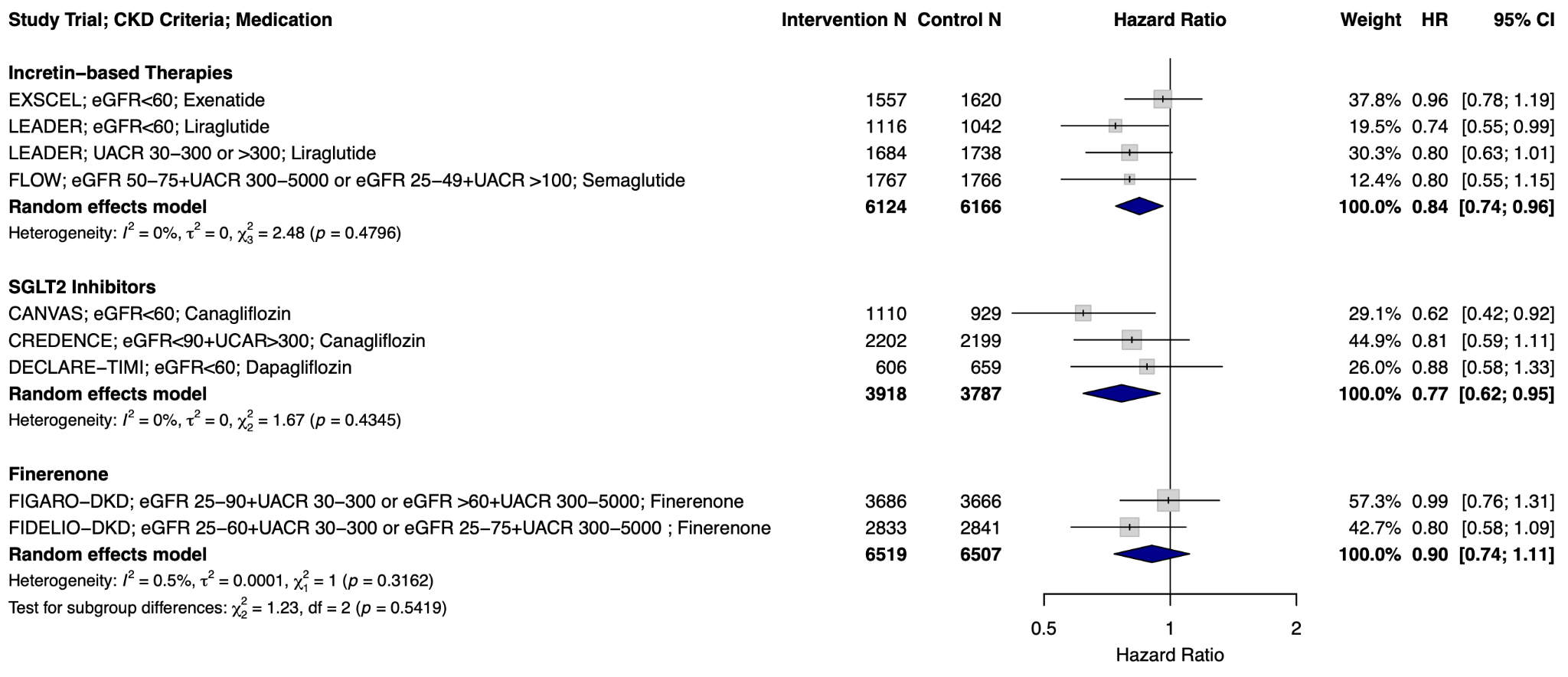


REML–modified HK:

**
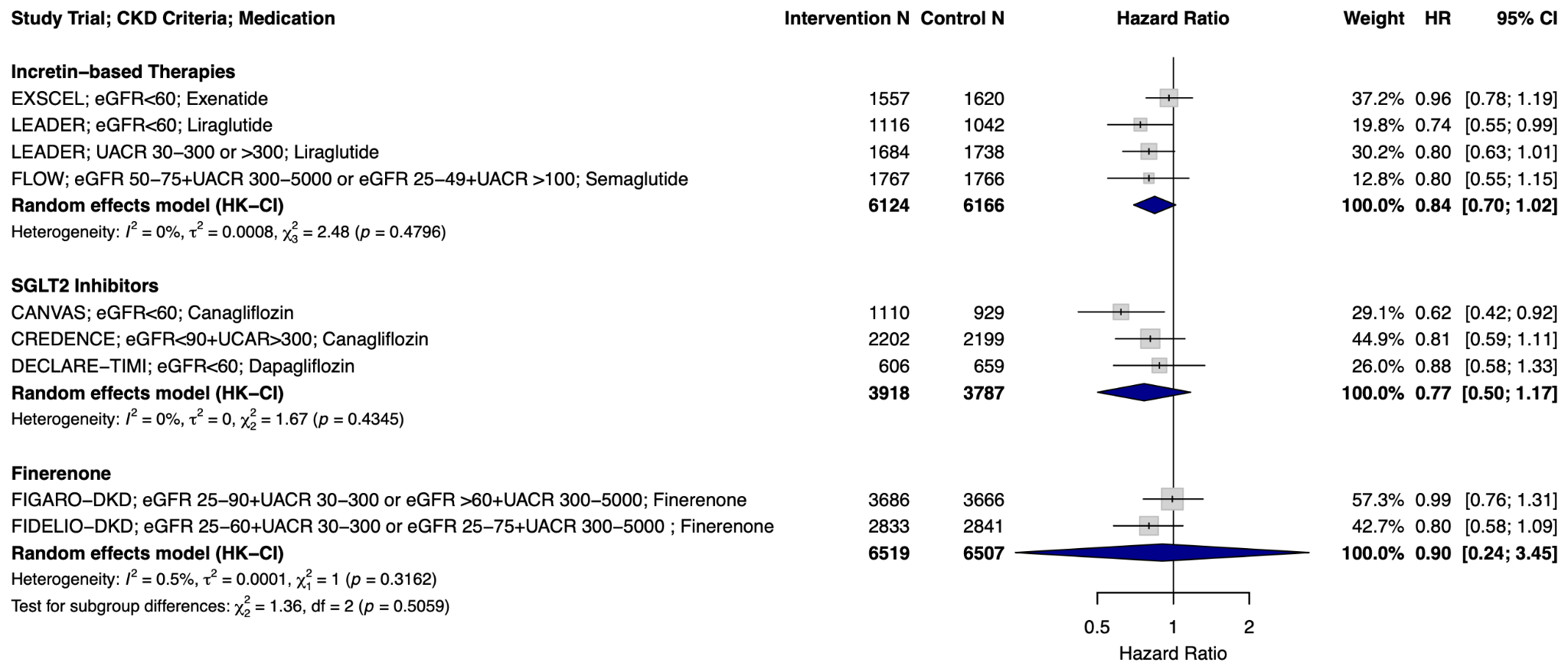
**

REML–Wald:

**
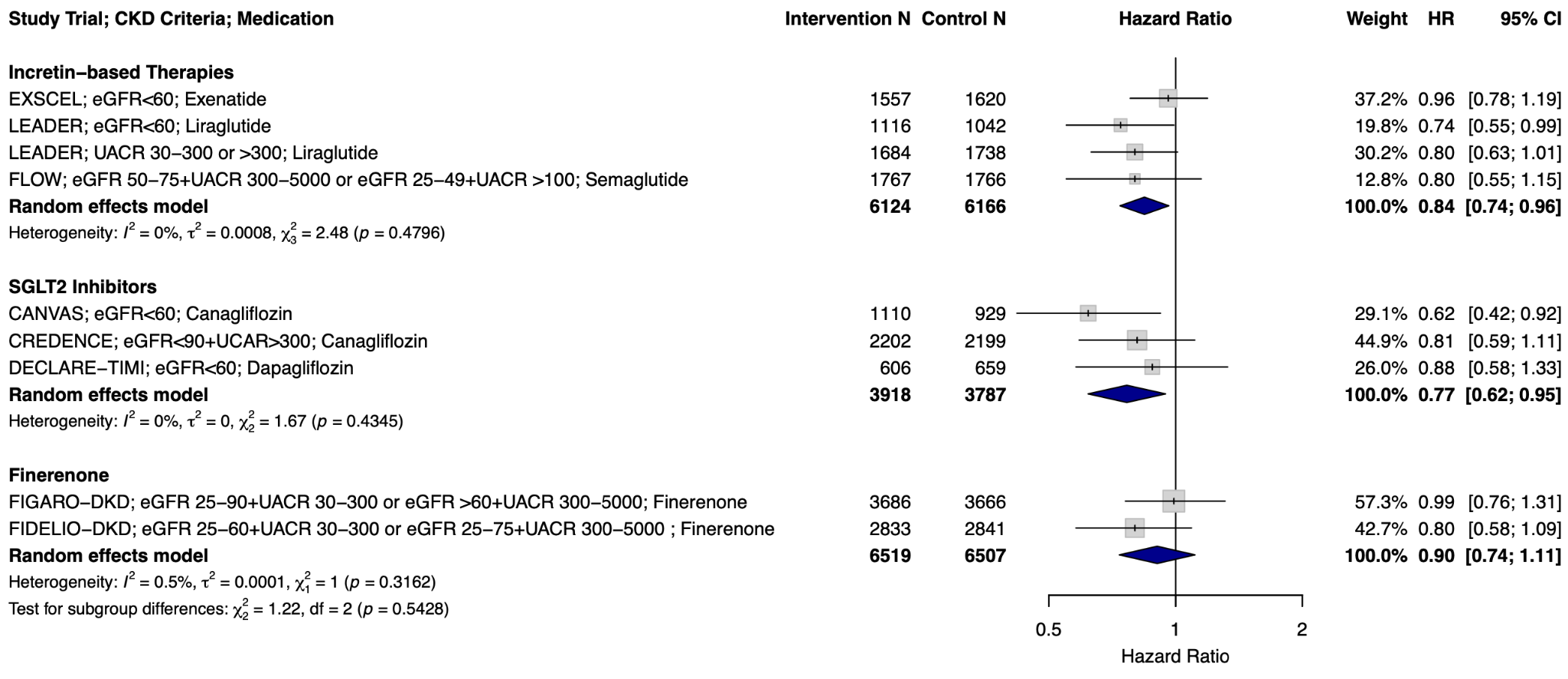
**

**Supplemental Figure S27 – Non-fatal Stroke (Chronic Kidney Disease)**

DerSimonian and Laird:


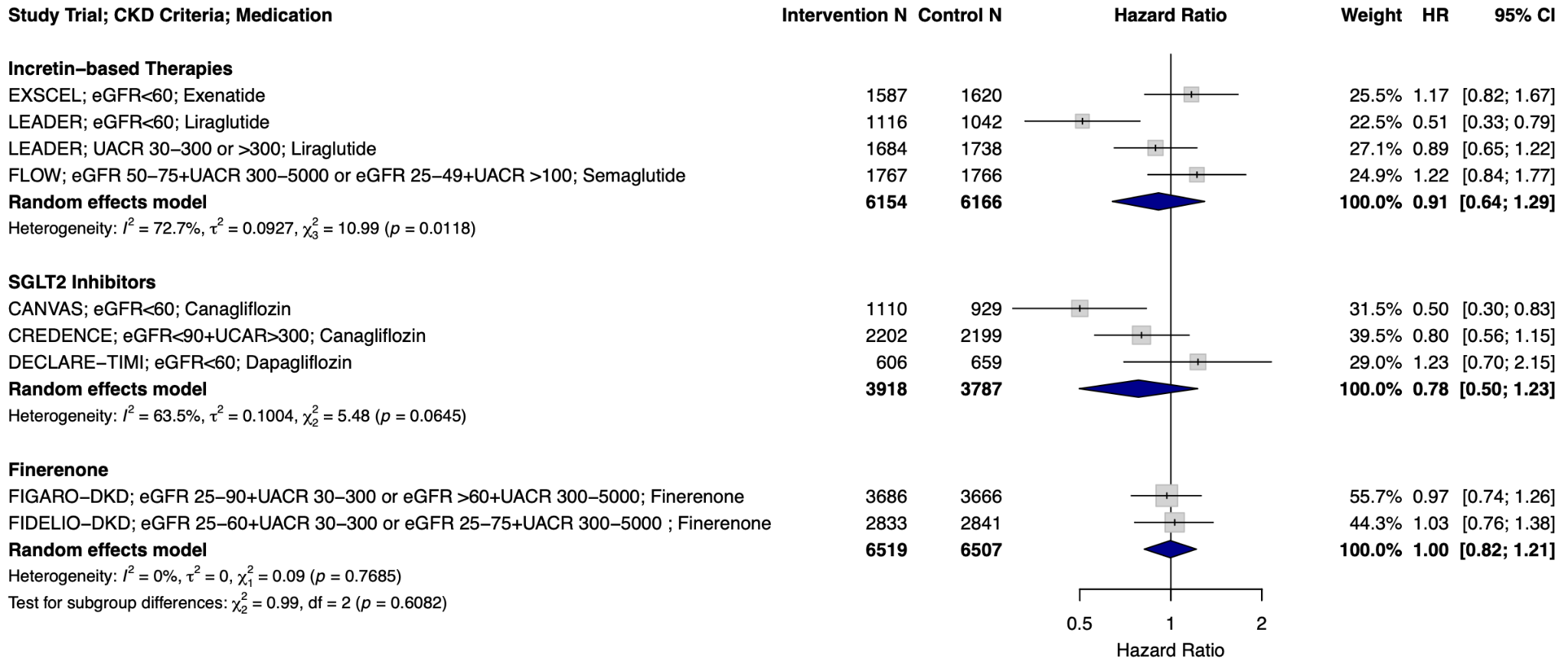


REML–modified HK:

**
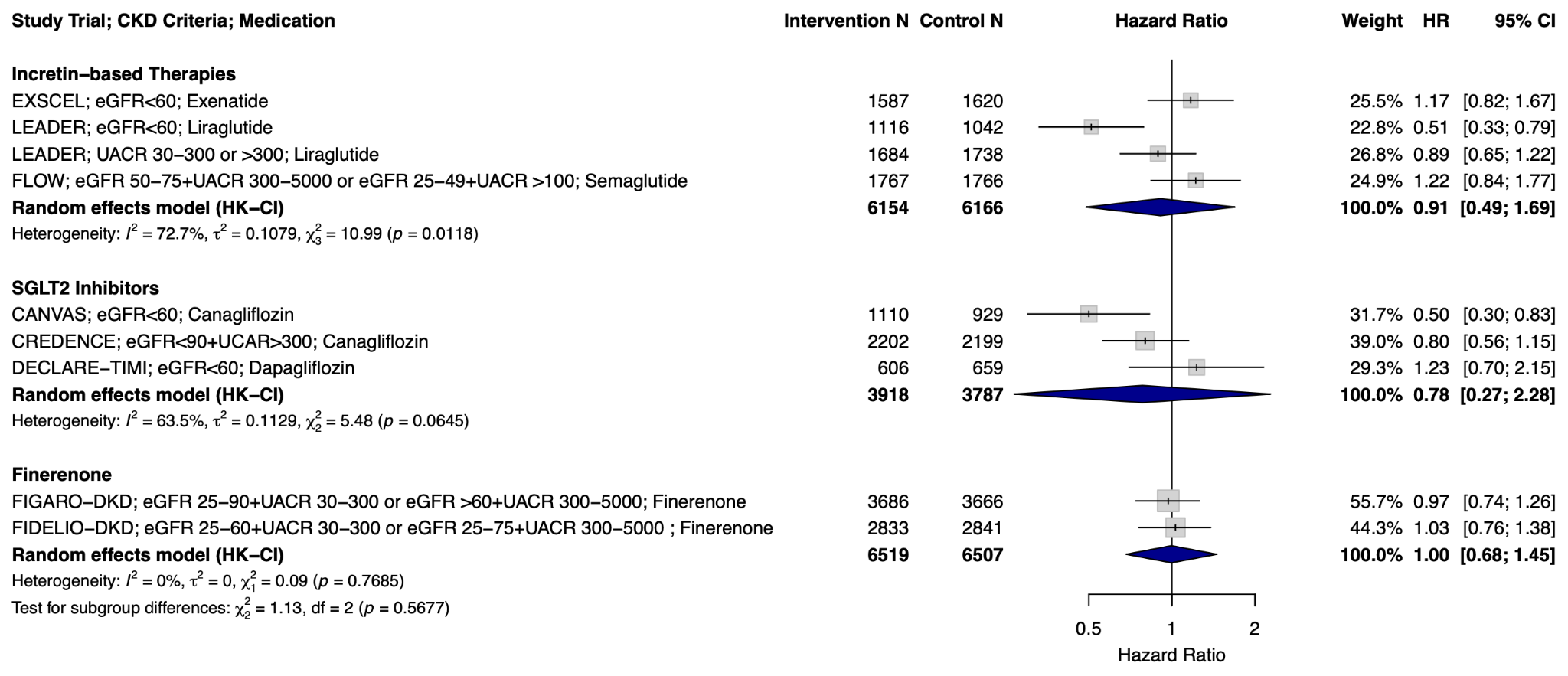
**

REML–Wald:

**
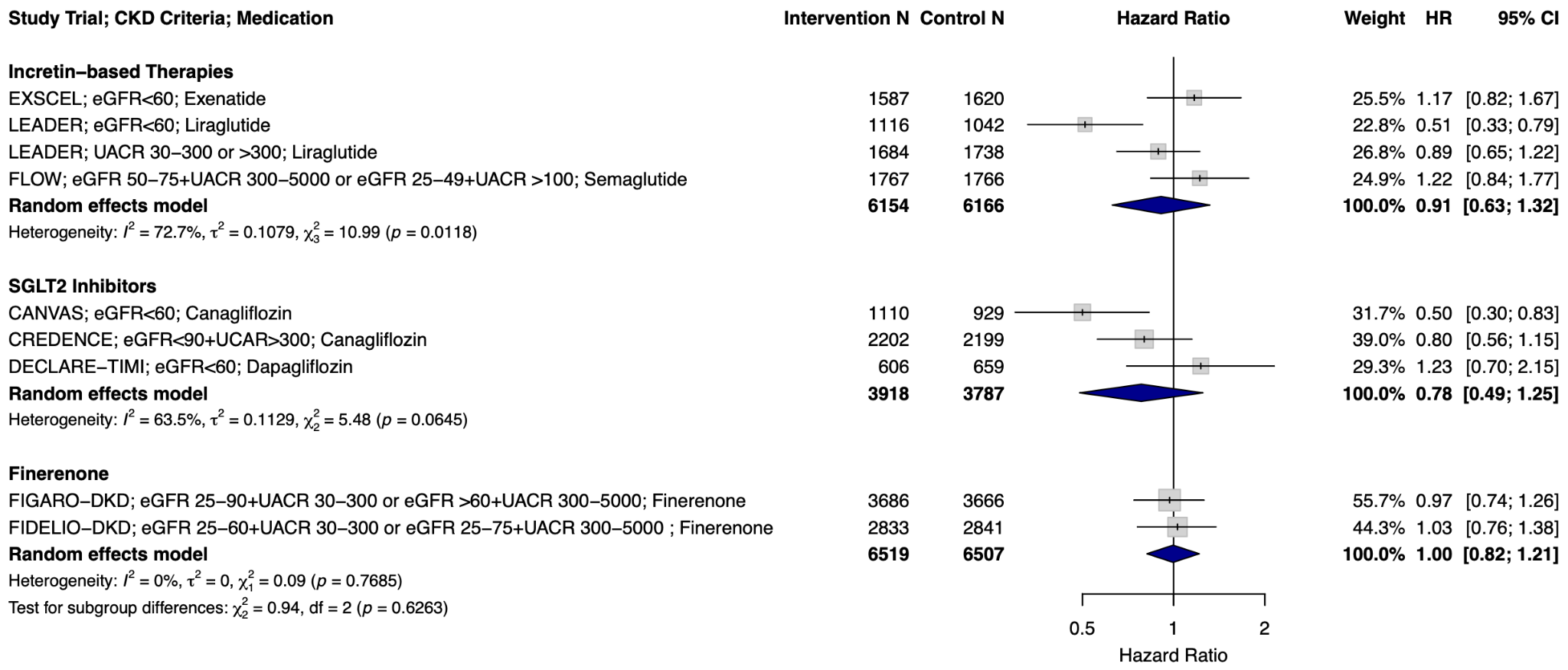
**

**Supplemental Figure S28 – MACE (Chronic Kidney Disease)**

DerSimonian and Laird:


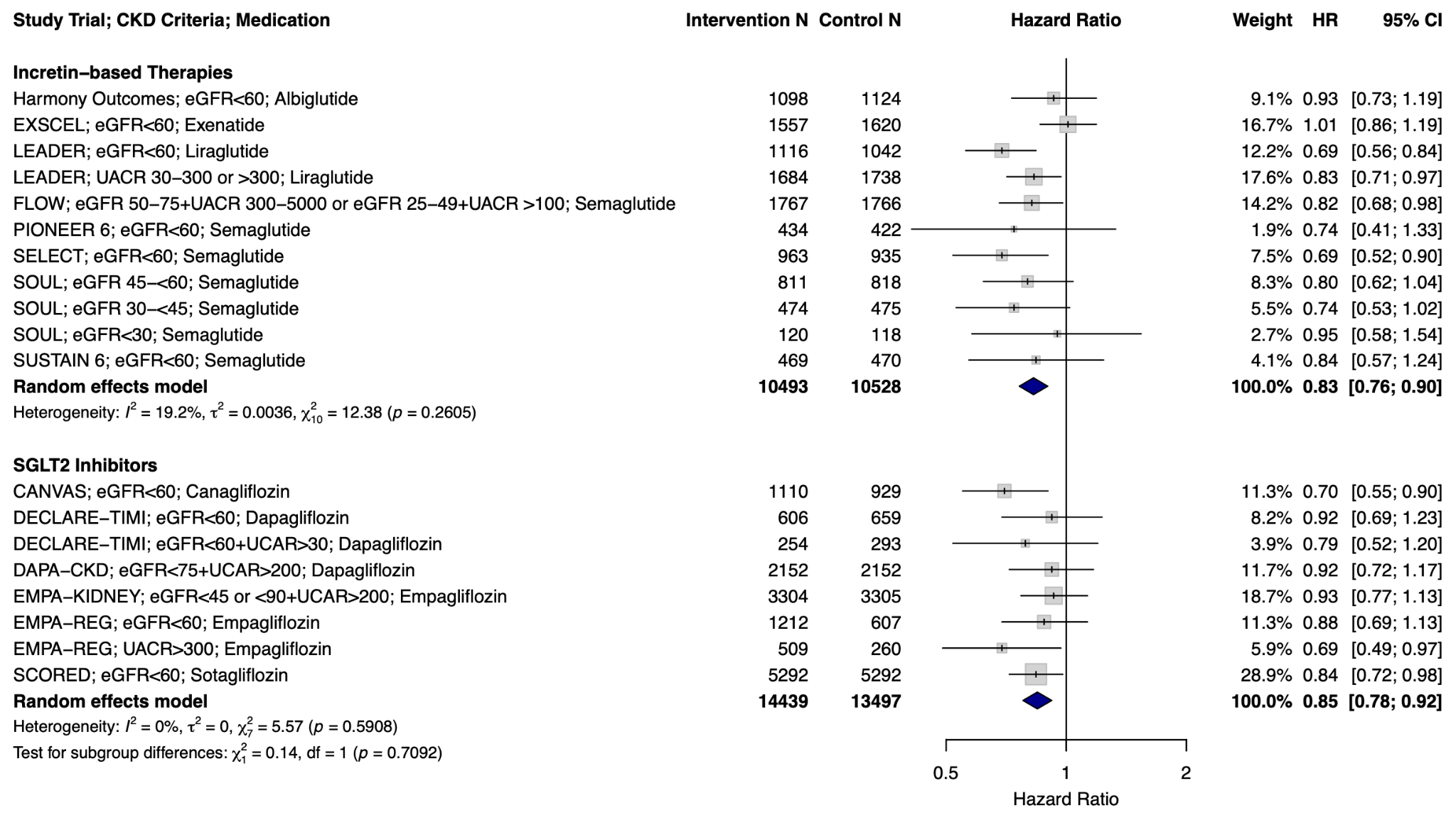


REML–modified HK:

**
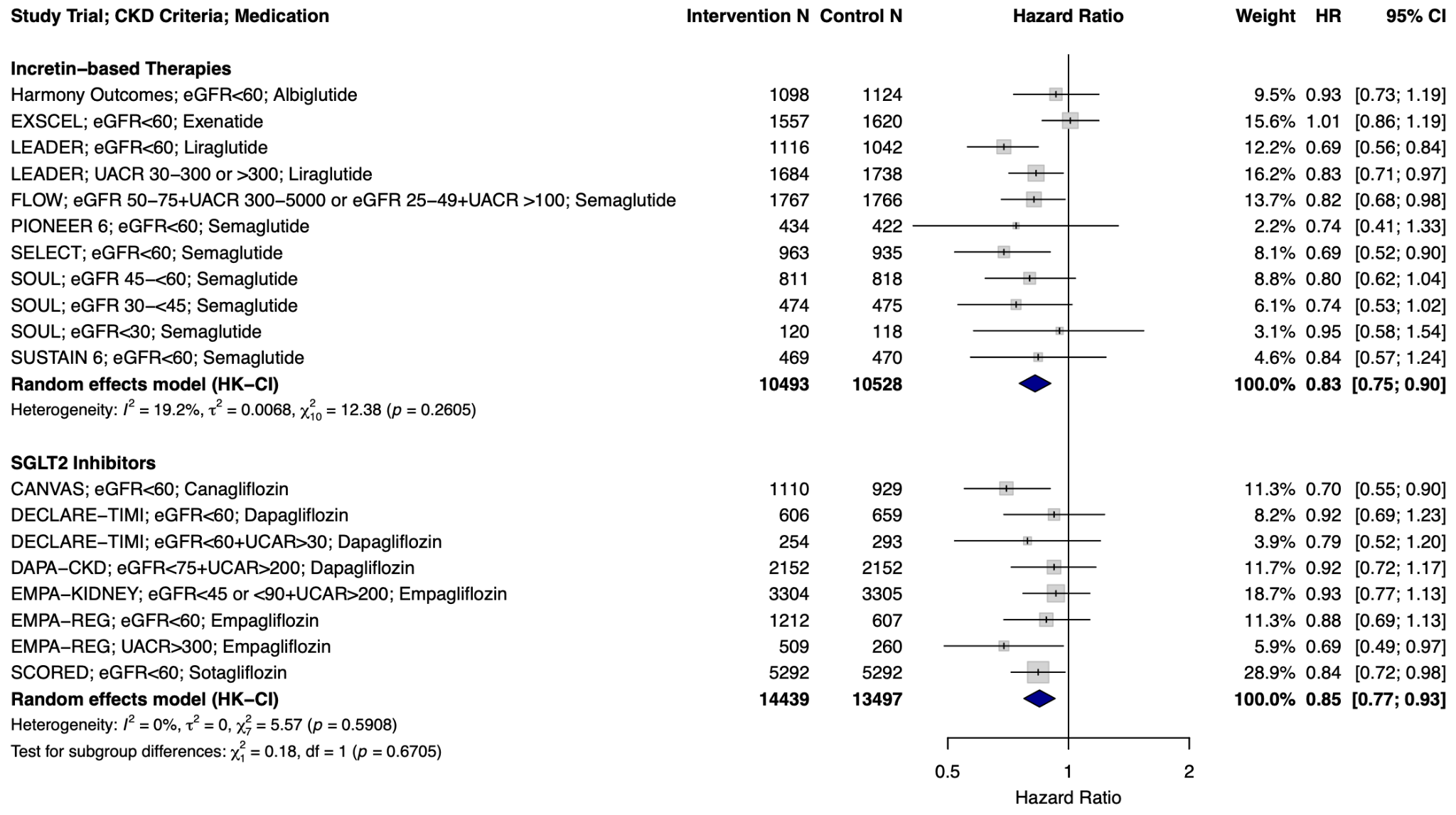
**

REML–Wald:

**
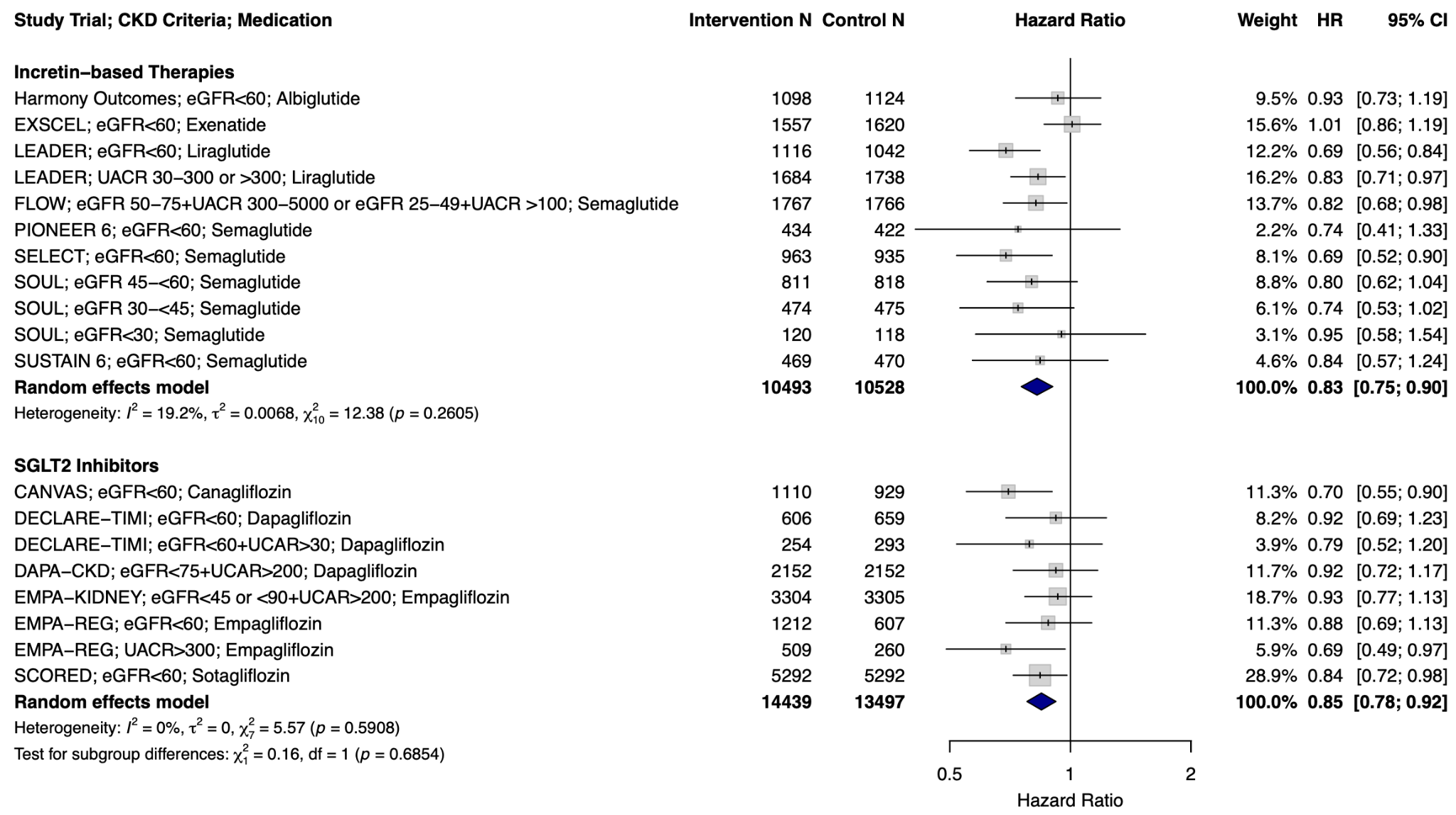
**

**Supplemental Figure S29 – Kidney Composite Outcome (Chronic Kidney Disease)**

DerSimonian and Laird:


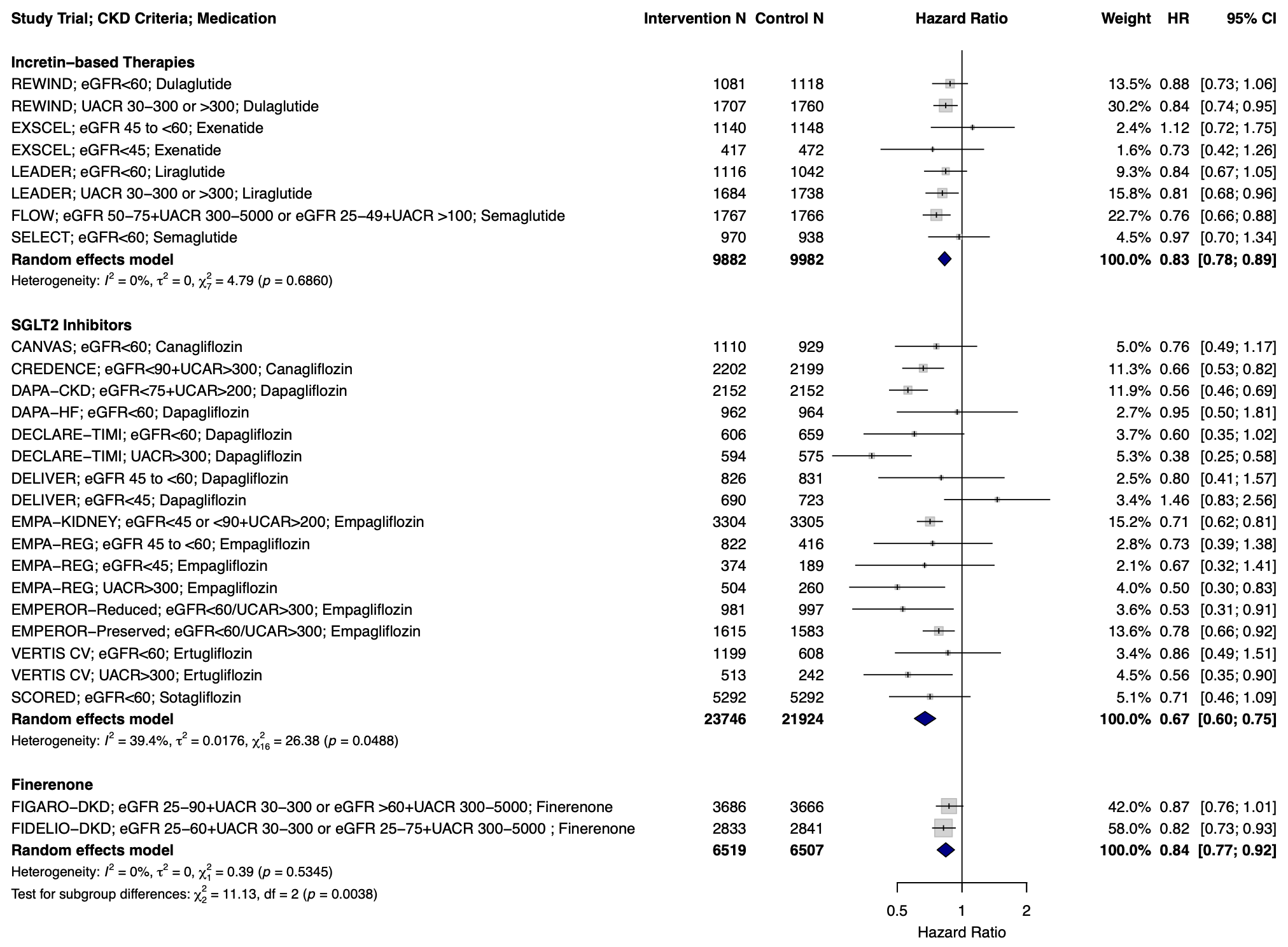


REML–modified HK:

**
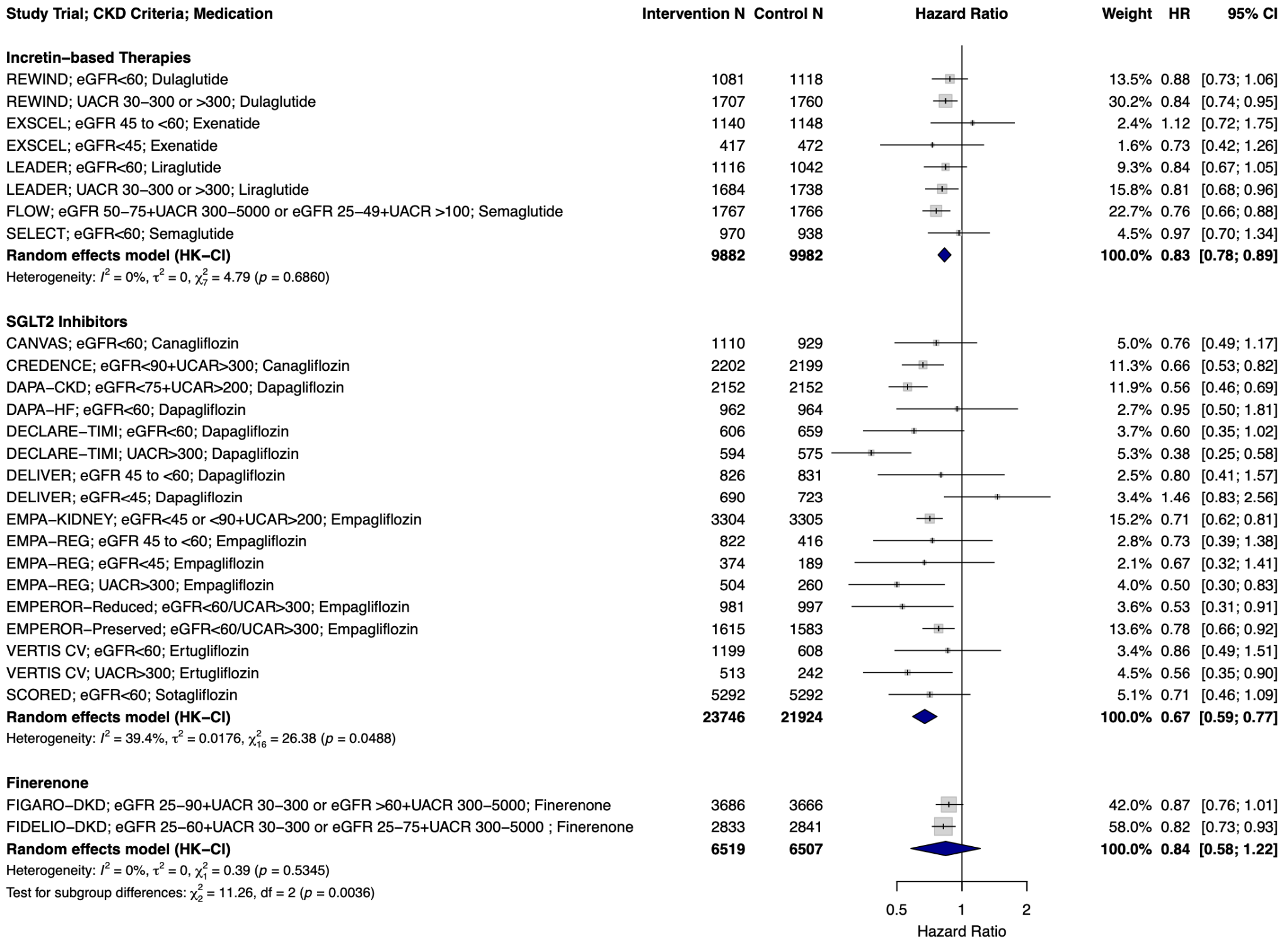
**

REML–Wald:

**
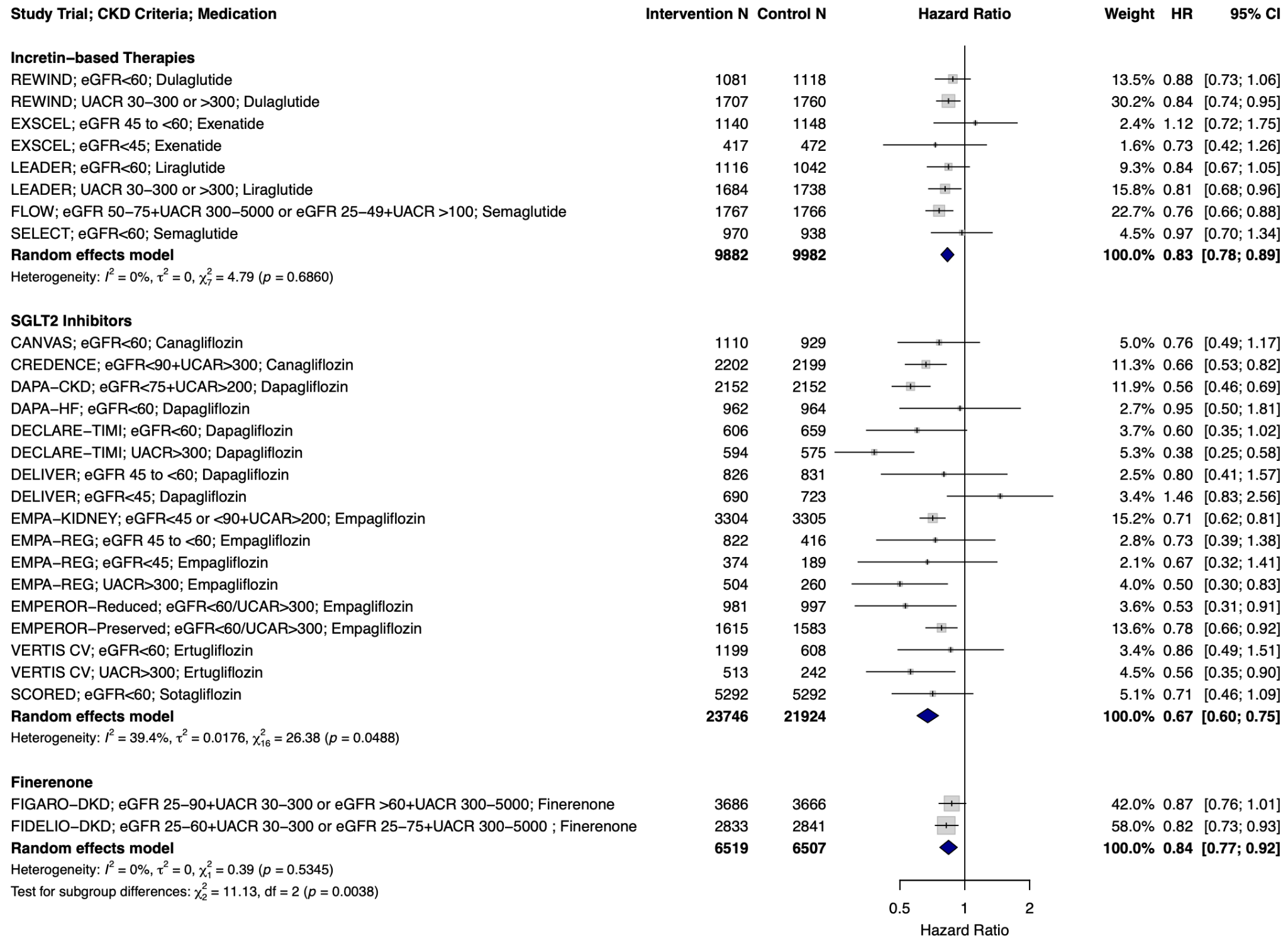
**

###

### **Heart Failure**

**Supplemental Figure S30 – Cardiovascular Mortality (Heart Failure)**

DerSimonian and Laird:


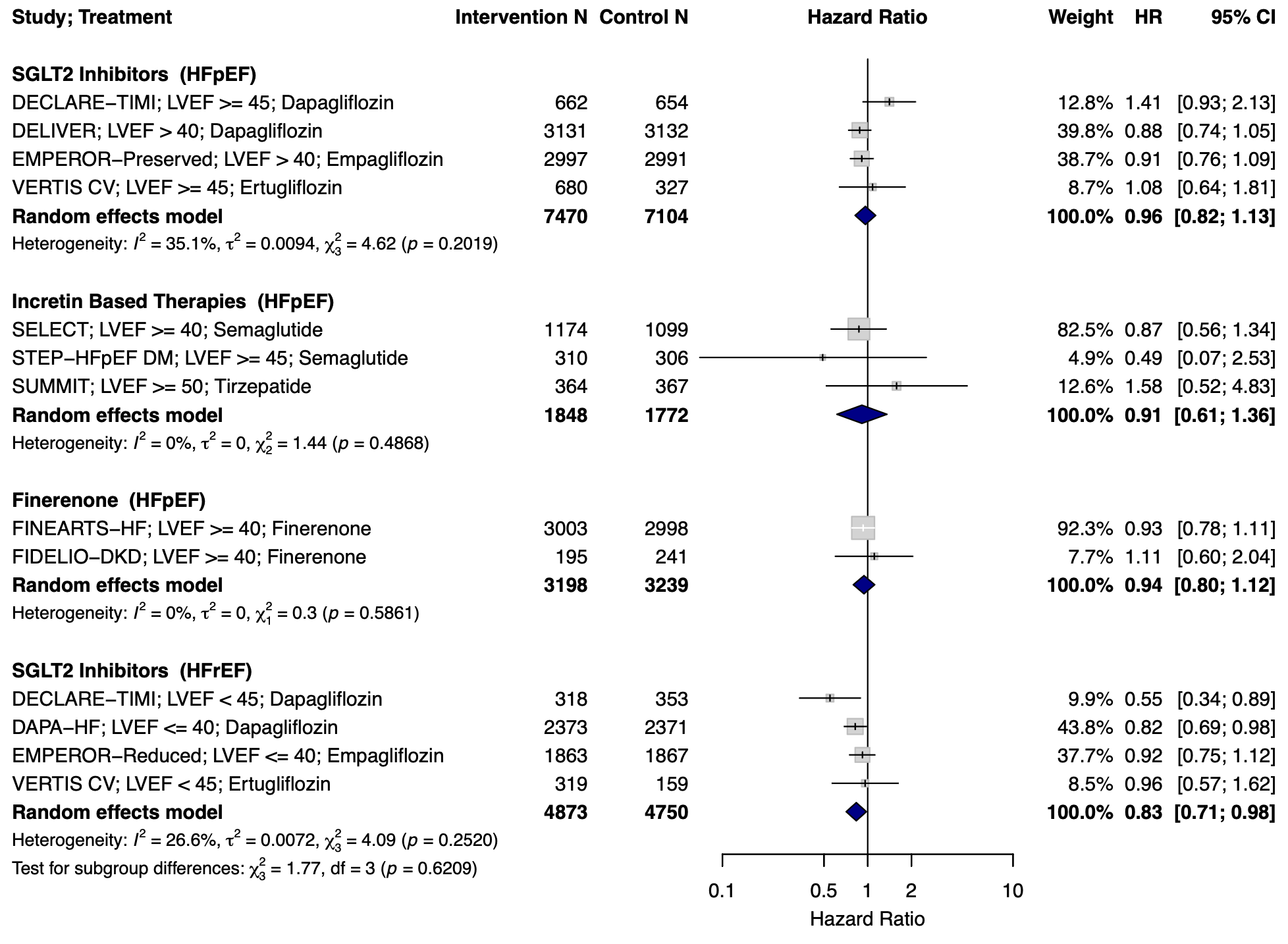


REML–modified HK:

**
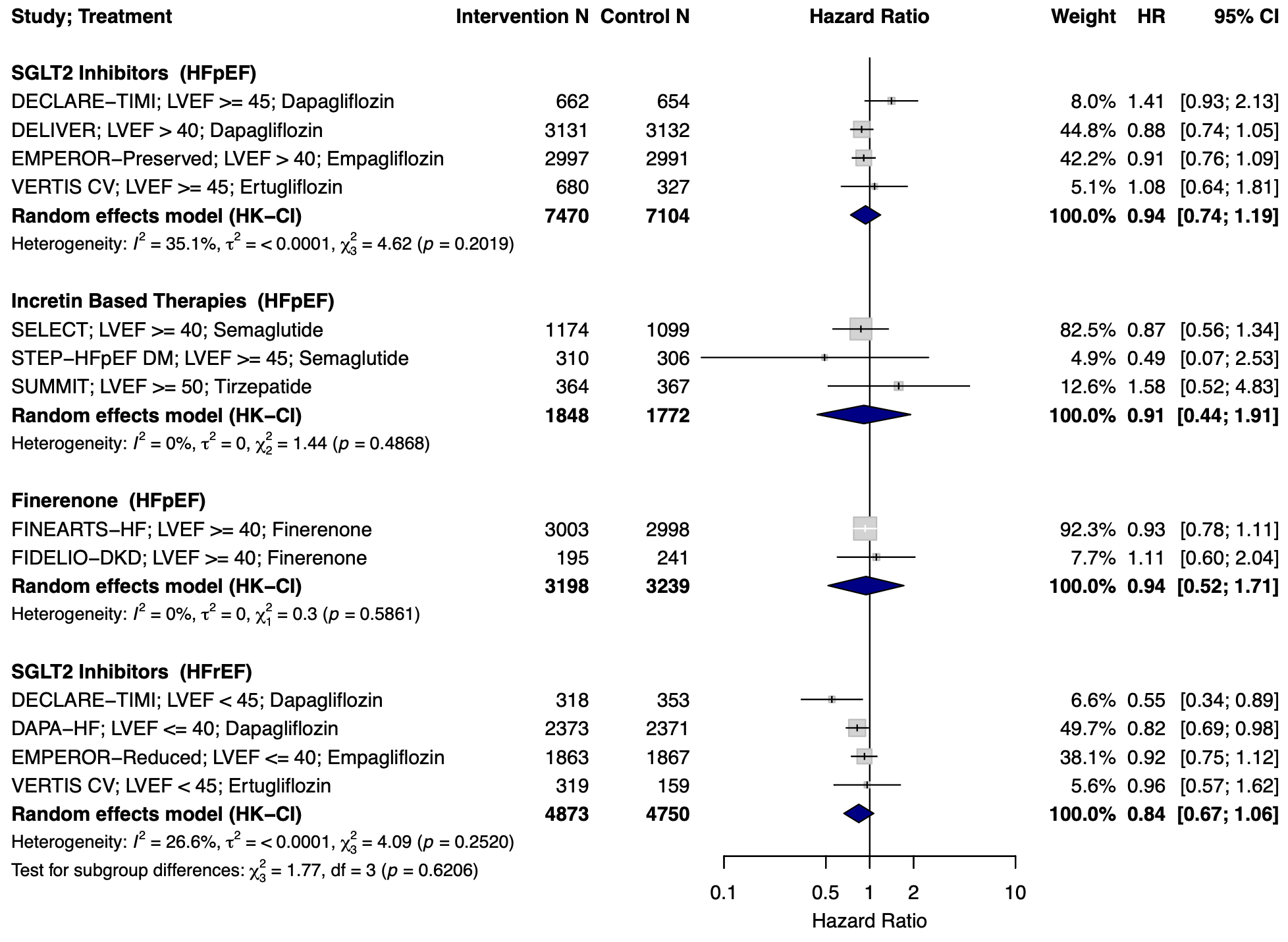
**

REML–Wald:

**
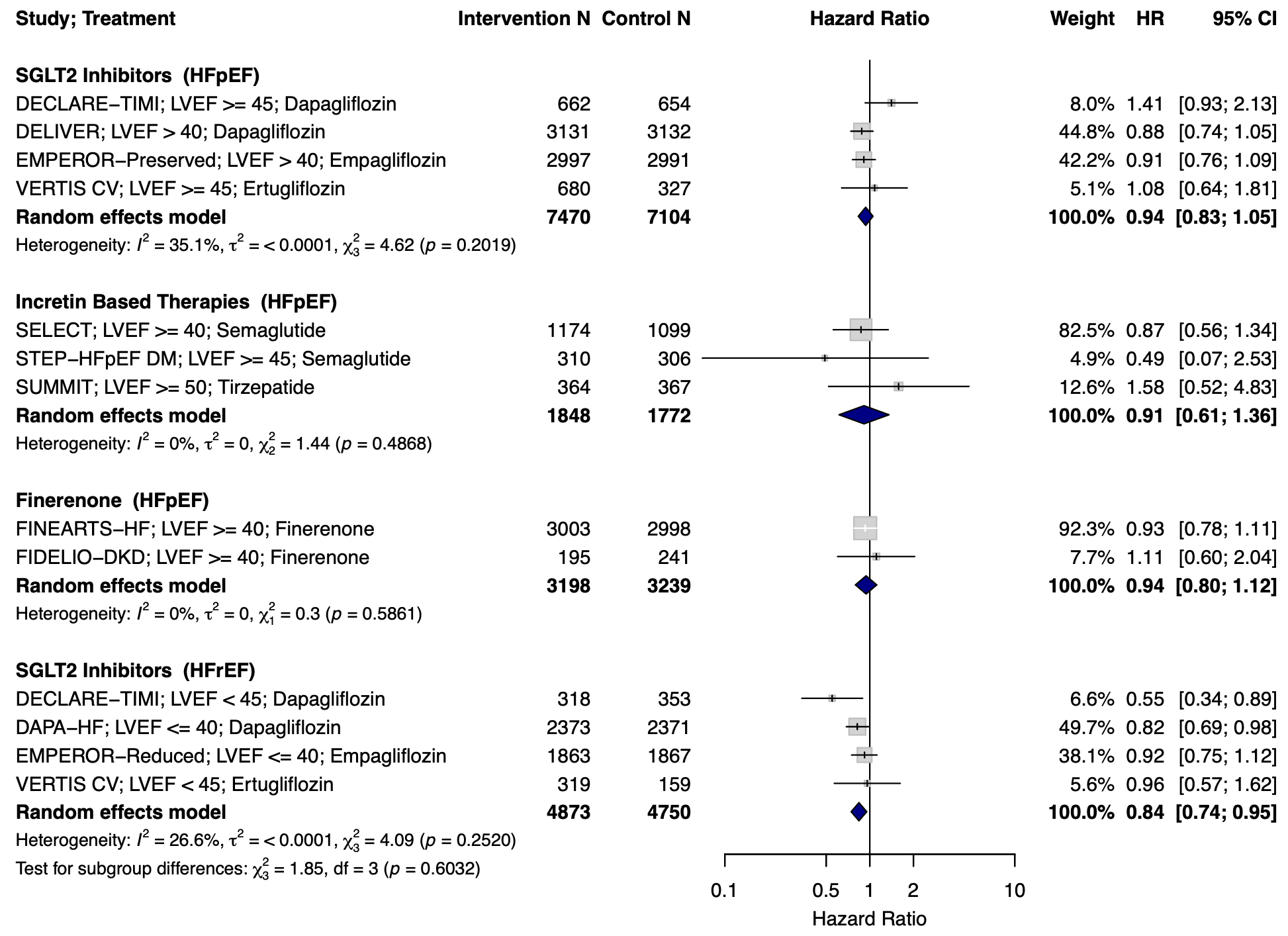
**

**Supplemental Figure S31 – All-Cause Mortality (Heart Failure)**

DerSimonian and Laird:


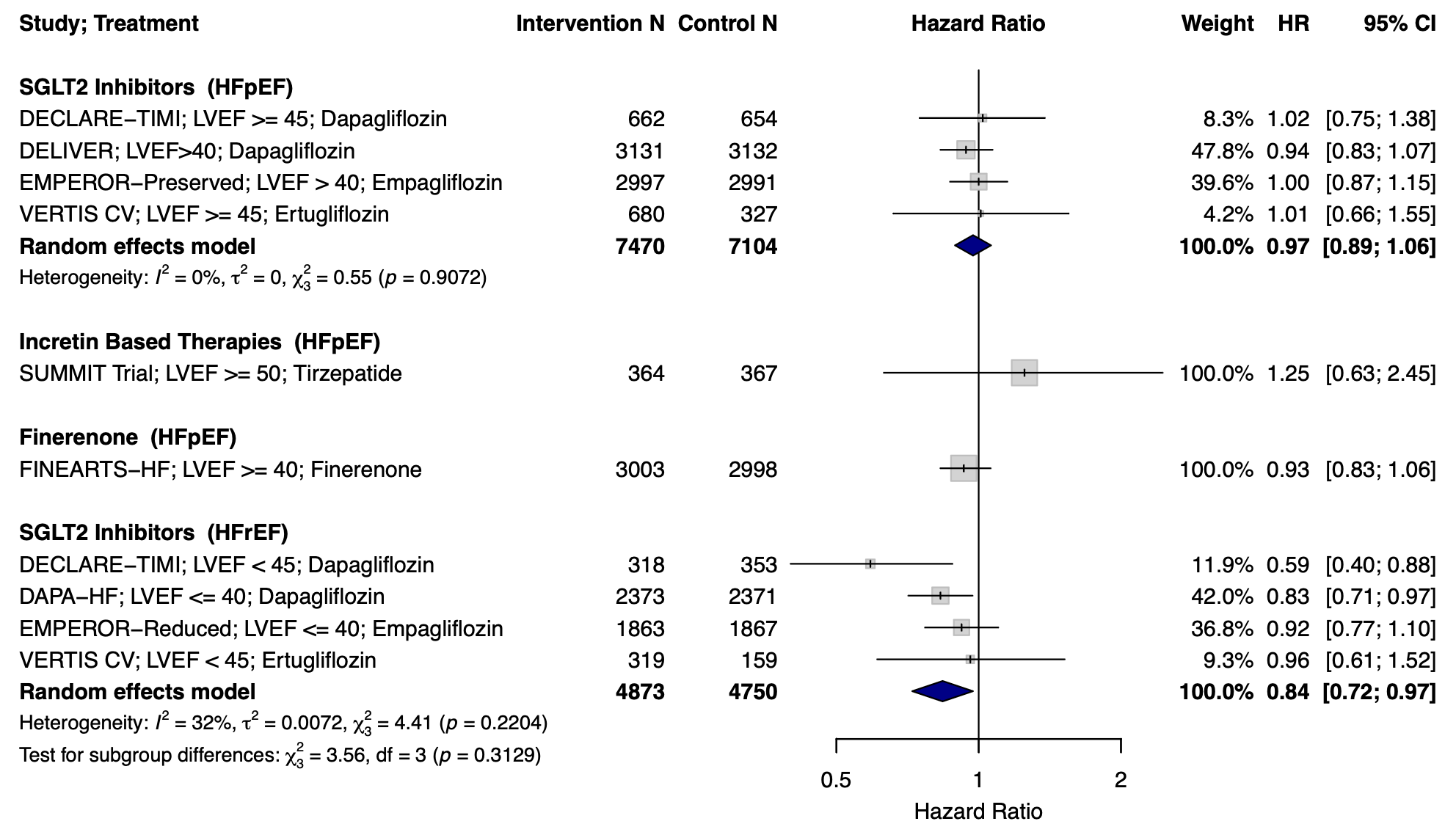


REML–modified HK:

**
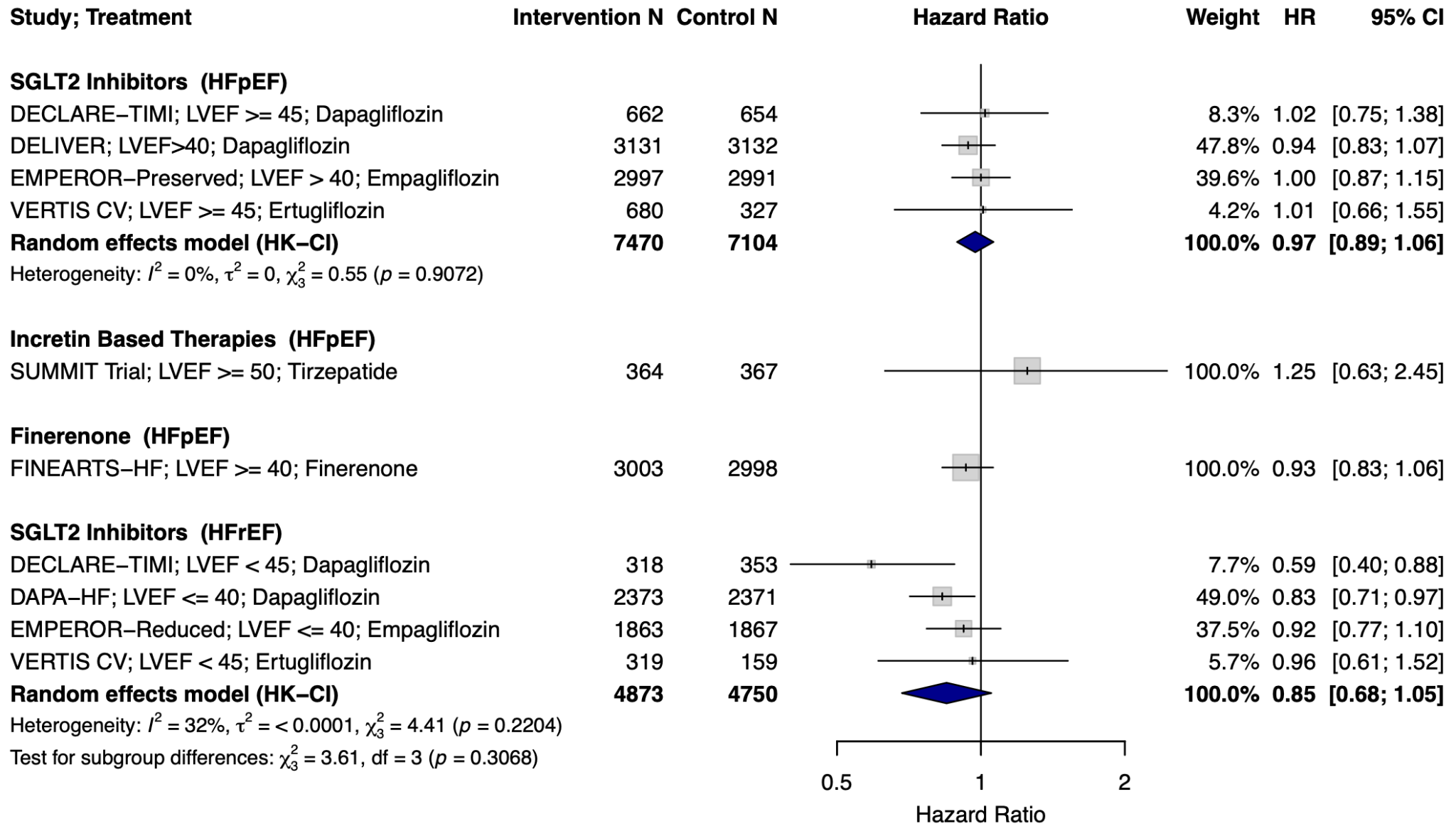
**

REML–Wald:

**
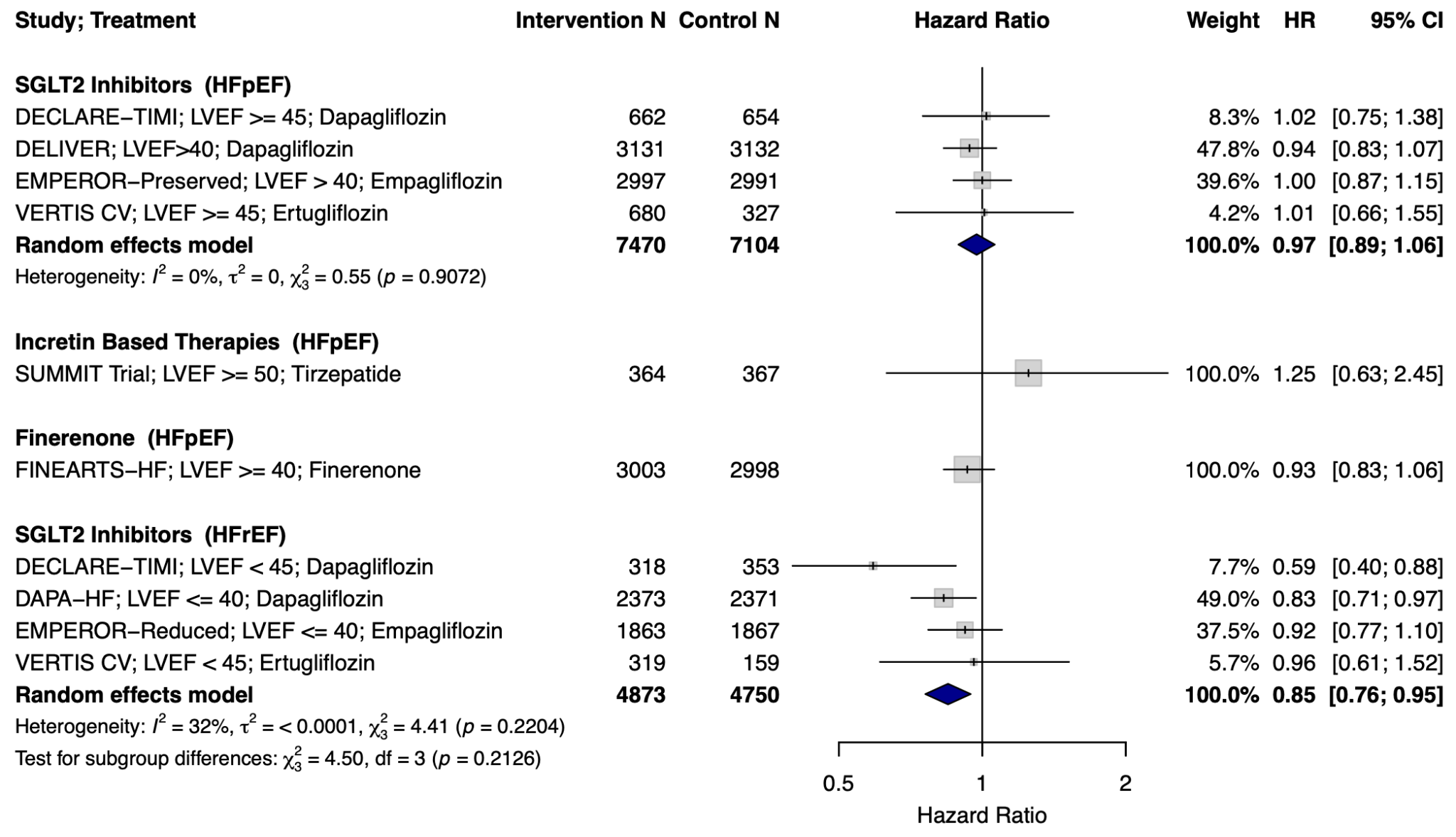
**

**Supplemental Figure S32 – CV Mortality or HF Hospitalization/HF Events (Heart Failure)**

DerSimonian and Laird:


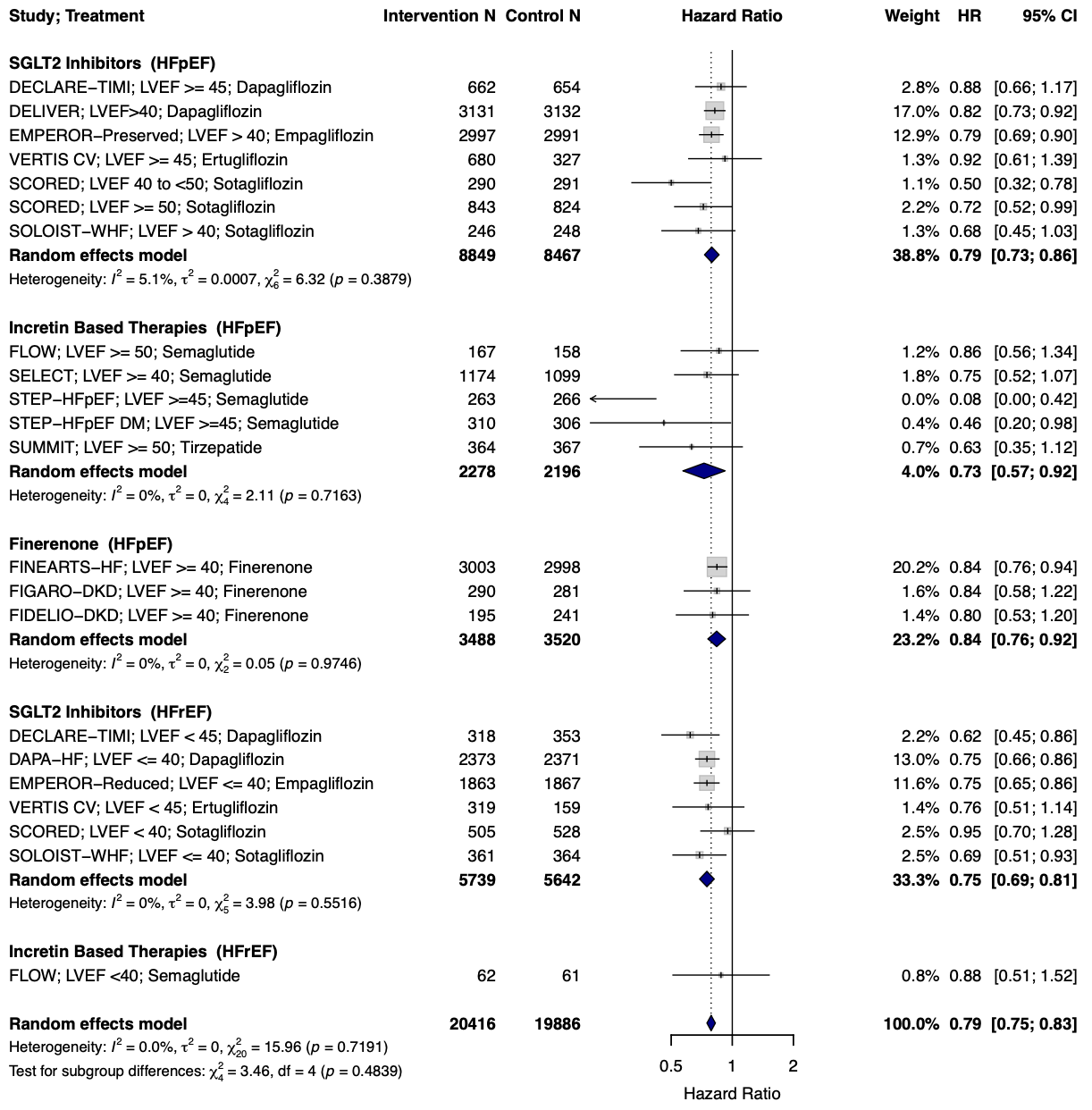


REML–modified HK:


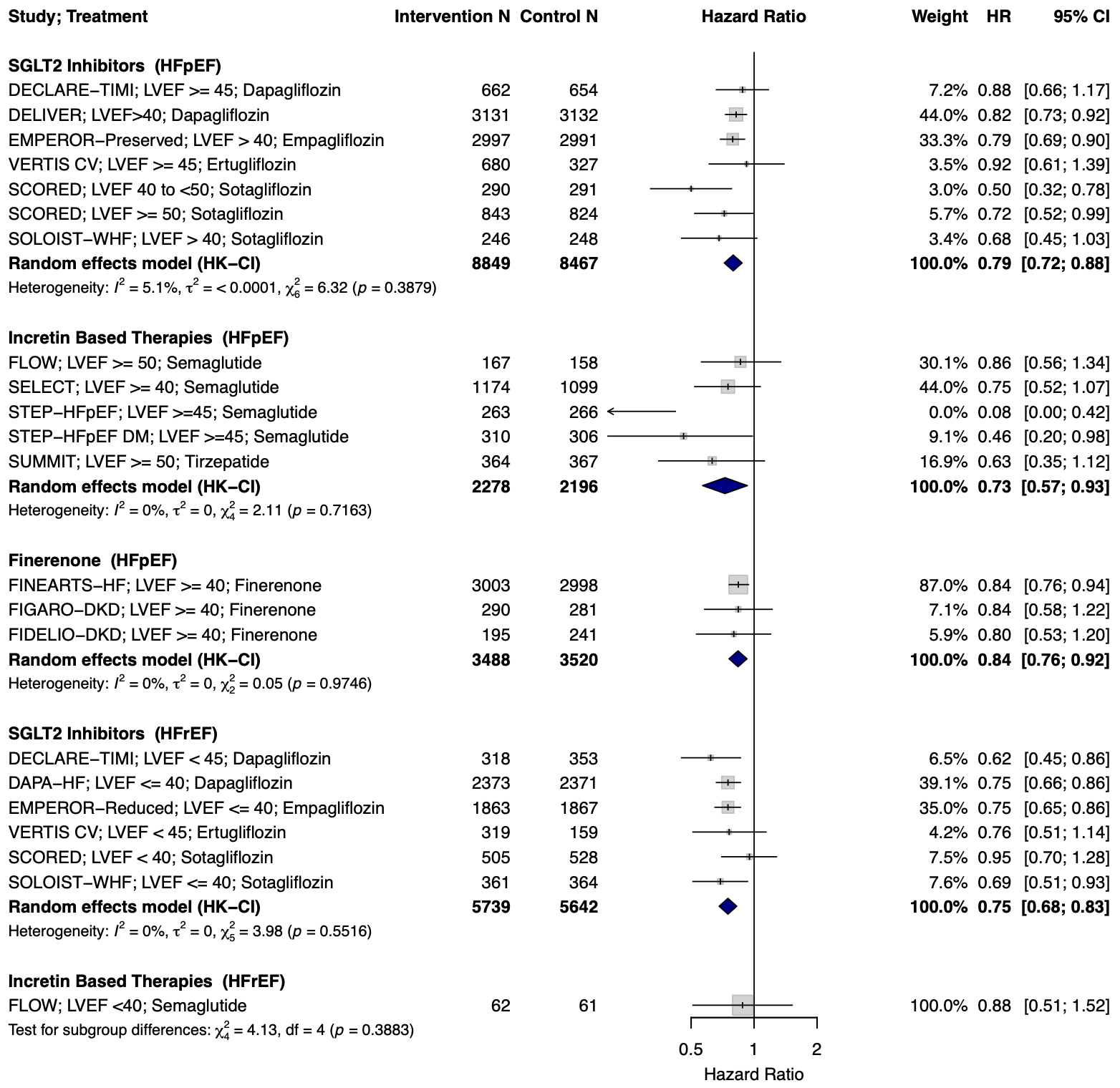


REML–Wald:

**
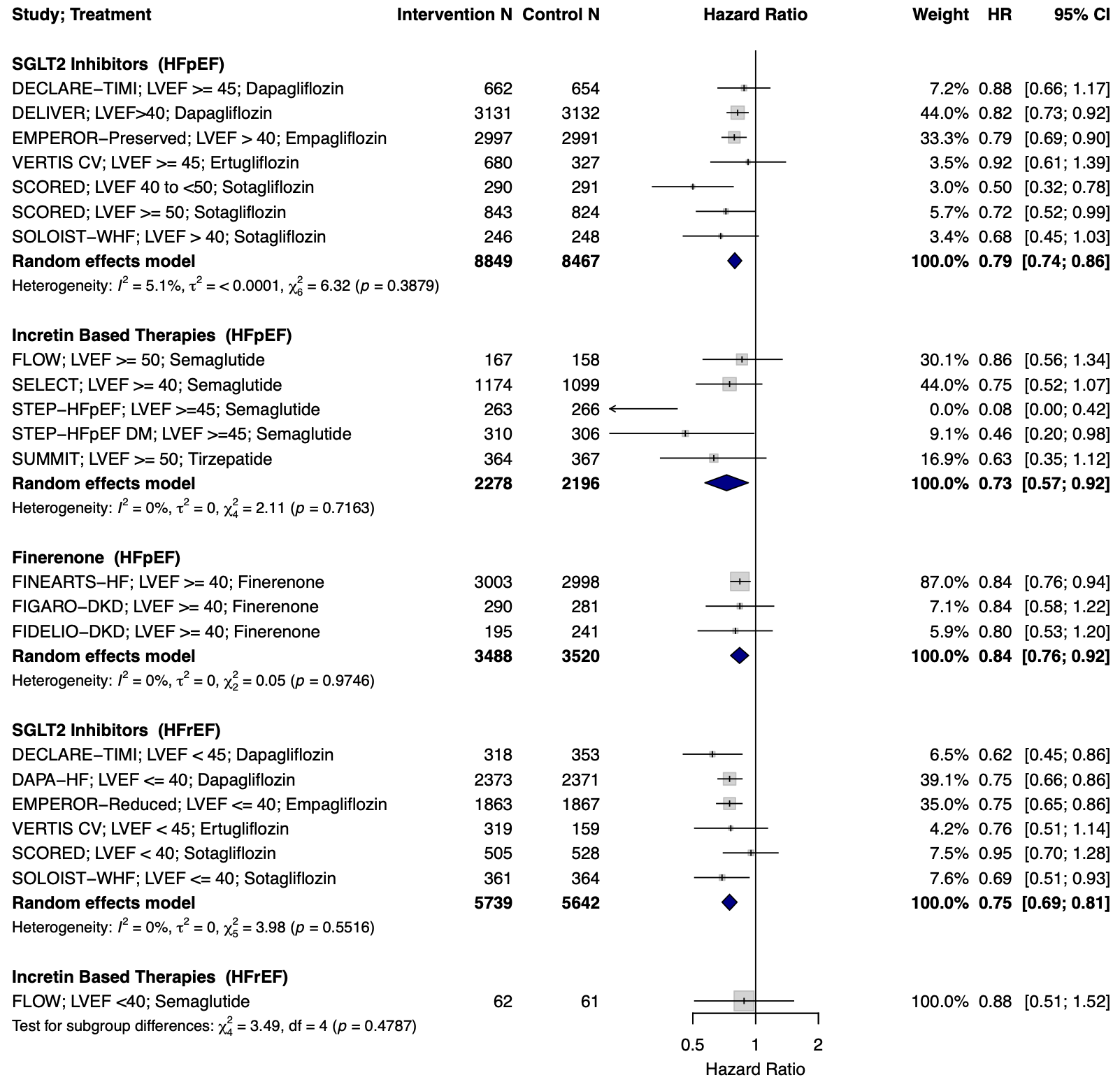
**

**Supplemental Figure S33 – CV Mortality or HF Hospitalization (Heart Failure)**

DerSimonian and Laird:


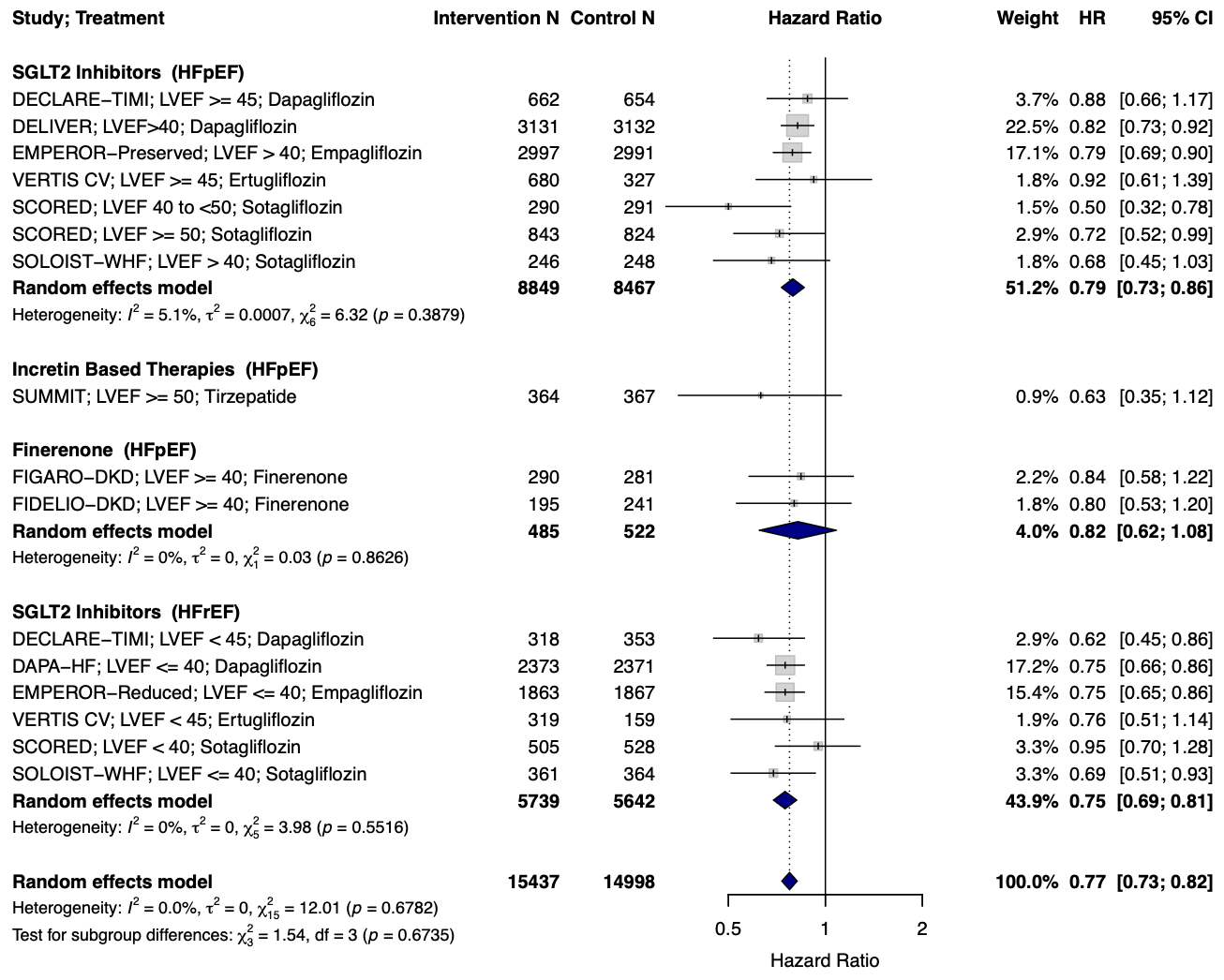


REML–modified HK:


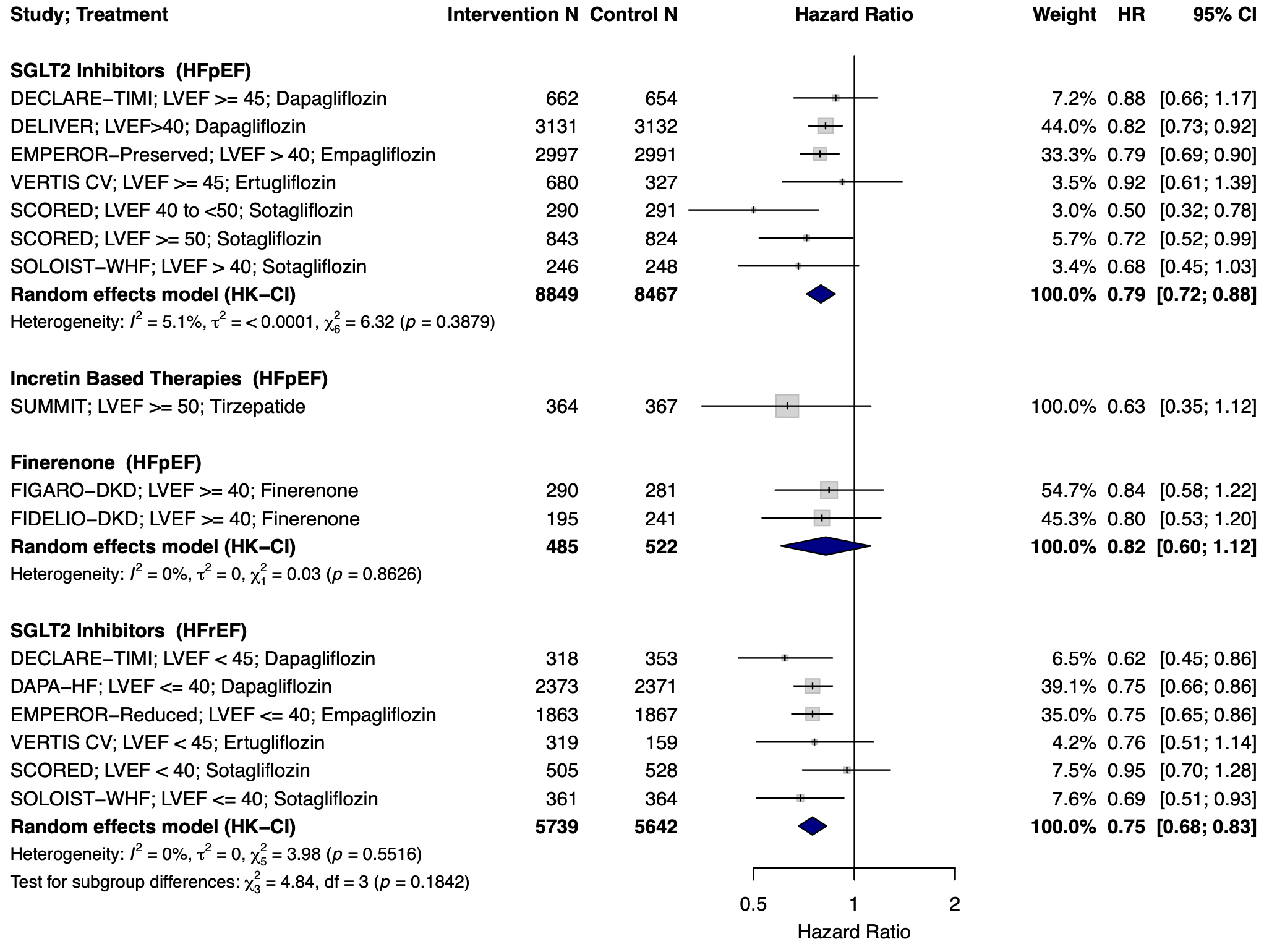


REML–Wald:


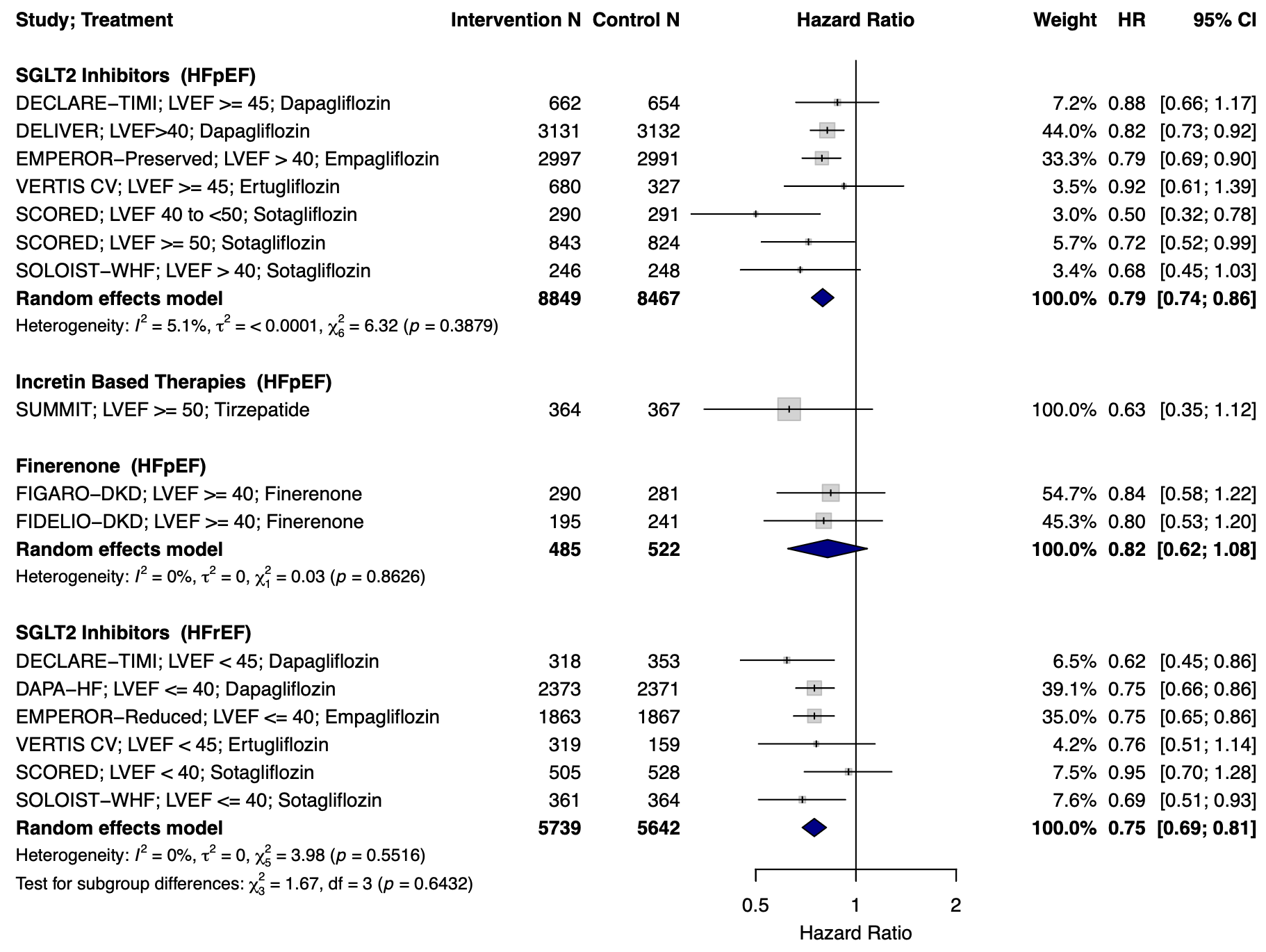


**Supplemental Figure S34 – HF Hospitalization/HF Events (Heart Failure)**

DerSimonian and Laird:


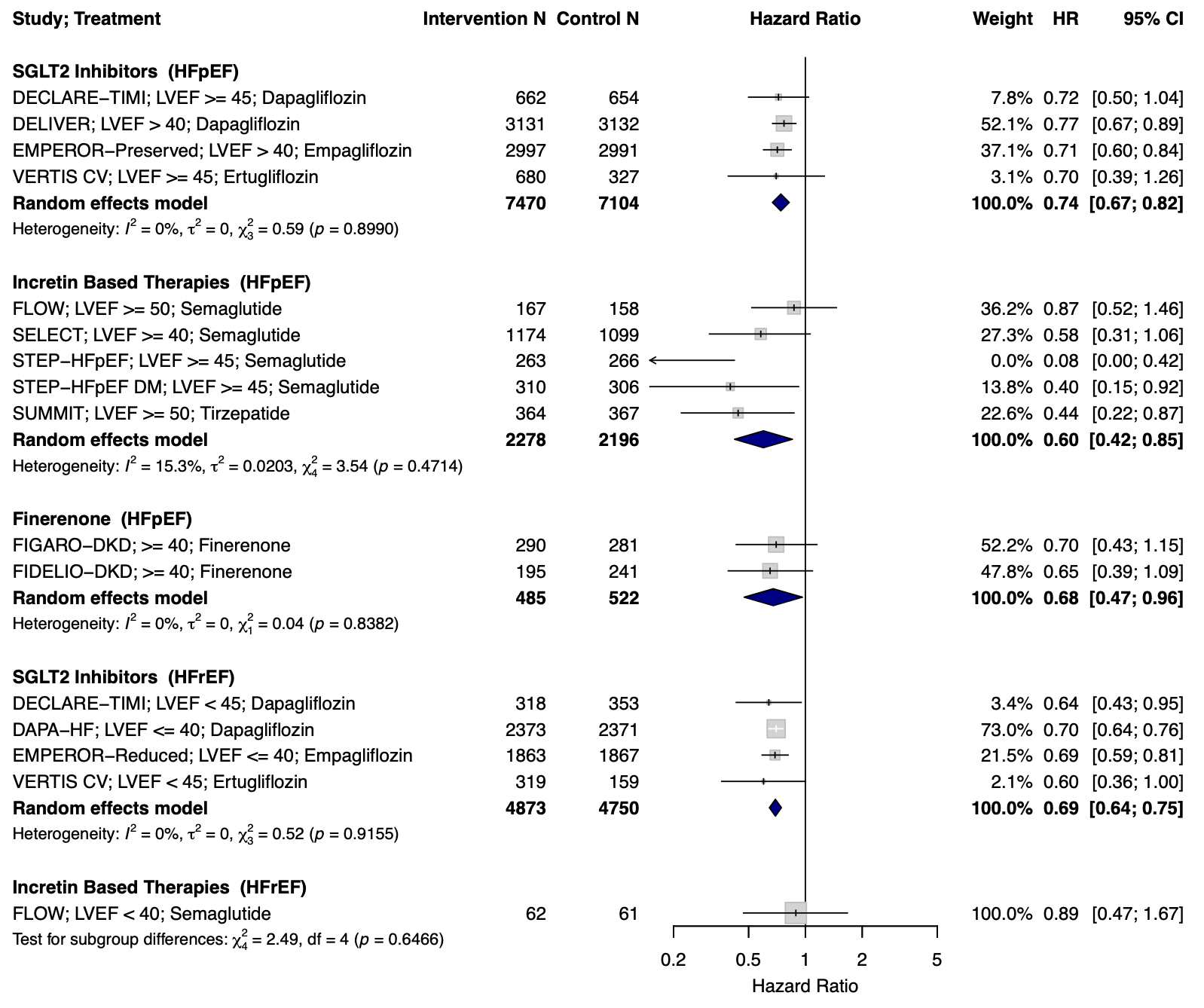


REML–modified HK:

REML–Wald:

**Supplemental Figure S35 – HF Hospitalization (Heart Failure)**

DerSimonian and Laird:

REML–modified HK:

REML–Wald:

**Supplemental Figure S36 – Non-fatal Myocardial Infarction (Heart Failure)**

**Supplemental Figure S37 – Non-fatal Stroke (Heart Failure)**

**Supplemental Figure S38 – MACE (Heart Failure)**

**Supplemental Figure S39 – Kidney Composite Outcome (Heart Failure)**

DerSimonian and Laird:

REML–modified HK:

REML–Wald:

### HFpEF with Obesity

**Supplemental Figure S40 – CV Mortality (HFpEF with Obesity)**

DerSimonian and Laird:

REML–modified HK:

REML–Wald:

**Supplemental Figure S41 – All-Cause Mortality (HFpEF with Obesity)**

**Supplemental Figure S42 – CV Mortality or HF Hospitalization/HF Events (HFpEF with Obesity)**

DerSimonian and Laird:

REML–modified HK:

REML–Wald:

**Supplemental Figure S43 – HF Hospitalization/HF Events (HFpEF with Obesity)**

DerSimonian and Laird:

REML–modified HK:

REML–Wald:

### ASCVD and overweight/obese without T2D

**Supplemental Figure S44 – CV Mortality (ASCVD and overweight/obese without T2D)**

**Supplemental Figure S45 – All-Cause Mortality (ASCVD and overweight/obese without T2D)**

**Supplemental Figure S46 – CV Mortality or HF Hospitalization/HF Events (ASCVD and overweight/obese without T2D)**

**Supplemental Figure S47 – HF Hospitalization/HF Events (ASCVD and overweight/obese without T2D)**

**Supplemental Figure S48 – Non-fatal Myocardial Infarction (ASCVD and overweight/obese without T2D)**

**Supplemental Figure S49 – Non-fatal Stroke (ASCVD and overweight/obese without T2D)**

**Supplemental Figure S50 – MACE (ASCVD and overweight/obese without T2D)**

**Supplemental Figure S51 – Kidney Composite Outcome (ASCVD and overweight/obese without T2D)**

###

### Post-MI

**Supplemental Figure S52 – CV Mortality (Post-MI)**

DerSimonian and Laird:

REML–modified HK:

REML–Wald:

**Supplemental Figure S53 – All-Cause Mortality (Post-MI)**

DerSimonian and Laird:

REML–modified HK:

REML–Wald:

**Supplemental Figure S54 – CV Mortality of HF Hospitalization (Post-MI)**

DerSimonian and Laird:

REML–modified HK:

REML–Wald:

**Supplemental Figure S55 – HF Hospitalization (Post-MI)**

DerSimonian and Laird:

REML–modified HK:

REML–Wald:

**Supplemental Figure S56 – HF Hospitalization (Post-MI with No T2D)**

DerSimonian and Laird:

REML–modified HK:

REML–Wald:

**Supplemental Figure S57 – MACE (Post-MI)**

###

### Acute HF

**Supplemental Figure S58 – CV Mortality (Acute HF)**

**Supplemental Figure S59 – All-Cause Mortality (Acute HF)**

**Supplemental Figure S60 – CV Mortality or HF Hospitalization/Events (Acute HF)**

DerSimonian and Laird:

REML–modified HK:

REML–Wald:

**Supplemental Figure S61 – HF Hospitalization (Acute HF)**

**Supplemental Figure S62 – MACE (Acute HF)**

### **Network Meta-Analyses**

**Supplemental Figure S63 – Cardiovascular Mortality (T2DM with ASCVD / high CVD risk) NMA**

Indirect comparison: SGLT2 Inhibitors vs Incretin-based Therapies:
HR 1.00 (95% CI 0.91 – 1.10)

Indirect comparison: SGLT2 Inhibitors vs Finerenone:
HR 0.96 (95% CI 0.84 – 1.10)

Indirect comparison: Incretin-based Therapies vs Finerenone:
HR 0.96 (95% CI 0.84 – 1.10)

**Supplemental Figure S64 – All-Cause Mortality (T2DM with ASCVD / high CVD risk) NMA**

Indirect comparison: SGLT2 Inhibitors vs Incretin-based Therapies
HR 1.01 (95% CI 0.94 – 1.09)

Indirect comparison: SGLT2 Inhibitors vs Finerenone
HR 1.00 (95% CI 0.85 – 1.18)

Indirect comparison: Incretin-based Therapies vs Finerenone
HR 0.99 (95% CI 0.85 – 1.17)

**Supplemental Figure S65 – CV Mortality or HF Hospitalization/HF Events (T2DM with ASCVD / high CVD risk) NMA**

Indirect comparison: SGLT2 Inhibitors vs Incretin-based Therapies
HR 0.86 (95% CI 0.78 – 0.95)

Indirect comparison: SGLT2 Inhibitors vs Finerenone
HR 0.92 (95% CI 0.78 – 1.08)

Indirect comparison: Incretin-based Therapies vs Finerenone
HR 1.07 (95% CI 0.90 – 1.27)

**Supplemental Figure S66 – CV Mortality or HF Hospitalization (T2DM with ASCVD / high CVD risk) NMA**

Indirect comparison: SGLT2 Inhibitors vs Incretin-based Therapies
HR 0.86 (95% CI 0.77 – 0.96)

Indirect comparison: SGLT2 Inhibitors vs Finerenone
HR 0.92 (95% CI 0.78 – 1.08)

Indirect comparison: Incretin-based Therapies vs Finerenone
HR 1.07 (95% CI 0.89 – 1.29)

**Supplemental Figure S67 – HF Hospitalization/Event (T2DM with ASCVD / high CVD risk) NMA**

Indirect comparison: SGLT2 Inhibitors vs Incretin-based Therapies
HR 0.78 (95% CI 0.70 – 0.87)

Indirect comparison: SGLT2 Inhibitors vs Finerenone
HR 0.90 (95% CI 0.75 – 1.07)

Indirect comparison: Incretin-based Therapies vs Finerenone
HR 1.15 (95% CI 0.95 – 1.38)

**Supplemental Figure S68 – HF Hospitalization (T2DM with ASCVD / high CVD risk) NMA**

Indirect comparison: SGLT2 Inhibitors vs Incretin-based Therapies
HR 0.77 (95% CI 0.69 – 0.86)

Indirect comparison: SGLT2 Inhibitors vs Finerenone
HR 0.90 (95% CI 0.75 – 1.07)

Indirect comparison: Incretin-based Therapies vs Finerenone
HR 1.17 (95% CI 0.97 – 1.41)

**Supplemental Figure S69 – Non-fatal Myocardial Infarction (T2DM with ASCVD / high CVD risk) NMA**

Indirect comparison: SGLT2 Inhibitors vs Incretin-based Therapies
HR 1.00 (95% CI 0.88 – 1.13)

Indirect comparison: SGLT2 Inhibitors vs Finerenone
HR 1.00 (95% CI 0.78 – 1.27)

Indirect comparison: Incretin-based Therapies vs Finerenone
HR 1.00 (95% CI 0.79 – 1.26)

**Supplemental Figure S70 – Non-fatal Stroke (T2DM with ASCVD / high CVD risk) NMA**

Indirect comparison: SGLT2 Inhibitors vs Incretin-based Therapies
HR 1.14 (95% CI 0.99 – 1.32)

Indirect comparison: SGLT2 Inhibitors vs Finerenone
HR 0.99 (95% CI 0.79 – 1.25)

Indirect comparison: Incretin-based Therapies vs Finerenone
HR 0.87 (95% CI 0.70 – 1.08)

**Supplemental Figure S71 – MACE (T2DM with ASCVD / high CVD risk) NMA**

Indirect comparison: SGLT2 Inhibitors vs Incretin-based Therapies
HR 1.01 (95% CI 0.94 – 1.10)

**Supplemental Figure S72 – Kidney Composite Outcome (T2DM with ASCVD / high CVD risk) NMA**

Indirect comparison: SGLT2 Inhibitors vs Incretin-based Therapies
HR 0.84 (95% CI 0.73 – 0.96)

Indirect comparison: SGLT2 Inhibitors vs Finerenone
HR 0.78 (95% CI 0.65 – 0.94)

Indirect comparison: Incretin-based Therapies vs Finerenone
HR 0.93 (95% CI 0.78 – 1.11)

**Supplemental Figure S73 – Cardiovascular Mortality (Chronic Kidney Disease) NMA**

Indirect comparison: SGLT2 Inhibitors vs Incretin-based Therapies
HR 1.08 (95% CI 0.93 – 1.26)

Indirect comparison: SGLT2 Inhibitors vs Finerenone
HR 0.97 (95% CI 0.81 – 1.15)

Indirect comparison: Incretin-based Therapies vs Finerenone
HR 0.89 (95% CI 0.73 – 1.08)

**Supplemental Figure S74 – All-Cause Mortality (Chronic Kidney Disease) NMA**

Indirect comparison: SGLT2 Inhibitors vs Incretin-based Therapies
HR 0.99 (95% CI 0.87 – 1.14)

Indirect comparison: SGLT2 Inhibitors vs Finerenone
HR 0.92 (95% CI 0.79 – 1.08)

Indirect comparison: Incretin-based Therapies vs Finerenone
HR 0.93 (95% CI 0.78 – 1.10)

###

**Supplemental Figure S75 – CV Mortality or HF Hospitalization/HF Events (Chronic Kidney Disease) NMA**

Indirect comparison: SGLT2 Inhibitors vs Incretin-based Therapies

HR 1.23 (95% CI 0.72 – 2.10)

Indirect comparison: SGLT2 Inhibitors vs Finerenone

HR 0.90 (95% CI 0.76 – 1.06)

Indirect comparison: Incretin-based Therapies vs Finerenone

HR 0.73 (95% CI 0.42 – 1.28)

**Supplemental Figure S76 – CV Mortality or HF Hospitalization (Chronic Kidney Disease) NMA**

Indirect comparison: SGLT2 Inhibitors vs Finerenone

HR 0.96 (95% CI 0.84 – 1.10)

**Supplemental Figure S77 – HF Hospitalization (Chronic Kidney Disease) NMA**

Indirect comparison: SGLT2 Inhibitors vs Incretin-based Therapies

HR 0.72 (95% CI 0.58 – 0.90)

Indirect comparison: SGLT2 Inhibitors vs Finerenone

HR 0.84 (95% CI 0.67 – 1.07)

Indirect comparison: Incretin-based Therapies vs Finerenone

HR 1.17 (95% CI 0.88 – 1.56)

**Supplemental Figure S78 – Non-Fatal Myocardial Infarction (Chronic Kidney Disease) NMA**

Indirect comparison: SGLT2 Inhibitors vs Incretin-based Therapies

HR 0.91 (95% CI 0.71 – 1.16)

Indirect comparison: SGLT2 Inhibitors vs Finerenone

HR 0.85 (95% CI 0.63 – 1.14)

Indirect comparison: Incretin-based Therapies vs Finerenone

HR 0.93 (95% CI 0.73 – 1.19)

**Supplemental Figure S79 – Non-Fatal Stroke (Chronic Kidney Disease) NMA**

Indirect comparison: SGLT2 Inhibitors vs Incretin-based Therapies

HR 0.86 (95% CI 0.51 – 1.45)

Indirect comparison: SGLT2 Inhibitors vs Finerenone

HR 0.78 (95% CI 0.43 – 1.42)

Indirect comparison: Incretin-based Therapies vs Finerenone

HR 0.91 (95% CI 0.53 – 1.57)

**Supplemental Figure S80 – MACE (Chronic Kidney Disease) NMA**

###

Indirect comparison: SGLT2 Inhibitors vs Incretin-based Therapies

HR 1.02 (95% CI 0.90 – 1.16)

**Supplemental Figure S81 – Kidney Composite Outcome (Chronic Kidney Disease) NMA**

###

Indirect comparison: SGLT2 Inhibitors vs Incretin-based Therapies

HR 0.82 (95% CI 0.73 – 0.91)

Indirect comparison: SGLT2 Inhibitors vs Finerenone

HR 0.81 (95% CI 0.71 – 0.92)

Indirect comparison: Incretin-based Therapies vs Finerenone

HR 0.99 (95% CI 0.87 – 1.12)

**Supplemental Figure S82 – CV Mortality (HFpEF) NMA**

###

Indirect comparison: SGLT2 Inhibitors vs Incretin-based Therapies

HR 1.03 (95% CI 0.68 – 1.55)

Indirect comparison: SGLT2 Inhibitors vs Finerenone

HR 0.99 (95% CI 0.81 – 1.22)

Indirect comparison: Incretin-based Therapies vs Finerenone

HR 0.97 (95% CI 0.63 – 1.49)

**Supplemental Figure S83 – All-Cause Mortality (HFpEF) NMA**

Indirect comparison: SGLT2 Inhibitors vs Incretin-based Therapies

HR 0.78 (95% CI 0.39 – 1.54)

Indirect comparison: SGLT2 Inhibitors vs Finerenone

HR 1.05 (95% CI 0.90 – 1.22)

Indirect comparison: Incretin-based Therapies vs Finerenone

HR 1.34 (95% CI 0.67 – 2.68)

**Supplemental Figure S84 – CV Mortality or HF Hospitalization/HF Events (HFpEF) NMA**

Indirect comparison: SGLT2 Inhibitors vs Incretin-based Therapies
HR 1.09 (95% CI 0.85 – 1.41)

Indirect comparison: SGLT2 Inhibitors vs Finerenone
HR 0.95 (95% CI 0.84 – 1.07)

Indirect comparison: Incretin-based Therapies vs Finerenone
HR 0.87 (95% CI 0.67 – 1.12)

**Supplemental Figure S85 – CV Mortality or HF Hospitalization (HFpEF) NMA**

Indirect comparison: SGLT2 Inhibitors vs Incretin-based Therapies

HR 1.26 (95% CI 0.70 – 2.27)

Indirect comparison: SGLT2 Inhibitors vs Finerenone

HR 0.97 (95% CI 0.73 – 1.29)

Indirect comparison: Incretin-based Therapies vs Finerenone

HR 0.77 (95% CI 0.40 – 1.46)

**Supplemental Figure S86 – HF Hospitzalition/HF Event (HFpEF) NMA**

Indirect comparison: SGLT2 Inhibitors vs Incretin-based Therapies

HR 1.22 (95% CI 0.87 – 1.70)

Indirect comparison: SGLT2 Inhibitors vs Finerenone

HR 1.10 (95% CI 0.76 – 1.59)

Indirect comparison: Incretin-based Therapies vs Finerenone

HR 0.90 (95% CI 0.56 – 1.46)

**Supplemental Figure S87 – HF Hospitzalition (HFpEF) NMA**

Indirect comparison: SGLT2 Inhibitors vs Incretin-based Therapies

HR 1.68 (95% CI 0.84 – 3.37)

Indirect comparison: SGLT2 Inhibitors vs Finerenone

HR 1.10 (95% CI 0.76 – 1.59)

Indirect comparison: Incretin-based Therapies vs Finerenone

HR 0.65 (95% CI 0.30 – 1.41)

**Supplemental Figure S88 – Kidney Composite Outcome (HFpEF) NMA**

Indirect comparison: SGLT2 Inhibitors vs Finerenone

HR 0.75 (95% CI 0.50 – 1.13)

###

### **Funnel Plots - Incretin-Based Therapies**

**Supplemental Figure S89 - CV Mortality - Overall**

p-value: 0.4581015

**Supplemental Figure S90 - CV Mortality - T2D with ASCVD/High CVD Risk**

p-value: 0.1647197

**Supplemental Figure S91 - All Cause Mortality - Overall**

p-value: 0.8444469

**Supplemental Figure S92 - All Cause Mortality - T2D**

p-value: 0.3331513

**Supplemental Figure S93 - HF Hospitalization/Event - Overall**

P-value < 0.05

**Supplemental Figure S94 - Non Fatal MI - Overall**

p-value: 0.638177

**Supplemental Figure S95 - Non Fatal Stroke - Overall**

p-value: 0.9591325

**Supplemental Figure S96 - MACE Overall**

p-value: 0.1359424

**Supplemental Figure S97 - MACE - T2D with ASCVD/High CVD Risk**

p-value: 0.09055541

###

###

###

###

###

###

**Funnel Plots - SGLT2i**

**Supplemental Figure S98 - CV Mortality - Overall**

p-value: 0.8032152

**Supplemental Figure S99 - CV Mortality - T2DM**

p-value: 0.3821079

**Supplemental Figure S100 - CV Mortality CKD**

p-value: 0.8469662

**Supplemental Figure S101 - All Cause Mortality - Overall**

p-value: 0.4513651

**Supplemental Figure S102 - All Cause Mortality - T2D with ASCVD/High CVD Risk**

p-value: 0.3099471

**Supplemental Figure S103 - HF Hospitalization and Event - Overall**

p-value: 0.254139

**Supplemental Figure S104 - HF Hospitalization/Event - T2D with ASCVD/High CVD Risk**

p-value: 0.5661992

**Supplemental Figure S105 - HF Hospitalization - CKD**

p-value: 0.1810376

**Supplemental Figure S106 - Kidney Composite Outcome - Overall**

p-value: 0.909449

**Supplemental Figure S107 - Kidney Composite Outcome - T2D with ASCVD/High CVD Risk**

p-value: 0.2985505

**Supplemental Figure S108 - Kidney Composite Outcome - CKD**

p-value: 0.8775025

**Supplemental Figure S109 - CV Mortality or HF Hospitalization/Events - Overall**

p-value: 0.6389211

**Supplemental Figure S110 - CV Mortality or HF Hospitalization/Events - T2D with ASCVD/High CVD Risk**

p-value: 0.01737793

**Supplemental Figure S111 - CV Mortality or HF Hospitalization/Events - CKD**

p-value: 0.2936406

**Supplemental Figure S112 - MACE - Overall**

p-value: 0.341624
